# Supplementary material for: Genome-Wide Identification and Expression Analysis of CsCaM/CML Gene Family in Response to Low-Temperature and Salt Stresses in Chrysanthemum seticuspe
Source: Plants (Basel). 2022 Jul 1;11(13):1760. doi: 10.3390/plants11131760 (PMC9268918; doi:10.3390/plants11131760)
Supplement: Supplementary file 1 [file plants-11-01760-s001.zip › Supplementary File S1 Protein sequences of CaM_CML.pdf]

>CsCML1
MMSESNDSSRWSTLTLEEKVCGALVPLLAFAEIILFAFSGCFDFGGAKKQNKHKLLPFDHH
QLTRLASGSNFNVNEVEAL
FELFKSLSSSIIDDGLIHKEELRLALFNTPQGENLFLDRVFDLFDEKRNGVIEFEEFVHALSIF
HPYAPIEDKINFAFRL
YDLRQTGYIEREEVKQMLIAILTESEMNLSDDLLDVIIDKTFADADTDGDGKICKEEWKEF
ALRYPSLLKNMTLPYLADI
TTAFPSFVFHTSVEDAT
>CsCML2
MGCFHSTVKKPVPGHENPTLLASQTAFSVSEVEALFELFKSISSSVIDDGLINKEEFQLALF
KNRKKENLFANRIFDLFD
VKQKGVIDFGDFVRALNVFHPNAPQEDKISFCFKLYDMDGTGFIERQEVKQMLIALLCES
ELKLADDTIETILDKTFSEA
DVDQDGKIDKSEWHNFVTHNPSLLKIMTLPYLRDITTTFPSFVFNSEVEEIAT
>CsCML3
MGCFHSTVKKPVPGHENPTLLASQTAFSVSEVEALFELFKSISSSVIDDGLINKEEFQLALF
KNRKKENLFANRIFDLFD
VKQKGVIDFGDFVRALNVFHPNAPQEDKISFCFKLYDMDGTGFIERQEVKQMLIALLCES
ELKLADDTIETILDKTFSEA
DVDQDGKIDKSEWHNFVTHNPSLLKIMTLPYLRDITTTFPSFVFNSEVEEIAT
>CsCML4
MIEKIDVNNDGCVDIDEFGELYKSIMDDRENEEDMMEAFNVFDINGDGFIAVEELRAVLES
LGLKQGRKAEDCRKMIMKV
DVDGDGMVSFDEFKEMMRSGGFAAMAQN
>CsCML5
MFDKNGDGRITKQELNDSLENMNIYICDNDLVHMIEKIDVNNDGCVDIDEFGELYKSIMD
DRENEEDMMEAFNVFDINGD
GFIAVEELRAVLESLGLKQGRKAEDCRKMIMKVDVDGDGMVSFDEFKEMMRSGGFAAM
AQN
>CsCaM1
MADQLTDDQISEFKEAFSLFDKDGDGCITTKELGTVMRSLGQNPTEAELQDMINEVDADG
NGTIDFPEFLNLMARKMKDT
DSEEELKEAFRVFDKDQNGFISAAELRHVMTNLGEKLTDEEVDEMIREADVDGDGQINYE
EFVKVMMAK
>CsCML6
MCPTGTLIPLATKQAELRSAFNVMDVDHDGKISHDDLKRFYADYAVNDNEIIIGTMMKET
DLNKDGYVEYEEFEKMLFLR
RSSNVMEEVFNEIDKVGDANDDENDGVTFEGFLKILAI
>CsCML7
MNTSRPCPSLKSLFYEVGGMLRCCNSTNEYERLDSELERKMIEVKKRYVPGNSSIRSINSII
LKFPQFRQGLEEIRGVFR
QFDVDSNGTIDREELRKCLHKFQFDCTEDEINDLFESCNLGRNQGMKFNEFIVVLCLIYLL
TCTSSSNHTATTMGSPELK
STFDTIIEAFLFLDKNGDGKLDKKDMTKAMNDDFPKEKSPNHITMSRFREMDWNKDGKV

GFREFLFSLINWVGIDSNNEV
HVEVIREQKP
>CsCaM2
MADQLTDDQISEFKEAFSLFDKDGDGCITTKELGTVMRSLGQNPTEAELQDMINEVDADG
NGTIDFPEFLNLMARKMKDT
DSEEELKEAFRVFDKDQNGFISAAELRHVMTNLGEKLTDEEVDEMIREADVDGDGQINYE
EFVKIMMAK
>CsCML8
MKFFSTSNVSIPFSLERSEILVVKHDSNLSKAHVSFTNKNIVDDACVQRDDVELIMANLGV
FCHPEGEKVPEVMTSNDLF
NIFEDEQPRLDEVKQAFDVFDQNKDGFIDASELQRVLVVLGLKERSNIEDCRKMIRAFDEN
ADGRIDFDEFVKFMEATFC
>CsCML9
MFQPDVDEMRRVFNKFDKNKDGKISKEEYGSAVGVLGSKNTKSDVIKTFQAIDTDGDGF
VDFNEFMEAQKSEGGVKTADI
KSAFKVFDLDGNGRITAEELVQVLRQLGERCSLESCRKMIKGVDADGDGMIDVDEFMGL
MTRNMKLA
>CsCML10
MADADHQANLERIFKKFDTNGDGKISSSELGEALKTLGSVSPEEVQRMMREIDTDGDGFI
SYQEYIDFCNANKGLMKDVS
KIV
>CsCML11
MADEDKAECDRIFGAFDKNGDGKISAAELGESLTKLGSVSPEEVQTMMDELDTDGDGNP
SISLGYKRNSNPIIRVSIHHL
V
>CsCML12
MSIMIAEFIQYLFSHVFLNMIIYPQLGYFLDDSKIHVEKRNKDSRLPKRLPSFKDGSVRGDE
VQTVMGNLGIFCNSKGES
FPERLSSNDLFNMFEEEHPELDEVKGAFDVFDENKDGFIDAKELQRVLSALGLKDRAAM
DDCKKMIRVFDENDDGRIDFD
EFVKFMEGTFC
>CsCML13
MDKEQQYKRVFGHLDANGDGKLSPPELQICLGKIGGELSLEEAEIAAALMDSDGDGLLS
MEDLVNVVESANEEEKIDDLK
MAFKMYEEKEGCITPKSLRRMLSKLGESRTVNDCKVMINKFDVNGDGVLNFDEFRIMMA
>CsCML14
MDKEQQYKRVFGQLDANGDGKLSPPELQSCLGKIGGELSLEEAEIAAALMDSDGDGLLS
MEDLVNVVESANEEERINDLK
MAFKMYEEKEGCITPKSLRRMLSKLGESRTVNDCKVMIARFDVNGDGVLNFDEFRIMMA
>CsCML15
MDKEQQYKRVFGHLDENGDGKLSPQELQICLGKIGGELSLEEAEIAAALMDSDGDGLLS
MEDLVNLVESANEEERINDLK
MAFKMYEEKEGCITPKSLRRMLSKLGESRTVNDCKVMIARFDVNGDGVLNFEEFRLMM
A

>CsCML16
MDKEQQYKRVFGQLDANGDGKLSPSELQICLGKIGGELSLEEAEIAAAWMDSDGDGLLS
MEDLVNVVESANEEERINDLK
MAFKMYEEKEGCITPKSLRRMLSKLGESRTVNDCKVMIARFDVNGDGVLNFDEFKIMMA
>CsCML17
MDKEQQYKRVFGHLDTNGDGKLSPPELQICFGKIGGELSLEEAEIAAALMDSDGDGLLSM
EDLVQAVEGANEEEKINDLK
MAFKMYEEKEGSGCITPKSLRRMLSKLGESRTVNDCKVMIAKFDVNGDGVLNFHEFREM
MV
>CsCML18
MDKEQQYKSVFRHLDKNGDGKLSPPELQTCIGKVGGELSLEEAEMAAALIDSDGDGLLS
MEDLVKVVEGANEEEKINDLK
MAFKMYEETEGSGCITAESLRRTLSKLGESKTVDDCKIMIAKFDVNGDGVLNFDEFREM
MA
>CsCML19
MATETTNPTTTTQQTSSSVNLTNIEEVKKVFNRFDTNHDGKISSSELISIMKSLGSNISEDEV
RQMMTKIDTDNDGCITL
EEFAGFCKDDTADDGGMKELHEAFELYDLNNNGLISSSELHQILTRLGESVSVEDCVGMIK
SVDADGDGFVNFEEFKKMM
SNGKTEP
>CsCML20
MGLKNLFKGKKKSTQNNNNVVVGVAVRNPNNTSSPVLSRSSSCNSRARIEEELTQVFRKF
DVNGDGKISASELGSIMGSL
GQKPTETELENMIKEVDADGDGFIDLHEFIALNTKDIDSNELLENLKEAFCVFDIDKNGSIS
AEELQKVLGRLEESCTIE
ECRKMISGVDVDGDGMISFDEFKVMMMSGNGFVSKKNNNREMKEE
>CsCML21
MSKVSVILYITVAILILILISNKNTNNTSSNRHNRRHRRLKLRSNFTTTPIPTTPIPTTPSDHHI
SFDPLIADIERKRED
KQWEQTHYFNKDHGDVHDHGGDDVAPGMEGQPEWEDFIDAEDYLNDEHKFNITHRLVL
LFPRIDVDPADGFVSEHELTQW
NLEQSQREVLHRSQREMELHDKNRDGLVSFHEYSPPSWVRDTDNNSFGYDMGWWKEEH
FNASDIDGDGFLNLTEFNDFQH
PADTKNPRLLQWLCKEEVRERDTDKDGKVNFKEFFHGLFDLVRNYDEESHHNSSHESNN
DSLESPAKRFFSELDKDADGF
LSDVELLPIIGKLHPSERYYAKQQADYIISQADTDKDGRLSLTEMIDSPYVFYSAIFNEDDE
EDYEYHDEFR
>CsCML22
MKTMTLKECCCFLKKLRNPSRIIQHALLASETSFTINEIEALYDLFERLSYAIIEDGRIHKEEF
RLALFSNSTMQNLFAD
RLFESFDIKKNGVIEFDEFVRSLSIFHPNAPESDKIEFMFRLYDLRHTGFIEREELKEMVVAL
LSEMDVSVSDEDIEAIL
DKTLLDADLNGDGKIDLEEWKIFISKNPSILKNMTLPLLREITQAFPNFVLNTQVQNLEVE
V

>CsCML23
MSSDKEPTVKLDDEQLSELREIFRSFDRNNDGSLTQLELGSLLRSLGLTPSSDQLDTLIQKA
DTNSNGLVEFSEFVALVA
PELLPAKSPYTDDQLKQLFKMFDRDGNGYITAAESFLAPLLQLLLIILFRDFGYSFAYLFVF
R
>CsCML24
MGCCYSAMGVPVVAREDSRTLASQTTFSVSEVEALFELFKSISSSVIDDGLISKEEFLLALF
KNKRKKNLFANRIFSLFD
VKRKGVIDFGDFVRSLGVFHPRAPLEDKINFSFKLYDLEGTGFIERREVKQMLIALLHESEL
KLADEAIEMILDNTFSEA
DGDRDGKIDRSEWHAFVTKNPSLLKTMTLPVLRDITSAFPSFVFNTQVDEISSPQVEEVEIS
SSQVEEIEISSSQVEEIP
K
>CsCML25
MDKEQQYKRVFRHLDVNGDGKISPPELQICIGKIGEDLSLEEAEIAAELMDSDGDGLLSFD
DLVKVVESANEEEKVKDLK
LAFKMYEEMEGCGCITPKSLKRMLSKLGESRTVDECELMIKKFDLDGNGVLDFQEFQDM
MS
>CsCML26
MDKEQQYERVFKHLDVNGDGKISPPELQICIGKIGEDLSLEEAEIAAELMDSDGDGLLSFD
DLVKVVESANEEEKVKDLK
LAFKMYEEMEGCGCITPKSLKRMLSKLGESRTVDDCELMIKKFDLDGNGVLDFQEFQDM
MS
>CsCML27
MESNKVPKSFGCFPQKGLVLRLSSFRSKSNNTSNSTSPTLMSPRSPKPNTNNREQEFRSVF
ARFDADNDGKISALELRSY
FGSIGEYMSHQEAQGVIDDLDTDGDGFIDFQDFMRLMKVSNEKDDVKAAFEMFEYEKGC
GQISPKSLQKTLSRLGDSKTY
DECLQMIKMFDTHGKGAVDFNEFQQMMTA
>CsCML28
MAQTDVEKVFKKFDVNGDGKISITELGSILAALSGAVTSETELKSVMKEIDTDGDGFIDFD
EFVAFHNGNGEEEESKELR
EAFDLYDEDKNGKISANELHSVMKRLGEKCSLKDCKKMIQSVDVDGDGCVNFEEFKKM
MNK
>CsCML29
MPAGYSWAKPPAMTLVHQNLSMGCFPRLVTPAFESYSCRWTIRTSALFVPQQRLFHDSAN
NILILLAILSLLSRWGYLEK
QNKAQPNIKSCFMEWRLKQVEAPLTDDQIKGLVSKFDTNGDGKISRRELRVGLKSLGLHF
AGLRAMGAVRHADANGDGVI
SDEEINELAKYISKWGISVT
>CsCML30
MRQSWCLRQVEAAPLTEDQIKGLLRKFDANGDGKISRRELRAGLKSLGLRFACFRARRAL
RYADANGDGVISDEEINELA
RYVSKWDISLT

>CsCML31
MTRKSWCLKQVEMPMTDDQIKGLVNKFDTNGDGKITRRELRVGLRKLGLRFATFRAMG
AVRYADANGDGVISDEEINELA
KYISKWGISVN
>CsCML32
MSLSEQFHIIGDLHVFSTQFSNLCLHKAKFIIDTPPTKTCTAATAHEKAHATNLGGVENGPG
KKKALGVNPKTMMERLGT
FWDPDDHREALDMDEMVNLFVEDEPSLDEVKQAFGVFDKNNDGYVDAKELQNVLSNM
GFLHICESDCRRMIVSYDADKDG
KLSFREFLKVVEDGFR
>CsCML33
MSTLPEDQLNQLKEIFTRFDLDKDGSLTHLEVAALLRSLGLKPSGDQIHKLFKNMDSDGS
GTVEFDELVNSMSSHMMTEE
ILVNQQQLMEIFRSFDRDGSGFITPAELAKSMTKMGQPLTYRELSEMVRDADTDGDGVISF
KEFQGIMARSAADSLGFSL
>CsCML34
MVVVHLDPILIHYNPGNDGRGGELVIIGNIEIELTDIGGKVMKAAKPALTFDDIRRLALNSP
FTVNEVEALRELFNKLSS
SIIDDGLIHKEELQLALLNSAGGENLFLNRVFDLFDEKQNGVIEFEEFIHVLSIFHPYAPIEEK
IDFAFRLYDLRKTGYI
EREEVKEMIVATLKETGMRLSEEILEEIIDNTFADADADMDGRINKEEWRDFVIQRPQLLK
NMTLPSLRDVTTAFPSFIF
NTGVDD
>CsCML35
MDPAELRRVFQMFDRNGDGKITKQELAKSLENLGIYIPDDDLAQMIEKIDVNKDGFVDME
EFGELYQTILGERDEEEDMR
EAFNVFDQNRDGFITVEELRSVLSSLGLRQGRSIEECRLMIKKVDEDGDGMVNYKEFKQ
MMKAGGFAGLET
>CsCML36
MSGYPQNPSGYGTPPAQPYGAPPQQPYGQPPQQSYGQPPPAQPYGAPPAQPYGAPAQPYG
APAQPYGAPSAPYGQNPNKP
PKENKPQGSGGYGAAPPPGGAYGQGGAPYGSPFAALLPSTFPPGTDPNVVACFQVADQDG
SGVIDDKELQRALSSYNQSF
SLRTVHLLMYLFTNTNTRKIGPKEFIQVFYSLQNWRANFEKFDRDRSGKIDINELREALMS
LGFAVSPVVLDLLVSKFDK
SGGKNKAIEYDNFIECCLTVKGLTEKFKEKDTSYSGNATFTYEAFMLTVLPFLIA
>CsCML37
MKLSGRINPKNIFRSKSHKKDSVSRSESSSFSSSITTSSGSPERSKGATTPTTVLPTQPLTRSD
LEALLRRITNDEAEVK
LMLDEVEGDGEVEGTMTEFGEEEMRGAFEFFDSDGDGMITADELFQVFKVINGDDGCTL
EESTIHIQSLLSSSNLLQISI
FRCIRVIRHVKGTLKRERE
>CsCML38
MKEAFELFDTDGNGTIDAKELSNAMRALGFEMTKEQLDQMIADVDRDGSGAIDFDEFVY

MMSDKIGERSNKQELTKAFNI
IDHDKNGKISILDIKNIAKELSVRFTDAEIHAMVEEADRDDDGEVSKEEFMRMMQTTSYG
Y
>CsCML39
MRALGFEMTEEQINQMIADVDKDGSGAIDFDEFAYMMTAKIGERDSKQELTKAFEIIDQD
KNGKISVADIKKIAKELGEH
FTDDEIHEMVEEADRDHDGEVSAEEFMRMMKRTSYGY
>CsCML40
MIYNYYNKSILEDLSGSFLLFSTRSDIFEPPVNDDRPSQTRSTRTFYRGKGEAVIFIKNSLQK
MGVRRESSRSDQPRGRH
HGLNPQKKQEINEAFDLFDTDGSGTIDAKELNVAMRALGFEMTEEQINQMIADVDKDGS
GAIDRDEFEYMMTAKIGERDS
KQELKKAFEIIDQDKNGKISFADIKKIAKELGEHFTDAEIHEMVEEADCDDRDGEVSSEEF
MKMMKRTSYGY
>CsCML41
MSKMSFLDIQYNISKRKFLRKPSRMFSSSERQPSGLPMFQPNVNEMRRVFDKFDRDKDGK
ISRGEYKAILRALKQGGTER
DIQKIFEVADLDGDGFIDFKEFMEVQKKGGAVKAVDVQSAFKTFDLDGDGKISVEEVYEL
MKRLGERCSLQDCRKMVRGV
DSNQDGVIDIDEFMTMMTQNMKI
>CsCML42
MSNSSERKAELKSVFATFDKNKDGFITKQELSDSLKNIGISTSEKDVVEMVQRVDVNGDG
LIDFDEFCELFESMMSKEDM
QGSKVGDDDDHEDGDLRDAFNVFDGDKNGLISVEELGLVLDSLGFKEGKKLEDCKKMIS
KVDIDGDGMINFNEFKSMMKS
GVSLISVS
>CsCML43
MSSPPSYNDLYRLFKKLDQNGDGLVSPHELQWLLDTMKVSSSVDDLRYLTGKTNINFTEF
LEFYGTITKEEKVTCDDESE
SDLFKAFEMFDKNRDGFICNEELMEALTRLGLWDDKSNMDVKSMIKAYDANCDGFIDFH
EFKKMMA
>CsCML44
MSQCLEGIKHLCTSLLSCCDLELTKQSQGLDDPAILASQTVFSVSEIEALYELFKKISSAVND
DGLINKEKFQLALFKTN
KKESLFADRVFDLFDTKHNGILGFEEFARALSVFHPNAPIDDKINFSFQLYDLKQQGFIERQ
EVKQMVVATLAESGMNLS
DDVIESIIDKTFEEADTKHDGKIDQEEWRNLVLRHPSLLKNMTLQYLKDITTTFPSFVFHSR
VEDM
>CsCML45
MAASKTQLEFHDHLPLIEEKLGGDGLIGELCKGFELIMDPNKGVITFDSLKNNASFLGLQD
LSDDDLMSMLKEGDYDGDG
ALNQMEFCVLMFRLSPDLMDQSEFLLEEALEQEVKNSYQ
>CsCML46
MVQEEFQLALFRNRNKRNLFADRIFDLFDLKRNGVIECGEFVRSLGVFHPDAPTEDKIKFA

FRLYDLRQTGFIEREELNE
MVIALLDESDLVLSEDVIEMIVDKTFSDADIKGDGKIDEEEWKQFVAHNPSLIKNMTLPYL
KDITLAFPSFVVTSEVEDS
EV
>CsCML47
MGCMCSSGIKHTPGYEDPAILANETPFTVSEVESLYELFNKLSSSIIDDGLIHKEEFQLALFR
NRNKRNLFADRIFDLFD
VKHNGVIERGEFVRSLGVFHPDAPTEDKIEFAFRLYDLRQTGFIEREELNEMVIALLDESDL
VLSEDVIEMIVDKTFSDA
DIKGDGKIDEEEWKQFVAHNPSLIKNMTLPYLKDISLAFPSFVLTSECEVEDSEV
>CsCML48
MELTSVATAATTGGILSMEVKYFIILYMFVEWVKLLHDCYFLLARPMWRFISTTENTTCRP
TPSTVAQAHDTIQVFHAYN
SRKKSLGVDLEIVLKRLGMFCDHDDKGQIIGSDEILGLFDEDEPSLDEVKEAFSAFDRNKD
GFIDAKELQHALSEMGCIQ
ISESNCRLMIGGYDVDQDDKISFREFLKLMEDCF
>CsCML49
MGCISSTPKEFKKLPAYDPVALAAETPFTVNEVEALYELFEKLSSSVVDDGLIGKDEFNLA
LFRNHSKRNLFADRIFDLF
DVNRSGHIDFNEFVRSLSVFHPKAPQADKVLYAFRLYDLRRTGFIEREELKDMVKALLSES
DLVLSDEIIESMVDKTFAE
ADYKGDGKIDQEEWREYVDKNPSLLKNMTLPHLMDITLRFPSFVMNSQVEETQSTD
>CsCML50
MSYNPSGYNYGAPPPSQPYYSTPYAAPSPYGQPQQPAFTSPYGYGTSTFPPGTDPNVIACF
QVADIDRSGVVDDKELQIA
LSSYNQSFSIRTVRLLMHHFTNTNTRKIGPKEFTQVFYSLQNWRAIFEKFDRDRSGHIDTW
ELREALMSLGFTVSPVVLD
LLVSKFDKTGGYKKAIEYDNFIECCLIVKGLTEKFKEKDTMYSGNATFTYEAFMLTVLPFL
IV
>CsCML51
MGNTSSMLTQYDIEEVQEHCNNTFSQQEIVSLYQRFCQLDRNSGGFISADEFLSVPEFAVN
PLSQRLFRMIDGLNFKEFV
AFLSAFSSRATLQHKVEFIFKVYDSDGNGKVAFSDLLDVLRDLTGQFISEQQRELVLTQVL
EEAGYKKDSLLVLSDFMKI
LGNTGLKMEVEVPTNPSKYVGIISILNDVIGTLKIHKPYSSLSQTPLFNYYLTTQLTDRKWL
DLV
>CsCML52
MSCLEGIRHLLAPLLRCFNLESFTKTNPLEDPEVLARETVFSVSEIEALYELFKKISSAVTDD
GLISKEEFQLALFKTNK
KESLFADRVFDLFDTKHNGILGFEEFARALSVFHPNAPVDEKIEFSFQLYDLKKQGFIERQE
VKQMVVATLAESKMKLTD
DVIENIINTTFQEADTKHDGKIDMEEWRSLALRHPSLLKNMTLHYLKDITTTFPSFVFHSR
VEDS
>CsCML53

MGGKLTKADESPKTSVPTTKLEAKILETIRRRESKGTSMKSFNTIILKFPKIDASLRKCKAIF
EQFDEDKSGTIDPKELN
HCFRKLEIDFTDEEISDLFKECDINHDMGINFKEFIVLLCLVYLLKNDPVSPHSSSRMGMPE
LQAFETLVDSFVFLDKNK
DGYVSRSEMVDAINETTTGERSSGRIAMRRFEEMDWDKNGMVNFKEFIFAFEKWIGIEDG
EEDEEEVADEQE
>CsCML54
MASTNNMQSEFQDYLPLMADKLGGDGLIQELCNGFQLLMDQDKGVITFDSLKKNSSVLG
LEGLSDDEVMSMLKEGDFDGD
GALNQMEFCVLMFRLSPNLMDQSEYLLEEALEQELNNFQY
>CsCML55
MTTNYNSSFHDFLPLMADKLGGDGLVDELCKGFELLMDQDKGVITFDSLKKNSSVLGLE
GLSDDEVMSMLKEGDFDGDGA
LNQMEFCVLMFRLSPNLMDQSEYLLEAALEQEFNYL
>CsCML56
MKAAITGLFNTQEVAQEYVVTGVGLLAGSTIFLLTLLWGTCIIIGSQKFSSESGASTSVDPT
QCPNKKFFSFLTSSGVTT
DPETCTAAQIMLLSVIPFLFLLIPKLFGMTYAPHGYIFLIALPVSVTFLLVYFIYQVFEPSIQK
RRLSYVKHEHLVLDIL
KHLQEQIPENILAEDGSVNLPAIKSLFKKIDQDGDDIISFSELKELLESIKFRQLKSDKQKTF
DQLIKEFDSDGNAQVSL
DEFIHRFTEWLDEAKNELSEVVKPLVQTKRNEDDMTQVLVSEIIGNAKSSPLGKFYKEDG
TPDISAIKKLFRSLDVNKDG
SVSLTELKKLTMHVNLGETSWNVDETTSRIMQNLDTNGDKEIDEQEFVDGFEKKLVNITN
DRSKTSGPKDVSRKACKKWK
GDNVDRSVWGWTKAIMLLVLGIAMLALMAEPLIHSVQNVSNSAAMPSFFISFILVPLATN
ARAAISAIRTASQGKERTTS
LTFSEQERINCFDQSKASMVEDDKHDNASSSASIREGNVSLQCPKLTDTNYTTWALMMKT
ILKAYGLWKVIDGMKETSG
>CsCML57
MGTSHTSPSFKSLSNKVGVMLCCCNSQNRYERLDNKLERKMMEVKQNSIQGQTSFRSIDS
IILRFPRFKEGLKEIQGVFE
LYDEDSNGTIDNEELKRCLQKLEFHCTEQEIRDLFESCDVDGSNGIQFNEFIVLLCLIYLLD
GPSSSSHVTSTVGSPELK
ATFDTIIEAFLFLDKNGDGKLNKKDMMKAMNEDFPMEKSPTHITKTRFKEMDWNKDGK
VSFREFLFSLINWVGLESTDEV
PETVF
>CsCML58
MSYSGYNPNTTIPSAPPAPPSQSHQPATAYPYQQPPPQNYNNQQTYNPSGYGGGYGSQYG
SYPPQQTVSFPPGTHPEVIR
SFQAVDLDRSGFIDAKELQQALTQAYLKFSSRTIKLLMFQFRNPTDPTRTGPKEFAELWSCL
GQWRAIFERFDRDRSGKI
DLAELRDALYSLGYAIPPSVLQLLISKYDDQSGRRVDLSFDSFVECGMIIKGLTEKFKEKDT
RYTGSATLSYETFMTMVI

PFLVAE
>CsCML59
MAFGSQSNVLDPPPKFDMYKYTFVMTDADLEGVVAGYGHPWISEDGVAPDKPLDSAVLS
RLTQFSAMNKLKRMALRVIVS
KLSEEETAGLKQMFKMIDTDKSGYITFEELKAGLKRFGSTLNESEIYDLMQSADIDNGTID
YEEFVASTLHMNKVDRDDH
LFAAFSYFDKDDSGYITLDELQQACKEFGLDDVHLEEIIKEADQNNVRFSLP
>CsCML60
MAEQLTEEQIAEFKEAFSLFDKDGDGCITTKELGTVMRSLGQNPTEAELQDMINEVDADQ
NGTIDFPEFLNLMARKMKDT
DSEEELKEAFKVFDKDQNGYISAAELRHVMTNLGEKLTDEEVDEMIREADMDGDGQVN
YEEFVRMMLAK
>CsCaM3
MRSLGQNPTEAELQDMINEVDADGNGTIDFPEFLNLMARKMKDTDSEEELKEAFRVFDK
DQNGFISAAELRHVITNLGEK
LTDEEVDEMIREA
>CsCML61
MSKSNDYKRVFDHFDEDSNGMVSPSELHRRVGMICHEQVLIEDVQVIVESLHGSKVDGH
ELGFDDFVSLMESDNEDEKVE
DLRKAFRLYENDGNDCITPKSLNRMLDRLGESRSVDECVGMINQFDLNGDGVLNFEEFK
AMML
>CsCML62
MNSCEVLVKSLQASVQSLLTHVSITWNKLGSISTSLDLNETVKKEFMTLGEINVIMGQLGL
QQRCSDQDSNIDILSVFDD
EEPTLEEVKVAFDVFDENSDGFIDENELRDMLCKLGKQENAMLKECRSMIKGFDVNGDG
LIDFDEFVRLMETCSF
>CsCML63
MGLKNLFNRKTKNTTKDNTTMVEETIATNTTLPVVPRQQTKEQQLEQVFKKLDVNNDG
KISYSELGSVMGSLAGNQPTDD
ELKKMIMEVDKDGDGFIDLEEFIELNTKVDSSELLELIEKAFSMFDVDKNGLITVEELLRV
MRSLHEDYSIEECKKMIAG
VDQDGDGMINLNEFKVMMMSGVRSDVSES
>CsCML64
MNTGEPSTSSSPVKFFRPPTTISPPSSSPLPTPCTDDIHQLFNYFDENGDGKITATELQNRLK
TVAGDEVQLSDEEAEMA
VRSSDADGDGVLGFDDFTKMMKEGAEEELREAFRMYSAKSGTVITANSLRRMLRRLGQS
TVTVEECKGMIGRFDVNGDAV
LDYDEFRAMMS
>CsCML65
METQTPTSKHAPLLKSCSNGSFRLRSPSLNSLRLRRIFDLFDSNHDSFITIEEITRALTLLGLD
TNASDLDTMIKSYVHP
GNVGLTYEDFVTLHRSINDLFFGMDEVEEAVGSKEDQEEADLNEAFKVFDENGDGFISAA
ELQMVLGKLGFTEAIEMGRV
KMMISSVDLNHDGCVDFSEFKDMMRVLQ

>CsCML66
MCDDSGVPHRFVLDIQVLQEISLLDSVAETAVEMMNILKKIKKNVKTASKPISNKIKHDPE
KKPKSIVDVGCGIGGSSRF
LAKKYGAKCRGITLNPVQAERAQVLADAQGLGDKPLSEVPHQGIPRVSILVLDSFQAQLD
ALIQKGDTNSNGLVDFSEFV
ALVAPELLSAKLPYTDDQLKQLFKMFDRDGNGYIMAAELAHSMAKLGHPLTAEELTGMI
KEADTDGDGRINFQEFSCAIT
SAAFDNSFS
>CsCML67
MCPTGTSLFPSRNITNLRSAFDILDVDHDGKISHEDLKTSYSHADDNIIGTMMKVADSNNN
GYVEYDEFEKVVLKTDGSN
VYGVMEDVFKAMDCDGDGKVGYGDLRSYLNMAGLDVNDDEIKAMIRFGGGGDYDDG
VTFDGFIKILSL
>CsCML68
MARVLAVQQRKQLLEIFKQFDMDSDGSLTYLELAALLRSIGLNLSGDQIYTLFNKIDSDGN
GKVTFEAFVDAMTIDVKTE
EIVIDQRQLFEAFRSFDREGNGFITPTQLAISMAKMGYPLTYHELVAFIEGLFVGLTRTLERL
VSELADFCIELCRTLEV
KVGMQRLTSHPTYVVF
>CsCML69
MSSNNEANFHDFLPIMADKLGGERLMDELCNGFRMLMDPIKRVVTFDSLKKNSAVLGIG
DLTNDDVLSMLKEGDLDGDGV
LNQMEFCVLMFRLSPDLMKQSWFLLEEALRTIVEFESN
>CsCML70
MLTLDPKKRITSAQVLEHPWIREDGEASDKPIDSAVLSRMKQFRAMTKLKKLALKVIAQN
LTTEEIQGLKSMFMNMDTDK
SGTITIEELKTGLARLGSKLTESEVRQLMDAADVDGNGSIDYIEFITARMHRHKLEREEDL
YKAFQHFDTDGSGFITRDE
LENAMKENGLGDEATIKDIISEVDTDNDGKINYEEFCTMMRSGTQGAKLF
>CsCML71
MLTLDPKKRITSAQVLEHPWIREDGEASDKPIDSAVLSRMKQFRAMNKLKKLALKVIAEN
LTTEEIQGLKSMFMNMDTDK
SGTITYEELKTGLARLGSKLTEAEVRQLMDAADVDGNGSIDYIEFITATMHRHKLEREEDL
YKAFQHFDTDGSGFITRDE
LENAMKENGLGDEATIKDIISEVDTDNDGKINYEEFCTMMRSGTQGAKLF
>CsCML72
MTQCLEGIRHLLASILRCCDLELYKQSRGLDDPEILARETVFSVSEIEALYELFKKISSAVID
DGLINKEEFQLALFKTN
KKESLFADRVFDLFDTKHNGILGFEEFARALSVFHPNAPIDDKIEFSFQLYDLKQQGFIERQ
EVKQMVVATLAESGMNLS
DDVIESIIDKTFEEADTKHDGKIDKEEWRSLVLRHPSLLKNMTLQYLKDITTTFPSFVFHSR
VEDT
>CsCML73
MGLKNLLNRKKKKKTGDDNSTHESEPQSEPASQTTNTSNNINAEASQAKSLDSRVRIEEE

LEQVFNKFDVNADGKICAAE
LGSIMGSLGHHPSQEELKNMIKEVDADGDGFINLQEFIELNTKDIDSSEVLENLKDAFSVF
DIDKNGLITAEELLNVLSS
LGENCTITESKKMIAGADRDGDGMINFDEFKDMMMSGSRFDSGLQKHEIAKED
>CsCML74
MSPETSNQSPSVFPTDKEEIKTIFNRFDTNGDGKISEDELINVLKSLGSDTSPEEVKRILTET
DTNSDGFISLDEFVVFC
KGIAGECDGDGLNDLKEAFKLYDQDNNGVISASELHQILSGMGLNYTLKDCENMINSVD
SDGDGCVDFEEFRKMMSKN
>CsCML75
MAEKLTDDQITEFRQAFSMIDKDSDGLISTEDLIGVIQTLNENATNEEVKEMMNEVDTNEE
GTIDFHDFLNIMSKRVKEN
ASDELKEAFKVFDRNQDGYISPDELRNVMINLGERLKDEELEQMMREADLDGDGVISYD
EFVRVMMNSS
>CsCML76
MFDHDGDGNITIQELSKSLESLGMVIPEKDLENMIKHIDTNGDGSVNMEEFKGLYETIMEE
KDEEEDIKEAFNVFDKNGD
GFISVEELMSVLTSLGFRQGRTIEDCQLMVKKVDEDGDGMVNYKEFRQMMKGGGFASM
>CsCML77
MGNDDHLKDAFAFFDKNKSGYIEIEELREALSDEDEANSEEVISAIIHDVDTDKDGKISFEE
FTAMMKAGTDWRKASRQY
SRERYNNLSLKLFQDGSLVSANEGR
>CsCaM4
MADQLTDDQISEFKEAFSLFDKDGDGCITTKELGTVMRSLGQNPTEAELQDMINEVDADG
NGTIDFPEFLNLMARKMKDT
DSEEELKEAFRVFDKDQNGFISAAELRHVMTNLGEKLTDEEVDEMIREADVDGDGQINYE
EFVKVMMAK
>CsCML78
MKLPAKINPKHIFRSRKHKTVTRSDQSSFSSSNTTSSDSPESSHHRRKANTSGVTTPTSVLP
SSADDYSDLQLDLIQAFR
FIDTDGDGKITTQELETILNRIVRSEPLIQSELKSMLTEIDSNGDGVITLEEFGAVSEAFGPAV
GDGELKEVFEFFDRDG
DGKITADELYEVFVSLGDGKVTVEECVGMIKSVDVNGDGFVCFDDFRSMMEQR
>CsCML79
MSTYKDRSCRATSPMPKSLQTRIRNLLKRVNFLTKVNNFKKKPPKNPCVLDAISSFIAMDV
SNQLKQVFKFFDIDGDGKI
SQVELTNVLLTFGQEKSMATKEAQGILKEVDFNGDGFIDLDEFMTIMDGSKPVFASSKEDN
GDDDLRNAFMVFDSDKNGL
ISAKELQSVLTSLGCSNSKLGQCRKMIKGVDKDGDGFVDFDEFKSMMSIGIK
>CsCML80
MAEKSYQDLLPIMAEKLELTTFMEELCSGFRLLADENTGLITPESLRKNSSILGMEGMSKE
DSEGMVIEGDLDGDGFLNE
TEFCILMVRLSPEMMQDAEMWLDKAIEDEIKNVSTSLPDNKV
>CsCML81

MASTNNMQSEFQDYLPLMADKLGGDGLIQELCNGFQLLMDQDKGVITFDSLKKNSSVLG
LEGLSDDEVMSMLKEGDFDGD
GALNQMEFCVLMFRLSPNLMDQSEYLLEEALEQELNNFQY
>CsCML82
MSQLKSSSFRLRSPSLNSVRLRRIFDLFDTNHDELITVDELSRALILLGLDTNMNELDSMIN
TFIQPGNAGLTFDDFHAL
HKEIDDLFFRLDDNDDLGNQDEDNDEANGDKQEEADLTEAFKVFDEDGDGYISATELQT
VLVKLGFAEGNEIGSVERMIS
SVDRNHDGRVDFTEFKDMMRNVIVLK
>AtCaM1|AT5G37780.3
MADQLTDEQISEFKEAFSLFDKDGDVFVLSDLGFDFKRLSNCLETTPELSHGCITTKELGT
VMRSLGQNPTEAELQDMIN
EVDADGNGTIDFPEFLNLMAKKMKDTDSEEELKEAFRVFDKDQNGFISAAELRHVMTNL
GEKLTDEEVEEMIREADVDGD
GQINYEEFVKIMMAK
>AtCaM4|AT1G66410.2
MADQLTDEQISEFKEAFSLFDKDGDDSISDSGDSCGCITTKELGTVMRSLGQNPTEAELQD
MINEVDADGNGTIDFPEFL
NLMAKKMKDTDSEEELKEAFRVFDKDQNGFISAAELRHVMTNLGEKLTDEEVEEMIREA
DVDGDGQINYEEFVKIMMAK
>AtCaM5|AT2G27030.3
MADQLTDDQISEFKEAFSLFDKDGDGCITTKELGTVMRSLGQNPTEAELQDMINEVDADG
NGTIDFPEFLNLMARKMKDT
DSEEELKEAFRVFDKDQNGFISAAELRHVMTNLGEKLTDEEVDEMIKEADVDGDGQINYE
EFVKVMMAKRRGKRVMAAKR
SSNSAEYKEKNGRRKSHCRIL
>AtCaM3|AT2G27030.3
MADQLTDDQISEFKEAFSLFDKDGDGCITTKELGTVMRSLGQNPTEAELQDMINEVDADG
NGTIDFPEFLNLMARKMKDT
DSEEELKEAFRVFDKDQNGFISAAELRHVMTNLGEKLTDEEVDEMIKEADVDGDGQINYE
EFVKVMMAK
>AtCaM2|AT2G41110.2
MADQLTDDQISEFKEAFSLFDKDGDGMLHPPFPSIIVGCITTKELGTVMRSLGQNPTEAEL
QDMINEVDADGNGTIDFPE
FLNLMARKMKDTDSEEELKEAFRVFDKDQNGFISAAELRHVMTNLGEKLTDEEVDEMIK
EADVDGDGQINYEEFVKVMMA
K
>AtCaM6|AT5G21274.1
MADQLTDDQISEFKEAFSLFDKDGDGCITTKELGTVMRSLGQNPTEAELQDMINEVDADG
NGTIDFPEFLNLMARKMKDT
DSEEELKEAFRVFDKDQNGFISAAELRHVMTNLGEKLSDEEVDEMIREADVDGDGQINYE
EFVKVMMAK
>AtCaM7|AT3G43810.2
MADQLTDDQISEFKEAFSLFDKDGDGCITTKELGTVMRSLGQNPTEAELQDMINEVDADG

NGTIDFPEFLNLMARKMKDT
DSEEELKEAFRVFDKDQNGFISAAELRHVMTNLGEKLTDEEVDEMIREADVDGDGQINYE
EFVKVMMAK
>AtCML1|AT3G59450.1
MPIFQWLKRCLCGDTNITIDQAIALVDEEIANLRKLERGYQTNIRNAENARDQTNVQIERD
SFNDTIRLLQAECNLINLE
IPTLNEEKFAITRLKSAKHFFGAVSSLKEGKALECCKEMIKQVDEDGHGRVDYKEFLQMM
KTGDFSNR
>AtCML2|AT4G12860.1
MDRGELSRVFQMFDKNGDGKIAKNELKDFFKSVGIMVPENEINEMIAKMDVNGDGAMD
IDEFGSLYQEMVEEKEEEEDMR
EAFRVFDQNGDGFITDEELRSVLASMGLKQGRTLEDCKKMISKVDVDGDGMVNFKEFKQ
MMRGGGFAALSSN
>AtCML3|AT3G07490.1
MDQAELARIFQMFDRNGDGKITKQELNDSLENLGIYIPDKDLVQMIEKIDLNGDGYVDIEE
FGGLYQTIMEERDEEEDMR
EAFNVFDQNRDGFITVEELRSVLASLGLKQGRTLEDCKRMISKVDVDGDGMVNFKEFKQ
MMKGGGFAALGSNL
>AtCML4|AT3G59440.1
MVRVFLLYNLFNSFLLCLVPKKLRVFFPPSWYIDDKNPPPPDESETESPVDLKRVFQMFDK
NGDGRITKEELNDSLENLG
IFMPDKDLIQMIQKMDANGDGCVDINEFESLYGSIVEEKEEGDMRDAFNVFDQDGDGFIT
VEELNSVMTSLGLKQGKTLE
CCKEMIMQVDEDGDGRVNYKEFLQMMKSGDFSNRS
>AtCML5|AT2G43290.1
MVRIFLLYNILNSFLLSLVPKKLRTLFPLSWFDKTLHKNSPPSPSTMLPSPSSSSAPTKRIDPS
ELKRVFQMFDKNGDGR
ITKEELNDSLENLGIYIPDKDLTQMIHKIDANGDGCVDIDEFESLYSSIVDEHHNDGETEEE
DMKDAFNVFDQDGDGFIT
VEELKSVMASLGLKQGKTLDGCKKMIMQVDADGDGRVNYKEFLQMMKGGGFSSSN
>AtCML6|AT4G03290.1
MDSTELNRVFQMFDKDGDGKITTKELNESFKNLGIIIPEDELTQIIQKIDVNGDGCVDIEEF
GELYKTIMVEDEDEVGEE
DMKEAFNVFDRNGDGFITVDELKAVLSSLGLKQGKTLEECRKMIMQVDVDGDGRVNYM
EFRQMMKKGRFFSSLS
>AtCML7|AT1G05990.1
MDPTELKRVFQMFDKNGDGTITGKELSETLRSLGIYIPDKELTQMIEKIDVNGDGCVDIDE
FGELYKTIMDEEDEEEEDM
KEAFNVFDQNGDGFITVDELKAVLSSLGLKQGKTLDDCKKMIKKVDVDGDGRVNYKEFR
QMMKGGGFNSL
>AtCML8|AT4G14640.1
MEETALTKDQITEFKEAFCLFDKDGDGCITVEELATVIRSLDQNPTEQELHDIITEIDSDSNG
TIEFAEFLNLMAKKLQE
SDAEEELKEAFKVFDKDQNGYISASELSHVMINLGEKLTDEEVEQMIKEADLDGDGQVN

YDEFVKMMINID
>AtCML9|AT3G51920.1
MADAFTDEQIQEFYEAFCLIDKDSDGFITKEKLTKVMKSMGKNPKAEQLQQMMSDVDIF
GNGGITFDDFLYIMAQNTSQE
SASDELIEVFRVFDRDGDGLISQLELGEGMKDMGMKITAEEAEHMVREADLDGDGFLSFH
EFSKMMIAASY
>AtCML10|AT2G41090.1
MANKFTRQQISEFREQFSVYDKNGDGHITTEEFGAVMRSLGLNLTQAELQEEINDSDLDG
DGTINFTEFLCAMAKDTYSE
KDLKKDFRLFDIDKNGFISAAEMRYVRTILRWKQTDEEIDEIIKAADVDGDGQINYREFAR
LMMAKNQGHDTKYDTTGGT
LERDLAAGVAKNIIAAPMTDFIKNLFEALFS
>AtCML11|AT3G22930.1
MEEIQQQQQQQQQQQQQQQQQQQQQQELTQEQIMEFKEAFCLFDKDGDGCITADELATV
IRSLDQNPTEQELQDMITEID
SDGNGTIEFSEFLNLMANQLQETDADEELKEAFKVFDKDQNGYISASELRHVMINLGEKL
TDEEVDQMIKEADLDGDGQV
NYDEFVRMMMING
>AtCML12|AT2G41100.4
MADKLTDDQITEYRESFRLFDKNGDGSITKKELGTMMRSIGEKPTKADLQDLMNEADLD
GDGTIDFPEFLCVMAKNQGHD
QAPRHTKKTMADKLTDDQITEYRESFRLFDKNGDGSITKKELRTVMFSLGKNRTKADLQ
DMMNEVDLDGDGTIDFPEFLY
LMAKNQGHDQAPRHTKKTMVDYQLTDDQILEFREAFRVFDKNGDGYITVNELRTTMRSL
GETQTKAELQDMINEADADGD
GTISFSEFVCVMTGKMIDTQSKKETYRVVNQGQGQVQRHTRNDRAGGTNWERDIAVGVA
SNIIASPISDFMKDRFKDLFE
ALLS
>AtCML13|AT1G12310.1
MGKDGLSDDQVSSMKEAFMLFDTDGDGKIAPSELGILMRSLGGNPTQAQLKSIIASENLS
SPFDFNRFLDLMAKHLKTEP
FDRQLRDAFKVLDKEGTGFVAVADLRHILTSIGEKLEPNEFDEWIKEVDVGSDGKIRYEDFI
ARMVAK
>AtCML14|AT1G62820.1
MSKDGLSNDQVSSMKEAFMLFDTDGDGKIAPSELGILMRSLGGNPTESQLKSIITTENLSS
PFDFNRFLDLMAKHLKTEP
FDRQLRDAFKVLDKEGTGFVAVADLRHILTSIGEKLQPSEFDEWIKEVDVGSDGKIRYEDFI
ARMVAK
>AtCML15|AT1G18530.1
MEDQIRQLKDIFDRFDMDADGSLTILELAALLRSLGLKPSGDQIHVLLASMDSNGNGFVEF
DELVGTILPDLNEEVLINS
EQLLEIFKSFDRDGNGFISAAELAGAMAKMGQPLTYKELTEMIKEADTNGDGVISFGEFAS
IMAKSAVDYFGLKINS
>AtCML16|AT3G25600.1

MASTKPTDQIKQLKDIFARFDMDKDGSLTQLELAALLRSLGIKPRGDQISLLLNQIDRNGN
GSVEFDELVVAILPDINEE
VLINQEQLMEVFRSFDRDGNGSITAAELAGSMAKMGHPLTYRELTEMMTEADSNGDGVI
SFNEFSHIMAKSAADFLGLTA
S
>AtCML17|AT1G32250.1
MSHKVSKKLDEEQINELREIFRSFDRNKDGSLTQLELGSLLRALGVKPSPDQFETLIDKAD
TKSNGLVEFPEFVALVSPE
LLSPAKRTTPYTEEQLLRLFRIFDTDGNGFITAAELAHSMAKLGHALTVAELTGMIKEADS
DGDGRINFQEFAKAINSAA
FDDIWG
>AtCML18|AT3G03000.1
MSCDGGKPAPAKLGDEQLAELREIFRSFDQNKDGSLTELELGSLLRSLGLKPSQDQLDTLI
QKADRNNNGLVEFSEFVAL
VEPDLVKCPYTDDQLKAIFRMFDRDGNGYITAAELAHSMAKLGHALTAEELTGMIKEADR
DGDGCIDFQEFVQAITSAAF
DNAWG
>AtCML19|AT4G37010.2
MANYMSEAAQLRRGLKPKGKTYGLTNQKRREIREIFDLFDIDGSGSIDASELNVAMRSLG
FEMNNQQINELMAEVDKNQS
GAIDFDEFVHMMTTKFGERDSIDELSKAFKIIDHDNNGKISPRDIKMIAKELGENFTDNDIE
EMIEEADRDKDGEVNLEE
FMKMMKRTSYG
>AtCML20|AT3G50360.1
MSSIYRTVSRKEKPRRHHGLTTQKKQEIKEAFELFDTDGSGTIDAKELNVAMRALGFEMT
EEQINKMIADVDKDGSGAID
FDEFVHMMTAKIGERDTKEELTKAFQIIDLDKNGKISPDDIKRMAKDLGENFTDAEIREMV
EEADRDRDGEVNMDEFMRM
MRRTAYGGN
>AtCML21|AT4G26470.1
MGGAVTKSETLQKEWVPETKLEAKIIEAVQRRASRGTTMKSFNSIVLKFPKIDDGLRNCK
AIFQEFDEDSNGSIDHTELK
NCIRKLEISFDEEEINDLFKACDINEDMGITFTEFIVLLCLVYLLKDDSSTLQKKWTMGMPK
LEPTFETLVDTFVFLDEN
KDGYVSREEMVRAIDESGERSSGRIAMKRFEEMDWDKNGMVNFKEFLFAFTQWVGIDE
NEEEEEEDNNEKA
>AtCML22|AT3G24110.1
MRNMLCCCVNSENNKKYAELDAKLARKMVESRSIYPGHRSLKSMDSIIMKFPKLREGLR
NIRSVFESYDNDTNGTIDIEE
LKKCLEELKLSLSDEEVKGLYSWCDVDGSKGIQFNEFIVLLCLIYLLAKPSSESSTESREMG
PKLVESIFDPIVEVFLFL
DKDGKGKLNKADVIKTLNNEDYPLERSPSHVTNMRFEEMDWGRKGKVGFREFLFAFMS
WVGLDDADDDFMSS
>AtCML23|AT1G66400.1

MSKNVSRNCLGSMEDIKKVFQRFDKNNDGKISIDELKDVIGALSPNASQEETKAMMKEF
DLDGNGFIDLDEFVALFQISD
QSSNNSAIRDLKEAFDLYDLDRNGRISANELHSVMKNLGEKCSIQDCQRMINKVDSDGDG
CVDFEEFKKMMMINGSA
>AtCML24|AT5G37770.1
MSSKNGVVRSCLGSMDDIKKVFQRFDKNGDGKISVDELKEVIRALSPTASPEETVTMMK
QFDLDGNGFIDLDEFVALFQI
GIGGGGNNRNDVSDLKEAFELYDLDGNGRISAKELHSVMKNLGEKCSVQDCKKMISKVD
IDGDGCVNFDEFKKMMSNGGG
A
>AtCML25|AT1G24620.1
MFNKNQGSNGGSSSNVGIGADSPYLQKARSGKTEIRELEAVFKKFDVNGDGKISSKELGA
IMTSLGHEVPEEELEKAITE
IDRKGDGYINFEEFVELNTKGMDQNDVLENLKDAFSVYDIDGNGSISAEELHEVLRSLGD
ECSIAECRKMIGGVDKDGDG
TIDFEEFKIMMTMGSRRDNVMGGGPR
>AtCML26|AT1G73630.1
MANTNLESTNKSTTPSTDMELKKVFDKFDANGDGKISVSELGNVFKSMGTSYTEEELNR
VLDEIDIDCDGFINQEEFATI
CRSSSSAVEIREAFDLYDQNKNGLISSSEIHKVLNRLGMTCSVEDCVRMIGHVDTDGDGN
VNFEEFQKMMSSPELVKGTV
ANS
>AtCML27|AT1G18210.2
MASANPETAKPTPATVDMANPEELKKVFDQFDSNGDGKISVLELGGVFKAMGTSYTETE
LNRVLEEVDTDRDGYINLDEF
STLCRSSSSAAEIRDAFDLYDQDKNGLISASELHQVLNRLGMSCSVEDCTRMIGPVDADG
DGNVNFEEFQKMMTSSSLLN
SNGSAAPPST
>AtCML28|AT3G03430.1
MADATEKAEHDRIFKKFDANGDGKISAAELGDALKNLGSVTHEDIKRMMAEIDTDGDGY
ISYQEFIDFASANRGLMKDVA
KIF
>AtCML29|AT5G17480.1
MADATEKAEHDRIFKKFDANGDGKISAAELEEALKTLGSVTADDVKRMMAEIDTDGDG
NISYQEFTDFAGANRGLMKDVA
KIF
>AtCML30|AT2G15680.1
MSNVSFLELQYKLSKNKMLRKPSRMFSRDRQSSGLSSPGPGGFSQPSVNEMRRVFSRFDL
DKDGKISQTEYKVVLRALGQ
ERAIEDVPKIFKAVDLDGDGFIDFREFIDAYKRSGGIRSSDIRNSFWTFDLNGDGKISAEEV
MSVLWKLGERCSLEDCNR
MVRAVDADGDGLVNMEEFIKMMSSNNV
>AtCML31|AT2G36180.1
MAEIFESVDKNKDGKILWDEFAEAIRVFSPQITSEEIDKMFIVLDVDGDGQIDDVEFASCLM

VNGGGEKDTEEEVVMKEA
FDLYDMDGDGKISASEIHVVLKRLGEKHTMEDCVVMVQTVDKDSDGFVNFEEFKIMMN
SNKESH
>AtCML32|AT5G17470.1
MSVAEIFERVDKNKDGKISWDEFAEAIRAFSPSITSEEIDNMFREIDVDGDNQIDVAEYASC
LMLGGEGNKEDEDIVMKE
AFDLYDIDGDGKISASEIHVVLKRLGEKQTIAECIAMVRAVDADGDGFVSFEEFKTMMSC
NNKKLQ
>AtCML33|AT3G03400.1
MNNMSLSDIFERFDTSKDGKISWEEFRDAIHALSPSIPSEKLVEMFIQLDTNGDGQVDAAK
FASCMDQTAQSSGGDVEKE
LKDAFKLYDINCDGKISANELHVVMTRLGEKCTVESCVGMVQAIDVDGDGYIRFVGV
>AtCML34|AT3G03410.1
MSAKRVFEKFDKNKDGKLSLDEFREVALAFSPYFTQEDIVKFFEEIDVDGNGELNADEFTS
CIEKMLKEVFVFCDVDGDG
KIPASESYVTMTSLGKKFTEETSAEKVRAADVDGDGYLNFDEFMALVIGDI
>AtCML35|AT2G41410.1
MKLAASLNRLSPKRLFRTKSKASVSRSEPSSFSSNASSSSSDGSYGNLKQGPTATPISVLPQ
NSGDFYTELVQAFKLIDR
DDDGVVSRGDLAALISRLSHEPPSQEEVSLMLREVDGGDGGCISLEDLASRVAGTSGEGS
VETEELREVFEIFDVDRNGK
ISAEELHRVFGVIGDERCTLEECMRMIATVDGNGDGFVCFDDFCRMMVPAMNDHHH
>AtCML36|AT3G10190.1
MKLAKLIPKRFFIRSKDRSTVSKSPTAFSFGSASSSSGQDCKNSGGDGGGGSVTPTSILPEV
PSPYSYVEILQAFKLIDR
DNDGAVSRHDLESLLSRLGPDPLTEEEINVMLKEVDCDGDGTIRLEELASRVVSLDPARDS
TELKETFEFFDADRDGLIS
ADELLRVFSTIGDERCTLDDCKRMIADVDEDGDGFVCFTEFSRMMDLQR
>AtCML37|AT5G42380.1
MTLAKNQKSSLSRLYKKVSSKRSESSRNLEDESRTSSNSSGSSSLNVNELRTVFDYMDANS
DGKISGEELQSCVSLLGGA
LSSREVEEVVKTSDVDGDGFIDFEEFLKLMEGEDGSDEERRKELKEAFGMYVMEGEEFIT
AASLRRTLSRLGESCTVDAC
KVMIRGFDQNDDGVLSFDEFVLMMR
>AtCML38|AT1G76650.1
MKNNTQPQSSFKKLCRKLSPKREDSAGEIQQHNSSNGEDKNRELEAVFSYMDANRDGRIS
PEELQKSFMTLGEQLSDEEA
VAAVRLSDTDGDGMLDFEEFSQLIKVDDEEEKKMELKGAFRLYIAEGEDCITPRSLKMML
KKLGESRTTDDCRVMISAFD
LNADGVLSFDEFALMMR
>AtCML39|AT1G76640.1
MKNTQRQLSSSFMKFLEEKNRDLEAVFAYMDANRDGRISAEELKKSFKTLGEQMSDEEA
EAAVKLSDIDGDGMLDINEFA
LLIKGNDEFTEEEKKRKIMEAFRMYIADGEDCITPGSLKMMLMKLGESRTTDDCKVMIQA

FDLNADGVLSFDEFALMMR

>AtCML40|AT3G01830.1

MKSENVNKRDEYQRVFSCFDKSHQGKVSVSTIERCVDAIKSGKRAVVDQEDTTNPNPEES

TDDKSLELEDFVKLVEEGEE

ADKEKDLKEAFKLYEESEGITPKSLKRMLSLLGESKSLKDCEVMISQFDINRDGIINFDEFR

AMMQ

>AtCML41|AT3G50770.1

MATQKEKPSSNSFKWFSTKTLKLNLSFQNRRRSPKSNSSSTLNSPRSNSDDNNNIKSHQAS

KEELRQVFSHFDSDGDGKI

SAFELRHYFGSVGEYISHEAAQEAINEVDTDADGSLGFEDFVGLMTRRDLYGDGEVDGD

GELKTAFEMFEVEKGSGCITP

KGLQKMLVKLGESRTYGECEAMIKFYDIDGNGILDFHEFRQMMTV

>AtCML42|AT4G20780.1

MESNNNEKKKVARQSSSFRLRSPSLNALRLQRIFDLFDKNGDGFITVEELSQALTRLGLNA

DLSDLKSTVESYIQPGNTG

LNFDDFSSLHKTLDDSFFGGACGGGENEDDPSSAAENESDLAEAFKVFDENGDGFISARE

LQTVLKKLGLPEGGEMERVE

KMIVSVDRNQDGRVDFFEFKNMMRTVVIPSS

>AtCML43|AT5G44460.1

MEINNEKKKLSRQSSSFRLRSPSLNALRLHRVFDLFDKNNDGFITVEELSQALSRLGLDAD

FSDLKSTVDSFIKPDKTGL

RFDDFAALHKTLDESFFGGEGSCCDGSPESDLEEAFNVFDEDGDGFISAVELQKVLKKLGL

PEAGEIEQVEKMIVSVDSN

HDGRVDFFEFKNMMQTVVVPS

>AtCML44|AT1G21550.1

MDCSFITTNDLRRMFKTLDKNQDGLVTLDELLWILDKLGWAEHTPDELELIVGKQSLDLD

EFLRFYYDAVLDSKGSKKNI

DVVADNDEAIARAFNVFDVNGDGYISAEELRDVLERLGFEEEAKAWDCGRMIRVHDKNL

DGFVDFEEFKNMILHV

>AtCML45|AT3G29000.1

MESKSSSSSLPLFALFNFFLISFCRWVSSTRIFLSRFVPLLQHHQRVFDKKNNKDQQETLTK

QEDDDDDDDDDDDDDDDD

IDISREEAEMVMRSLGLFYNDDQLQEQYSAKEVSSLFEEKEASLEEVKQAFDVFDENKDG

FIDAIELQRVLTILGFKQGS

YLDNCLVMIRSLDGNKDGKIDFNEFVKFMETSFY

>AtCML46|AT5G39670.1

MTENQLYSFITMKSSLSKCKQSSSLSFPLFGLINFFLIGFFRWVSFAQLFFSRFWPLVQHQQC

VSEKKSKDLEFQTSIKH

EEYRDDDDDGLCREDVGMVMKSLGLSTDQENEGLQKQYSSKEVSNLFEEKEPSLEEVKQ

AFDVFDENRDGFIDPIDLQRV

LTILGLKQGSNLENCRRMIRSFDGSKDGRIDFYGFVKFMENNFC

>AtCML47|AT3G47480.1

MEDSSLLSPISLLTIVIFLFILNLMMIIQDFSSSFPFRFHLFFSNAYILFTSIRNNKQNTELPIIK

KVVVPNRADIKTSV

EEVKAIIDDSEALYECLIEEGEEYLLEKNEMMGKEIVKEAFRLFDENQDGFIDENELKHVL
SLLGYDECTKMECRKMVKV
YDENRDGKIDFYEFVKLIEKSFS
>AtCML48|AT2G27480.1
MSYSNAYAPSAPELPESFVQQQHDGESRYTYAYPSYQPTQQFSSYSGMFSPETHPEIVRSFE
SADRNRSGFLEESELRQA
LSLSGYDGISNRTIRLLLFIYKIPVDSLLRLGPKEYVELWNCLAQWRAIFNRYDRDRSGKM
NSTQLRDAFYNLGCVLPTS
VHQLIVSQFDDGTGKTVDLCFDSFLECGMIVKGLTEKFRENDPGYTGYATLSYDVFMLMV
IPFIATYD
>AtCML49|AT3G10300.3
MSGYPPSSQGYGYGGNPPPPQPPYGSTGNNPPPYGSSGSNPPPPYGSSASSPYAVPYGAQP
APYGAPPSAPYASLPGDHN
KPHKEKPHGASYGSPSPGGYGAHPSSGPSDYGGYGGAPQQSGHGGGYGGAPQQSGHGG
GYGAPPPQASYGSPFASLVPSA
FPPGTDPNIVACFQAADRDNSGFIDDKELQGALSSYNQSFSIRTVHLLMYLFTNSNVRKIGP
KEFTSLFFSLQNWRSIFE
RFDKDRSGRIDTNELRDALMSLGFSVSPVILDLLVSKFDKSGGRNRAIEYDNFIECCLTVK
GLTEKFKEKDTALSGSAIF
NYENFMLTVLPFLVA
>AtCML50|AT5G04170.1
MSGYPPTSQGYGYGYGGGNQPPPPQPPYSSGGNNPPYGSSTTSSPYAVPYGASKPQSSSSS
APTYGSSSYGAPPPSAPYA
PSPGDYNKPPKEKPYGGGYGAPPPSGSSDYGSYGAGPRPSQPSGHGGGYGATPPHGVSDY
GSYGGAPPRPASSGHGGGYG
GYPPQASYGSPFASLIPSGFAPGTDPNIVACFQAADQDGSGFIDDKELQGALSSYQQRFSM
RTVHLLMYLFTNSNAMKIG
PKEFTALFYSLQNWRSIFERSDKDRSGRIDVNELRDALLSLGFSVSPVVLDLLVSKFDKSG
GKNRAIEYDNFIECCLTVK
GLTEKFKEKDTAYSGSATFNYESFMLTVLPFLIA
