# Supplementary material for: Genome-Wide Identification and Expression Analysis of CsCaM/CML Gene Family in Response to Low-Temperature and Salt Stresses in Chrysanthemum seticuspe
Source: Plants (Basel). 2022 Jul 1;11(13):1760. doi: 10.3390/plants11131760 (PMC9268918; doi:10.3390/plants11131760)
Supplement: Supplementary file 1 [file plants-11-01760-s001.zip › Supplementary File S2 Coding sequences of CsCaM_CML.pdf]

>CsCML1
ATGATGAGTGAATCCAATGATTCTTCGAGATGGAGTACATTAACATTGGAAGAAAAAGT
GTGTGGTGCATTAGTCCCATT
ATTGGCTTTTGCTGAGATTATTTGTTTGCTTTTTCTGGCTGCTTTGATTTTGGTGGTGCT
AAGAAGCAAAATAAGCACA
AGCTGCTTCCTTTTGATCATCATCAGCTTACAAGACTTGCTTCTGGATCTAATTTTAATG
TAAATGAAGTTGAGGCGCTT
TTCGAACTGTTTAAGAGCCTGAGTTCGTCTATCATCGACGATGGATTGATACACAAGGA
GGAGCTTCGATTAGCACTATT
CAATACCCCTCAGGGTGAAAATCTGTTTTTGGATCGGGTTTTTGATCTTTTTGACGAGA
AGAGAAATGGTGTAATTGAAT
TCGAGGAATTTGTCCATGCTCTCAGTATCTTCCATCCGTATGCACCTATAGAAGATAAAA
TAAACTTTGCTTTTAGGCTC
TATGATCTAAGACAAACGGGGTATATAGAGCGAGAAGAAGTCAAACAGATGCTAATAG
CAATATTAACAGAATCCGAGAT
GAACTTATCAGATGACCTTCTTGACGTTATTATTGACAAAACATTTGCTGATGCGGACA
CTGATGGAGATGGTAAAATCT
GTAAAGAGGAATGGAAAGAGTTTGCACTCAGATACCCGAGTCTTTTAAAGAACATGAC
TCTTCCTTATCTAGCGGACATC
ACAACAGCATTTCCAAGTTTTGTTTTTCACACGTCGGTGGAGGATGCAACATGA
>CsCML2
ATGGGCTGCTTTCATTCTACTGTAAAAAAACCGGTTCCTGGGCATGAAAATCCGACTTT
ACTTGCTTCTCAAACAGCCTT
TAGTGTAAGTGAAGTTGAAGCTCTATTTGAGCTATTCAAGAGCATAAGTAGTTCTGTAA
TCGATGATGGGTTGATTAATA
AGGAAGAGTTCCAACTAGCATTATTCAAGAACAGAAAGAAAGAGAATCTCTTTGCAA
ACAGGATATTTGATCTTTTCGAC
GTCAAGCAGAAGGGGGTTATTGATTTTGGTGACTTTGTTAGAGCACTTAACGTTTTCCA
TCCTAATGCTCCTCAAGAAGA
CAAGATCAGTTTTTGTTTCAAGCTTTATGATATGGACGGAACAGGATTCATAGAGCGCC
AGGAGGTTAAGCAAATGTTAA
TCGCACTTTTATGTGAGTCGGAGCTAAAGCTAGCTGATGACACCATTGAGACAATACTT
GATAAGACGTTTTCAGAAGCC
GATGTAGACCAGGATGGGAAGATAGACAAATCAGAATGGCACAATTTTGTCACCCACA
ACCCTTCCTTGCTCAAAATAAT
GACTCTTCCATATCTAAGGGATATTACAACCACCTTCCCAAGTTTTGTGTTCAATTCTGA
AGTTGAAGAAATTGCAACAT
AA
>CsCML3
ATGGGCTGCTTTCATTCTACTGTAAAAAAACCGGTTCCTGGGCATGAAAATCCGACTTT
ACTTGCTTCTCAAACAGCCTT
TAGTGTAAGTGAAGTTGAAGCTCTATTTGAGCTATTCAAGAGCATAAGTAGTTCTGTAA
TCGATGATGGGTTGATTAATA
AGGAAGAGTTCCAACTAGCGTTATTCAAGAACAGAAAGAAAGAGAATCTCTTTGCAA

ACAGGATATTTGATCTTTTCGAC
GTCAAGCAGAAGGGGGTTATTGATTTTGGTGACTTTGTTAGAGCACTTAACGTTTTCCA
TCCTAATGCTCCTCAAGAAGA
CAAGATTAGTTTTTGTTTCAAGCTTTATGATATGGACGGAACAGGATTCATAGAGCGCC
AGGAGGTTAAGCAAATGCTAA
TCGCACTTTTATGTGAGTCGGAGCTAAAGCTAGCTGATGACACCATTGAGACAATACTT
GATAAGACGTTTTCAGAAGCC
GATGTAGATCAGGATGGGAAGATAGACAAATCAGAATGGCACAATTTTGTCACCCACA
ACCCTTCCTTGCTCAAGATAAT
GACTCTTCCATATCTAAGGGATATTACAACCACCTTCCCAAGTTTTGTGTTCAATTCTGA
AGTTGAAGAAATTGCAACAT
AA
>CsCML4
ATGATAGAGAAGATAGATGTGAACAATGATGGATGTGTTGACATTGATGAATTTGGTGA
GTTGTATAAAAGTATCATGGA
TGATCGTGAGAATGAGGAGGATATGATGGAGGCTTTTAATGTATTTGATATAAATGGAG
ACGGGTTTATCGCGGTTGAGG
AGCTTAGAGCGGTTTTGGAGTCTTTGGGGCTGAAGCAAGGCCGAAAAGCCGAAGATT
GTAGGAAAATGATCATGAAAGTT
GATGTGGATGGTGATGGTATGGTTAGTTTTGATGAGTTTAAGGAAATGATGAGATCAGG
TGGCTTTGCTGCTATGGCTCA
AAATTGA
>CsCML5
ATGTTTGACAAAAATGGCGATGGAAGGATCACAAAGCAAGAACTAAATGATTCCTTAG
AGAACATGAACATTTACATATG
TGACAATGATCTTGTACACATGATAGAGAAGATAGATGTGAACAATGATGGATGTGTTG
ACATTGATGAATTTGGTGAGT
TGTATAAAAGTATCATGGATGATCGTGAGAATGAGGAGGATATGATGGAGGCTTTTAAT
GTATTTGATATAAATGGAGAC
GGGTTTATCGCGGTTGAGGAGCTTAGAGCGGTTTTGGAGTCTTTGGGGTTGAAGCAAG
GCCGAAAAGCCGAAGATTGTAG
GAAAATGATCATGAAAGTTGATGTGGATGGTGATGGTATGGTTAGTTTTGATGAGTTTA
AGGAAATGATGAGATCAGGTG
GCTTTGCTGCTATGGCTCAAAATTGA
>CsCaM1
ATGGCAGATCAGCTCACCGATGATCAGATCTCTGAATTCAAAGAAGCTTTTAGCCTATT
TGATAAAGATGGCGATGGTTG
TATCACAACCAAGGAACTCGGAACAGTTATGAGGTCTCTAGGACAAAACCCAACCGA
GGCTGAGCTTCAAGATATGATCA
ACGAAGTCGATGCTGATGGCAACGGTACTATTGATTTCCCTGAGTTTCTCAACTTGATG
GCCCGCAAGATGAAGGACACT
GATTCTGAGGAAGAGCTCAAGGAGGCTTTCCGGGTTTTTGACAAGGACCAAAATGGTT
TCATATCTGCGGCCGAGCTTCG
TCACGTCATGACTAATCTTGGTGAGAAGTTGACAGATGAGGAAGTCGATGAGATGATC

CGTGAAGCTGATGTGGACGGTG
ATGGCCAGATCAACTATGAGGAATTCGTCAAGGTCATGATGGCTAAGTGA
>CsCML6
ATGTGTCCCACAGGAACTCTTATACCCCTAGCAACGAAACAAGCCGAACTACGATCAG
CATTCAACGTCATGGACGTCGA
CCATGATGGCAAAATAAGCCACGACGATCTAAAGCGTTTTTACGCAGACTACGCGGTA
AATGACAATGAAATTATCATAG
GAACAATGATGAAGGAGACGGATTTGAATAAAGACGGGTACGTTGAATACGAAGAGTT
TGAAAAAATGTTGTTTCTTAGG
AGAAGTAGTAATGTGATGGAAGAAGTGTTTAATGAAATAGATAAAGTTGGTGATGCTAA
TGATGATGAGAATGATGGTGT
TACTTTTGAAGGGTTTCTTAAGATATTAGCCATTTGA
>CsCML7
ATGAATACGTCCCGTCCCTGTCCTTCTCTCAAGTCATTATTCTACGAAGTTGGAGGAAT
GCTCCGATGTTGCAATTCCAC
AAATGAGTACGAGAGGCTGGACAGTGAGCTCGAGAGGAAAATGATAGAGGTCAAGAA
AAGATATGTACCAGGAAATAGCA
GCATCAGGTCCATCAACAGCATTATTCTGAAGTTTCCTCAGTTCAGACAAGGATTGGAA
GAGATTAGAGGCGTTTTTCGA
CAGTTTGATGTTGATTCAAACGGAACCATCGACCGTGAGGAGTTAAGAAAATGCTTAC
ACAAATTCCAATTCGACTGCAC
AGAGGACGAAATTAATGACCTTTTTGAGTCTTGTAACTTGGGCAGGAATCAAGGCATG
AAATTTAACGAGTTTATCGTTG
TCTTATGCCTTATCTATCTCCTTACATGTACCTCCTCTTCAAACCATACTGCGACAACAAT
GGGATCACCAGAGCTCAAA
TCAACATTTGATACCATAATTGAAGCGTTCTTATTTCTTGATAAAAATGGTGATGGGAAA
CTAGACAAGAAGGATATGAC
TAAAGCTATGAACGATGATTTTCCTAAGGAGAAATCTCCGAATCACATCACCATGTCAC
GATTCAGAGAAATGGACTGGA
ACAAGGATGGGAAAGTCGGCTTCAGGGAGTTCCTATTCTCCCTGATCAATTGGGTTGG
GATCGACTCCAATAATGAAGTT
CATGTCGAAGTAATCCGAGAACAGAAACCGTGA
>CsCaM2
ATGGCCGATCAACTCACCGATGATCAGATCTCTGAGTTCAAGGAAGCTTTCAGTCTATT
CGATAAAGATGGAGATGGTTG
TATCACTACCAAGGAGCTTGGAACAGTTATGAGGTCTCTTGGACAAAACCCCACGGAG
GCTGAACTCCAAGACATGATCA
ATGAGGTCGATGCTGATGGAAACGGTACTATTGATTTCCCTGAATTTCTTAATCTGATGG
CCAGGAAGATGAAGGACACT
GACTCTGAGGAGGAGCTTAAGGAGGCTTTCCGTGTTTTCGACAAGGACCAAAATGGC
TTCATTTCTGCAGCTGAGCTACG
TCATGTTATGACAAATCTTGGTGAGAAGCTGACTGATGAGGAAGTTGATGAGATGATCC
GTGAAGCTGATGTGGATGGTG
ATGGTCAAATCAACTATGAGGAATTCGTCAAGATCATGATGGCCAAGTGA

>CsCML8
ATGAAGTTTTTCTCAACATCTAATGTTTCCATCCCGTTTTCGCTTGAGCGCTCCGAGATT
TTGGTCGTTAAGCATGATTC
CAACTTGTCAAAAGCACACGTTTCCTTCACCAACAAGAACATTGTGGACGATGCATGT
GTCCAAAGAGACGACGTGGAGC
TAATAATGGCGAATTTAGGTGTTTTTTGCCATCCTGAAGGCGAGAAGGTGCCCGAGGT
GATGACTTCTAATGACTTGTTT
AATATTTTTGAAGATGAGCAACCAAGGTTGGATGAAGTGAAACAAGCTTTTGATGTGT
TTGATCAAAACAAAGATGGGTT
TATTGATGCAAGTGAGTTGCAACGAGTTCTTGTTGTTTTGGGCTTGAAGGAAAGATCG
AATATCGAAGATTGTAGGAAGA
TGATCCGAGCGTTTGATGAGAACGCCGATGGTAGGATAGATTTTGATGAGTTTGTTAAG
TTTATGGAAGCTACCTTTTGT
TGA
>CsCML9
ATGTTCCAGCCAGACGTCGATGAAATGAGACGAGTTTTCAACAAATTTGACAAGAACA
AGGACGGTAAGATATCCAAGGA
AGAGTATGGTTCGGCCGTTGGAGTACTTGGTAGCAAAAACACTAAATCAGACGTGATC
AAGACGTTTCAAGCCATAGACA
CTGACGGGGACGGGTTTGTGGACTTTAATGAGTTCATGGAGGCTCAAAAGTCGGAAG
GTGGTGTTAAGACAGCGGATATA
AAGAGTGCGTTTAAGGTTTTTGATTTGGATGGCAATGGGAGAATAACAGCAGAGGAGC
TTGTTCAAGTGCTGAGGCAGTT
AGGAGAGAGGTGCAGCTTGGAGTCTTGCCGAAAAATGATTAAAGGGGTCGATGCAGA
TGGGGATGGAATGATCGACGTTG
ATGAATTCATGGGTCTAATGACCCGTAACATGAAACTGGCATAA
>CsCML10
ATGGCAGATGCTGATCATCAAGCGAACCTTGAACGAATCTTCAAGAAATTTGATACCA
ATGGAGATGGCAAGATCTCATC
ATCAGAGCTTGGAGAAGCTTTGAAGACGCTCGGCTCTGTGTCACCTGAAGAAGTGCA
ACGTATGATGCGTGAAATCGATA
CTGATGGAGATGGATTTATTTCTTATCAAGAATATATAGATTTTTGTAATGCTAACAAGG
GGTTAATGAAGGACGTTTCC
AAGATCGTATAA
>CsCML11
ATGGCTGATGAAGACAAGGCAGAATGTGATCGCATCTTTGGCGCATTTGATAAAAATG
GAGATGGTAAGATCTCTGCAGC
TGAGCTTGGAGAATCTTTGACGAAGCTCGGCTCTGTGTCACCTGAAGAGGTCCAAACT
ATGATGGATGAACTTGATACCG
ATGGAGATGGAAACCCTAGCATTAGCCTCGGCTATAAAAGGAACTCTAATCCTATCATA
AGGGTTTCAATCCATCACTTG
GTTTAG
>CsCML12
ATGTCTATAATGATAGCTGAATTTATCCAATACTTGTTCTCCCATGTGTTTCTCAACATGA

TCATCTACCCACAACTCGG
GTATTTCTTGGATGACTCCAAGATTCATGTTGAGAAGAGGAACAAAGATTCTAGATTGC
CGAAAAGGTTGCCTTCATTTA
AAGACGGAAGTGTACGAGGAGACGAAGTGCAGACGGTTATGGGGAACCTTGGAATCT
TTTGCAATTCTAAAGGGGAAAGC
TTTCCAGAGAGATTAAGTTCTAACGATCTTTTTAACATGTTTGAAGAAGAGCATCCGGA
ATTGGATGAAGTGAAGGGAGC
TTTTGACGTGTTTGATGAGAATAAAGATGGGTTTATTGATGCAAAAGAGTTGCAGAGA
GTTTTATCTGCTTTAGGATTGA
AGGATAGAGCAGCTATGGATGACTGCAAGAAGATGATCAGAGTGTTTGATGAAAACGA
TGATGGTAGAATAGATTTTGAT
GAGTTTGTCAAGTTCATGGAAGGCACATTCTGTTGA
>CsCML13
ATGGATAAAGAACAACAATACAAACGTGTGTTTGGACACTTGGATGCAAATGGTGACG
GAAAGCTATCTCCACCAGAGCT
CCAAATTTGTCTTGGAAAGATTGGAGGAGAGTTGTCATTGGAGGAAGCTGAGATCGCG
GCTGCTTTGATGGATTCGGATG
GGGACGGGTTGTTGAGCATGGAGGACTTGGTGAATGTGGTTGAAAGTGCAAACGAAG
AGGAAAAGATTGATGATTTGAAG
ATGGCTTTTAAGATGTACGAAGAAAAGGAAGGATGTATAACTCCGAAAAGCTTGCGAA
GAATGCTTAGCAAACTGGGAGA
GTCGAGAACCGTCAATGATTGCAAGGTGATGATTAATAAGTTTGATGTTAATGGTGATG
GTGTCCTTAACTTCGATGAAT
TCAGGATAATGATGGCATGA
>CsCML14
ATGGATAAAGAACAACAATACAAACGTGTGTTTGGACAATTGGATGCAAATGGTGACG
GAAAGCTATCTCCACCAGAGCT
TCAAAGTTGTCTTGGAAAAATTGGAGGAGAGCTGTCATTGGAGGAGGCTGAGATCGC
GGCTGCTTTGATGGATTCGGATG
GAGACGGGTTGTTGAGCATGGAGGACTTGGTGAATGTGGTTGAAAGCGCAAACGAAG
AGGAAAGGATTAATGATTTGAAG
ATGGCTTTTAAGATGTACGAGGAAAAGGAAGGATGTATAACTCCGAAAAGCTTGCGAA
GAATGCTTAGCAAATTGGGAGA
GTCGAGAACCGTCAATGATTGCAAGGTGATGATTGCTAGGTTTGATGTTAATGGTGATG
GTGTCCTTAACTTCGACGAGT
TCAGGATAATGATGGCATGA
>CsCML15
ATGGATAAAGAGCAACAATACAAACGTGTGTTTGGACACTTGGATGAAAATGGTGACG
GAAAGCTATCTCCACAAGAGCT
CCAAATTTGTCTTGGAAAGATTGGAGGAGAGCTGTCATTGGAGGAGGCTGAGATCGCT
GCTGCTTTGATGGATTCGGATG
GAGACGGGTTGTTAAGCATGGAGGACTTGGTGAACCTGGTTGAAAGCGCTAACGAGG
AGGAAAGGATTAATGATCTGAAG
ATGGCTTTTAAGATGTACGAAGAAAAGGAAGGATGTATAACTCCGAAAAGCTTGCGAA

GAATGCTTAGCAAATTGGGAGA
GTCGAGAACCGTCAATGATTGCAAGGTGATGATTGCTAGGTTTGATGTTAATGGCGATG
GTGTCCTTAACTTCGAGGAAT
TCAGATTAATGATGGCATGA
>CsCML16
ATGGATAAAGAACAACAATACAAACGCGTGTTCGGACAATTGGATGCAAATGGTGACG
GAAAGCTGTCTCCATCAGAGCT
CCAAATTTGCCTTGGAAAGATTGGAGGAGAGCTGTCCTTGGAGGAGGCCGAGATCGC
GGCTGCTTGGATGGATTCGGATG
GAGACGGGTTGTTAAGCATGGAGGACTTGGTGAATGTGGTAGAAAGTGCTAACGAGG
AGGAAAGGATTAATGATCTGAAG
ATGGCTTTTAAGATGTACGAAGAAAAGGAAGGATGTATAACTCCGAAAAGCTTGCGAA
GAATGCTTAGTAAATTGGGAGA
GTCGAGAACGGTCAATGATTGTAAGGTGATGATTGCGAGGTTTGATGTTAATGGTGATG
GTGTCCTTAACTTCGACGAAT
TCAAGATAATGATGGCGTGA
>CsCML17
ATGGACAAAGAACAGCAATACAAACGCGTATTTGGGCACCTGGACACAAATGGAGAC
GGGAAGCTATCTCCTCCTGAGCT
CCAAATTTGCTTTGGGAAGATTGGTGGAGAATTGTCATTGGAGGAGGCTGAGATAGCA
GCTGCTTTGATGGATTCGGATG
GAGACGGATTGCTAAGCATGGAGGACTTGGTACAAGCGGTTGAGGGCGCTAACGAAG
AGGAAAAAATTAATGATTTGAAG
ATGGCTTTTAAGATGTATGAAGAAAAGGAAGGGAGTGGATGTATAACTCCGAAAAGCT
TGCGAAGAATGCTTAGCAAGTT
GGGAGAGTCAAGAACCGTCAATGATTGTAAGGTGATGATTGCTAAGTTTGATGTTAATG
GTGATGGTGTCCTCAACTTCC
ACGAATTCAGGGAAATGATGGTGTAA
>CsCML18
ATGGACAAAGAACAACAATACAAAAGTGTGTTCAGACACTTGGACAAAAATGGAGAT
GGGAAGCTATCGCCACCGGAGCT
CCAAACCTGCATTGGAAAGGTTGGTGGAGAGTTGTCACTGGAGGAAGCCGAGATGGC
AGCTGCTTTGATAGATTCAGATG
GAGACGGGTTGTTGAGCATGGAGGACTTGGTGAAAGTGGTCGAAGGTGCAAACGAAG
AGGAAAAAATTAATGATTTGAAG
ATGGCTTTTAAGATGTATGAAGAAACGGAAGGGAGTGGATGCATAACCGCGGAAAGCT
TGAGAAGAACGCTTAGCAAACT
GGGAGAGTCGAAAACAGTTGATGATTGTAAAATAATGATTGCTAAGTTTGATGTTAACG
GTGATGGAGTCCTCAATTTTG
ACGAATTCAGGGAAATGATGGCGTGA
>CsCML19
ATGGCAACTGAAACAACAAACCCTACCACAACTACCCAACAAACATCATCATCAGTGA
ACCTAACCAACATCGAAGAAGT
AAAGAAAGTATTCAACCGTTTCGACACCAACCACGACGGCAAGATCTCATCTTCGGAG

CTCATCTCCATCATGAAATCCC
TCGGATCCAACATCTCAGAGGACGAAGTCAGACAAATGATGACCAAGATTGACACTGA
TAACGACGGGTGCATAACCCTG
GAAGAATTTGCAGGGTTTTGTAAAGATGATACTGCTGATGATGGGGGAATGAAGGAGT
TGCATGAAGCTTTTGAGCTTTA
TGATTTGAATAACAATGGTTTGATTAGCTCTAGTGAGTTGCATCAGATTTTGACGAGGT
TGGGTGAGAGTGTGAGCGTTG
AGGATTGTGTTGGGATGATTAAGTCTGTTGATGCTGATGGTGATGGCTTTGTAAACTTT
GAGGAGTTTAAGAAGATGATG
AGTAATGGCAAGACTGAGCCGTAG
>CsCML20
ATGGGTTTAAAAAATTTGTTCAAGGGTAAAAAAAAATCAACCCAGAACAACAACAAC
GTGGTCGTGGGTGTTGCTGTTAG
AAATCCAAACAATACATCATCGCCAGTTCTTTCGCGATCAAGCTCGTGCAACTCACGTG
CTCGGATCGAGGAAGAACTAA
CGCAAGTTTTCAGAAAATTCGACGTAAACGGAGACGGAAAGATCTCCGCCTCCGAAC
TGGGATCAATAATGGGGTCCCTG
GGCCAAAAACCCACAGAAACCGAGCTCGAAAACATGATCAAGGAAGTTGATGCTGAT
GGGGATGGGTTCATAGATTTGCA
TGAATTCATTGCTCTTAATACTAAAGATATCGATTCAAATGAGCTTTTGGAGAATCTAAA
AGAAGCCTTTTGTGTATTCG
ATATTGATAAGAATGGTTCTATAAGTGCTGAAGAGCTGCAGAAAGTTTTGGGCAGGTTG
GAAGAAAGCTGCACCATTGAA
GAGTGTAGGAAAATGATTAGTGGTGTTGATGTTGATGGAGATGGTATGATTAGTTTTGA
TGAGTTTAAGGTTATGATGAT
GAGTGGTAATGGGTTTGTGTCTAAGAAGAATAATAACCGAGAGATGAAGGAAGAATAA
>CsCML21
ATGAGCAAAGTGTCAGTAATACTCTACATTACAGTAGCCATACTAATCCTCATTCTAATC
TCCAACAAAAACACCAACAA
CACCTCCTCCAACCGCCACAACCGCCGCCACCGCCGCCTCAAGCTCCGCTCAAACTTC
ACCACCACCCCCATACCCACCA
CCCCCATACCCACCACCCCATCAGATCACCATATCTCATTTGACCCCCTTATCGCAGACA
TTGAACGTAAACGTGAAGAC
AAACAATGGGAACAAACCCATTACTTTAATAAGGACCATGGTGATGTTCATGATCATGG
TGGTGATGACGTGGCACCTGG
AATGGAGGGACAGCCAGAATGGGAGGATTTTATAGATGCTGAGGATTATTTGAATGATG
AGCATAAGTTTAATATTACGC
ATAGGTTGGTGTTGTTGTTTCCCAGGATTGATGTTGATCCTGCTGATGGGTTTGTTTCGG
AGCATGAGTTGACGCAGTGG
AACTTGGAGCAGAGTCAGAGGGAGGTGTTGCATAGGAGCCAGAGGGAGATGGAGCTG
CATGATAAGAATCGGGATGGGTT
GGTTAGTTTCCACGAGTATTCGCCGCCTAGTTGGGTGAGGGATACAGATAATAACTCAT
TTGGGTATGATATGGGTTGGT
GGAAAGAGGAGCATTTCAATGCATCTGATATTGACGGTGATGGTTTTTGAATTTGACC

GAGTTCAATGACTTTCAGCAT
CCCGCAGACACTAAAAACCCAAGACTTCTTCAGTGGCTGTGCAAGGAGGAAGTAAGG
GAACGAGATACTGACAAAGATGG
GAAGGTGAATTTTAAAGAGTTTTTCCATGGACTATTTGACCTCGTAAGAAACTATGACG
AAGAAAGTCATCATAATTCTT
CACATGAATCTAATAACGATTCATTGGAATCACCGGCAAAGAGATTTTTCTCCGAGCTT
GACAAGGATGCTGACGGATTC
TTGTCTGACGTGGAGCTACTACCTATCATCGGAAAGCTTCATCCATCAGAGCGTTATTAT
GCTAAGCAACAGGCAGATTA
TATTATATCACAGGCTGATACGGATAAAGATGGACGGCTATCCTTGACTGAAATGATCGA
TAGTCCATATGTATTTTATA
GTGCTATCTTTAACGAGGATGATGAAGAAGATTATGAATACCACGATGAGTTCCGTTAA
>CsCML22
ATGAAAACCATGACATTAAAAGAATGTTGTTGTTTCTTAAAGAAACTACGGAATCCTAG
TCGAATTATCCAACATGCTCT
TCTTGCTTCCGAGACATCATTTACCATCAATGAAATAGAGGCATTATACGATCTTTTTGA
GAGATTAAGCTATGCTATCA
TTGAAGATGGCCGTATTCACAAGGAAGAGTTCCGGCTTGCTCTTTTCAGCAATAGCAC
CATGCAGAATCTCTTTGCAGAT
AGACTATTTGAGTCATTTGACATCAAGAAAAATGGAGTTATTGAATTCGATGAATTCGT
TCGTTCGCTAAGCATCTTCCA
TCCTAATGCACCAGAATCAGATAAAATTGAATTCATGTTTAGATTATATGACTTGAGGCA
CACTGGATTCATAGAACGTG
AAGAGTTGAAGGAGATGGTAGTGGCTCTCCTGAGTGAAATGGATGTGAGTGTATCAGA
TGAAGATATCGAAGCTATCCTA
GACAAGACACTTTTGGATGCAGATCTAAACGGGGATGGAAAGATTGATTTAGAAGAGT
GGAAGATATTCATCTCAAAGAA
TCCATCCATTCTCAAGAACATGACTCTTCCCCTTCTTAGGGAGATCACTCAAGCCTTTC
CAAACTTCGTCTTGAATACTC
AAGTCCAAAACCTAGAAGTAGAAGTATAG
>CsCML23
ATGAGCAGTGACAAAGAACCAACCGTGAAACTCGACGACGAACAGCTAAGCGAGCTA
CGTGAAATATTTAGATCATTCGA
TAGAAACAACGACGGAAGCCTAACACAGCTTGAACTCGGTTCATTATTACGCTCACTA
GGCCTCACACCTAGCTCAGATC
AACTCGATACATTAATACAAAAAGCCGATACAAATAGCAATGGTTTGGTTGAATTTTCT
GAGTTTGTGGCACTCGTGGCA
CCCGAGCTTCTTCCTGCTAAATCGCCTTATACAGATGATCAGCTGAAGCAGCTGTTTAA
GATGTTTGATAGGGACGGAAA
TGGCTATATAACGGCTGCTGAGAGTTTTCTCGCGCCATTACTTCAGCTGCTTTTGATAAT
TCTTTTTCGTGATTTTGGTT
ATTCGTTTGCATACCTTTTTGTTTTCAGATGA
>CsCML24
ATGGGCTGTTGTTATTCTGCCATGGGTGTTCCGGTTGTTGCCCGTGAAGATTCTAGGAC

CCTTGCTTCTCAAACAACTTT
TAGTGTTAGTGAAGTTGAAGCACTATTTGAGTTATTCAAGAGCATAAGCAGTTCTGTAA
TCGACGATGGGCTAATTAGCA
AGGAAGAATTCTTACTAGCCTTGTTCAAGAACAAAAGGAAAAAGAATCTGTTTGCAAA
TAGGATTTTTTCTCTTTTTGAC
GTTAAGCGTAAGGGGGTCATTGATTTTGGTGACTTTGTTAGATCACTAGGCGTGTTTCA
CCCTAGAGCTCCGTTAGAAGA
TAAAATTAATTTTTCGTTTAAGCTTTATGATTTGGAAGGAACAGGCTTCATTGAGCGCC
GAGAGGTGAAACAAATGTTAA
TTGCACTTTTACATGAGTCAGAGCTAAAACTGGCTGACGAGGCTATTGAAATGATACTG
GACAATACATTTTCAGAGGCG
GATGGAGACCGGGATGGAAAGATAGACAGATCAGAATGGCACGCTTTTGTTACCAAGA
ATCCTTCCTTGTTGAAGACAAT
GACACTTCCTGTTTTAAGGGATATTACATCAGCATTTCCAAGCTTTGTTTTCAATACTCA
AGTTGATGAGATTTCGTCTC
CTCAAGTTGAAGAGGTTGAGATTTCATCTTCTCAAGTTGAAGAGATTGAGATTTCTTCT
TCTCAAGTTGAAGAGATTCCA
AAATGA
>CsCML25
ATGGACAAAGAACAACAATACAAACGTGTATTCAGACATTTGGACGTGAATGGTGACG
GGAAGATATCGCCACCCGAGCT
CCAAATTTGTATTGGTAAGATAGGTGAGGACTTGTCATTGGAGGAAGCAGAAATCGCG
GCAGAGTTAATGGACTCGGATG
GTGACGGGTTGTTGAGCTTCGATGATTTGGTTAAAGTGGTCGAAAGTGCTAATGAAGA
AGAGAAAGTAAAAGACTTGAAG
TTGGCATTTAAGATGTATGAAGAAATGGAAGGCTGTGGCTGTATAACTCCTAAGAGCTT
GAAGAGAATGCTTAGCAAATT
AGGAGAGTCGAGAACTGTTGACGAGTGTGAGTTGATGATCAAGAAGTTCGACCTTGAT
GGCAATGGTGTACTTGATTTTC
AGGAGTTTCAAGACATGATGTCATGA
>CsCML26
ATGGACAAAGAACAACAATACGAACGTGTATTCAAACATTTGGACGTGAATGGTGACG
GGAAGATATCGCCACCCGAGCT
CCAAATTTGCATCGGTAAGATAGGTGAGGACTTGTCATTGGAGGAAGCAGAGATCGCG
GCAGAGTTAATGGACTCGGATG
GTGACGGGTTGTTGAGCTTCGATGATTTGGTTAAAGTGGTCGAAAGTGCTAATGAAGA
AGAGAAAGTAAAAGACTTGAAG
TTGGCATTTAAGATGTATGAAGAAATGGAGGGTTGTGGCTGTATAACTCCTAAGAGCTT
GAAGAGAATGCTTAGCAAATT
AGGAGAGTCGAGAACCGTTGACGACTGTGAGTTGATGATCAAGAAGTTCGACCTTGAT
GGCAATGGTGTACTTGATTTTC
AGGAGTTTCAAGATATGATGTCGTGA
>CsCML27
ATGGAGTCTAATAAAGTTCCAAAATCTTTTGGATGTTTTCCTCAAAAGGGTCTCGTTTT

AAGGTTAAGTAGTTTTCGTTC
CAAGAGTAACAATACTTCGAACTCCACTTCTCCTACGTTAATGTCTCCTAGATCGCCGA
AACCAAACACTAACAACAGAG
AACAAGAGTTTAGATCAGTCTTTGCGCGTTTTGATGCTGATAATGATGGAAAGATTTCA
GCATTGGAGCTTCGGTCATAC
TTTGGCTCCATTGGTGAGTACATGTCGCACCAAGAGGCTCAGGGCGTGATCGATGATCT
GGACACAGATGGCGATGGCTT
TATTGATTTTCAAGATTTTATGAGGCTAATGAAGGTGAGCAATGAAAAGGATGATGTGA
AGGCGGCGTTTGAGATGTTTG
AGTACGAGAAAGGGTGTGGACAGATTAGTCCAAAGAGCTTGCAAAAGACATTAAGTC
GACTTGGCGATTCAAAAACTTAT
GATGAGTGTTTGCAAATGATTAAGATGTTTGATACTCATGGCAAAGGAGCTGTTGATTT
CAATGAGTTTCAACAGATGAT
GACGGCTTAA
>CsCML28
ATGGCACAAACCGACGTCGAAAAAGTGTTCAAAAAATTCGACGTGAACGGCGACGGC
AAGATCTCCATAACCGAACTCGG
CTCAATCCTTGCCGCACTCAGCGGCGCCGTCACATCTGAAACCGAACTGAAATCGGTC
ATGAAAGAAATCGACACTGACG
GTGATGGATTCATTGATTTTGACGAGTTTGTCGCGTTTCATAACGGTAACGGTGAAGAA
GAAGAGAGTAAGGAGTTGCGT
GAAGCGTTTGATTTGTATGATGAGGATAAGAATGGGAAGATCTCAGCGAATGAGTTGC
ATTCGGTTATGAAGAGGTTAGG
TGAGAAGTGTTCGTTGAAGGATTGTAAGAAGATGATTCAGAGCGTTGATGTTGATGGT
GATGGTTGTGTTAATTTTGAAG
AGTTTAAGAAGATGATGAATAAGTGA
>CsCML29
ATGCCTGCGGGCTACAGCTGGGCCAAACCGCCTGCTATGACTCTTGTCCATCAAAATCT
TTCTATGGGTTGCTTTCCAAG
ACTTGTAACTCCTGCATTTGAAAGTTACTCTTGCCGTTGGACTATTCGGACATCTGCATT
ATTCGTGCCTCAGCAACGTC
TTTTCCATGACTCAGCTAACAATATTCTGATATTGCTTGCTATATTGTCCCTTCTGAGTAG
ATGGGGATATCTTGAAAAG
CAAAACAAAGCACAGCCAAACATTAAGTCATGTTTTATGGAGTGGCGTCTTAAACAGG
TAGAAGCACCCTTGACAGATGA
TCAAATAAAGGGTCTAGTCAGCAAGTTTGACACTAATGGAGATGGAAAGATCAGTCGG
AGGGAACTGAGGGTAGGCTTGA
AGAGTCTTGGCCTGCATTTCGCAGGCTTAAGAGCTATGGGTGCAGTACGTCATGCTGAT
GCTAATGGAGATGGCGTTATT
AGCGACGAAGAGATAAACGAGCTTGCTAAATATATTTCCAAGTGGGGAATTTCTGTAAC
TTAA
>CsCML30
ATGCGGCAGTCCTGGTGTCTTAGACAGGTAGAAGCGGCGCCCCTGACAGAAGACCAA
ATAAAAGGTCTGCTCAGAAAATT

CGACGCAAATGGTGACGGCAAGATCAGCAGAAGGGAACTAAGGGCTGGCTTGAAAA
GCCTTGGTCTACGTTTCGCGTGCT
TTAGAGCTAGACGTGCACTGCGTTATGCTGATGCTAACGGAGATGGTGTTATCAGCGAT
GAAGAAATAAACGAGCTCGCC
AGATATGTTTCCAAGTGGGACATTTCTTTAACTTAA
>CsCML31
ATGACGCGAAAGTCGTGGTGTCTTAAACAGGTAGAAATGCCCATGACGGATGACCAAA
TAAAGGGTTTAGTCAACAAGTT
CGACACTAATGGAGACGGAAAGATCACTCGGAGGGAACTGAGGGTAGGTTTGAGGAA
ACTTGGCCTACGTTTCGCAACCT
TTAGAGCTATGGGTGCAGTACGTTATGCTGATGCTAACGGAGATGGTGTTATTAGCGAT
GAAGAGATAAACGAGCTTGCT
AAATATATTTCCAAGTGGGGAATTTCTGTAAATTAA
>CsCML32
ATGTCGCTTTCGGAACAATTTCATATCATAGGTGACTTGCATGTATTTTCCACACAATTT
TCCAATTTATGTCTACATAA
AGCAAAGTTTATCATTGACACACCCCCAACAAAGACTTGCACGGCAGCCACAGCACA
CGAAAAGGCTCATGCAACGAACC
TTGGAGGTGTTGAGAATGGCCCGGGTAAGAAGAAGGCGTTGGGAGTGAATCCAAAGA
CTATGATGGAAAGATTGGGAACG
TTTTGGGATCCTGATGACCATAGGGAGGCTCTTGATATGGACGAAATGGTGAATTTGTT
TGTTGAGGACGAGCCAAGCTT
AGATGAAGTGAAACAAGCGTTTGGCGTGTTTGATAAGAACAATGATGGCTACGTAGAT
GCAAAAGAGCTACAAAATGTGC
TTTCTAATATGGGCTTTTTACATATATGTGAAAGTGATTGTAGAAGGATGATTGTTAGCTA
TGATGCTGACAAAGACGGT
AAGCTTAGTTTTCGCGAATTTTTGAAGGTCGTGGAAGATGGCTTTCGGTAA
>CsCML33
ATGTCAACATTGCCCGAGGACCAACTCAATCAACTCAAAGAAATATTCACACGTTTCG
ACCTAGACAAAGACGGGTCCCT
AACACATCTAGAGGTCGCAGCCCTCCTTCGGTCTCTTGGCCTCAAACCATCTGGAGAC
CAAATTCACAAACTATTTAAAA
ACATGGATTCTGATGGCAGTGGGACCGTGGAATTCGACGAATTAGTGAATTCCATGTCG
TCCCATATGATGACTGAAGAG
ATATTGGTTAATCAACAACAACTTATGGAAATATTTCGATCTTTTGATAGAGATGGAAGC
GGGTTTATAACGCCTGCTGA
GCTAGCGAAATCTATGACGAAAATGGGACAACCTTTAACGTATCGCGAGTTGTCTGAA
ATGGTTCGAGACGCGGATACGG
ATGGAGATGGTGTTATTAGTTTTAAGGAGTTTCAAGGGATTATGGCTAGGTCGGCAGCT
GATTCACTTGGGTTCTCATTG
TAA
>CsCML34
ATGGTTGTAGTCCATCTAGATCCAATCCTGATCCATTATAATCCCGGCAACGATGGGCGC
GGCGGCGAGCTCGTCATCAT

CGGAAATATAGAGATTGAGCTCACTGACATTGGGGGAAAAGTTATGAAAGCAGCTAAA
CCGGCACTGACTTTTGATGACA
TTAGACGGCTGGCTCTTAATTCGCCATTTACGGTTAATGAAGTGGAAGCATTGCGTGAA
TTGTTCAATAAGTTGAGCAGT
TCGATTATAGACGATGGACTCATACACAAGGAAGAGCTCCAATTGGCGTTGCTCAATTC
AGCCGGTGGAGAAAATCTTTT
CTTGAACAGGGTTTTTGATCTATTTGATGAGAAGCAAAATGGCGTTATTGAATTCGAGG
AGTTTATCCATGTATTGAGCA
TCTTCCATCCTTATGCCCCAATAGAAGAAAAAATTGATTTTGCCTTCAGGTTATATGATT
TGAGAAAAACCGGGTACATA
GAACGGGAAGAAGTTAAAGAAATGATTGTTGCCACTTTAAAGGAAACCGGGATGCGT
CTTTCAGAAGAAATACTTGAAGA
GATAATTGATAATACATTTGCAGATGCAGATGCGGACATGGATGGAAGAATCAACAAAG
AAGAGTGGAGGGACTTTGTTA
TCCAACGACCCCAACTCTTGAAGAACATGACCCTTCCATCTCTTAGGGATGTGACGAC
AGCCTTCCCAAGTTTTATATTC
AACACAGGAGTAGATGATTGA
>CsCML35
ATGGATCCAGCTGAGCTACGCCGTGTTTTCCAAATGTTTGATCGCAATGGCGATGGCAA
GATCACAAAGCAAGAGCTTGC
AAAATCTCTAGAAAACCTTGGAATATACATCCCGGATGATGACCTAGCCCAAATGATCG
AGAAGATTGATGTCAACAAGG
ACGGCTTTGTGGACATGGAGGAATTTGGTGAGCTTTATCAAACAATATTGGGTGAGAG
GGACGAGGAAGAGGATATGAGA
GAGGCGTTCAATGTGTTTGATCAAAATAGGGACGGTTTTATCACCGTGGAAGAGCTTA
GGTCTGTTTTGAGCTCGCTTGG
GTTAAGGCAAGGCCGGTCTATCGAGGAATGTAGGCTCATGATAAAGAAGGTGGACGAA
GATGGTGATGGAATGGTTAACT
ACAAAGAGTTCAAGCAAATGATGAAAGCAGGTGGTTTTGCAGGCTTGGAAACTTAA
>CsCML36
ATGTCAGGCTACCCTCAAAACCCCTCAGGCTACGGCACTCCACCCGCCCAACCTTACG
GTGCCCCACCCCAACAACCTTA
CGGTCAACCCCCTCAACAATCCTACGGTCAACCCCCACCAGCCCAACCCTACGGCGCA
CCACCTGCCCAACCTTACGGTG
CACCTGCACAGCCCTACGGTGCACCCGCACAGCCTTACGGTGCTCCCTCAGCCCCATA
CGGCCAAAACCCCAACAAACCT
CCTAAAGAAAACAAACCACAGGGAAGTGGTGGGTACGGTGCTGCTCCTCCACCTGGT
GGCGCGTACGGCCAGGGAGGGGC
GCCGTACGGGAGTCCGTTCGCGGCGTTGTTGCCGTCGACGTTCCCGCCAGGGACTGAT
CCGAACGTGGTGGCGTGTTTTC
AGGTGGCGGATCAGGATGGGAGTGGTGTGATTGATGATAAGGAGCTGCAGAGGGCTTT
GAGTAGCTATAATCAGAGCTTT
AGTCTCCGTACTGTTCATTTGCTCATGTATCTTTTCACCAATACTAACACCAGGAAGATC
GGACCCAAGGAGTTCATTCA

AGTTTTCTACAGCTTGCAGAACTGGAGGGCAAACTTTGAGAAATTTGACAGAGATCGC
AGTGGCAAGATTGATATCAATG
AATTGCGAGAGGCCCTCATGAGCCTTGGCTTTGCAGTTTCACCTGTGGTTTTGGATTTG
CTAGTGTCCAAGTTTGACAAG
AGTGGTGGAAAAAACAAGGCTATTGAATATGACAACTTCATCGAGTGCTGCTTGACTG
TTAAGGGCCTAACAGAAAAGTT
CAAGGAGAAGGACACATCGTACTCAGGGAATGCTACCTTTACGTATGAGGCGTTCATG
TTGACTGTTTTGCCCTTCCTCA
TTGCTTAG
>CsCML37
ATGAAGCTCTCCGGTAGAATCAACCCAAAAAACATCTTCCGATCCAAAAGCCACAAAA
AAGACTCCGTCTCCAGATCCGA
ATCCTCTTCATTCAGCTCATCCATTACGACGTCGTCTGGTTCACCAGAACGATCAAAAG
GGGCCACAACACCAACAACTG
TCTTACCAACACAACCCTTAACAAGATCTGACTTAGAAGCTTTGCTGCGCCGTATAACC
AATGATGAAGCTGAAGTTAAG
TTAATGCTTGATGAAGTCGAAGGTGACGGTGAAGTTGAGGGAACGATGACGGAGTTT
GGTGAAGAAGAGATGAGAGGCGC
CTTTGAGTTTTTTGACAGTGATGGCGACGGAATGATAACGGCGGATGAGCTGTTTCAG
GTTTTTAAAGTGATTAATGGTG
ATGATGGGTGTACGTTAGAAGAGTCCACCATACATATCCAGTCATTGCTTTCATCTTCTA
ATCTTCTACAAATATCTATA
TTCAGATGTATACGGGTCATTCGACATGTGAAAGGTACTTTGAAAAGAGAAAGAGAGT
GA
>CsCML38
ATGAAGGAAGCATTCGAGCTATTTGATACTGATGGCAATGGTACCATTGATGCTAAGGA
GTTGAGCAATGCAATGAGGGC
CCTTGGTTTCGAAATGACAAAGGAGCAACTTGATCAGATGATAGCGGATGTAGACAGA
GATGGCAGTGGTGCAATTGATT
TTGATGAATTTGTGTACATGATGAGTGATAAAATTGGGGAAAGGAGCAACAAACAGGA
GCTTACAAAAGCATTCAACATT
ATTGATCATGATAAAAATGGAAAGATATCAATTTTGGACATCAAGAATATTGCTAAGGA
GTTGAGTGTACGCTTTACCGA
TGCAGAAATTCACGCAATGGTGGAGGAAGCAGATCGTGATGATGATGGAGAAGTTAGC
AAAGAGGAGTTCATGAGAATGA
TGCAGACAACTTCTTATGGATATTAG
>CsCML39
ATGAGGGCCTTGGGTTTTGAAATGACGGAAGAGCAAATCAATCAAATGATAGCAGACG
TAGACAAAGATGGGAGCGGTGC
AATTGATTTTGATGAATTTGCATACATGATGACTGCCAAAATTGGTGAGAGGGACAGCA
AACAAGAGCTTACAAAAGCAT
TCGAAATCATTGACCAAGATAAAAATGGGAAGATATCCGTTGCTGACATTAAGAAAATA
GCTAAGGAGTTGGGTGAACAT
TTTACCGATGATGAGATTCACGAGATGGTCGAGGAAGCAGATCGTGACCATGATGGTG

AAGTCAGTGCAGAAGAGTTTAT
GAGAATGATGAAGAGAACTTCATATGGATATTAA
>CsCML40
ATGATCTATAATTATTATAACAAATCGATTTTAGAAGATCTATCCGGTTCTTTCTTGCTTTT
CTCAACAAGGAGTGATAT
ATTCGAACCTCCAGTTAATGATGATCGTCCTTCACAAACTCGTTCAACGCGCACTTTCT
ATAGAGGAAAAGGAGAAGCTG
TTATTTTTATAAAGAATTCGTTGCAAAAAATGGGAGTTCGCAGAGAATCATCAAGAAGC
GACCAGCCCAGAGGACGTCAT
CATGGGTTAAATCCGCAGAAAAAGCAAGAGATCAATGAAGCATTTGACCTATTTGATAC
TGATGGATCTGGTACCATTGA
TGCCAAGGAGTTAAATGTTGCAATGAGGGCCTTGGGATTTGAAATGACAGAGGAGCA
AATTAATCAAATGATAGCGGATG
TAGACAAGGATGGCAGTGGTGCAATTGATCGTGATGAATTTGAGTACATGATGACTGCC
AAAATTGGTGAAAGGGACAGC
AAACAAGAGCTTAAAAAAGCATTTGAAATCATTGACCAAGATAAAAATGGCAAGATAT
CTTTTGCAGACATTAAGAAAAT
TGCTAAGGAGTTGGGTGAACATTTTACTGATGCAGAGATCCACGAGATGGTCGAGGAA
GCCGATTGTGATGATCGTGATG
GTGAAGTCAGTTCAGAGGAGTTCATGAAAATGATGAAGAGAACTTCTTATGGATATTA
G
>CsCML41
ATGTCGAAAATGAGTTTTCTTGACATCCAATACAACATCTCTAAGCGTAAGTTCCTTAG
GAAACCATCTAGGATGTTCTC
TAGTAGCGAAAGACAACCTTCGGGCCTGCCTATGTTCCAACCAAATGTGAACGAGATG
AGGCGTGTTTTTGACAAGTTTG
ATCGTGATAAAGATGGCAAGATATCAAGAGGCGAATACAAGGCTATTCTTAGAGCACTG
AAACAAGGAGGTACAGAAAGA
GACATCCAAAAGATATTTGAGGTGGCGGATTTGGATGGAGATGGATTTATTGATTTCAA
GGAGTTTATGGAGGTGCAAAA
GAAAGGTGGTGCAGTTAAAGCGGTAGATGTGCAAAGTGCGTTTAAGACGTTTGATCTC
GATGGAGATGGGAAAATAAGCG
TCGAGGAAGTGTATGAGTTGATGAAGAGGCTTGGGGAGAGGTGTAGCTTGCAAGATTG
TCGAAAAATGGTGAGAGGTGTG
GATTCGAACCAAGATGGTGTGATTGATATTGATGAGTTTATGACGATGATGACACAAAA
CATGAAGATTTAA
>CsCML42
ATGAGCAATTCAAGTGAGAGAAAAGCTGAGCTAAAAAGTGTTTTTGCCACCTTTGACA
AGAACAAAGATGGGTTCATCAC
AAAACAAGAACTTAGTGATTCACTCAAGAATATAGGCATATCAACTAGTGAGAAAGAT
GTGGTAGAAATGGTTCAAAGGG
TTGATGTTAATGGTGATGGATTGATAGATTTTGATGAGTTTTGTGAGCTTTTTGAGTCCA
TGATGAGTAAAGAAGACATG
CAAGGGAGTAAAGTTGGTGATGATGATGATCATGAAGATGGTGATTTAAGAGATGCTTT

TAATGTATTTGATGGTGATAA
AAATGGGCTTATAAGTGTTGAAGAATTAGGGTTGGTTTTGGACTCATTGGGTTTTAAGG
AAGGGAAAAAGTTGGAAGATT
GTAAGAAGATGATTAGCAAAGTTGATATTGATGGTGATGGTATGATCAATTTTAATGAGT
TCAAGAGCATGATGAAAAGT
GGTGTTAGTCTTATTTCAGTTTCTTGA
>CsCML43
ATGTCTTCTCCTCCTAGCTACAACGATCTGTATCGCCTTTTCAAGAAGCTAGACCAAAA
TGGAGACGGTCTTGTCAGCCC
GCATGAGCTCCAATGGCTTCTTGATACCATGAAAGTGTCTTCAAGTGTCGATGACCTAA
GATACTTAACGGGTAAAACCA
ATATTAATTTTACTGAGTTCTTAGAGTTTTACGGTACCATTACAAAGGAAGAAAAGGTA
ACCTGTGATGATGAATCAGAA
AGTGACCTCTTCAAGGCGTTTGAGATGTTTGATAAGAACCGTGATGGATTCATTTGTAA
TGAGGAGCTAATGGAGGCGTT
AACAAGGTTGGGATTATGGGACGATAAGAGTAACATGGATGTTAAGAGTATGATCAAA
GCCTATGATGCCAATTGTGACG
GTTTTATTGATTTCCATGAGTTCAAGAAAATGATGGCTTAG
>CsCML44
ATGTCTCAGTGCTTAGAAGGGATCAAGCATCTATGCACTTCCCTACTTTCCTGTTGTGAT
CTTGAATTAACTAAACAATC
GCAGGGGCTCGATGACCCTGCAATTCTCGCTAGCCAGACAGTTTTTAGTGTAAGTGAA
ATAGAAGCGCTTTACGAGCTAT
TTAAGAAGATTAGCAGTGCAGTCAATGACGATGGGTTGATTAATAAGGAAAAGTTTCA
GTTGGCATTGTTTAAGACCAAT
AAAAAAGAGAGCTTGTTTGCTGATCGGGTGTTTGACTTATTCGACACCAAACACAATG
GAATTTTGGGATTTGAGGAGTT
TGCCCGTGCACTCTCGGTGTTTCATCCAAACGCCCCTATTGATGATAAGATCAACTTTTC
CTTTCAGCTTTACGATCTAA
AGCAGCAAGGTTTCATTGAGAGGCAAGAGGTGAAGCAAATGGTGGTAGCTACACTTG
CTGAATCGGGGATGAATCTTTCA
GACGATGTTATAGAGAGTATCATTGACAAGACCTTTGAGGAAGCTGATACGAAGCATG
ATGGTAAAATCGACCAAGAAGA
ATGGAGAAACCTCGTGCTACGACATCCTTCTCTCTTGAAAAACATGACCCTGCAGTAC
CTCAAGGACATTACGACTACAT
TCCCAAGCTTCGTTTTTCACTCGAGAGTCGAAGATATGTAA
>CsCML45
ATGGCTGCATCCAAGACACAACTAGAGTTTCATGATCATTTACCTTTGATAGAAGAAAA
GCTAGGCGGAGACGGTCTCAT
AGGTGAATTATGCAAAGGATTTGAGCTTATAATGGACCCGAATAAAGGGGTGATCACAT
TTGATAGCTTGAAGAATAACG
CGTCGTTTTTGGGCCTCCAAGACTTGAGTGATGATGATTTGATGAGCATGCTAAAGGAA
GGTGATTATGATGGTGATGGC
GCGTTAAATCAGATGGAATTTTGTGTTCTTATGTTTAGATTAAGTCCTGATTTGATGGAT

CAATCTGAGTTTTTGTTGGA
AGAAGCTCTTGAACAAGAGGTTAAGAATTCATATCAATAG
>CsCML46
ATGGTTCAGGAAGAGTTCCAGCTCGCACTATTCAGAAATAGAAACAAGCGTAATCTATT
TGCGGACAGGATATTCGATCT
ATTTGATTTGAAACGCAATGGAGTTATTGAGTGTGGAGAATTTGTTCGATCATTAGGTG
TTTTCCATCCGGATGCACCAA
CAGAAGATAAAATAAAATTTGCTTTTAGGCTTTATGATCTCAGACAAACTGGTTTTATCG
AGAGAGAGGAGTTGAACGAA
ATGGTAATAGCACTGTTAGACGAATCTGATTTGGTACTTTCAGAAGACGTTATTGAAAT
GATAGTCGACAAAACATTTAG
TGATGCGGATATTAAAGGGGATGGAAAGATAGATGAAGAGGAATGGAAGCAATTCGTA
GCACATAATCCATCTCTTATAA
AGAACATGACACTTCCCTATTTAAAGGACATAACTTTGGCATTCCCGAGCTTTGTTGTA
ACCTCTGAAGTAGAAGACTCG
GAAGTATAG
>CsCML47
ATGGGCTGCATGTGCTCTAGTGGCATTAAGCATACTCCTGGGTATGAAGATCCTGCTATT
CTAGCTAACGAAACCCCGTT
CACCGTGAGCGAAGTTGAGTCATTGTATGAGCTATTTAACAAGTTGAGCAGTTCCATTA
TCGATGATGGCCTTATTCACA
AGGAAGAGTTCCAGCTCGCGCTCTTCAGAAACAGAAACAAGCGTAATCTTTTCGCAG
ACAGGATATTCGATCTATTTGAT
GTGAAACACAATGGAGTTATTGAGCGTGGAGAATTTGTTCGATCATTAGGTGTTTTCCA
TCCGGATGCACCAACCGAAGA
TAAAATTGAATTTGCTTTTAGGCTTTATGATCTCAGACAAACTGGTTTTATCGAGAGAG
AGGAGTTGAATGAGATGGTAA
TAGCATTGTTAGACGAATCAGATTTGGTACTTTCGGAAGACGTTATAGAGATGATAGTC
GACAAAACATTTAGTGATGCG
GATATTAAAGGGGATGGAAAGATAGATGAAGAGGAATGGAAGCAATTCGTAGCGCATA
ATCCATCTCTTATAAAGAACAT
GACCCTTCCCTATTTAAAGGACATAAGTTTGGCATTCCCGAGCTTTGTTCTAACCTCTG
AGTGTGAAGTAGAAGACTCAG
AAGTATAG
>CsCML48
ATGGAGCTTACGTCCGTAGCAACTGCAGCCACAACAGGAGGTATACTGTCCATGGAGG
TCAAATATTTCATAATCTTATA
CATGTTTGTAGAGTGGGTGAAACTTTTACATGATTGTTATTTCCTTTTGGCACGACCCAT
GTGGCGGTTTATCTCTACTA
CAGAAAACACTACTTGTAGACCAACTCCAAGCACAGTTGCTCAAGCCCACGACACTAT
ACAGGTTTTCCATGCATATAAT
AGCAGGAAGAAGTCATTAGGAGTAGATCTTGAAATAGTTTTGAAAAGATTAGGAATGT
TTTGTGATCATGATGACAAGGG
ACAAATTATTGGCTCTGATGAGATATTGGGTTTGTTTGACGAGGACGAGCCAAGCTTGG

ATGAAGTGAAAGAAGCATTTA
GTGCGTTTGACAGAAACAAAGACGGGTTCATAGACGCAAAGGAGTTGCAACACGCGC
TTTCAGAGATGGGGTGTATACAA
ATATCAGAAAGCAATTGTAGACTGATGATTGGAGGATATGATGTCGATCAAGATGATAA
GATTAGTTTTAGAGAGTTTTT
GAAGCTCATGGAAGACTGTTTTTGA
>CsCML49
ATGGGTTGTATAAGTTCAACTCCGAAGGAATTCAAAAAGTTACCAGCTTATGATCCGGT
TGCGCTTGCTGCTGAGACCCC
TTTTACTGTGAATGAGGTGGAGGCTTTATACGAGCTGTTTGAGAAACTAAGCAGTTCCG
TGGTGGATGATGGGCTTATAG
GGAAGGACGAGTTTAACCTAGCGCTATTTCGAAATCATAGTAAACGAAACCTGTTTGC
CGATCGGATATTTGATCTTTTT
GACGTAAATAGGAGTGGGCATATTGACTTTAACGAGTTTGTCCGTTCGTTGAGTGTATT
TCATCCAAAAGCTCCTCAAGC
CGACAAAGTTTTGTATGCGTTCAGACTTTATGATTTAAGACGCACTGGCTTCATCGAAC
GTGAGGAGTTGAAGGATATGG
TTAAGGCTCTTCTGAGTGAATCTGATCTTGTTCTATCAGATGAAATAATTGAATCGATGG
TAGACAAGACTTTCGCAGAA
GCAGATTATAAAGGTGATGGTAAGATTGACCAAGAAGAATGGAGGGAGTATGTCGACA
AGAACCCGTCACTATTGAAGAA
CATGACTCTTCCTCACTTAATGGACATAACTTTGAGGTTTCCAAGCTTTGTGATGAACA
GCCAGGTAGAAGAGACTCAGT
CAACAGACTAG
>CsCML50
ATGTCTTATAACCCATCAGGTTACAACTATGGTGCACCACCACCTTCTCAACCATACTAC
TCCACCCCTTACGCCGCACC
GTCACCTTACGGTCAGCCACAACAGCCCGCTTTCACGAGCCCCTACGGTTATGGAACA
TCCACATTTCCACCGGGGACGG
ATCCTAATGTGATTGCGTGTTTTCAAGTGGCTGACATAGACCGAAGTGGTGTCGTTGAT
GATAAGGAGTTGCAAATAGCG
TTGTCGTCGTATAATCAAAGTTTTAGTATCCGTACTGTTCGTCTACTTATGCATCATTTTA
CAAATACCAACACTAGAAA
GATTGGACCAAAGGAATTTACTCAAGTGTTTTATAGTCTACAAAATTGGAGAGCAATTT
TTGAGAAATTTGATCGAGATC
GAAGTGGTCATATCGACACCTGGGAACTGAGAGAGGCACTCATGAGCCTCGGATTTAC
TGTTTCACCCGTTGTCTTGGAT
TTACTCGTCTCCAAGTTTGACAAGACTGGTGGATATAAGAAGGCCATTGAGTACGACA
ATTTCATCGAGTGTTGCCTCAT
TGTTAAGGGGTTAACCGAAAAGTTCAAGGAGAAAGACACAATGTATTCAGGCAATGC
GACATTCACATACGAGGCTTTTA
TGTTAACAGTTCTTCCGTTTCTCATTGTTTAA
>CsCML51
ATGGGAAACACATCATCAATGCTGACACAATACGACATCGAAGAAGTACAAGAACATT

GTAACAACACATTTTCACAACA
AGAGATAGTGTCATTGTACCAAAGGTTTTGTCAGTTGGATCGAAATAGTGGTGGATTTA
TCTCTGCTGATGAGTTTCTTT
CGGTCCCCGAATTCGCTGTTAATCCCTTGTCTCAGAGGTTGTTCAGGATGATTGATGGG
TTGAATTTCAAGGAGTTTGTG
GCATTTTTGTCTGCATTTAGTTCGCGTGCCACCTTACAGCATAAAGTGGAATTTATATTT
AAGGTGTATGATTCCGATGG
CAATGGGAAGGTTGCATTCTCTGATCTGTTAGACGTTTTGCGGGACTTGACAGGGCAA
TTCATTTCTGAACAACAAAGAG
AGCTTGTCCTGACGCAAGTTCTGGAAGAAGCTGGGTACAAGAAAGATTCGCTATTAGT
TTTGTCCGACTTCATGAAGATT
CTTGGCAACACAGGCTTGAAGATGGAGGTTGAGGTTCCCACAAACCCTTCCAAATATG
TTGGAATAATTTCAATTCTAAA
TGATGTCATTGGAACCTTAAAAATTCACAAACCCTACTCATCTTTATCACAAACACCTC
TCTTCAACTACTACTTGACAA
CACAGTTAACTGACAGGAAATGGCTCGACTTAGTTTAA
>CsCML52
ATGTCGTGCTTAGAGGGGATCAGGCATTTACTTGCTCCCCTGCTCCGTTGCTTTAATCTT
GAATCATTCACAAAAACAAA
CCCCCTTGAAGATCCTGAAGTTCTAGCCCGAGAGACTGTTTTTAGTGTAAGCGAAATA
GAAGCACTCTATGAGTTGTTTA
AGAAAATTAGCAGTGCTGTGACTGATGACGGGCTAATTAGTAAGGAAGAGTTCCAGTT
AGCCTTATTTAAGACAAATAAA
AAGGAGAGCCTTTTTGCTGATCGAGTGTTCGATCTGTTTGATACCAAGCATAATGGAAT
CTTAGGTTTCGAAGAGTTTGC
TCGTGCACTCTCTGTATTTCATCCAAATGCCCCTGTTGATGAAAAGATCGAGTTTTCCTT
TCAACTTTATGATCTTAAGA
AACAAGGTTTCATAGAGAGGCAAGAGGTGAAGCAAATGGTGGTGGCGACTCTTGCTG
AATCCAAAATGAAACTTACTGAT
GATGTTATTGAGAATATCATTAATACGACATTTCAGGAAGCTGATACGAAACATGATGG
GAAGATTGACATGGAAGAATG
GAGAAGCCTTGCTCTACGACATCCGTCCCTTTTGAAGAATATGACCCTTCATTACCTTA
AGGACATCACAACAACATTTC
CGAGCTTTGTTTTTCACTCAAGAGTTGAGGATTCGTAA
>CsCML53
ATGGGTGGTAAGTTGACCAAGGCTGATGAATCACCTAAGACTTCGGTACCTACAACCA
AGCTTGAAGCTAAAATCTTGGA
GACAATCCGTCGCAGAGAATCTAAAGGAACTTCCATGAAATCGTTCAATACTATAATCC
TGAAGTTCCCAAAAATTGACG
CAAGCCTAAGAAAGTGCAAAGCTATATTTGAGCAATTTGATGAAGATAAAAGTGGTAC
AATTGATCCGAAAGAGCTGAAT
CATTGTTTTCGTAAGCTGGAAATAGATTTTACAGATGAAGAAATCAGTGATTTATTTAAA
GAATGTGATATAAACCATGA
CATGGGAATAAACTTCAAAGAGTTCATTGTGCTCCTGTGCCTCGTTTATCTTCTGAAGA

ATGACCCTGTTTCCCCCCATT
CTAGTTCACGCATGGGGATGCCGGAACTACAAGCTTTTGAAACTTTGGTGGACTCATTT
GTGTTCTTGGACAAGAACAAA
GATGGTTATGTTAGTCGAAGCGAGATGGTTGATGCCATAAATGAAACTACAACTGGCGA
GCGGTCTTCTGGGCGCATAGC
TATGAGAAGATTCGAAGAGATGGATTGGGATAAAAATGGAATGGTGAACTTCAAGGAG
TTCATCTTTGCCTTCGAGAAAT
GGATTGGAATCGAGGATGGCGAGGAGGATGAAGAAGAGGTAGCAGACGAGCAAGAAT
GA
>CsCML54
ATGGCATCAACCAATAATATGCAATCTGAGTTCCAAGATTACTTGCCATTAATGGCTGAT
AAGTTGGGTGGTGATGGTTT
AATACAAGAACTATGTAATGGGTTTCAGTTACTTATGGATCAAGATAAAGGGGTGATTA
CTTTTGATAGTTTGAAGAAGA
ATTCATCAGTTTTGGGGCTTGAAGGATTGAGTGATGATGAAGTCATGAGTATGCTGAAA
GAAGGTGATTTTGATGGTGAT
GGTGCTTTGAATCAAATGGAGTTTTGTGTTCTTATGTTTAGATTAAGTCCGAATTTGATG
GATCAATCTGAATACTTGTT
GGAAGAAGCTTTGGAACAAGAGTTAAATAATTTTCAATACTGA
>CsCML55
ATGACAACCAATTATAATTCTAGTTTTCATGATTTCTTGCCGTTAATGGCGGATAAGTTG
GGTGGTGATGGTTTGGTAGA
TGAACTATGCAAAGGGTTCGAGTTGCTGATGGATCAAGATAAAGGGGTGATTACTTTTG
ATAGTTTGAAGAAGAATTCAT
CAGTTTTGGGGCTTGAAGGATTGAGTGATGATGAAGTTATGAGTATGTTGAAAGAAGG
TGATTTTGATGGTGATGGTGCT
TTGAATCAAATGGAGTTTTGTGTTCTTATGTTTAGATTAAGTCCAAATTTAATGGATCAA
TCTGAATACTTGTTGGAAGC
AGCTCTTGAACAAGAATTTAATTATTTGTGA
>CsCML56
ATGAAAGCCGCTATTACCGGTCTGTTTAACACTCAAGAGGTAGCTCAAGAGTATGTCGT
GACTGGAGTTGGTTTGCTAGC
CGGATCAACGATATTCCTTCTCACTTTATTATGGGGAACTTGTATCATTATTGGAAGCCA
AAAGTTTTCTTCAGAATCGG
GTGCCAGTACTTCCGTGGATCCTACCCAATGCCCAAACAAGAAATTCTTCTCATTTCTT
ACTAGTTCTGGTGTAACTACA
GACCCCGAGACATGTACAGCGGCGCAAATCATGCTTCTGTCAGTTATACCGTTTCTGTT
TCTGTTGATCCCGAAGTTGTT
TGGCATGACGTATGCACCTCATGGATACATTTTTTTAATAGCACTTCCTGTTTCAGTTAC
CTTCTTGCTGGTATACTTCA
TTTATCAGGTGTTTGAGCCTTCAATTCAAAAGAGACGATTGTCATACGTCAAACACGAG
CATTTAGTTTTAGACATACTA
AAACATTTACAGGAGCAAATCCCAGAAAATATACTCGCTGAAGATGGTTCAGTCAACC
TGCCTGCTATAAAAAGTTTGTT

TAAGAAGATTGATCAAGATGGAGATGACATCATATCTTTTTCTGAGCTCAAAGAACTTC
TCGAAAGTATTAAGTTCAGGC
AATTAAAATCGGACAAACAGAAAACATTCGATCAACTGATAAAAGAATTTGATTCTGAT
GGTAACGCACAAGTATCGCTA
GATGAATTCATCCATCGGTTCACAGAATGGCTTGATGAGGCTAAAAATGAATTATCTGA
GGTCGTCAAACCATTGGTTCA
AACCAAAAGGAATGAAGATGACATGACGCAAGTCCTCGTATCTGAAATTATCGGCAAT
GCTAAAAGCTCTCCACTAGGGA
AGTTTTACAAAGAAGATGGAACACCGGACATATCTGCCATAAAAAAATTGTTTAGGAG
CTTGGATGTCAACAAAGATGGT
TCTGTGTCGTTAACCGAGTTGAAAAAACTTACAATGCATGTCAACTTGGGTGAGACAT
CATGGAATGTGGATGAAACAAC
ATCTCGCATAATGCAAAATCTTGATACAAATGGAGATAAAGAGATCGATGAACAGGAAT
TCGTTGATGGATTTGAAAAGA
AATTGGTGAACATAACTAATGACCGATCCAAGACATCTGGGCCTAAAGATGTATCACGG
AAAGCATGCAAAAAATGGAAA
GGTGATAATGTGGATAGATCTGTGTGGGGATGGACAAAGGCTATAATGCTACTGGTGCT
CGGGATAGCAATGTTGGCTCT
GATGGCTGAACCTCTTATACACAGTGTTCAAAATGTCTCCAACTCGGCTGCCATGCCGT
CCTTTTTCATATCGTTTATCT
TGGTTCCATTAGCCACAAATGCTAGAGCAGCTATCTCTGCAATCCGAACTGCAAGTCAA
GGGAAAGAACGAACTACTTCA
TTAACATTTTCTGAGCAAGAAAGGATCAATTGTTTTGATCAAAGCAAAGCAAGCATGG
TAGAAGATGACAAGCATGATAA
CGCATCAAGCAGTGCATCAATTCGAGAAGGGAATGTTTCACTTCAATGTCCAAAACTG
ACAGATACTAATTATACAACTT
GGGCACTGATGATGAAAACAATATTAAAGGCGTATGGACTTTGGAAAGTGATTGATGG
CATGAAAGAAACTAGCGGCTAG
>CsCML57
ATGGGTACATCTCACACAAGCCCTTCATTCAAGTCGTTATCCAACAAAGTCGGAGTTAT
GCTATGTTGTTGCAATTCACA
AAACCGATACGAGAGATTAGACAATAAGCTCGAAAGAAAAATGATGGAGGTCAAACA
GAACAGCATACAAGGTCAAACCA
GTTTCAGATCTATCGATAGCATTATCTTAAGGTTCCCGCGGTTCAAAGAAGGATTAAAG
GAGATTCAAGGTGTTTTTGAA
CTATACGATGAAGATTCAAATGGAACCATTGATAACGAGGAGCTAAAGAGATGCTTACA
GAAGCTGGAATTTCATTGTAC
CGAGCAGGAGATTAGAGATCTTTTCGAGTCTTGTGATGTGGATGGGAGCAATGGGATA
CAGTTTAACGAGTTTATTGTTC
TTCTGTGTCTCATTTATCTCTTAGACGGTCCTTCCTCATCATCTCATGTGACATCGACAG
TGGGGTCACCCGAGCTCAAA
GCAACATTTGATACCATCATCGAAGCTTTCTTGTTTCTTGATAAAAACGGTGATGGAAA
GCTGAACAAAAAGGACATGAT
GAAGGCAATGAATGAAGACTTCCCAATGGAGAAATCACCTACACATATCACCAAGACC

CGATTCAAAGAAATGGATTGGA
ACAAGGATGGTAAGGTGAGCTTCAGGGAGTTCTTGTTCTCTTTAATTAACTGGGTCGG
GTTAGAATCCACTGATGAAGTA
CCTGAAACGGTGTTTTGA
>CsCML58
ATGTCATACTCCGGTTACAATCCAAACACCACCATTCCATCAGCCCCACCCGCACCACC
CTCCCAATCCCACCAACCCGC
AACCGCATACCCTTACCAACAACCACCACCTCAAAATTACAACAACCAACAAACCTAC
AACCCCTCCGGGTATGGAGGCG
GCTACGGGTCCCAATACGGGTCGTACCCGCCTCAACAAACGGTGTCGTTTCCACCAGG
GACGCACCCCGAAGTGATTCGA
AGCTTCCAGGCTGTTGATTTGGATAGGAGTGGGTTTATTGATGCTAAGGAGCTTCAACA
GGCTTTGACTCAGGCGTATCT
CAAGTTTAGTTCCAGGACTATTAAGCTGCTCATGTTTCAGTTTAGGAACCCCACTGATC
CCACACGAACCGGTCCTAAAG
AGTTTGCTGAGTTATGGAGTTGTCTTGGCCAATGGCGGGCGATCTTTGAGAGGTTTGA
CAGGGATCGAAGTGGAAAGATT
GATCTAGCGGAACTAAGAGATGCCTTATACAGTCTTGGATATGCAATTCCACCTTCTGTC
CTACAACTCTTGATTTCAAA
ATACGACGACCAAAGTGGAAGGAGGGTAGATCTGTCCTTTGACAGCTTTGTTGAGTGC
GGAATGATTATAAAGGGTTTGA
CGGAGAAGTTCAAGGAGAAAGATACTAGGTACACGGGTTCAGCCACGCTTTCATACGA
GACATTTATGACCATGGTCATC
CCATTTCTTGTAGCAGAATAA
>CsCML59
ATGGCCTTCGGTTCTCAGTCGAATGTTCTCGATCCTCCTCCAAAGTTTGATATGTACAA
ATATACTTTTGTCATGACTGA
CGCTGACCTGGAGGGTGTTGTCGCTGGGTATGGCCACCCTTGGATAAGTGAAGATGGT
GTTGCTCCAGACAAGCCTCTTG
ATTCTGCAGTCTTGAGCCGCTTAACTCAGTTTTCTGCCATGAACAAGCTCAAAAGAAT
GGCTCTTAGGGTCATCGTGTCA
AAACTCTCAGAAGAAGAAACTGCAGGCTTAAAACAAATGTTCAAGATGATAGACACA
GACAAGAGTGGTTATATTACATT
TGAAGAACTAAAGGCTGGACTCAAGCGATTCGGTTCTACTCTTAATGAGTCTGAGATTT
ATGATCTAATGCAATCTGCGG
ATATTGATAATGGTACTATCGACTATGAAGAATTTGTAGCATCAACGTTACATATGAACA
AAGTTGATAGAGACGATCAT
TTGTTTGCTGCTTTTTCATATTTTGACAAAGATGATAGTGGTTATATAACTCTTGATGAAC
TCCAACAAGCGTGCAAAGA
GTTTGGATTAGATGATGTTCATTTAGAAGAAATTATCAAAGAAGCTGACCAAAACAATG
TACGTTTTTCACTACCATAA
>CsCML60
ATGGCGGAACAACTGACTGAAGAACAAATTGCTGAGTTCAAGGAAGCTTTCAGCCTC
TTTGACAAAGACGGTGATGGCTG

TATCACCACCAAAGAGTTGGGGACAGTTATGAGATCATTGGGTCAGAATCCTACTGAA
GCTGAACTGCAAGATATGATCA
ATGAAGTTGATGCTGATCAGAATGGAACAATCGATTTCCCTGAGTTTCTGAACCTGATG
GCCAGGAAAATGAAGGACACT
GATTCGGAGGAGGAACTCAAGGAAGCTTTCAAGGTCTTTGATAAGGATCAGAATGGAT
ACATTTCTGCTGCTGAACTTCG
CCATGTGATGACAAACCTCGGGGAGAAGTTAACTGATGAAGAAGTGGATGAAATGATC
CGTGAAGCTGATATGGATGGTG
ATGGTCAAGTGAATTACGAGGAGTTTGTGAGGATGATGCTTGCCAAGTGA
>CsCaM3
ATGAGGTCTCTAGGACAAAACCCAACCGAGGCTGAACTTCAAGATATGATCAACGAAG
TCGATGCTGATGGCAATGGTAC
TATTGATTTCCCTGAGTTTCTCAACTTGATGGCCCGCAAGATGAAGGACACTGATTCTG
AGGAAGAGCTCAAGGAGGCTT
TCCGGGTTTTTGACAAGGACCAAAATGGTTTCATCTCTGCGGCTGAGCTTCGTCACGT
CATAACTAATCTTGGTGAGAAG
TTGACAGATGAGGAAGTCGATGAGATGATCCGTGAAGCTTGA
>CsCML61
ATGAGTAAATCGAACGATTACAAGCGTGTATTTGATCACTTTGATGAAGACAGCAATGG
CATGGTCTCACCATCCGAGCT
ACACCGCCGTGTAGGCATGATTTGTCACGAGCAAGTTTTAATAGAAGATGTACAAGTTA
TAGTCGAGTCATTGCATGGGA
GCAAAGTTGACGGGCATGAACTAGGATTTGACGATTTCGTTAGTTTAATGGAGAGCGA
TAATGAAGATGAGAAGGTTGAG
GATTTAAGGAAGGCTTTTAGATTGTATGAAAACGATGGAAACGATTGTATAACACCGAA
AAGCTTGAATCGGATGTTGGA
TCGATTAGGTGAGTCGAGAAGTGTTGATGAGTGTGTTGGTATGATTAATCAGTTTGATC
TGAATGGTGATGGTGTGCTTA
ACTTTGAAGAGTTTAAAGCAATGATGCTTTGA
>CsCML62
ATGAATAGTTGTGAAGTTTTGGTAAAATCCCTTCAAGCTTCGGTTCAATCGCTATTGAC
CCATGTTTCAATAACATGGAA
CAAGTTAGGATCCATTTCCACATCACTTGACCTAAATGAGACCGTAAAGAAAGAGTTC
ATGACCCTTGGAGAGATAAACG
TGATCATGGGGCAACTAGGGCTCCAACAACGTTGTAGTGATCAGGATAGCAATATTGAC
ATTTTATCGGTGTTTGATGAT
GAGGAACCAACTTTGGAAGAAGTTAAGGTGGCTTTTGACGTGTTTGATGAGAACTCG
GACGGTTTCATAGATGAGAATGA
GTTGCGTGACATGTTATGCAAGTTAGGAAAACAAGAAAACGCAATGTTAAAGGAATGC
CGGAGTATGATAAAAGGATTTG
ATGTCAATGGGGATGGGCTCATTGACTTTGATGAATTTGTTAGGCTCATGGAGACATGT
TCTTTTTAA
>CsCML63
ATGGGTTTAAAGAATCTATTCAACCGTAAAACCAAGAACACGACCAAAGACAATACTA

CTATGGTCGAAGAAACAATCGC
CACAAACACAACGTTGCCCGTTGTACCTCGTCAACAAACCAAGGAACAACAACTAGA
GCAAGTTTTCAAGAAACTCGACG
TGAACAACGATGGAAAAATCTCTTACTCCGAGCTAGGATCAGTGATGGGGAGCCTAGC
AGGAAACCAACCAACGGACGAC
GAGTTAAAGAAAATGATCATGGAAGTTGATAAAGACGGGGACGGGTTCATAGACTTGG
AAGAGTTTATTGAGTTGAACAC
AAAAGTCGATTCTAGTGAGTTGTTGGAGCTTATAGAAAAAGCGTTTTCCATGTTTGATG
TTGATAAAAACGGCTTGATTA
CGGTTGAAGAGTTGTTGAGGGTTATGCGGAGCTTGCATGAAGATTATAGTATTGAAGAG
TGCAAGAAGATGATTGCTGGA
GTTGATCAAGATGGTGATGGTATGATTAATTTGAATGAGTTTAAGGTTATGATGATGAGT
GGAGTAAGGTCAGATGTTAG
TGAATCTTGA
>CsCML64
ATGAATACAGGCGAACCATCAACTTCATCTTCTCCGGTCAAATTCTTCCGACCCCCCAC
CACCATATCACCTCCGTCATC
GTCTCCATTACCAACTCCGTGCACCGACGACATCCATCAACTCTTCAACTACTTCGATG
AAAATGGCGACGGTAAAATAA
CCGCAACGGAGCTTCAAAACCGGTTAAAAACCGTCGCCGGAGATGAAGTTCAGTTATC
CGACGAGGAGGCGGAGATGGCG
GTGAGGTCATCGGATGCTGACGGTGACGGTGTGTTAGGGTTTGATGATTTCACGAAGA
TGATGAAGGAAGGGGCGGAGGA
GGAGTTGCGAGAGGCGTTTCGGATGTATTCAGCGAAATCGGGGACTGTTATTACGGCC
AACAGTTTGAGAAGGATGTTGC
GGCGGTTGGGCCAAAGTACGGTTACGGTGGAGGAGTGTAAGGGGATGATTGGGAGAT
TTGATGTTAATGGTGACGCCGTG
TTGGATTACGATGAGTTTCGAGCTATGATGAGTTAG
>CsCML65
ATGGAAACACAAACACCTACATCTAAACACGCTCCTCTTCTTAAATCATGTTCCAACGG
TTCGTTTCGTCTGCGTTCACC
CAGTTTAAACTCCTTACGTCTTCGTCGAATCTTTGATCTATTCGACTCTAATCACGACTC
ATTTATCACTATAGAAGAAA
TCACACGTGCCTTGACCCTTCTAGGCCTCGACACCAATGCCTCGGATTTGGACACTATG
ATCAAATCATACGTGCATCCA
GGCAACGTTGGGCTAACCTATGAGGACTTTGTGACATTACATAGGTCCATAAATGACTT
GTTTTTCGGTATGGACGAGGT
AGAGGAAGCTGTGGGAAGTAAAGAGGACCAAGAGGAGGCGGACTTAAACGAGGCGT
TTAAGGTTTTTGATGAGAACGGGG
ACGGGTTTATATCGGCTGCAGAGTTGCAAATGGTGCTTGGAAAGCTCGGCTTTACTGA
AGCTATTGAGATGGGGAGAGTT
AAGATGATGATCTCGTCTGTTGATCTTAACCATGACGGATGCGTTGACTTTTCTGAGTTT
AAAGACATGATGCGGGTGCT
CCAATAG

>CsCML66
ATGTGTGATGACTCAGGCGTGCCACATAGATTCGTGCTTGATATACAAGTTTTGCAAGA
GATCTCATTGCTAGATTCTGT
TGCTGAAACGGCAGTTGAGATGATGAACATCCTAAAGAAAATAAAGAAAAATGTCAA
AACCGCATCAAAGCCTATTTCAA
ATAAAATAAAACATGATCCAGAAAAGAAACCAAAAAGTATAGTTGATGTTGGTTGTGG
AATAGGTGGTAGCTCAAGGTTT
CTAGCTAAAAAGTACGGAGCTAAATGTCGTGGAATTACTCTCAACCCTGTACAAGCTG
AGCGGGCTCAGGTACTAGCTGA
TGCTCAAGGATTGGGCGATAAGCCACTATCTGAAGTACCTCATCAAGGCATACCTCGGG
TAAGTATCTTGGTTTTGGATT
CTTTTCAAGCTCAACTCGATGCATTAATACAAAAAGGCGATACAAATAGCAATGGTTTG
GTTGATTTTTCTGAGTTTGTA
GCACTCGTGGCACCCGAGCTTCTTTCTGCTAAATTGCCTTATACAGATGATCAACTGAA
ACAGCTGTTTAAGATGTTTGA
TAGGGACGGAAATGGTTATATAATGGCTGCTGAGTTGGCTCATTCTATGGCGAAACTAG
GACATCCTTTGACGGCTGAGG
AACTTACTGGGATGATCAAGGAAGCTGATACGGATGGGGATGGACGGATTAACTTTCA
GGAGTTTTCTTGCGCGATTACT
TCAGCTGCTTTTGATAATTCTTTTTCATGA
>CsCML67
ATGTGTCCAACAGGAACATCCTTATTTCCATCAAGAAACATAACCAACCTACGTTCCGC
ATTCGACATCCTAGACGTGGA
CCATGATGGCAAAATCAGCCATGAAGATCTCAAAACATCTTACTCCCATGCAGACGAC
AACATCATAGGTACGATGATGA
AAGTAGCTGACTCAAACAACAATGGGTACGTAGAATACGATGAGTTTGAGAAGGTGGT
GTTGAAAACTGATGGTAGTAAT
GTTTATGGTGTTATGGAGGATGTGTTTAAGGCTATGGATTGTGATGGTGATGGAAAAGT
TGGATATGGAGATTTAAGAAG
CTATTTGAATATGGCTGGTTTAGATGTTAATGATGATGAGATTAAGGCCATGATAAGATT
TGGTGGTGGTGGTGATTATG
ATGATGGTGTTACTTTTGATGGATTTATCAAGATATTGTCTCTTTGA
>CsCML68
ATGGCGAGAGTACTGGCCGTCCAACAACGTAAACAACTACTTGAAATTTTTAAACAGT
TTGACATGGACTCAGATGGATC
CTTAACATATCTTGAGCTAGCAGCCCTCCTTCGCTCTATAGGCCTTAATCTCTCGGGTGA
CCAAATCTATACGCTCTTTA
ATAAAATAGATTCTGATGGAAATGGGAAGGTTACATTTGAAGCATTTGTTGATGCAATG
ACGATTGATGTGAAGACAGAA
GAGATTGTTATTGATCAGAGACAACTTTTTGAAGCTTTTCGCTCATTTGATAGAGAGGG
AAATGGATTCATCACACCCAC
ACAACTAGCTATATCAATGGCTAAGATGGGTTATCCATTGACATACCATGAGCTTGTAGC
TTTCATAGAGGGTCTTTTTG
TAGGACTCACTAGGACGTTGGAAAGATTGGTATCGGAGCTCGCTGATTTCTGTATAGAA

CTGTGTAGGACGTTAGAAGTT
AAAGTGGGGATGCAGAGATTGACATCCCACCCCACGTATGTAGTATTTTGA
>CsCML69
ATGTCATCAAATAACGAAGCCAACTTTCATGATTTCTTACCCATAATGGCGGATAAACTA
GGTGGTGAACGACTAATGGA
TGAGTTATGTAACGGGTTCCGAATGCTAATGGACCCTATCAAACGTGTTGTCACGTTTG
ATAGCTTAAAGAAAAACTCGG
CCGTATTGGGTATTGGAGACTTGACTAATGATGATGTGTTGAGTATGTTGAAAGAAGGC
GATTTAGACGGTGATGGTGTT
TTGAATCAGATGGAGTTTTGTGTTCTTATGTTTAGATTAAGTCCTGATTTGATGAAACAA
TCTTGGTTTTTGTTAGAAGA
AGCTTTAAGAACAATAGTTGAATTTGAATCTAATTAA
>CsCML70
ATGCTTACACTTGACCCGAAGAAGCGCATAACCTCTGCTCAAGTCCTTGAGCATCCATG
GATTAGAGAAGACGGAGAAGC
ATCAGACAAACCAATCGACAGTGCGGTTCTTTCAAGGATGAAGCAATTTAGAGCTATG
ACTAAACTCAAAAAACTTGCAC
TCAAGGTGATTGCTCAAAATCTAACAACAGAGGAAATTCAAGGGTTAAAATCGATGTT
CATGAACATGGACACAGACAAA
AGTGGCACAATCACCATCGAAGAACTTAAAACCGGGTTGGCTAGACTCGGGTCAAAG
CTCACAGAATCTGAAGTTAGACA
GCTCATGGACGCCGCTGATGTTGATGGAAATGGGTCGATTGATTACATTGAGTTCATTA
CGGCAAGAATGCATCGACACA
AACTTGAACGTGAAGAAGATTTGTACAAAGCGTTTCAGCATTTTGATACAGATGGTAG
CGGGTTTATTACAAGAGACGAA
CTAGAAAATGCAATGAAAGAAAATGGATTGGGCGATGAAGCTACCATAAAAGATATCA
TATCAGAAGTTGACACCGATAA
CGACGGGAAGATAAACTATGAAGAGTTTTGTACAATGATGAGAAGTGGAACCCAAGG
AGCAAAGCTGTTTTAA
>CsCML71
ATGCTTACACTTGACCCGAAGAAGCGTATAACCTCTGCTCAAGTCCTTGAGCATCCATG
GATTAGAGAAGACGGAGAAGC
ATCAGACAAACCAATCGACAGTGCGGTTCTTTCAAGGATGAAGCAATTTAGAGCTATG
AATAAACTCAAAAAACTTGCAC
TCAAGGTGATTGCTGAAAATCTAACAACAGAGGAAATTCAAGGGTTAAAATCGATGTT
CATGAACATGGACACAGACAAA
AGTGGCACAATCACCTACGAAGAACTTAAAACCGGGTTGGCTAGACTCGGGTCAAAG
CTCACAGAAGCTGAAGTTAGACA
ACTCATGGACGCCGCTGATGTTGATGGAAATGGGTCGATTGATTACATTGAGTTCATCA
CAGCAACAATGCATCGACACA
AACTTGAACGTGAAGAAGATTTGTACAAAGCTTTTCAGCATTTTGATACAGATGGTAGC
GGGTTTATTACAAGAGACGAA
CTAGAAAATGCAATGAAAGAAAATGGATTGGGCGATGAAGCTACCATAAAAGATATCA
TATCAGAAGTTGACACCGATAA

TGACGGGAAGATAAACTATGAAGAGTTTTGTACAATGATGAGAAGTGGAACCCAAGG
AGCAAAGCTGTTTTAA
>CsCML72
ATGACTCAGTGCTTAGAGGGGATCAGGCATTTACTTGCTTCCATATTGCGCTGCTGTGA
TCTTGAATTGTACAAACAATC
AAGAGGCCTCGACGATCCTGAAATTTTAGCTAGAGAGACAGTGTTTAGTGTAAGTGAA
ATTGAGGCACTTTATGAGTTAT
TTAAGAAGATTAGCAGTGCAGTTATTGATGATGGTTTAATAAATAAGGAAGAGTTCCAG
TTGGCGTTGTTTAAGACTAAT
AAAAAAGAAAGTTTGTTTGCTGATCGGGTGTTTGACCTATTTGACACAAAGCATAATG
GAATCCTGGGATTTGAGGAGTT
TGCTCGTGCACTATCAGTATTTCATCCTAATGCTCCTATTGATGATAAGATTGAATTTTCC
TTTCAATTATATGATCTTA
AGCAACAAGGTTTCATAGAGAGGCAAGAGGTAAAGCAAATGGTGGTAGCTACCCTTG
CTGAGTCTGGCATGAATCTCTCA
GACGATGTCATAGAGAGCATTATTGACAAGACGTTTGAGGAAGCTGATACAAAACATG
ATGGGAAGATTGACAAGGAAGA
ATGGAGGAGTCTTGTATTACGACATCCATCGCTTTTGAAGAATATGACGCTTCAGTACC
TTAAGGATATCACGACGACAT
TTCCAAGCTTTGTTTTTCACTCGAGAGTTGAGGATACATAA
>CsCML73
ATGGGTTTGAAAAATCTCTTAAATCGAAAGAAGAAAAAGAAAACAGGAGATGATAAC
AGCACTCACGAATCTGAACCTCA
ATCTGAACCCGCGTCACAAACAACAAACACGTCGAATAATATCAATGCCGAGGCATCG
CAAGCCAAGTCTTTGGATTCAC
GCGTACGTATTGAAGAGGAGCTAGAGCAAGTTTTTAACAAATTTGACGTAAATGCTGAT
GGAAAAATATGCGCGGCAGAG
CTAGGATCCATAATGGGAAGCCTTGGACACCACCCGTCACAAGAGGAGCTAAAAAAC
ATGATCAAGGAGGTGGATGCAGA
CGGGGACGGCTTCATAAATTTGCAAGAATTTATTGAGTTGAACACGAAAGATATTGATT
CGTCTGAGGTGTTGGAAAATC
TAAAAGACGCGTTTTCTGTTTTTGATATTGATAAAAATGGCTTGATCACTGCAGAGGAG
TTGTTGAATGTTTTGAGTAGT
TTAGGAGAAAATTGCACTATCACCGAGAGCAAGAAAATGATAGCTGGTGCTGATCGTG
ATGGCGATGGTATGATTAACTT
TGACGAGTTTAAGGATATGATGATGTCTGGTTCTCGGTTTGATTCTGGGTTGCAAAAAC
ATGAGATAGCGAAGGAAGATT
AA
>CsCML74
ATGTCACCCGAAACATCCAACCAATCGCCATCCGTATTCCCAACGGACAAAGAAGAAA
TCAAGACCATCTTTAACCGTTT
CGACACAAATGGTGATGGAAAGATCTCTGAGGACGAGCTAATCAATGTCTTGAAATCA
CTAGGATCCGACACGTCTCCTG
AAGAGGTCAAGCGCATATTGACAGAAACTGATACCAATTCTGATGGTTTCATTAGCCTA

GACGAGTTTGTTGTATTTTGC
AAAGGAATTGCGGGTGAATGTGATGGTGACGGGCTTAATGATCTTAAGGAAGCATTTA
AGCTATACGATCAAGATAATAA
TGGAGTCATTTCAGCTAGCGAGTTGCATCAAATATTGAGCGGAATGGGGTTGAATTACA
CGCTTAAGGATTGCGAGAATA
TGATTAACTCGGTTGATTCAGATGGTGATGGTTGCGTCGATTTTGAAGAATTTAGGAAA
ATGATGTCGAAGAATTAA
>CsCML75
ATGGCCGAAAAATTGACAGACGATCAAATTACCGAGTTCCGACAAGCATTTTCAATGA
TCGATAAAGATTCTGATGGATT
GATTAGTACAGAGGATTTAATAGGCGTGATTCAAACATTGAACGAAAACGCTACTAATG
AAGAGGTTAAAGAAATGATGA
ACGAAGTAGATACAAATGAAGAAGGCACAATCGATTTCCATGATTTTCTTAATATCATGT
CCAAAAGAGTTAAAGAAAAT
GCGAGTGACGAGCTAAAAGAAGCTTTCAAAGTATTCGATCGGAATCAAGATGGTTACA
TTTCACCTGATGAGTTGCGAAA
TGTTATGATAAACTTGGGGGAGCGATTAAAAGATGAAGAGCTGGAGCAAATGATGCGA
GAAGCTGATCTTGATGGAGACG
GTGTTATAAGTTATGATGAGTTCGTTCGGGTTATGATGAACTCCTCCTGA
>CsCML76
ATGTTTGATCATGATGGTGATGGTAACATCACCATACAAGAGCTATCAAAGTCTCTTGA
AAGCCTTGGTATGGTTATACC
CGAGAAAGATCTTGAAAACATGATCAAACATATTGACACAAATGGTGATGGTTCCGTG
AACATGGAGGAGTTTAAAGGGT
TGTATGAAACAATAATGGAGGAGAAGGACGAAGAAGAGGATATTAAAGAAGCGTTTA
ATGTTTTTGACAAAAACGGAGAT
GGGTTCATCTCGGTTGAGGAGCTTATGTCCGTTTTAACTTCACTTGGATTTAGACAAGG
TCGAACCATCGAGGATTGCCA
ACTTATGGTCAAGAAGGTGGATGAGGATGGCGACGGGATGGTTAACTATAAAGAGTTC
AGGCAAATGATGAAAGGTGGCG
GCTTTGCATCAATGTAA
>CsCML77
ATGGGAAACGATGATCATCTTAAAGATGCCTTTGCATTCTTTGATAAAAACAAAAGTGG
GTACATAGAGATTGAAGAACT
AAGGGAAGCCTTATCCGATGAAGATGAAGCCAACAGTGAAGAAGTAATTTCGGCAATC
ATTCATGATGTAGACACCGACA
AGGACGGAAAGATTAGTTTTGAGGAATTCACGGCAATGATGAAGGCGGGAACGGATT
GGAGAAAAGCATCACGACAATAT
TCGAGAGAAAGATATAATAATTTGAGCTTGAAATTGTTCCAAGATGGATCATTGGTTTC
AGCAAATGAGGGAAGATGA
>CsCaM4
ATGGCGGATCAGTTGACCGATGATCAGATCTCTGAGTTTAAGGAAGCTTTCAGTTTGTT
TGACAAGGATGGCGATGGTTG
CATCACCACAAAGGAGCTTGGGACTGTGATGAGATCCCTAGGGCAGAATCCCACAGA

AGCTGAACTTCAGGACATGATTA
ATGAAGTTGATGCTGATGGAAACGGAACCATTGATTTCCCTGAGTTTCTAAATCTTATG
GCCAGGAAGATGAAAGATACT
GATTCAGAGGAAGAACTTAAGGAAGCATTCAGAGTTTTTGACAAGGACCAAAATGGC
TTCATTTCTGCTGCTGAATTGCG
TCACGTTATGACAAATCTTGGTGAAAAGCTTACTGATGAAGAAGTTGATGAGATGATCC
GAGAGGCTGATGTTGATGGTG
ATGGTCAAATCAACTACGAGGAGTTTGTTAAGGTTATGATGGCCAAGTAA
>CsCML78
ATGAAGCTTCCCGCCAAAATCAACCCTAAGCACATCTTCCGATCAAGAAAACACAAAA
CAGTCACCAGATCTGATCAATC
TTCATTCAGTTCCTCCAATACGACGTCGTCAGACTCGCCAGAATCCAGTCATCACCGTC
GCAAGGCCAATACTTCCGGCG
TCACTACTCCGACGAGCGTCCTTCCTTCCTCCGCCGACGACTACTCCGACCTTCAACTC
GACCTAATCCAAGCCTTCCGA
TTCATCGACACCGACGGCGACGGAAAAATCACAACGCAAGAACTCGAAACGATCCTA
AACCGTATCGTAAGATCTGAGCC
GTTGATTCAATCAGAGCTGAAATCGATGTTAACCGAGATAGACAGTAACGGCGACGGC
GTTATCACTTTAGAAGAATTTG
GAGCGGTTAGTGAAGCTTTTGGACCGGCGGTTGGCGACGGAGAGTTAAAAGAAGTGT
TTGAGTTTTTGACAGAGACGGT
GACGGAAAGATAACGGCGGATGAGCTGTATGAGGTTTTTGTTTCGCTAGGTGATGGGA
AGGTGACTGTGGAGGAATGTGT
TGGCATGATTAAAAGTGTTGATGTTAATGGAGATGGGTTTGTTTGTTTTGATGATTTTAG
GAGTATGATGGAACAAAGAT
GA
>CsCML79
ATGAGCACATACAAAGACCGAAGTTGCCGCGCTACATCTCCAATGCCTAAATCCTTGCA
AACACGCATAAGGAATCTCCT
TAAGAGAGTCAACTTCTTAACCAAAGTCAATAACTTCAAGAAAAAACCACCCAAGAAT
CCTTGTGTTCTAGATGCCATCT
CATCATTTATAGCCATGGATGTCTCAAACCAACTCAAGCAAGTCTTCAAATTCTTCGATA
TCGACGGTGATGGCAAGATT
TCTCAAGTGGAGCTCACAAATGTTTTGTTGACTTTTGGTCAAGAGAAGTCAATGGCCA
CAAAAGAAGCTCAAGGAATACT
TAAAGAAGTTGATTTTAATGGAGATGGTTTCATTGACTTGGATGAATTTATGACCATTAT
GGATGGTTCCAAGCCGGTTT
TCGCTAGCTCAAAGGAAGATAATGGTGATGATGATCTTAGAAATGCTTTTATGGTTTTTG
ATAGTGATAAAAATGGACTC
ATTTCTGCAAAGGAGTTGCAAAGTGTGCTCACTAGTCTTGGATGTAGTAATTCCAAACT
TGGACAATGTAGAAAAATGAT
TAAAGGTGTTGATAAGGATGGTGATGGATTTGTAGACTTTGATGAATTTAAGTCAATGA
TGTCCATCGGGATTAAGTAG
>CsCML80

ATGGCAGAAAAAAGCTACCAAGACTTGCTTCCTATCATGGCTGAAAAGCTAGAGTTAA
CAACCTTCATGGAGGAGTTATG
TAGCGGTTTTCGTCTGCTAGCTGACGAAAACACCGGCCTGATCACCCCTGAAAGTTTG
AGAAAAAATTCAAGTATATTGG
GTATGGAGGGAATGAGCAAAGAAGATTCAGAAGGTATGGTGATCGAAGGAGACCTTG
ATGGAGATGGTTTCTTGAACGAA
ACCGAGTTTTGCATACTCATGGTGAGACTTAGTCCCGAAATGATGCAAGATGCTGAGAT
GTGGTTGGACAAAGCGATTGA
AGATGAGATCAAGAACGTCTCTACTTCATTACCAGATAATAAAGTTTAA
>CsCML81
ATGGCATCAACCAATAATATGCAATCTGAGTTCCAAGATTACTTGCCATTAATGGCTGAT
AAGTTGGGTGGTGATGGTTT
AATACAAGAACTATGTAATGGGTTTCAGTTACTTATGGATCAAGATAAAGGGGTGATTA
CTTTTGATAGTTTGAAGAAGA
ATTCATCAGTTTTGGGGCTTGAAGGATTGAGTGATGATGAAGTCATGAGTATGCTGAAA
GAAGGTGATTTTGATGGTGAT
GGTGCTTTGAATCAAATGGAGTTTTGTGTTCTTATGTTTAGATTAAGTCCGAATTTGATG
GATCAATCTGAATACTTGTT
GGAAGAAGCTTTGGAACAAGAGTTAAATAATTTTCAATACTGA
>CsCML82
ATGAGTCAACTAAAATCATCGTCGTTTCGTCTTAGATCACCCTCGTTGAATTCTGTCCGT
TTACGTCGTATTTTTGATTT
GTTTGATACCAACCATGATGAGTTAATCACGGTTGATGAACTTAGCCGCGCTTTGATAC
TTTTGGGACTTGATACTAATA
TGAATGAATTGGATTCGATGATTAATACGTTTATTCAACCAGGGAATGCTGGGCTTACGT
TTGATGATTTCCATGCTTTG
CATAAGGAAATTGATGATTTGTTCTTTCGTCTTGATGATAATGATGATTTAGGTAATCAA
GATGAAGATAACGACGAGGC
TAATGGCGATAAGCAAGAGGAGGCGGATCTAACTGAGGCATTTAAGGTTTTCGATGAA
GATGGAGATGGCTACATATCAG
CGACCGAGTTACAAACTGTGCTTGTGAAGTTAGGGTTTGCCGAAGGTAATGAGATTGG
AAGTGTGGAGAGAATGATATCA
TCCGTTGATCGAAATCATGATGGACGTGTTGATTTCACAGAGTTTAAAGACATGATGCG
TAATGTGATCGTTCTCAAGTA
A
