# Supplementary material for: Genome-Wide Identification and Expression Analysis of CsCaM/CML Gene Family in Response to Low-Temperature and Salt Stresses in Chrysanthemum seticuspe
Source: Plants (Basel). 2022 Jul 1;11(13):1760. doi: 10.3390/plants11131760 (PMC9268918; doi:10.3390/plants11131760)
Supplement: Supplementary file 1 [file plants-11-01760-s001.zip › Supplementary File S3 Genomic sequences.pdf]

>CsCML1
ATGATGAGTGAATCCAATGATTCTTCGGTACATGCATGTTTTTCTTTTACCATGTTTTCAT
GTTTGTTTTTTTAATAATG
TTTTTGTGTTTTGAAACATGTTTTTTTGTTGTGTTTAATGATGATGAACAGAGATGGAGT
ACATTAACATTGGAAGAAAA
AGTGTGTGGTGCATTAGTCCCATTATTGGCTTTTGCTGAGATTATTTTGTTTGCTTTTTCT
GGCTGCTTTGATTTTGGTG
GTGCTAAGAAGCAAAATAAGCACAAGCTGCTTCCTTTTGATCATCATCAGCTTACAAG
ACTTGCTTCTGGATCTAATTGT
AAGATTGCATCACTAGTATTTGCTTATTTGTTTATATGTTTTGAATAAGCACTTTTTGAGA
TCATATGTTTAGAGTTATA
ACCTGTTTTCTGCTTGTTTGTTAAATAAGCACTTTTTGAAACCTAATGTTTAGAATAAGC
TGTTTTCTGCTTATTTGTCA
AATGTTTGGTGTTTTTTGAATAAGCACTTTTTGAAATGAAATGTTTTAGAATTATAACCT
GTTTTTTTGCTTATTTGTTG
AATGTTGGGTGTTTAGAATAAGCACTTTTGAGGGTTTAGCTGTTTTGAATAAGCTATTTG
GAAACTGATGTTCTTAAGAT
TGAATTTTGGAAATTGGTGTTGAATAAGCACTTTTTGAAATCAGATGTTTAGAATTATAA
CCTGTTTTTTGCTTATTTGT
TGAATGTTGGGTGTTTAGAATAAGCACTTTTGAGGGTTTAGCTGTTTTGAATAAGCAGT
TTGGAAAGGTTAATTGCCTGA
AAATGTATCTAACTTACCATTTTTTATGGTTCCGTTTAGTGGACTTTTTTTTTTCCAAGTA
AATAAAAAACTTTTGAATT
CGTGCTTTTCTGTGTATTTAGCTCGTGGAAAATGACCGTTTTACCTCTCGGTATCAATAT
ATTTACCGAGAGGTAAAATG
GTCATAGGATTGCACAGAAAAGTACAAATTCAAAAGTTTTTTATTTAATTGGAAAAAAA
AAAGTCTACTACACGGAACTA
TACAAAATGGTGAGTTAGATACTTTTCAGCCAATTAACCCATTTGGAAATTGGTGTTCTT
AAGATTTAATTTTGGAAATG
GAATACGAAACGGGTAAAGGGATGAGGTTCTTCATTTTCAGGTTACCCGAGGCGGGTG
AGTCTTTGAGTTAGATGTACCC
GGCCTACCCGAGGAATACTCGTGACCCTATGTCAGGCTATTATGTTTTGATAAAGTAGAT
TTGAGTAGTTTTGGATTTCG
ATTATTTCAATGGTTAGTGTGATAAGCTGTTTTGAATAAAGGACTTGAAACTCGTTATTC
TAAACATTTGATTTCAAAGA
AGAATAAACAATGAGCATATAGAAAAATTGTGATAAGCAATTCTAAACACCTAGATAAG
AGGGTGTTTGGGATTGTATAT
TTAAGTGCTTGTTGCGTTTTAAAGAAAGAAAAAGCACTTTCGAGCGGAAGTGTTTAGA
CTTTGGATTTCCTTATGCTTAT
TGGCTTATTTGCTTATAATTTGGTGAAATAAGCAGTTTAAAATTAGCACATGTGTAGATG
ACTATATTAAATTGCTTATA
CTCCGTAATACATAAGCATAAGCTAAATGTACGCAATCCCAAACACCCCCTAAAAGCTA
TAGTTTGACTTAACAGTTTCA
GTAAGCAACTCCGACAAGTATATTTAAAACTACTTATTTCACCCTCATATAAGCAGATCA

TCACAGATGTTAGTTTTTGC
AGTTAATGTAAATGAAGTTGAGGCGCTTTTCGAACTGTTTAAGAGCCTGAGTTCGTCTA
TCATCGACGATGGATTGATAC
ACAAGGTATATTTTTGAGTTCTCATTTAAGTCTGCCATATTACATTGTATATAGTAATTTTA
GCTAGTAAGAGCTTTTGC
ATAAGTTGTTATTATCTTTTCTGAAGGAGGAGCTTCGATTAGCACTATTCAATACCCCTC
AGGGTGAAAATCTGTTTTTG
GATCGGGTAAGAACTGAAATTTAATCTGCATTGTGTTATTTGAATATTTTCGGTTAAATC
CTATATTTTACCATTCCTTA
TAGAAATGCTCTTTTGTTATGTAGGTTTTTGATCTTTTTGACGAGAAGAGAAATGGTGT
AATTGAATTCGAGGAATTTGT
CCATGCTCTCAGTATCTTCCATCCGTATGCACCTATAGAAGATAAAATAAACTGTAAGAT
GTAAAAGCTATCTTTCCGTA
CACAGTTAAATTGTATCTCATGAAAAAACGTACCCTGATGACTTCTATCCTTTTTTCCTC
CTGCAGTTGCTTTTAGGCTC
TATGATCTAAGACAAACGGGGTATATAGAGCGAGAAGAAGTGAGTATGCTGTAATATTA
GTAAAGTTAACAGATTTGAGT
AAATGTAGCAATTTTTGACCCGTTTACTTATGAATGGGTCAATTTGTGTTACGTTATATTT
CAAAAACGTAAAAAGTTAG
TCAAACGGGTTGAAAGTAGCCCAAAGCATAAATTAAGGCATGCAACCCATTAAATAAG
TATATTAATAGCGATACATACC
TTTGGACAAAAAGTGTTATGGTAACCTGACCCAACGCGTTTCAACCCGTACAAATAATT
ACCCTTAACCTGTTTGACCGT
ACATTATTGACTCATTTTGCACTTCATTTTAAATAGGTCAAACAGATGCTAATAGCAATA
TTAACAGAATCCGAGATGAA
CTTATCAGATGACCTTCTTGACGTTATTATTGACAAAGTAAGATCTCTGCAAACTGGTTT
GGTTTTTCCAAATCTTTAAC
AGTAGTAATTAATAGAGGGACGATGCTTGTTGCAGACATTTGCTGATGCGGACACTGAT
GGAGATGGTAAAATCTGTAAA
GAGGAATGGAAAGAGTTTGCACTCAGATACCCGAGTCTTTTAAAGAACATGACTCTTC
CTTATCTAGCGTATGTCACTCT
ACTTCTTTTGAAATTATAACAAGTTACTAGTTACAAAATTTTCAAAACGAAAACGGGCT
AAAATGTTTTTTGATACAACC
CATCCGTCTGATCCATAATTCCTCGGCCCCTCTTTGCTACAAATTACATTGATTAGTTAA
CTGAACTTGTCGTTAATATT
TTGATGAAAATTCAGGGACATCACAACAGCATTTCCAAGTTTTGTTTTTCACACGTCGG
TGGAGGATGCAACATGA
>CsCML2
ATGGGCTGCTTTCATTCTACTGTAAAAAAACCGGTTCCTGGGCATGAAAATCCGACTTT
ACTTGCTTCTCAAACAGCCTG
TAAGTTAATCATTGTTGCAATGTTTTATCCAACATGATTGGTACATGTTGCTTATGTTGGT
CTTTTTTTATGCTCTTATG
TCTCTCCAGTTAGTGTAAGTGAAGTTGAAGCTCTATTTGAGCTATTCAAGAGCATAAGT
AGTTCTGTAATCGATGATGGG

TTGATTAATAAGGTAATCAATTTGTTTGTTGATGTTAAGTTATCCTCAATGTCTAGAAAC
AAATTATGCGTGTTTATCTG
TTTGGTAGTTAGGAAATTTCTGTCCGTTGCAACATTTATTGTTTAATGAATCAAACTCTC
TTTAAGAAACTTGGTGATTG
TGGGTCACAAGTTTCTAATGTTCTCTTCGCTAAAGGGAAATTTAATTTTTTACCTGAACT
ATGTATCGGCTCAATTATTG
TGTCTTTCATAAAACACGAGTATTAATTGTCTCCTTGCACTTCATTTTTTTCTTCGTTTAT
CATTGGTTTTATTATATTG
GTATCAGATCCAGTTATGATATCTGTGATTGCCTTGGATGTATCCCTGATTTTCTCTTGAA
CAGGAAGAGTTCCAACTAG
CATTATTCAAGAACAGAAAGAAAGAGAATCTCTTTGCAAACAGGGTATGATGCTTCTT
CAGTTTATTTTAAGTCACGTAA
AGTTCGTTCTCTTTGTATTTTTTCTTTTGGGCATTGTCAGTATGTAGCTAATCTGAAAGAT
TGATTGTGTTATGTTGAAA
TTTACTCTGCTGGTCTTGGACTACAAGAAATTGATTTTAGCAAATTTTTGTTATTGAGAA
ATTTTCATGATAAGTATCAC
TAGTCGGCAGTTTGGTTAGAATGATGGTTGGAACCTGCATGTTAATTTATTATACTTGTC
GCTAGTATTTATTTTACTTG
TGACATGTCACGTACACCTTCTTTTCTTTTAGGAGTTTGCTACATTAAATATGCATATATT
GATAAGAATAATTAGGTAA
GAACCTGTAGGAGCCTAATAGGGTATAACATATCTGGTAAACTATAAGATTTAGTATTAA
ACCCTAATAATTATTGAGAT
TGTAACCTGCATCTCAATCATATTTCATCATTTGAAGTTACCTTTTCAAGAAGGTGAGTG
ATGTAACTGATCGGGTTGCG
CACATATAGCTTTGCGACTTTAAAAAAAAAAGGCACGTCAATAATTGAATGAGTGTGC
GTCTTTGTGCTTTAGGAGTATC
ATTTTGTAATGAACTAGTAATAACTCTGTAACATAATATATGTTAAGATTTAAGAATATAC
TAATACTTTCAGATATGGC
TATGACTGATCTTTACATTAACACTTCAGATTCGATACTGGTCCAGGCATATATATACACT
TTCTGAAATTATTTTACTA
CCTTTGTTGCAGTTTAAACTACATAAGCTGCTTCATTTTGGAGTTCATACTTTAATAATTT
CAGTTTTTTGCATTTTGGG
GATTACATATCCATGTTATTTCAGAGCAAGTGCATGTGAACCATGACTCATTGTATCTCC
TCACCATTCAATTCAAACCC
AACAGATGAAAATGATCTACAACTAAAATTTTGTTAATATTTTCATCTATTCCCTTTTCTG
GCACATAATCATAAATTTA
TAATAACTTAAATACCTTACATCTCATGAAGTTAGCCTTTTTTTTAGTGCTCACATAATAA
CTAAAACGCCCTACCTTTT
TCATCATATACCGTTTTTATACATAATCTTCACATAAATGAATTTGAGCACCTAATCTTAG
ATTTTGTTGAAACAGACCA
TCTGATATCATTAAGGAGTTTAGGTGCTAAAAATGCTGTGCAAGGCGCGGAAATGATAA
ATGCACTCGTATTAGATTTCG
GATTTGGTGTGCTGTTTTTATACTTCGTTACTGCTGTTTGCAATGCACTCGTATATGTGC
ATTGATATGAAATGTCAATT

GCATGCATATTGGATAGAGTACAAGATACATTTTGAAAAACATTGGTATCCTATCTGTTA
TACAGTTTTCTTAAGCTTGT
AAGTTTCATATGCAGATATTTGATCTTTTCGACGTCAAGCAGAAGGGGGTTATTGATTTT
GGTGACTTTGTTAGAGCACT
TAACGTTTTCCATCCTAATGCTCCTCAAGAAGACAAGATCAGTTGTAAGTTGATCATGA
TGACCCAAACTGTAAATTTCA
AGTGGCCATGAATGGTCTTGTAATACTCATTGTTCGTGTTTCAGTTTGTTTCAAGCTTTA
TGATATGGACGGAACAGGAT
TCATAGAGCGCCAGGAGGTACATTTCTGCCATTTTAATGCTCTATGCTTTTCCATGTTTG
TGTCCAAATATTATTACTAA
AATATAATAAGAATTGTATTAACAAGCTCGTATAAAGGAAAATAACCAAGCCACCTGAA
TATCGTTTTTCGTTTTCTAAA
GCTATTATGGCACGTGTGTAATTATTTGTCATAAAGAAGTTTGAAATATTTACTAAAGAT
AATAAGAATCGTATTAACAA
GCTCCTTCATATAAAGGAAATAACCAAGCCCCTACATAAACATTGTGTTTTGTTTTCTAA
AGCTATTATGGCACATGTGT
AATTATTTGTCATAACATCTTCATTATGTGTTATCTTTTGTAGGTTAAGCAAATGTTAATC
GCACTTTTATGTGAGTCGG
AGCTAAAGCTAGCTGATGACACCATTGAGACAATACTTGATAAGGTGGGTTCAATTTTA
TTTATTATGACCATTTTGAAA
CCGACACCAGCACCTAACGATATAAATAAGTTTCATTGAGAAAAAAATGAACTCTTTTA
GTTTGGCCCGCGTACTATTGA
ATAAGATTTTTCTTTCACACTACACCTTTCTGCCCGTAAGGTCCTACTATTCGGTCAAAA
AGAAACCAAATTGACCATTT
TAAACTGTCTGTTATCATTTAAAGACAAAAACTATGTCGTCACGTTAACTTTTTGGCAG
ACAAGAGAACATGAAAAAAGT
GATTAGAACATCATGTTCAGTTGCTGGATGAATAACTTTTATCGTACAGTAGGTGTTGGC
ATAAGGGTAGACAAACTAAT
ATTTCATTAAAGCATGATTCATGTTTCCATTTTTGTTTGAGCAGACGTTTTCAGAAGCCG
ATGTAGACCAGGATGGGAAG
ATAGACAAATCAGAATGGCACAATTTTGTCACCCACAACCCTTCCTTGCTCAAAATAAT
GACTCTTCCATATCTAAGGTA
TCTAGTTCTACAGTTACGTTCTTCTTTGTGCGTACCCACTTTAGAGGAATACCTCATTAC
CTGTACTGAAAATAGAACCT
TTCCACACATATTCAATGGGCGGGTTGGGTAATGAGTTACATTGGGTTCTAGCATATACA
AAGGTACTTTGAATAAAATG
ATTCAAGAGGTATGTATCAAAACCAAATTTCCACTGTACCCCTTTTAGCTCAATTAATGA
ATTATAATTTTGACCCATTT
GAATCCTTTAAGATAAAACTGAACCCAAATCACATCCAATTTACTTATCAAATTTATGCG
AAATGCAGGGATATTACAAC
CACCTTCCCAAGTTTTGTGTTCAATTCTGAAGTTGAAGAAATTGCAACATAA
>CsCML3
ATGGGCTGCTTTCATTCTACTGTAAAAAAACCGGTTCCTGGGCATGAAAATCCGACTTT
ACTTGCTTCTCAAACAGCCTG

TAAGTTAATCATTGTTGCAATGTTTTATCCAACGTGATTGGTACGTGTTGCTTATGTTGG
TCTTTCTTAATTGTCTCTCC
AGTTAGTGTAAGTGAAGTTGAAGCTCTATTTGAGCTATTCAAGAGCATAAGTAGTTCTG
TAATCGATGATGGGTTGATTA
ATAAGGTAATCAATTTGTTCGTTGATGTTAAGTTATCCTCAATGTCTAAAAACAAATTAT
GCGGTCAATCTGTTTGTCTG
TGTAGATAGGAAATTTATGTGGCATCTTTTCCGTTGCAACATTTATCGTTTAATGAATCA
AACTCTCTTTAAGAAACTTG
GGTTCTAATGTTCTTTTCGCTAAAGGGAAATTTAATTTTTTACCTGAACTATCGGTTCAA
TTATTGTGTCTTTCATAAAA
CACGAAGTATTAATTTGTCTCCTTACACTTCATTTTTTTCTTCGTTCATCATTGTTTTATTA
TGTTAGTATCAGATCCAG
TTATGATATCTGTGATTGCCTTTGATGTATCCCTGATTTTCTCTTGAACAGGAAGAGTTC
CAACTAGCGTTATTCAAGAA
CAGAAAGAAAGAGAATCTCTTTGCAAACAGGGTATGATGCTTCTTCAGTTTATTTTAAG
TCACGTATAGTTCTCTTTGTG
CATTATTTCTTTTGGGCATTGTCAGTATGTAGCTAATCTGAAAGATTGATTGTGTTATGTT
GAAAATTTACTCTGCTGGT
CTAGGACTACAAGAAATTGATTTTACTAAGTTTTCGTTGTTGAGAAATTTTCATGATAAG
TATCAGTAGTCGGCAGTTCG
GCTAGAAGATGGTTGGAACCTGCATGTTAATTTAGTAATTATTTTACTTGCTGACATGTC
ACGTAACACCTTCTTTTCTT
TAGGAGTTTGCTACATTAAATATGCATATATTGATAAGAATAATTAGGTAAGAACCTGTAT
GAGCCTAATAGGGTATAAC
ATATCTGGTAAACTATAAGATTTAGTATTAAAACCTAATAATTATTGAGATTGTAACCTGC
ATCTCAATCATATTTCATC
ATTCGAAATTACTCTTTCAAGAAGGTGAGTGATGTAACTGATCGGGTTGCGCACATATA
GCTTTGCGACTTAAAAAAAAA
AGGCACGTCAATAATCGAATGAGTGTGCGTCTTTGTGCTTTAGGAGTATCATTTTGTAAT
GGACTAGTAATAACTCTGTA
ACATAATATATGTTAAGATTTAAGAATATACTAGGATACTTTCAGATATGGCTATGACTGA
TCTTTATATTAACACTTCA
GATTCGATACTGGTCCAGGCATATATATACACTTTTTGAAATTATTTTACTACCTTTGTTG
CAGTTTAAACTACATAAGC
TGCTTCATTTTGGAGTTCATACTTTAATAACTTCAGTTTTTTGCATTTTGGGGGTTACATA
TCCATGTTATTTCAGAGCA
AGTGCACGTGAACGATGACGCATTGTATCTCCTCACCATTCAATTCAAACCCAACAGAT
GAAAATGATCTACAACTAAAA
TTTGTTAATATTTTCATCTATTCCCTTTTCTGGCACATAATCATAAATTTATAATAACTTAA
ATACCTTACATCTCATGA
AGTTAGCCTTTTTTTTTAGTGCTTACATAATAACTAAAACGCCCTACCTTTTTCATCATAT
ACCGTTTTTATACATAATC
TTCACATAAATGAATTTGAGCACCTAATCTTAGATTTTGTTGAAACAGACCATCTAATAT
CATTAAGGAGTTTAGGTGCT

AAAAATGTTGTGCAAGGCGGCTGAAATGATAAATGCACTCGTATTAGATTTCGGATTTG
GTGGTGCTGTTTTTATACTTC
GTTACTGCTGTTCAAGAGAGTTTGTAAGTTTTCTTAAGCTTGTAAGTTTCATATGCAGAT
ATTTGATCTTTTCGACGTCA
AGCAGAAGGGGGTTATTGATTTTGGTGACTTTGTTAGAGCACTTAACGTTTTCCATCCT
AATGCTCCTCAAGAAGACAAG
ATTAGTTGTAAGTTGATCATGATGACCCAAACTGTAAATTTCAAGTGGCCATGAATGGT
CTTGTAATACTCATTGTTCGT
GTTTCAGTTTGTTTCAAGCTTTATGATATGGACGGAACAGGATTCATAGAGCGCCAGGA
GGTACATTTCTGCCATTTTAA
TGCTCTATGCTTTTCCATGTTTGTGTCCAAATATTATAGATTCTTAATAATTCCTTTCGATA
AAGAAGATTGTAATATTT
ACTAAATATAGTAAGAATTGTATTAACAAGCTCGTATAAAGGAAAAAAAAAGCCACCT
GAATATCGTTTTTCGTTTTCTA
AAGCTATTATGGCACGTGTGTAATTATTTGTCATAAAGAAGATTGAAATATTTACTAAAG
ATAATAAGAATCGTATTAAC
AAGCTCCTTCGTATAAAGGAAATAACCAAGCCCCTACATAAATATTGTGTTTTGTTTTCT
AAAGCTATTATGGCTCGTGT
GTAATTATTTGTCATAACATCTTCATTATGTGTATCGTTTGTAGGTTAAGCAAATGCTAAT
CGCACTTTTATGTGAGTCG
GAGCTAAAGCTAGCTGATGACACCATTGAGACAATACTTGATAAGGTGGGTTCAATTTT
ATTTATTATGACCATTTTGAA
ACCGACACCAGCACCTAACGATATAAATAAGTTTCATGAGAAAAAAATGAACTCTTTTA
ATTTGGCCCGTGTACTATTGA
ATAAGATTTTTCATTCACACTACACCTTTCTGCCTGTAAGGTCCCTACTATTTGGTCAAA
AAGAAACCAAATTGACCATT
TTAAAATGTCTGTTATCATTTAAAAACAAAAACTATGTGGTCATGTTAACTTTTTGACAG
ACAAGAGAACATGAAAAAAG
TGATTAGAACATCATGTTCAGTTGCTGGATGAATAACTTTTCTCGTACAGTAGGTGTTG
GCATAAGGGTAGACAAACTAA
TTTTTCATTAAAGCATGATTCATGTCTCCATTTTTGTTTGAGCAGACGTTTTCAGAAGCC
GATGTAGATCAGGATGGGAA
GATAGACAAATCAGAATGGCACAATTTTGTCACCCACAACCCTTCCTTGCTCAAGATAA
TGACTCTTCCATATCTAAGGT
ATCTAGCTCTACAGTTACGTTCTTCCTTGTGCGACTGTGCGTACACACTATAGAGGAAT
ACCTCATTACCTGTACTGAAA
ATAGAACCTTTCCACACATATTCAATGGGCGGGTTGGGTAATGAGTTACATTGGGTTCT
AGCATATACAAAGGTATTTTG
AATAAAATGATTCAAGAGGTATCTATCAAAACTAAATTTCCACTGTACCCCTTTTAGCTC
AATTAATGAATTATAATTTT
GACCCATTTGAATCCTTTAAGATAAAACTGAACCCAAATCACATCCAATTTACTTATCAA
ATTTATGCGAAATGCAGGGA
TATTACAACCACCTTCCCAAGTTTTGTGTTCAATTCTGAAGTTGAAGAAATTGCAACAT
AA

>CsCML4
ATGATAGAGAAGATAGATGTGAACAATGATGGATGTGTTGACATTGATGAATTTGGTGA
GTTGTATAAAAGTATCATGGA
TGATCGTGAGAATGAGGAGGATATGATGGAGGCTTTTAATGTATTTGATATAAATGGAG
ACGGGTTTATCGCGGTTGAGG
AGCTTAGAGCGGTTTTGGAGTCTTTGGGGCTGAAGCAAGGCCGAAAAGCCGAAGATT
GTAGGAAAATGATCATGAAAGTT
GATGTGGATGGTGATGGTATGGTTAGTTTTGATGAGTTTAAGGAAATGATGAGATCAGG
TGGCTTTGCTGCTATGGCTCA
AAATTGA
>CsCML5
ATGTTTGACAAAAATGGCGATGGAAGGATCACAAAGCAAGAACTAAATGATTCCTTAG
AGAACATGAACATTTACATATG
TGACAATGATCTTGTACACATGATAGAGAAGATAGATGTGAACAATGATGGATGTGTTG
ACATTGATGAATTTGGTGAGT
TGTATAAAAGTATCATGGATGATCGTGAGAATGAGGAGGATATGATGGAGGCTTTTAAT
GTATTTGATATAAATGGAGAC
GGGTTTATCGCGGTTGAGGAGCTTAGAGCGGTTTTGGAGTCTTTGGGGTTGAAGCAAG
GCCGAAAAGCCGAAGATTGTAG
GAAAATGATCATGAAAGTTGATGTGGATGGTGATGGTATGGTTAGTTTTGATGAGTTTA
AGGAAATGATGAGATCAGGTG
GCTTTGCTGCTATGGCTCAAAATTGA
>CsCaM1
ATGGCAGATCAGCTCACCGATGATCAGATCTCTGAATTCAAAGAAGCTTTTAGCCTATT
TGATAAAGATGGCGATGGTTA
GTTCCTTCATATTTCTTATAAAAAAATCCCTTTTTTTATTATTTGTTATATGACATGTAATG
ATATGTTTTAATGAATGT
GTTATAACCCATGGATTGATTTATTTGATTCTTTTTATTGTATTTGGTTATGTTATGTTGTT
AGATCTGGTTTATTTGGT
TGTTTGACTTTTTTTTATAACATTTTTTGTGTATATGTGATGTGGGTCTAATTATAGATCA
ATTTTGGATCTGTTTTTAT
TTGTAATTATGTTGTGTAATATAGGAGAATGTTTTCTATGTATGTTTTTGTGTCAGATCTG
TTTGGGGAATTATGGATGA
TTGTTTTAGGACATTATGTGTCTGGAGATAGGATAGATCAATATAGTAATATATGGTTTTG
GATGGTTTTTGCACATTTT
GGTGCATAATTATGTTTGGGCGAATCATAACTTTTTTGGGGTTGGGACGTCAAGAATAG
AATAAAAGCAGTGGCGTCTCG
AGGGGAAATGAGCTTGTCCAACATGTTATAAGTGGGGAGTATATGACAAATTTACAACT
TGTCCCACAATAGGTATTCGA
TAAGATGTAAAGGGTGTTTGGGATTGCTTAATAAAGCTGTTCATCTGTTTATTGCGTTTT
GATACTGTGAAAAAGCCTTT
TATTAGTATGCTACATTATTTATTAGTATGCGGCAAGGTTCATGGTTTTGTAGATGGAATG
CAGATAATGAGTTTTAGTA
ATTTGGAAAACAAAGCTATAATATGCACTTCGAAAGTTGCTTGTTTCCATTTGTTGTGAT

AATGTGATTATTTACGCGTG
TATCAGGTTGTATCACAACCAAGGAACTCGGAACAGTTATGAGGTCTCTAGGACAAAA
CCCAACCGAGGCTGAGCTTCAA
GATATGATCAACGAAGTCGATGCTGATGGCAACGGTACTATTGATTTCCCTGAGTTTCT
CAACTTGATGGCCCGCAAGAT
GAAGGACACTGATTCTGAGGAAGAGCTCAAGGAGGCTTTCCGGGTTTTTGACAAGGA
CCAAAATGGTTTCATATCTGCGG
CCGAGCTTCGTCACGTCATGACTAATCTTGGTGAGAAGTTGACAGATGAGGAAGTCGA
TGAGATGATCCGTGAAGCTGAT
GTGGACGGTGATGGCCAGATCAACTATGAGGAATTCGTCAAGGTCATGATGGCTAAGT
GA
>CsCML6
ATGTGTCCCACAGGAACTCTTATACCCCTAGCAACGAAACAAGCCGAACTACGATCAG
CATTCAACGTCATGGACGTCGA
CCATGATGGCAAAATAAGCCACGACGATCTAAAGCGTTTTTACGCAGACTACGCGGTA
AATGACAATGAAATTATCATAG
GAACAATGATGAAGGAGACGGATTTGAATAAAGACGGGTACGTTGAATACGAAGAGTT
TGAAAAAATGTTGTTTCTTAGG
AGAAGTAGTAATGTGATGGAAGAAGTGTTTAATGAAATAGATAAAGTTGGTGATGGTA
AACTAGGGTTTAGTGATTTACG
AAGTTATCTTGACATGGCTGGGTTAATGTTAATGATGATGATGATGATGATGATATTAAG
GCTATGCTATTGTTAGCTAA
TGATGATGAGAATGATGGTGTTACTTTTGAAGGGTTTCTTAAGATATTAGCCATTTGA
>CsCML7
ATGAGTAAGTCCATTTTTCCGAGTTTCGTTTACATTTTTAGTACTTTCTACTTGATGATAT
TAGTTTTTCATTCCTTTTA
TCGTATGTGTTATCTTATGCAAGTTTAGATTCTTAACATAGAGGTTCTCATCAGCTTACA
GGAGCGTATCTGATTATTAC
GTTTGAAAAACACAGATAGTACGAAAATAGTGTTAGGCAAACTGTTTAGAAAAGGTGA
TTTTCTGCGTTTTCCTGGAGAG
AAAACACAGTTTTGAAAAAGTAGCTTGACATATGCTTTTTGAAAAGACTGTTTTTAAC
GCATAATCAGTTTTGTAAACGC
AATCCAATTGTTGATTAAAAGCCGTGCCCTTGTCTGCTTTATAATAGAACATCGATTTTA
TTACAGATTTTATCACAGGG
ATAACATGTTTGATATTTTATGAAGATACGTCCCGTCCCTGTCCTTCTCTCAAGTCATTAT
TCTACGAAGTTGGAGGAAT
GCTCCGATGTTGCAATTCCACAAATGAGTACGAGAGGCTGGACAGTGAGCTCGAGAG
GAAAATGATAGAGGTCAAGAAAA
GATATGTACCAGGAAATAGCAGCATCAGGTCCATCAACAGCATTATTCTGAAGTTTCCT
CAGTTCAGACAAGGATTGGAA
GAGATTAGAGGCGTTTTTCGACAGTTTGGTCAGTTTAACAAGAAATGGTGTAAAATAAT
ATACCAACGGTTTTATCAGAT
TCCCGTTTGTCTGATTCTCATTTCTTATGCAGATGTTGATTCAAACGGAACCATCGACCG
TGAGGAGTTAAGAAAATGCT

TACACAAATTCCAATTCGACTGCACAGAGGACGAAATTAATGACCTTTTTGAGTCTTGT
AACTTGGGCAGGAATCAAGGC
ATGAAATTTAACGAGTTTATCGTTGTCTTATGCCTTATCTATCTCCTTACATGTACCTCCT
CTTCAAACCATACTGTAAG
CACAATGTTCTCAAATCTTTTGACAGTTCTCGAGCACAATCTAATAAATGGGCTACCTG
TTTTTAGGCGACAACAATGGG
ATCACCAGAGCTCAAATCAACATTTGATACCATAATTGAAGCGTTCTTATTTCTTGATAA
AAATGGTGATGGGAAACTAG
ACAAGAAGGATATGACTAAAGCTATGAACGATGATTTTCCTAAGGAGAAATCTCCGAAT
CACATCACCATGTCACGATTC
AGTAATACTTTTGGACTTAACATAAGTTCTTATAAGCTAAACCTTTTTTGTACAAGCAAA
AAACAGTCAATTAATTGTCA
AATCATTTTTCTTTAGTATGTAAATTGTGCATAAACGCAGGAGAAATGGACTGGAACAA
GGATGGGAAAGTCGGCTTCAG
GGAGTTCCTATTCTCCCTGATCAATTGGGTTGGGATCGACTCCAATAATGAAGTTCATGT
CGAAGTAATCCGAGAACAGA
AACCGTGA
>CsCaM2
ATGGCCGATCAACTCACCGATGATCAGATCTCTGAGTTCAAGGAAGCTTTCAGTCTATT
CGATAAAGATGGAGATGGTCA
GCTTTTCTCTCTTCATATTCATTTTCATCTATATCATGGTTGTTGTTTACATAATAGGATTG
ATATTCATTTTTGAGATC
TGGGTTTTCATTTTTGATGTTTGACTTTATGGATCACTGTGTTTTCTTGGTTAATGTCTGT
CAATATTGATTTTGAGGTT
AACTTGTTTTTTTTTTTTTTTTTTTTATATATATATATCTGTTCATTGTCAGATCCGCTTAG
CTGCAATTTTTAGTATT
CGTTTGTAAGGTTTGTAGATTTATATGTGGTATGTGCTTAAGATCTAAAGTAAACTAAGT
TTTTGTAAATGTTTTTATGC
TTTTTTAGGAGGTGTTTGGGATTGGTTATAAGGGGGTGTTTTGAATTTGCTTTTTTCAAA
CTGCTTTTTGTTGCTTTTAA
ATAAGCGAATATACAGTTTGACTCGAGCACTGTAAACTGCTCTTTTAAAAAAAAAAAA
AAAAAAAAAAAAAAGCAAATATG
TACAAATCATCAATCTATAGGAACCCTTTAAGTGCATATCTACTTATCGAAATTTGAAAT
AAGCAGTTGGGTTAAGTATC
TTTAAACTAAGTTTTTCAAAATTGCTTATTTTACTCATGAAGGGTAAGTTAAGCAGATTG
AATAAGAATATTAACAAACA
TCTTTGACCTTATAAACTGGTTATATGATGTACTCAAACATATTGTCATGGAAGAAGTTA
TGTGTATGTTGTTACAGGTT
GTATCACTACCAAGGAGCTTGGAACAGTTATGAGGTCTCTTGGACAAAACCCCACGGA
GGCTGAACTCCAAGACATGATC
AATGAGGTCGATGCTGATGGAAACGGTACTATTGATTTCCCTGAATTTCTTAATCTGATG
GCCAGGAAGATGAAGGACAC
TGACTCTGAGGAGGAGCTTAAGGAGGCTTTCCGTGTTTTCGACAAGGACCAAAATGG
CTTCATTTCTGCAGCTGAGCTAC

GTCATGTTATGACAAATCTTGGTGAGAAGCTGACTGATGAGGAAGTTGATGAGATGAT
CCGTGAAGCTGATGTGGATGGT
GATGGTCAAATCAACTATGAGGAATTCGTCAAGATCATGATGGCCAAGTGA
>CsCML8
ATGAAGTTTTTCTCAACATCTAATGTTTCCATCCCGTTTTCGCTTGAGCGCTCCGAGATT
TTGGTCGTTAAGCATGATTC
CAACTTGTCAAAAGCACACGTTTCCTTCACCAACAAGAACATTGTGGACGATGCATGT
GTCCAAAGAGACGACGTGGAGC
TAATAATGGCGAATTTAGGTGTTTTTTGCCATCCTGAAGGCGAGAAGGTGCCCGAGGT
GATGACTTCTAATGACTTGTTT
AATATTTTTGAAGATGAGCAACCAAGGTTGGATGAAGTGAAACAAGCTTTTGATGTGT
TTGATCAAAACAAAGATGGGTT
TATTGATGCAAGTGAGTTGCAACGAGTTCTTGTTGTTTTGGGCTTGAAGGAAAGATCG
AATATCGAAGATTGTAGGAAGA
TGATCCGAGCGTTTGATGAGAACGCCGATGGTAGGATAGATTTTGATGAGTTTGTTAAG
TTTATGGAAGCTACCTTTTGT
TGA
>CsCML9
ATGTTCCAGCCAGACGTCGATGAAATGAGACGAGTTTTCAACAAATTTGACAAGAACA
AGGACGGTAAGATATCCAAGGA
AGAGTATGGTTCGGCCGTTGGAGTACTTGGTAGCAAAAACACTAAATCAGACGTGATC
AAGACGTTTCAAGCCATAGACA
CTGACGGGGACGGGTTTGTGGACTTTAATGAGTTCATGGAGGCTCAAAAGTCGGAAG
GTGGTGTTAAGACAGCGGATATA
AAGAGTGCGTTTAAGGTTTTTGATTTGGATGGCAATGGGAGAATAACAGCAGAGGAGC
TTGTTCAAGTGCTGAGGCAGTT
AGGAGAGAGGTGCAGCTTGGAGTCTTGCCGAAAAATGATTAAAGGGGTCGATGCAGA
TGGGGATGGAATGATCGACGTTG
ATGAATTCATGGGTCTAATGACCCGTAACATGAAACTGGCATAA
>CsCML10
ATGGCAGATGCTGATCATCAAGCGAACCTTGAACGAATCTTCAAGAAATTTGATACCA
ATGGAGATGGCAAGATCTCATC
ATCAGAGCTTGGAGAAGCTTTGAAGACGCTCGGCTCTGTGTCACCTGAAGAAGTGCA
ACGTATGATGCGTGAAATCGATA
CTGATGGAGATGGATTTATTTCTTATCAAGAATATATAGATTTTTGTAATGCTAACAAGG
GGTTAATGAAGGACGTTTCC
AAGATCGTATAA
>CsCML11
ATGGCTGATGAAGACAAGGCAGAATGTGATCGCATCTTTGGCGCATTTGATAAAAATG
GAGATGGTAAGATCTCTGCAGC
TGAGCTTGGAGAATCTTTGACGAAGCTCGGCTCTGTGTCACCTGAAGAGGTCCAAACT
ATGATGGATGAACTTGATACCG
ATGGAGATGGGTACATTTCTTATGATGAGTTCGCTGAATTTTTTAACGCAAACCGGGGC
TTAATGAAGGACGTTGGCAAA

ATCTTCTAAGAATTGACAATACTGATATCTTTATTTTTGGCAATACCTTTCTCAATTGATA
AGTTTCAGTTGCTTTATAT
CTTTGTTTATTATGTTTGTGTTAACAGTTAACGGTGACATATTTGCTTAAGATATATAAAA
TTACAAGCAATTATTTGCA
ATAGAGAAGATTGTTCAATCTAGTAATCTACAATCATATCCGTAAGTGTATTTAACTACT
CCGTATTTACTATTTTTTTT
AACATGTATGTTGCAAAATGAACATGTAAAAAGGAAAATCAATTACCGGAACAAACAC
TTCTCACTTAGGGGGTGTTTGG
GATTGCTTATTAAAAATAGATTATGCGTTTTGAAAACGGATTATCAGATAATCAGTTGTT
ACAAAACGTGATTTTAACAT
ATAGTGTTTGGACATGACAAAGTAGATTATTTCATAACTTATTAGTAAAATGACCAAAGT
AGCCACACAAAAAATAAAGA
AAAGTGAAATAGGAGGAGTGACTTTGTAATATTGCCCTAAAAAGTGAATTGCGTTTCCC
AATTGGATGACTCTTCCTACT
GCCACAAAACGTGGTTTTGAAGCAAGTCAAACGCAGAAAAGCAATTATCACTTTATGT
TTCCCAAACACTTAAAATGGAT
TATTTGCGTTTGTGAAACGCAATAATCAAATAATTTAAACCAAAACGCAATGCCAAACA
CCCTCTTAATTACAAAAATAT
TCAAAAAAAGTTACTAAAATCGACACTTTGGCCGAGCAAACCATTTGCTCTCTAATTCA
TTTCGCCCACACATCCTTGCT
ACAGGATAAAGAACATGGAAAGCAAATGTTATGCTCTCTCTCCCAATTGCTCTCCGGAT
CGCTACAAGACAAAGAACATG
AAGAGCAAATGCTTGCTCGCCCTATGTTTGCTCACCATTTTCAACGACAAGACAAAGG
ACATGAAGAACAAATGCTTGCT
CGCCTAAATTGTTGATTTTGGTAAAACGGGTTTAGAAGGTTCATTTTAGTAAAGGTGAA
GATAAAGTGTGTATTAAAAAA
AATCTCTTAATTCAAATGCAACTGGTACGTGCAGTAACGAGCAAAGTTTTTTGGACCGT
GAACTTCCGAATGTGAGTTTC
TATTCCCTCTTACAACGTTACAGAGTTCGAGATAAAGCTTCGTAGCTTGTGGGGTTATG
GATAGCAATTTTGACACGGTA
ACTTATGAACGGATCAATACATGTAATTCTTTAAATAGGTCAAATGCGTAAAATGAAATA
GTTTGTTTAAAAGGATATGA
GGCAAACATGTTAACCTTTTCGAAAAATGCTCAATCTCTATTTTTTTCATATTCGTTATAA
TCATATTTGACACCCTTTA
TTATCAACTTTGGACAAAAATCCACTTTCGGGGGTCTGATGACGTATAGCACGTATAAC
AGGCCTATTATACGCATTTCG
GGTCCAGGCCCAACAACAAAACAACCACAAGCACCAGACTGCAAGTAAGCCCAATAA
CAAATTGGTTACGGTTCGGACTC
AAAGACGCACGCAGGGTACTTAGCGGACCAGACCCATGGCCGCGGAAGGTGTTCTGT
GTCCGCGTCCACGACTACTGCGG
AACCTTGCATGACAAAGGTCCCACTTCGGGCACTAACTTTTATAACCGAAATTTTAAGA
AACCCTAGCATTAGCCTCGGC
TATAAAAGGAACTCTAATCCTATCATAAGGTACAACTTTCTCTCACATAGTAATACACAC
ACTTTTATTCTCTGACAAGA

ATGTATATGTACTCTCACGCCGGAGCTTAATCATTGGGTCCGTCCCCACGGTAAGGCTA
ACGGTTCGTTTTCATTTGTGC
AGGGTTTCAATCCATCACTTGGTTTAG
>CsCML12
ATGTCTATAATGATAGCTGAATTTATCCAATACTTGTTCTCCCATGTGTTTCTCAACATGA
TCATCTACCCACAACTCGG
GTATTTCTTGGATGACTCCAAGATTCATGTTGAGAAGAGGAACAAAGATTCTAGATTGC
CGAAAAGGTTGCCTTCATTTA
AAGACGGAAGTGTACGAGGAGACGAAGTGCAGACGGTTATGGGGAACCTTGGAATCT
TTTGCAATTCTAAAGGGGAAAGC
TTTCCAGAGAGATTAAGTTCTAACGATCTTTTTAACATGTTTGAAGAAGAGCATCCGGA
ATTGGATGAAGTGAAGGGAGC
TTTTGACGTGTTTGATGAGAATAAAGATGGGTTTATTGATGCAAAAGAGTTGCAGAGA
GTTTTATCTGCTTTAGGATTGA
AGGATAGAGCAGCTATGGATGACTGCAAGAAGATGATCAGAGTGTTTGATGAAAACGA
TGATGGTAGAATAGATTTTGAT
GAGTTTGTCAAGTTCATGGAAGGCACATTCTGTTGA
>CsCML13
ATGGATAAAGAACAACAATACAAACGTGTGTTTGGACACTTGGATGCAAATGGTGACG
GAAAGCTATCTCCACCAGAGCT
CCAAATTTGTCTTGGAAAGATTGGAGGAGAGTTGTCATTGGAGGAAGCTGAGATCGCG
GCTGCTTTGATGGATTCGGATG
GGGACGGGTTGTTGAGCATGGAGGACTTGGTGAATGTGGTTGAAAGTGCAAACGAAG
AGGAAAAGATTGATGATTTGAAG
ATGGCTTTTAAGATGTACGAAGAAAAGGAAGGATGTATAACTCCGAAAAGCTTGCGAA
GAATGCTTAGCAAACTGGGAGA
GTCGAGAACCGTCAATGATTGCAAGGTGATGATTAATAAGTTTGATGTTAATGGTGATG
GTGTCCTTAACTTCGATGAAT
TCAGGATAATGATGGCATGA
>CsCML14
ATGGATAAAGAACAACAATACAAACGTGTGTTTGGACAATTGGATGCAAATGGTGACG
GAAAGCTATCTCCACCAGAGCT
TCAAAGTTGTCTTGGAAAAATTGGAGGAGAGCTGTCATTGGAGGAGGCTGAGATCGC
GGCTGCTTTGATGGATTCGGATG
GAGACGGGTTGTTGAGCATGGAGGACTTGGTGAATGTGGTTGAAAGCGCAAACGAAG
AGGAAAGGATTAATGATTTGAAG
ATGGCTTTTAAGATGTACGAGGAAAAGGAAGGATGTATAACTCCGAAAAGCTTGCGAA
GAATGCTTAGCAAATTGGGAGA
GTCGAGAACCGTCAATGATTGCAAGGTGATGATTGCTAGGTTTGATGTTAATGGTGATG
GTGTCCTTAACTTCGACGAGT
TCAGGATAATGATGGCATGA
>CsCML15
ATGGATAAAGAGCAACAATACAAACGTGTGTTTGGACACTTGGATGAAAATGGTGACG
GAAAGCTATCTCCACAAGAGCT

CCAAATTTGTCTTGGAAAGATTGGAGGAGAGCTGTCATTGGAGGAGGCTGAGATCGCT
GCTGCTTTGATGGATTCGGATG
GAGACGGGTTGTTAAGCATGGAGGACTTGGTGAACCTGGTTGAAAGCGCTAACGAGG
AGGAAAGGATTAATGATCTGAAG
ATGGCTTTTAAGATGTACGAAGAAAAGGAAGGATGTATAACTCCGAAAAGCTTGCGAA
GAATGCTTAGCAAATTGGGAGA
GTCGAGAACCGTCAATGATTGCAAGGTGATGATTGCTAGGTTTGATGTTAATGGCGATG
GTGTCCTTAACTTCGAGGAAT
TCAGATTAATGATGGCATGA
>CsCML16
ATGGATAAAGAACAACAATACAAACGCGTGTTCGGACAATTGGATGCAAATGGTGACG
GAAAGCTGTCTCCATCAGAGCT
CCAAATTTGCCTTGGAAAGATTGGAGGAGAGCTGTCCTTGGAGGAGGCCGAGATCGC
GGCTGCTTGGATGGATTCGGATG
GAGACGGGTTGTTAAGCATGGAGGACTTGGTGAATGTGGTAGAAAGTGCTAACGAGG
AGGAAAGGATTAATGATCTGAAG
ATGGCTTTTAAGATGTACGAAGAAAAGGAAGGATGTATAACTCCGAAAAGCTTGCGAA
GAATGCTTAGTAAATTGGGAGA
GTCGAGAACGGTCAATGATTGTAAGGTGATGATTGCGAGGTTTGATGTTAATGGTGATG
GTGTCCTTAACTTCGACGAAT
TCAAGATAATGATGGCGTGA
>CsCML17
ATGGACAAAGAACAGCAATACAAACGCGTATTTGGGCACCTGGACACAAATGGAGAC
GGGAAGCTATCTCCTCCTGAGCT
CCAAATTTGCTTTGGGAAGATTGGTGGAGAATTGTCATTGGAGGAGGCTGAGATAGCA
GCTGCTTTGATGGATTCGGATG
GAGACGGATTGCTAAGCATGGAGGACTTGGTACAAGCGGTTGAGGGCGCTAACGAAG
AGGAAAAAATTAATGATTTGAAG
ATGGCTTTTAAGATGTATGAAGAAAAGGAAGGGAGTGGATGTATAACTCCGAAAAGCT
TGCGAAGAATGCTTAGCAAGTT
GGGAGAGTCAAGAACCGTCAATGATTGTAAGGTGATGATTGCTAAGTTTGATGTTAATG
GTGATGGTGTCCTCAACTTCC
ACGAATTCAGGGAAATGATGGTGTAA
>CsCML18
ATGGACAAAGAACAACAATACAAAAGTGTGTTCAGACACTTGGACAAAAATGGAGAT
GGGAAGCTATCGCCACCGGAGCT
CCAAACCTGCATTGGAAAGGTTGGTGGAGAGTTGTCACTGGAGGAAGCCGAGATGGC
AGCTGCTTTGATAGATTCAGATG
GAGACGGGTTGTTGAGCATGGAGGACTTGGTGAAAGTGGTCGAAGGTGCAAACGAAG
AGGAAAAAATTAATGATTTGAAG
ATGGCTTTTAAGATGTATGAAGAAACGGAAGGGAGTGGATGCATAACCGCGGAAAGCT
TGAGAAGAACGCTTAGCAAACT
GGGAGAGTCGAAAACAGTTGATGATTGTAAAATAATGATTGCTAAGTTTGATGTTAACG
GTGATGGAGTCCTCAATTTTG

ACGAATTCAGGGAAATGATGGCGTGA
>CsCML19
ATGGCAACTGAAACAACAAACCCTACCACAACTACCCAACAAACATCATCATCAGTGA
ACCTAACCAACATCGAAGAAGT
AAAGAAAGTATTCAACCGTTTCGACACCAACCACGACGGCAAGATCTCATCTTCGGAG
CTCATCTCCATCATGAAATCCC
TCGGATCCAACATCTCAGAGGACGAAGTCAGACAAATGATGACCAAGATTGACACTGA
TAACGACGGGTGCATAACCCTG
GAAGAATTTGCAGGGTTTTGTAAAGATGATACTGCTGATGATGGGGGAATGAAGGAGT
TGCATGAAGCTTTTGAGCTTTA
TGATTTGAATAACAATGGTTTGATTAGCTCTAGTGAGTTGCATCAGATTTTGACGAGGT
TGGGTGAGAGTGTGAGCGTTG
AGGATTGTGTTGGGATGATTAAGTCTGTTGATGCTGATGGTGATGGCTTTGTAAACTTT
GAGGAGTTTAAGAAGATGATG
AGTAATGGCAAGACTGAGCCGTAG
>CsCML20
ATGGGTTTAAAAAATTTGTTCAAGGGTAAAAAAAAATCAACCCAGAACAACAACAAC
GTGGTCGTGGGTGTTGCTGTTAG
AAATCCAAACAATACATCATCGCCAGTTCTTTCGCGATCAAGCTCGTGCAACTCACGTG
CTCGGATCGAGGAAGAACTAA
CGCAAGTTTTCAGAAAATTCGACGTAAACGGAGACGGAAAGATCTCCGCCTCCGAAC
TGGGATCAATAATGGGGTCCCTG
GGCCAAAAACCCACAGAAACCGAGCTCGAAAACATGATCAAGGAAGTTGATGCTGAT
GGGGATGGGTTCATAGATTTGCA
TGAATTCATTGCTCTTAATACTAAAGATATCGATTCAAATGAGCTTTTGGAGAATCTAAA
AGAAGCCTTTTGTGTATTCG
ATATTGATAAGAATGGTTCTATAAGTGCTGAAGAGCTGCAGAAAGTTTTGGGCAGGTTG
GAAGAAAGCTGCACCATTGAA
GAGTGTAGGAAAATGATTAGTGGTGTTGATGTTGATGGAGATGGTATGATTAGTTTTGA
TGAGTTTAAGGTTATGATGAT
GAGTGGTAATGGGTTTGTGTCTAAGAAGAATAATAACCGAGAGATGAAGGAAGAATAA
>CsCML21
ATGAGCAAAGTGTCAGTAATACTCTACATTACAGTAGCCATACTAATCCTCATTCTAATC
TCCAACAAAAACACCAACAA
CACCTCCTCCAACCGCCACAACCGCCGCCACCGCCGCCTCAAGCTCCGCTCAAACTTC
ACCACCACCCCCATACCCACCA
CCCCCATACCCACCACCCCATCAGATCACCATATCTCATTTGACCCCCTTATCGCAGACA
TTGAACGTAAACGTGAAGAC
AAACAATGGGAACAAACCCATTACTTTAATAAGGACCATGGTGATGTTCATGATCATGG
TGGTGATGACGTGGCACCTGG
AATGGAGGGACAGCCAGAATGGGAGGATTTTATAGATGCTGAGGATTATTTGAATGATG
AGCATAAGTTTAATATTACGC
ATAGGTTGGTGTTGTTGTTTCCCAGGATTGATGTTGATCCTGCTGATGGGTTTGTTTCGG
AGCATGAGTTGACGCAGTGG

AACTTGGAGCAGAGTCAGAGGGAGGTGTTGCATAGGAGCCAGAGGGAGATGGAGCTG
CATGATAAGAATCGGGATGGGTT
GGTTAGTTTCCACGAGTATTCGCCGCCTAGTTGGGTGAGGGATACAGGTAATAATTTCT
TTGTATTTTGGCAACTTTTTA
GTGGTTGATTGGTAGTATAGAAATGTTGGCATTGTGTTTATTATGTGGTTATGTAATGTG
GAGTACTTAGGATGGCATAA
TTAGTTGTAAGGAAATAAATTTGAAGTTGTGATACGAGTAATTATTTAAGGATTTAGAGA
TTTAAATGTAAAATGGCCCC
TAAATACTGAGATAATCATATGTGAAATTTGGGGTTGCTTTTTGAAAAGCAGTTTGGAA
TAAGCAACTCCATTAGTACAT
GTTGCTTATCCAAGACAGCTTATTTGACAGGTGAATAATCTATAAGCAATCCCGAAAGC
TCTCACAACTGCTTATGTGTT
GTTTCTAAGTGCAATAAGCAAATTTGTTAGCGATCCTATGCACCCCATTCAGTTTGTTGT
CGCTGATCACTTGAATAACC
AGAATCAGTTTCACTACCCAAACGCTAGACGCTGCCATACATATCCTATACGTAGCTAG
TAACATCAGGTTTGGGTTTTT
AACGCTACCTTCTATTAGGAGTTAAACTGTAATTTCAAATTGATGATTATTTCTTAAATGT
AGAGAAGTTGGTCATTGCT
GCATTTAACTGTATGAAGGTTTGATGATGCAGAATAGTTACATGTTCGGGTAAAAAAAA
ATTAAGCACTAGGTACAAAAC
ATGAACTCATTCTATTCAGAACTGTTAAAAGTGTCACTTAGGGAACTTGTAGCGGATAT
GTAATCATGTAGTTGTGAGTG
GTGTATTTTGAGGGATATCTAGTATCGATTTACATGCTGAATCTCGTAAGAGGATAGAGA
AAGAAACACGCCATCTAAAA
AATGCACTTGAAGTATTATATGCTAGGAAGAGTCTGTCTGCGATACGAAAGTGGGTGCT
TTATGTTTGTTATGTAAGATT
TAGAAAGCTTTTGTTATACCATCTTAAAAGTGTTTCAGAGATTCAGAGACTAAACTTAA
TGTATGCATTATCTTTCTGAT
TCTTATGATTTACAAGATACCATAGTGGTCTTAGCATAAATGATTGTTACTTGAAGAGTT
GAAGTAGGTTTCTGTATATT
CTATAAAATTTGGGTATTCCACTTGCAACTTAATCTGACTATATCTTGAGCAGATAATAA
CTCATTTGGGTATGATATGG
GTTGGTGGAAAGAGGAGCATTTCAATGCATCTGATATTGACGGTGATGGTTTTTTGAAT
TTGACCGAGTTCAATGAGTAA
GATACATGCTCTCTTCAAGTATATGAATTTGTTGCATCTTATAGACTCATATCTTGTTCAT
ATCCTTAATTTGTGTTTTC
TTTCGTGATATATACAGCTTTCAGCATCCCGCAGACACTAAAAACCCAAGACTTCTTCA
GTGGCTGTGCAAGGAGGAAGT
AAGGTTGACATGCTACTTTCAACTAGTTATATTTTAGATTAATTTTCTAGTACATGTTTGC
CACACACACACACATGTTT
ATTATATCTCAACTATGTATTATAAATAGATTCTCCTAAGGAAATGTGCATGTTCTTCTTT
GTAGTGGGGTTATTGATTT
TTTAATCCTATTGGGGAATTAGTAGCTTTCCACTAAGTACGTGTCTCTTTACTAGGTGAG
ACCTAGTCAATTCTGTTTAT

GTCCATACAGGTCTGCTGCTAAAGTTTAGATGTTCTTTGTGTTTCAAGAAAACATTTTTA
GAAGTCGAATGCCCTTTTAG
ACTAGATATTGCGCCTTTTAAGCATATGCTTAAGTGGGCACTCAAGATTGATGCAGCAG
AATAATAAAAAAAAGAAAAAA
AAAATCTAGTTTGGCTTCTGTAGTTTATGATAGTGTAATATGCTAATTGCCAAAAGCAAG
GAGATGAACAGATGGTGTTT
GGATGTGTCTTTACTTATATAAAGTTGTAAAAATGAAACAATTAACTGTTTGAGCGTTTG
TCCAATTCTCTTCATGTAAG
AAGTTTTTTACATAAAATGACCGAAACGGACATTTATATGAAAGAAGGGAGCTACATCT
TGTGAAATGGAAGTAAAAGTC
AACAAGAATTTAGTTTTATTACAGCTAGTATGAAACTAATGAATCCAAAACGAATTTGT
TGCAGGGAACGAGATACTGAC
AAAGATGGGAAGGTGAATTTTAAAGAGTTTTTCCATGGACTATTTGACCTCGTAAGAA
ACTATGACGAAGAAAGTCATCA
TAATTCTTCACATGAATCTAATAACGATTCATTGGAATCACCGGCAAAGAGATTTTTCTC
CGAGCTTGACAAGGATGCTG
ACGGGTAAATCCCGCACCTTTACGCTTTATCACTATATAGAATACATGTCCATAACTACG
TAATTTTGATTATGCTAACT
TTTCTGTTGTAGATTCTTGTCTGACGTGGAGCTACTACCTATCATCGGAAAGCTTCATCC
ATCAGAGCGTTATTATGCTA
AGCAACAGGCAGATTATATTATATCACAGGTAAGAATGTTTGAATATAATTTTTCATGTC
GCTGTGTATATATGAATATA
GAATCCACAATATGATAATCTTAATATTATTTCAGGCTGATACGGATAAAGATGGACGGC
TATCCTTGACTGAAATGATC
GATAGTCCATATGTATTTTATAGTGCTATCTTTAACGAGGATGATGAAGAAGATTATGAAT
ACCACGATGAGTTCCGTTA
A
>CsCML22
ATGAAAACCATGACATTAAAAGAATGTTGTTGTTTCTTAAAGAAACTACGGAATCCTAG
TCGAATTATCCAACATGCTCT
TCTTGCTTCCGAGACATCATGTTAGTAGCGAAAATTTACATATTTGACACATAATTTAGT
GCGAGTTAATCTAATTGGCA
TCTTTTTGTTTCACAGTTACCATCAATGAAATAGAGGCATTATACGATCTTTTTGAGAGA
TTAAGCTATGCTATCATTGA
AGATGGCCGTATTCACAAGGTATTATAAAAAAATAAGTTATAGACGTATATGAATCATGA
ATTTTGATAGCAGTGCTCTC
ATTGCAAAAATAATGGAAAATCATTCAAATGTGTCTTTTTTGCATTTTATAAACAGGAA
GAGTTCCGGCTTGCTCTTTTC
AGCAATAGCACCATGCAGAATCTCTTTGCAGATAGAGTGAGTACACAGTTTTCGAAAA
TTTCTTATCTCAAATGTGTTAT
TTCTGCCTTAAACGTTGAGATTTTTTAATCGTTTCAATCTAGGCTGTCAGCCCAAATGTT
TGATTTCTTGATTGCATTAC
TGCAGCTATTTGAGTCATTTGACATCAAGAAAAAATGGAGTTATTGAATTCGATGAATTC
GTTCGTTCGCTAAGCATCTTC

CATCCTAATGCACCAGAATCAGATAAAATTGAATGTAAGATACCTTTAACACAAGTTGA
TGATCTATTGCCACAAAACGA
GTTTATTTTACTTCTAAAAGTCGTATTTGACTACTTTTGAAATTTGTGTCATTGTAAATTC
ATTGATACTGACATTATAT
AATATCATTGCAGTCATGTTTAGATTATATGACTTGAGGCACACTGGATTCATAGAACGT
GAAGAGGTAAATTAAAACAC
ACTAAAGTTCCGACCTTTAGTCGATGTTTAACTTTTACTGAACTCTGATCTTGGAAATAC
TTCAAAATTTGAAGTTGAAG
GAGATGGTAGTGGCTCTCCTGAGTGAAATGGATGTGAGTGTATCAGATGAAGATATCGA
AGCTATCCTAGACAAGGTAGC
AATCATAGTTTTAAGGCTTTAGTTATAACTTTACAATCTTCAACTTTTGACATTTTATTCG
TGATCTTTCGTGTTTTGTC
TTTGACCGTTCCCTCTTATGTTTGATCAGACACTTTTGGATGCAGATCTAAACGGGGAT
GGAAAGATTGATTTAGAAGAG
TGGAAGATATTCATCTCAAAGAATCCATCCATTCTCAAGAACATGACTCTTCCCCTTCTT
AGGTAAGTCTTGATTATCTA
GTGCTTATTTTGATCATAATAACTATAAGCTGTTGTTTATAATAGTTTATTTTGATCAAAA
CAACAGGGAGATCACTCAA
GCCTTTCCAAACTTCGTCTTGAATACTCAAGTCCAAAACCTAGAAGTAGAAGTATAG
>CsCML23
ATGAGCAGTGACAAAGAACCAACCGTGAAACTCGACGACGAACAGCTAAGCGAGCTA
CGTGAAATATTTAGATCATTCGA
TAGAAACAACGACGGAAGCCTAACACAGCTTGAACTCGGTTCATTATTACGCTCACTA
GGCCTCACACCTAGCTCAGATC
AACTCGATACATTAATACAAAAAGCCGATACAAATAGCAATGGTTTGGTTGAATTTTCT
GAGTTTGTGGCACTCGTGGCA
CCCGAGCTTCTTCCTGCTAAATCGCCTTATACAGATGATCAGCTGAAGCAGCTGTTTAA
GATGTTTGATAGGGACGGAAA
TGGCTATATAACGGCTGCTGAGTTGGCTCATTCTATGGCGAAACTAGGACATGCTTTGA
CGGCTGAGGAGCTTACTGGGA
TGATCAAGGAAGCTGATACGGATGGGGATGGACGGATTAACTTTCAGGAGTTTTCTCG
CGCCATTACTTCAGCTGCTTTT
GATAATTCTTTTTCGTGATTTTGGTTATTCGTTTGCATACCTTTTTGTTTTCAGGTATGGT
TTTCGGTTTATCATGATCG
TTTGTTATTTCAGTTTTGAAAGAGATTAGTTCGTTATATGTGTTTAAGTAATAGAAAATC
GGCTTTGTGTTTAGTATAAG
TATAAACCATAGTGATTATTTGATTAATTTTGATCGGTTTACAGCTGCAAAAGCCTGCAA
CAAAACTAATTTTGATTTAT
CAGTTACTGTCAAACACTAAAAATCATAGGTGATATCAAATTTTTACGACTTCAGCTACT
AGAAGAACATCCAAACATTA
TCTACTGCGACCCGATTTGTTACTAGTCTATCTTTTCTTATTTTGATGAATTCATATCAGA
TAATCAGATTTCAAAATAT
TATGTGAAATATTCCTTAGTGTTTTCGATTTTTTTTACAGGAATTAGATTCTTTAGTTATTA
GAGGTGAATTACACACCG

CACTTTTTTCTTTGTACAGCACAAACATGTTTCTCTTGAGCATCTCACTCTTGCAGGCT
GTTGTTGTCTTATTGGTACTC
TAGGTTGTTTGGTCTTCCAACTTTATACATAAAATTTAGGTTTTGTTAGTGTAACCAATAT
ATGTGAAAACGTCAGATAC
TAATTACAGAGTACTAAAGACTAACATTGATGTTTACACGTTTACAAGAGTTAACAGAC
AAAAATTGGTCTTGATTTGTG
GCTCGCAGGATTTTTAATAGCAAACTATCAACCAAAACTGACAGATATTATTTGTTGGTT
CACAGATGA
>CsCML24
ATGGGCTGTTGTTATTCTGCCATGGGTGTTCCGGTTGTTGCCCGTGAAGATTCTAGGAC
CCTTGCTTCTCAAACAACTTG
TAAGTTGCTTCGCTAATTGATAGTCACTTTGAGTTTTATATTCATCGTATGCCTATGAACG
TGATATATCTTAATTGAGG
AGTACATAAAATGTAACAACAAACTGGCTTGTGTAACTTCTATAGAACATTTATGCTCA
AGGTGAACCATATTTTGATTG
TATCACCCAATATTAAAATTCTTTGCTTTCATTTTATAAACAATCATCTCTATGCAGTTAG
TGTTAGTGAAGTTGAAGCA
CTATTTGAGTTATTCAAGAGCATAAGCAGTTCTGTAATCGACGATGGGCTAATTAGCAA
GGTATTTTATGTTAGCATGTC
TAGTTAAGAATCATTCCGAATTTGTCCATGTATATCCATTAGCGATGATTTTTGTTCAATA
TGCTATGCACCTCTATTTT
TTTGGATAGCGATATCTGATAACGCACATGCTATAATGCAGGAAGAATTCTTACTAGCCT
TGTTCAAGAACAAAAGGAAA
AAGAATCTGTTTGCAAATAGGGTACTTTCCTCCAATTTACTTTCTGATATATGTAGCAAG
TTACTTCAATATATAAGACC
AGTCTGAATGAATAATACGGAATTTGTGTATTGTATCAGTTGTAATATGACATCTTTCAAT
ATGGACAGATTTTTTCTCT
TTTTGACGTTAAGCGTAAGGGGGTCATTGATTTTGGTGACTTTGTTAGATCACTAGGCG
TGTTTCACCCTAGAGCTCCGT
TAGAAGATAAAATTAATTGTAAGTTGGTTATTCTTAATCTTAATTGGATTTCCTTATCAAC
GTAATAATCTTATAACTAC
TTTTCTGCTTTAGTTTCGTTTAAGCTTTATGATTTGGAAGGAACAGGCTTCATTGAGCGC
CGAGAGGTACTGTCTATTCT
AATTTTGCTCTTATACAAATGAAACAATAGCGTTATTTTTCAATATCATTTTCTCCTGCAA
TTTTTGCAGGTGAAACAAA
TGTTAATTGCACTTTTACATGAGTCAGAGCTAAAACTGGCTGACGAGGCTATTGAAATG
ATACTGGACAATGTGAGTTTG
CTGCTAATGGAAATATTTATGCCTTCCTTTACGTATTTGGTAAACTTGATTTGAATTGTTT
TTTTCTTTCTTTCTGGCAG
ACATTTTCAGAGGCGGATGGAGACCGGGATGGAAAGATAGACAGATCAGAATGGCAC
GCTTTTGTTACCAAGAATCCTTC
CTTGTTGAAGACAATGACACTTCCTGTTTTAAGGTATGTCGTATCATAAGATCATGCACC
AATACATTATGAATACTAAC
GTCAAACTTTTGTTACTTACTCATTATTCTTTATAGATTCGATCTCACTTTGATATTTGTA

AAATGCCAGGGATATTACA
TCAGCATTTCCAAGCTTTGTTTTCAATACTCAAGTTGATGAGATTTCGTCTCCTCAAGTT
GAAGAGGTTGAGATTTCATC
TTCTCAAGTTGAAGAGATTGAGATTTCTTCTTCTCAAGTTGAAGAGATTCCAAAATGA
>CsCML25
ATGGACAAAGAACAACAATACAAACGTGTATTCAGACATTTGGACGTGAATGGTGACG
GGAAGATATCGCCACCCGAGCT
CCAAATTTGTATTGGTAAGATAGGTGAGGACTTGTCATTGGAGGAAGCAGAAATCGCG
GCAGAGTTAATGGACTCGGATG
GTGACGGGTTGTTGAGCTTCGATGATTTGGTTAAAGTGGTCGAAAGTGCTAATGAAGA
AGAGAAAGTAAAAGACTTGAAG
TTGGCATTTAAGATGTATGAAGAAATGGAAGGCTGTGGCTGTATAACTCCTAAGAGCTT
GAAGAGAATGCTTAGCAAATT
AGGAGAGTCGAGAACTGTTGACGAGTGTGAGTTGATGATCAAGAAGTTCGACCTTGAT
GGCAATGGTGTACTTGATTTTC
AGGAGTTTCAAGACATGATGTCATGA
>CsCML26
ATGGACAAAGAACAACAATACGAACGTGTATTCAAACATTTGGACGTGAATGGTGACG
GGAAGATATCGCCACCCGAGCT
CCAAATTTGCATCGGTAAGATAGGTGAGGACTTGTCATTGGAGGAAGCAGAGATCGCG
GCAGAGTTAATGGACTCGGATG
GTGACGGGTTGTTGAGCTTCGATGATTTGGTTAAAGTGGTCGAAAGTGCTAATGAAGA
AGAGAAAGTAAAAGACTTGAAG
TTGGCATTTAAGATGTATGAAGAAATGGAGGGTTGTGGCTGTATAACTCCTAAGAGCTT
GAAGAGAATGCTTAGCAAATT
AGGAGAGTCGAGAACCGTTGACGACTGTGAGTTGATGATCAAGAAGTTCGACCTTGAT
GGCAATGGTGTACTTGATTTTC
AGGAGTTTCAAGATATGATGTCGTGA
>CsCML27
ATGGAGTCTAATAAAGTTCCAAAATCTTTTGGATGTTTTCCTCAAAAGGGTCTCGTTTT
AAGGTTAAGTAGTTTTCGTTC
CAAGAGTAACAATACTTCGAACTCCACTTCTCCTACGTTAATGTCTCCTAGATCGCCGA
AACCAAACACTAACAACAGAG
AACAAGAGTTTAGATCAGTCTTTGCGCGTTTTGATGCTGATAATGATGGAAAGATTTCA
GCATTGGAGCTTCGGTCATAC
TTTGGCTCCATTGGTGAGTACATGTCGCACCAAGAGGCTCAGGGCGTGATCGATGATCT
GGACACAGATGGCGATGGCTT
TATTGATTTTCAAGATTTTATGAGGCTAATGAAGGTGAGCAATGAAAAGGATGATGTGA
AGGCGGCGTTTGAGATGTTTG
AGTACGAGAAAGGGTGTGGACAGATTAGTCCAAAGAGCTTGCAAAAGACATTAAGTC
GACTTGGCGATTCAAAAACTTAT
GATGAGTGTTTGCAAATGATTAAGATGTTTGATACTCATGGCAAAGGAGCTGTTGATTT
CAATGAGTTTCAACAGATGAT
GACGGCTTAA

>CsCML28
ATGGCACAAACCGACGTCGAAAAAGTGTTCAAAAAATTCGACGTGAACGGCGACGGC
AAGATCTCCATAACCGAACTCGG
CTCAATCCTTGCCGCACTCAGCGGCGCCGTCACATCTGAAACCGAACTGAAATCGGTC
ATGAAAGAAATCGACACTGACG
GTGATGGATTCATTGATTTTGACGAGTTTGTCGCGTTTCATAACGGTAACGGTGAAGAA
GAAGAGAGTAAGGAGTTGCGT
GAAGCGTTTGATTTGTATGATGAGGATAAGAATGGGAAGATCTCAGCGAATGAGTTGC
ATTCGGTTATGAAGAGGTTAGG
TGAGAAGTGTTCGTTGAAGGATTGTAAGAAGATGATTCAGAGCGTTGATGTTGATGGT
GATGGTTGTGTTAATTTTGAAG
AGTTTAAGAAGATGATGAATAAGTGA
>CsCML29
ATGCCTGCGGGCTACAGCTGGGCCAAACCGCCTGCTATGACTCTTGTCCATCAAAATCT
TTCTATGGGTTGCTTTCCAAG
ACTTGTAACTCCTGCATTTGAAAGTTACTCTTGCCGTTGGACTATTCGGACATCTGCATT
ATTCGTGCCTCAGCAACGTC
TTTTCCATGACTCAGCTAACAATATTCTGATATTGCTTGCTATATTGTCCCTTCTGAGTAG
ATGGGGATATCTTGAAAAG
CAAAACAAAGCACAGCCAAACATTAAGTCATGTTTTATGGTAATATCTTGAAAAAGAAT
ATTAATCTAACAATTGAGCAG
TCCTCTGCGCCCAGTCGCAACCATTGAAACTCTATTGTTGGAATCAAAGACCTGAACA
CAATTATTTTTGATAGCTGATA
GATGCTGTTTTTCACCATCATGTCAAGCAAATTTCAGGTTACAAAATCGAATGGTTGTG
ACTGCCTGTTAACAATAGATC
GACTTGTGTATAATTAAGGTCAACAACAGCACATGCCCAAGTGGTCGAAATGACCGAT
AACAAGGCTAGGATAATCTTTA
CATAGTAATCTAATTATGTCGCTGCGTTCCCGAGTTTTATTTTTGAAAAAAGAGAAAAG
AAGTGAGAACAAATCAAAATG
ATCATTCCTAAGTACTCGTCAAATGCATCCTTCCACATAAAATATCCGCATCCATTATCTT
TTCTGGGCTGTTACATAAT
AATAAAATTATAAGATATTACAACCCAATTATTTGTATATTTACATATATAGGAAATGTATG
ATTGTAGGACATGAATAA
TATTGTCGTTCAGATGAATAATCTCTATGTGATGTTCTTAATTTTATATTTCCTGCTCCAC
AAGAACACATAAAAGGATT
AAATTGCTCCATCTCAGGTTTTGAAATTTGTGTGAAGATAAGATATGGTAGAAATTTGT
GAAGATAAGATATTATGGTTT
TGAGGATAAGAAATTTGTGAAGATAAGAAATTTGAGGATAAGATATGGTATATAACGTG
TAATTTTTCTGGATAAAGGGT
TTTGCTGGGGACATGTCCCCGGAAAAAGGTCTGGCGGGAATTTTCCCGCGAACAATAT
TAACATGCTTTAGACTTTGGTC
AAAGTTTGATAAAGGTTGAAAATAGTTTTGGCGGGGACATGTCCCCGGAGAAATTTAC
GTTGAATTTTGACTATCCGGTC
AAAGTTTAATAAAAAATCAAACTTACTTTCGCCGGGGACACGTCCCCGGCGAAACATT

ACGTTGAATTTTCATTAACAAG
TCAGCGAATTCATGTGCCACGTCAGATTCAGTTTCGTCGTTTAGTGACTTTCGCCGGCG
ACTTTTTGTCCCCGGCAAAAG
TCTCCTCGCCCGTCCATCCGATTGTTGTAGTGTGAATTCGTGTTCCACAATTTTTTTCAA
GAAAGAAGAGAAGAGAAGAG
GAGAGGAAATTTGGCTAAACTTCTCTCCAAAACTTTCTTCCCAAAAGTGGGATGATTT
GGAGAGAAGTTATTTAGGAAG
CTTTCAATAAATGTAATTACCTAAATGACCCAATATAAATCATAACTTTTATATATAATAA
AGGGTATAATTGTAAAATC
ATCAGTTATCTCTTCTTTTCTCTCTTAACTTAGGAACATGATATAAAAAATAAAATTTCAT
TCATCTTCTCTTCTTTTCT
GTCCATTTTTAACTCTTCTTTTATTTTCTTTCATTTCAAAAACTTGGGAACAGAGTGTTC
AAGTGTACATTGCGGTATTT
TTTCTTCCTTTGCATTGGTATTCTATTTATTCATAATGTTCGCCAATCCCATACTTTTCTTT
GCAGACTAACTTTGTGTA
TTTGCTCTTACTTTCAAATGATAACCATAATAACAAAACACCCTTCAAAGATGCATGTTG
ACAAGGACTTTATCATAAAA
GAATCGATCTGCACACTAGCTTTTTGGTACGTGCCAAGCTAGAAAGATAAACAGCATAC
TGAAAACTACTTACTCTTTAC
GTATGACTAGCTAGCGATCTTAGTTTCTTTCATTTGACATCCCAAAAATAGTGCAACAAT
TTCTTGCCGTACATTATTTT
TGTTGGTCAAATCAAGTGGATGGAGTATTTTTCGATATAAGAATAACTTGATGGTATATA
ATCCGTTGTGATTAGATGGT
ATGTCACCTGGAATGTATAGCTAGCTAGTCTCTTTTTATTTTTCGATTCTATGATAGTTCA
CTTAGTTTCTCAATGTTAT
GGTTATGATATAATGACACCGGTCTGGGTTTATAAAATGTCTTAATATTACAAGGGACTG
AAAATATTTTGTTTAAAATA
AACTAATCTAAGACTATCCTAATTTTTAAATAGTGTAACTTTGAGGATAATAAAAGAATT
TGATTCTTTTAATCCAGGCT
CTTAATTAACATCCAAATTAAAGGGAAAGAATAACGAATACCCCTTTATTATAAAACAA
AGTGCATCTAAAATGCACTTG
TGTTCGTGTATATATATAAATGAATCGACTCCTTGGTCCTAGCATGCAATTCAAAGCACT
TTCTAAGACATTTAGCATAA
AACTTTCTTAATTCTATTATCTTTTGTTAAGTATGCGAAAGGAGTGGCGTCTTAAACAGG
TAGAAGCACCCTTGACAGAT
GATCAAATAAAGGGTCTAGTCAGCAAGTTTGACACTAATGGAGATGGAAAGATCAGTC
GGAGGGAACTGAGGGTAGGCTT
GAAGAGTCTTGGCCTGCATTTCGCAGGCTTAAGAGCTATGGGTGCAGTACGTCATGCT
GATGCTAATGGAGATGGCGTTA
TTAGCGACGAAGAGATAAACGAGCTTGCTAAATATATTTCCAAGTGGGGAATTTCTGTA
ACTTAA
>CsCML30
ATGCGGCAGTCCTGGTGTCTTAGACAGGTAGAAGCGGCGCCCCTGACAGAAGACCAA
ATAAAAGGTCTGCTCAGAAAATT

CGACGCAAATGGTGACGGCAAGATCAGCAGAAGGGAACTAAGGGCTGGCTTGAAAA
GCCTTGGTCTACGTTTCGCGTGCT
TTAGAGCTAGACGTGCACTGCGTTATGCTGATGCTAACGGAGATGGTGTTATCAGCGAT
GAAGAAATAAACGAGCTCGCC
AGATATGTTTCCAAGTGGGACATTTCTTTAACTTAA
>CsCML31
ATGACGCGAAAGTCGTGGTGTCTTAAACAGGTAGAAATGCCCATGACGGATGACCAAA
TAAAGGGTTTAGTCAACAAGTT
CGACACTAATGGAGACGGAAAGATCACTCGGAGGGAACTGAGGGTAGGTTTGAGGAA
ACTTGGCCTACGTTTCGCAACCT
TTAGAGCTATGGGTGCAGTACGTTATGCTGATGCTAACGGAGATGGTGTTATTAGCGAT
GAAGAGATAAACGAGCTTGCT
AAATATATTTCCAAGTGGGGAATTTCTGTAAATTAA
>CsCML32
ATGTCGCTTTCGGAACAATTTCATATCATAGGTGACTTGCATGTATTTTCCACACAATTT
TCCAATTTATGTCTACATAA
AGCAAAGGTTCCAACATTCTTCGTCGTGCCATACTAAGTTAACTACTAACAATTCTAGA
TTTTAGAAACTAACAAAATGA
TTTTCGTGTATATGTTCATATCTTTCATGGTTATTGAATGGTTTATCTTGTTACATGACTTG
TATTCGCTTTTTGCTCGT
CCTTTGTTTCAGTTTATCATTGACACACCCCCAACAAAGACTTGCACGGCAGCCACAG
CACACGAAAAGGCTCATGCAAC
GAACCTTGGAGGTGTTGAGAATGGCCCGGGTAAGAAGAAGGCGTTGGGAGTGAATCC
AAAGACTATGATGGAAAGATTGG
GAACGTTTTGGGATCCTGATGACCATAGGGAGGCTCTTGATATGGACGAAATGGTGAAT
TTGTTTGTTGAGGACGAGCCA
AGCTTAGATGAAGTGAAACAAGCGTTTGGCGTGTTTGATAAGAACAATGATGGCTACG
TAGATGCAAAAGAGCTACAAAA
TGTGCTTTCTAATATGGGCTTTTTACATATATGTGAAAGTGATTGTAGAAGGATGATTGT
TAGCTATGATGCTGACAAAG
ACGGTAAGCTTAGTTTTCGCGAATTTTTGAAGGTCGTGGAAGATGGCTTTCGGTAA
>CsCML33
ATGTCAACATTGCCCGAGGACCAACTCAATCAACTCAAAGAAATATTCACACGTTTCG
ACCTAGACAAAGACGGGTCCCT
AACACATCTAGAGGTCGCAGCCCTCCTTCGGTCTCTTGGCCTCAAACCATCTGGAGAC
CAAATTCACAAACTATTTAAAA
ACATGGATTCTGATGGCAGTGGGACCGTGGAATTCGACGAATTAGTGAATTCCATGTCG
TCCCATATGATGACTGAAGAG
ATATTGGTTAATCAACAACAACTTATGGAAATATTTCGATCTTTTGATAGAGATGGAAGC
GGGTTTATAACGCCTGCTGA
GCTAGCGAAATCTATGACGAAAATGGGACAACCTTTAACGTATCGCGAGTTGTCTGAA
ATGGTTCGAGACGCGGATACGG
ATGGAGATGGTGTTATTAGTTTTAAGGAGTTTCAAGGGATTATGGCTAGGTCGGCAGCT
GATTCACTTGGGTTCTCATTG

TAA
>CsCML34
ATGGTTGTAGTCCATCTAGATCCAATCCTGATCCAGTAAGATACACAATTCATTAAAAAT
AATAAATTTGTAGATTTTGT
TTACGTTATTTCAGTTGACCAATTTTTTAAACCTTCATTTCCAAAAAAATTACAAAATTC
AGCAAATTTTATAAACACAA
ATTTTTTCTTTCTACATTTTTAGTTATAATCCCGGCAACGATGGGCGCGGCGGCGAGCTC
GTCATCATCGGTACTTTCCT
CTCTCTTATATAAACAAGTATCCTTATGGGGTGTTTGTTTCGGCTTACACAGCGGGCTTA
TGGCTTATTTGGTTTTGGGC
TTACGTTTCGGAAAGGCCATTTTTTATGGGTTATTTTGAAAAACTTTTCGGCTTATATAA
ACTTATTATTTGTCATAATA
AGGTTATATAAATATAAGCGAAAAGTTTCAAAATGACCCACAAAAAGTGGGCTTACGA
AACGTAAGCCCAAAACCATATA
AGACATAGGCCCGTTATATAAGCCAAAACAAACACCCCCCGTATGTATGTATGTGTATG
AAACTGTGTATGTGTATGGGA
CTGAAAATGTGAATGTGGATTAGGAAATATAGAGATCGAGCTCGCGGACATTGGGTGTT
TGTTTTGGCTTATATAGCGGG
CTTAAGACTTATTTGGTTTTCGGCTTATGTTTCGTAAGCTCACGTTTTGTGATTCATTTTG
AAACTTTTCGGCTTATATA
AACTTATTATGTGTCATAATAAGCTTATATAAATACAAAGCCGAAAAGTTTCAAAATAAC
CCACAAAAAGTGGGCTTACA
AAACATAAGCCCAAAACCATATAAGCCATAAACCCGCTATATAAGCTTAAACAAACACC
CCCTGTATGTATGTGTATGCA
AATGTGAATGAGTATGTGTATGTGTATGGGACTGAAAATGTGAATGTGGATTAGGAAAT
ATAGAGATTGAGCTCACTGAC
ATTGGGGGAAAAGTTATGTGCGGCTTTGATACCATTTGCGGTTTTAGCTGAGGTGTTGA
TTTTCAGCTTCTCGTCTTGTT
TTAATGATCGAGGGAGGAAAGCAGCTAAACCGGCACTGACTTTTGATGACATTAGACG
GCTGGCTCTTAATTCGCCATGT
TAGTTTATGTTTATTTCTTTTAGTAGGTTTTGTTGGTTTAGGATTTATTAATGTTGTTTGAA
TTGTTTCAGTTACGGTTA
ATGAAGTGGAAGCATTGCGTGAATTGTTCAATAAGTTGAGCAGTTCGATTATAGACGAT
GGACTCATACACAAGGTATGT
TTTCTCATTTGATTTTTACCAAATTTCGTGCAAAATGCTTGGTGGTGCAAATATGATTAC
CGACTTGCATTCCACTGATG
TCTAATGACAATGCATAATACAGACTAATCAATGAATGTATGTAGAACAGTAGAAGGAT
TTAAGTTGAGTTAACGTGAAA
CAACATATATTTGCGATAATGGAACTGCGTCTGATGAATAATACGATGTTAATAGAGGCT
AGGGAATACTTAATGGCATT
GTGAATCATTAGATTCGTCTGACTCTTATGAGCATTACTCAGTTAGTAGACAAAGGCATA
GCACTTATGTTCAGAACAGA
GGAGCTGCCATGAGCAAGTATATACTTACCTGTGTAGTAAACATCTTCGTGAGACTAAA
TCTGACTGCTGCATCGCTATA

TAAAATAGATATGTAGAAACAAGACCAAATTGTTGCTAATGAAACACAGTTTTATCAAT
GCACGTCAAGATTAACATGAT
ACAATGATACCACATTCCTCATCTTATCCTTGTTTTTCTTAACAGGAAGAGCTCCAATT
GGCGTTGCTCAATTCAGCCG
GTGGAGAAAATCTTTTCTTGAACAGGGTAAATCCTTTATTACATTCCATGTTTCCATCTT
AAAGCGGCTTTACATTAAAA
GTTTGGAATGTAAATCGGTACCCTATTGACCTATTCTGCTGACAATTAATCAAAGGCTTT
GCCTTGCAAATAAACTCTGA
AAGTAGAACAGTTTGTAGGTCACATGATGTAGTTTTGTACCTAAGCCAAGCTTAAGGA
ACGTTTCTGTTGATACATATAT
GAATGCTTACTCAGACAGAATTGCATATACAATAAGTTTAAGGCAATGGTGGTGTTGAG
GTCTTGAATTCGAGGAGTTTA
TTCTTGTATTGAGCATCTTTCATCCATTTGTAGGTTTTTGATCTATTTGATGAGAAGCAA
AATGGCGTTATTGAATTCGA
GGAGTTTATCCATGTATTGAGCATCTTCCATCCTTATGCCCCAATAGAAGAAAAAATTGA
TTGTAAGTTCTAAGAACACA
ATCCACATGAATTAGTTTTGCAGGTGTCTTCATGGTATGGTACTGTCTTTTCTCATTTTTT
TCCCATTTTATGCAGTTGC
CTTCAGGTTATATGATTTGAGAAAAACCGGGTACATAGAACGGGAAGAAGTAAGTGGT
TGATCTGTTTCCTATATTAGCT
CTGTCTCACATTCATTATCATTAATGGCGTCTTCTTATATCAGGTTAAAGAAATGATTGTT
GCCACTTTAAAGGAAACCG
GGATGCGTCTTTCAGAAGAAATACTTGAAGAGATAATTGATAATGTAATACTTCTTAAG
GTTGATTACTTTTTCTTTTTC
TTTCTAAGTTAACAGGCATTCGTCTTGTGTTTTTGTGTTCTCAGACATTTGCAGATGCAG
ATGCGGACATGGATGGAAGA
ATCAACAAAGAAGAGTGGAGGGACTTTGTTATCCAACGACCCCAACTCTTGAAGAAC
ATGACCCTTCCATCTCTTAGGTA
CAATTTTCTTTTGACTGACATTCAGTCATCGGTTGAAGACTTTGATATTTGTTAATGTGT
CTAGTTAAAAGTTACATTTT
CTAGAGAAACTTTTTTTTCGTTTCATAAGGGTTTGTTTTAGTTTGAAGATTTTTAGTCTT
TTTGTCCATTTGCTAGGGAT
GTGACGACAGCCTTCCCAAGTTTTATATTCAACACAGGAGTAGATGATTGA
>CsCML35
ATGGATCCAGCTGAGCTACGCCGTGTTTTCCAAATGTTTGATCGCAATGGCGATGGCAA
GATCACAAAGCAAGAGCTTGC
AAAATCTCTAGAAAACCTTGGAATATACATCCCGGATGATGACCTAGCCCAAATGATCG
AGAAGATTGATGTCAACAAGG
ACGGCTTTGTGGACATGGAGGAATTTGGTGAGCTTTATCAAACAATATTGGGTGAGAG
GGACGAGGAAGAGGATATGAGA
GAGGCGTTCAATGTGTTTGATCAAAATAGGGACGGTTTTATCACCGTGGAAGAGCTTA
GGTCTGTTTTGAGCTCGCTTGG
GTTAAGGCAAGGCCGGTCTATCGAGGAATGTAGGCTCATGATAAAGAAGGTGGACGAA
GATGGTGATGGAATGGTTAACT

ACAAAGAGTTCAAGCAAATGATGAAAGCAGGTGGTTTTGCAGGCTTGGAAACTTAA
>CsCML36
ATGTCAGGCTACCCTCAAAACCCCTCAGGCTACGGCACTCCACCCGCCCAACCTTACG
GTGCCCCACCCCAACAACCTTA
CGGTCAACCCCCTCAACAATCCTACGGTCAACCCCCACCAGCCCAACCCTACGGCGCA
CCACCTGCCCAACCTTACGGTG
CACCTGCACAGCCCTACGGTGCACCCGCACAGCCTTACGGTGCTCCCTCAGCCCCATA
CGGCCAAAACCCCAACAAACCT
CCTAAAGAAAACAAACCACAGGGAAGTGGTGGGTACGGTGCTGCTCCTCCACCTGGT
GGCGCGTACGGCCAGGGAGGGGC
GCCGTACGGGAGTCCGTTCGCGGCGTTGTTGCCGTCGACGTTCCCGCCAGGGACTGAT
CCGAACGTGGTGGCGTGTTTTC
AGGTGGCGGATCAGGATGGGAGTGGTGTGATTGATGATAAGGAGCTGCAGAGGGCTTT
GAGTAGCTATAATCAGAGCTTT
AGTCTCCGTACTGTTCATTTGCTCATGTATCTTTTCACCAATACTAACACCAGGAAGATC
GGTAACTATCTATTCTTTTT
TACTTTTTACTATTTCTCTCTCACATGGTAGTAAGGTAATTAATTAATTACACGCGTTGGG
GTAAAAGTGAAACACCACC
GCGTATATATTTAGTACAAGTCGTTTTAATTGGACGTAAAGCATAAATGCTAAAACGCGT
GGGGATGGGGCCAAACTGGT
TTTCATCCACATTTAAGAAAACGTGATATTAAATTATTAAGATTTTCTTTTTTTTAATCTG
CGTGTCAAGTTAATTACCA
AAATTAGCGTTATGCAGTGAAAATATAAAATCTATGGTTGTGATGTGGGCCCTGTATTAG
ATGAACCACTTTACTACAAG
TGTGTGACGTGTGGGTTTGAATTTAGCCTCAAATATCTGATGACTATTACACGACAAAA
TTTTAACTTATTTTGGTAGTA
TTGTTTAAATAATTAAATATAGTAAGCAGATATTGGAGGCTAGCAAGTCATAGCAGATTT
TGGATCAGTTAAAATAACTA
GAGTGAAACTATCTTGGACTTTACTTAGCAATGGGAACTTTTGACTTATTTCGTACTTTG
ATTATAACCATGAAGACACT
TGTGTAATAATGTCATTTGCTTTTGAAATCTTTTCAAGAAGGCATTATTTGGATGGTCAA
TTGGTTCTGACCCAGCTCAT
TATCAACGTGTCATTTACTCATTTTGGCCCGTTACAGTCCATAGTGCATTCTTTAAGAAA
AGATTGGACGTAATGACAGA
CTTTTGAATCATTTGAAATGCCATCTATATTTTATTATTTTATCATGACTACGGGTTTTTAT
CTTTCTAAAATATATTTA
TAAGTATGGAACTGTTGATGCAATCTATATATTACACGATGGTTAAATGACAAGTAAATA
TGACCGTGTAAGTTATGGTA
ATGGCTAGTTTCTGTATTGACTTCTTGTTGTTCTTTATGCTATCAGGACCCAAGGAGTTC
ATTCAAGTTTTCTACAGCTT
GCAGAACTGGAGGGTGAGTCTCAACAGGAATTTCCTCATTATTGTTTTGAGTGTTCTAC
CCATTATTTTATAGATCAGTC
GTTATTAGGTTATGGTTTACCTTTAAACCCGATTAAATTAAAGAGTTTAGCTAAATAAGT
TATGGGAGAATTCAGAATTG

TGTCAAAAGTGGTTGAAACTTGAAAGTAGCCTTTTCTAAATTGTTTTTATTTTTCATTAT
AAAAGTTATTATCATAATAA
CATCCTTCTTATAACCATATTTAACAGATTATATAGTTATGGAAAATGACCAAAATATGTC
GTGGGTCAAATCATACTGA
CCCAATTAGTAACCTGTAATAAGAACGGCTTTTTCTAACCTAATTTTTTACAGTGACAAC
GTGACAAACTGATGGACATG
GTATTTCTTGTACGTTTTACAGGCAAACTTTGAGAAATTTGACAGAGATCGCAGTGGCA
AGATTGATATCAATGAATTGC
GAGAGGCCCTCATGAGCCTTGGCTTTGCAGTTTCACCTGTGGTTTTGGATTTGCTAGTG
TCCAAGTTTGACAAGAGTGGT
GGAAAAAACAAGGCTATTGAATATGACAACTTCATCGAGTATGTTTCTGGAAACAGATT
ATCTATATATATCCTTATACA
TTTGCAATTTTTTTGGTTAGTTGTCTAATAAATTTCACGTTTTGTGATTTCAGGTGCTGC
TTGACTGTTAAGGTATGTAA
CAAATGTTTATGTTAAAAACAACTGCTTTGTTTGGTCAAACATAGCATGCTAAGCTCGA
CTACTACATTCGTTGTCGTCT
TTTCTCCTTTATATATGTGATTTTTTTATCAAGTATTCTTTAAAAATTTAGCAGTTTGGCA
AAGTTTTGAAATTGTAAAG
ATGGTTGAGTGTAAAATTCCAAGTGTCCTTACAGTCGTCTATGAGTTGCAGAATAGCAG
AGTGGAGGATTAAAGGATTGG
TTCGGTAAAGGGTCAATATGGGATAATATTTAATAGAGTTGCGATCAGGTCATGGTTGTA
AATAATTAATGTGTTGCAAA
ACTAACCTCGGAAACTTTAAGCTTCACCCACTTGGCTAAAATAACAACTCGACCCTTAT
GCACTATAATGAATTATTGAA
ATGTATGCTAAACAGCTACGAAACATTATTCTCAAGGTGTGTTTTTTGGTGATTGCAGG
GCCTAACAGAAAAGTTCAAGG
AGAAGGACACATCGTACTCAGGGAATGCTACCTTTACGTATGAGGCGTTCATGTTGACT
GTTTTGCCCTTCCTCATTGCT
TAG
>CsCML37
ATGAAGCTCTCCGGTAGAATCAACCCAAAAAACATCTTCCGATCCAAAAGCCACAAAA
AAGACTCCGTCTCCAGATCCGA
ATCCTCTTCATTCAGCTCATCCATTACGACGTCGTCTGGTTCACCAGAACGATCAAAAG
GGGCCACAACACCAACAACTG
TCTTACCAACACAACCCTTAACAAGATCTGACTTAGAAGCTTTGCTGCGCCGTATAACC
AATGATGAAGCTGAAGTTAAG
TTAATGCTTGATGAAGTCGAAGGTGACGGTGAAGTTGAGGGAACGATGACGGAGTTT
GGTGAAGAAGAGATGAGAGGCGC
CTTTGAGTTTTTTGACAGTGATGGCGACGGAATGATAACGGCGGATGAGCTGTTTCAG
GTTTTTAAAGTGATTAATGGTG
ATGATGGGTGTACGTTAGAAGAGTGTAAGCGTATGATAGCAAATGTTGATTTGAATGGT
GATGGGTTTGTTTGTTTTCAA
GATTTTGCCCGTATGATGGAGCAACGTCATATCTGAACCGAGAAATGATATGAGTACTA
AATTTAAGTTTAATCTTGTGA

CTAAAATATGTGTGGCACAACACATACTTTAGTAATGAAATTAGGCATGAATTATACTAG
TAAAATTTAATCGTATGGCA
TGTGAATTTTAGCAGACATGAAAACAGTTATTCGTGTATTATTAGATTCTCTGAAAATAT
GGAGTTTAATATTGAATCAA
AAGACAAATATTATATTCTTTGAATTTATTTGTGTTGTTTTGAGTCTTTTATTATTTTTATT
ATTGTGGGAATATCGTGT
GGGTGGTGTTGTGTCCGTTGGTTGTACGTTAGGTTGGAAATATGGACGCTTTTGATTAT
GGGGTTGATATTCACATAAAA
ATAAGACTAAACTCCAAAATACATTGTTTACAGTTTCTGAGACCTTACATCTTTTTCAGA
AATTATTGGCTAGTTACATC
ATTCGTTAGCCAAACGTTAGCCATTCTACATATTTGGTCACTAACTCTGTTAGTTTTCTT
CGTTAACTGCTAACGGGAGT
ATAAAGGAGGGCTGTTTTGGTCTTTTTACTCATATAAATGATATATTTTGCAGTTTACTCT
TTTACATATAAGAGGGATG
TATTTTGCAATTTACTTTTATACTTTACTTTTTTTTTAATTATTATTATTATTCATTCATATTT
TATTTTCTAACATTTT
CATTCGTTTTTTCTATTTTTATTTGTTTTTGCCGGTTTTCATTCATTTTTGTTTGTTTTTTT
ACATTTTTGTCCGTTTTT
TTTATATTTTGTCTGTTTTTCTTACGTTTTTGTATTATTTCAAAATTTAAAAAAAAAATCA
AAAAAAAAATTTTTTGAAA
CAAATTTTCTAAAAAAAAAAATTGTAATTTTTTCGCAAATTTCAAAAAAAATTTCCAAA
AAAATTTTGAAAAAAAAAATT
CTATTTTTTTTTTCGTAATTTTTTTTTTTGAAAAAAAAATTTCGAAAAAAACAAATTTAG
AAAAAAAAAATTCCAACTTT
TTTTTCGGAAAATGTTTTTTCAAAATTTTTCTGGAATTTTTTTTCAAATTTGCGTAAAA
ATTCCAATTTTTTTTTTGGA
AATCTTTTTTCAAAATTTTTTTTTTGAAAAAAATTTTCAATATGTTAAAATAGTACAAA
AACAAACAAAAATGTATAAA
AACATAAGAAAACAGGAAAAACGAAAAAAAAAGTACAAAACAGAAGAAAACGAAA
AAAAAGGGCAAAAACAAGAAAAACA
TATGAAAGCATATAAAAACTGATGAAAATGAATAAAAATGTACAAAAAAACTGAAAAC
AGGAAAAAAAAAATGGACGAAA
CGAAAAAACATACGAAAATGTATAAAAAAAACGGACGAAAACGAATGAAAACAAAAT
ATGAGTGAATAATAATAATAATA
ATAATAATAATAATAATAATAATAATTAGAAAAAAAAGTAAAGTATAAAAGTAAATTGCA
AAATACATCCCTTTTATATG
TAAAAGAGTAAACTGCAAAATACATCCCTTATATGAGTAAAAAGACCAAAACAACCCT
TCTCTATACTCCTGTTAGCAGT
TAACGAAGAAAACTAACAGAGTTAGTGACTAAAGATGTAGAATGACCAACGTTTGGTT
AACGAAAGATGTAACTAGCCAA
TAGTTTCTGACAGGGATGTAAGGTCTCAGAAACTGTAAACCACAGGGATGTATTTTGTA
GTTTACTCTAAAAATAATTTG
ATTCTCATATCATAATTATGCATATATTATGTTGTAACGTGTGCAATATTTATGTGATATTGT
GATGTATGAATCATTTT

TTTTGTGTGATTATCAACTCCCTTTTGATTAGAGCAAAGGCTAAATAAACTCTTGACGTT
TTCATTTTGCACCCTCGATA
GGTAGATGGAAAGATGGATTTGACTAGCTACTACTATATTTATAATATGTAAGTTCATAT
ATGAAAATGTTTTTAACAA
GTGGCTTCACGGCATCAGTAGTTAAGTGTATTAATTTAATATAATTTTTTTTTTAAATTA
AAAATTACATTTAATGCAC
ATCAGTAAATGTTATAAACAAGGTGGCAAGCAAAAGTAATTTTTTAAATGTTGGGGAA
AGTGCCGTCGGGCAATGTTTTA
TCTACCTATACTATAAGGAAGAAGCCTCAGGTTCTTCCAAAAAAATGCCACATCAGCATT
GAACAGTTGCACACTGTTTTA
TCTTTGAAAGTTTTGGTTCCTTATCTACCTAATATATGTTAGAAGTCATTTTATTTCACTC
ACACCGTATCTCTCTGTCA
ACTCTCAAGTTTTAACCTCTGTCTTGCCTGGTAGCCATATTCCGGCGACACCATTTTACA
ACCATGATAACATCATCTTT
TACATGTCTGACCACCACTGATTTTTCCGGCCAGCCACCATACATATCCAGTCATTGCTT
TCATCTTCTAATCTTCTACA
AATATCTATATTCAGGTTTGTTTAGATTTCATCATCCTATTATTAGAACTACTGACAAATC
ATATTAAAGGATCATTTGT
TGTTATTCTAAACTTTTGTTGCTGATTACGGCAGATGTATACGGGTCATTCGACATGTGA
AAGGTATCTATTTTTTCATG
CTTTTTGGGTTTGTTTTTATAGGTATGATTTTTTTTGGTTAATATATGGGCTCAGTTTTGTA
ATTTTTTTTAGGTGCTTT
TTCATATAGTGGTGCTATGAACTTGAGCATCATCTTTTTTCCTTCTAATGTACTAACTTGA
GCATCATCTTTATTTCTTT
TATGACAGTTCTTAAAAAAAAAAAAAAAAAAAAAAACTACGGTTATAATCTTTTGTTGTTG
TGTTTCACTACATCTATTGTT
ATTGTTTAGATATTTTTAGTTAAATTTTTTATGACATATTTTTTATTGATGGTATTTATTTTT
CATGATATAGGTACTTT
GAAAAGAGAAAGAGAGTGA
>CsCML38
GGGGTTTTATGGAGAATCCAGGAGAGACAAATCGAGAGGTCGTCATCATGGAGTTACT
CAGCAAGTAAAGCAAGAGATGA
AGGAAGCATTCGAGCTATTTGATACTGATGGCAATGGTATGACATGAACCCAATGAACT
CTTGTTATTTGAGTGAAAATG
ATTTTAACTTGTGTGGATTCTTACAAATATAGGTACCATTGATGCTAAGGAGTTGAGCAA
TGCAATGAGGTACGAGCATC
ATTTAAATGGAATATCTTATGATATAGGATTATGCATACAATGATACATCGTTATTATTTCA
TTTCAGGGCCCTTGGTTT
CGAAATGACAAAGGAGGTAACATCCAGCCGCCAGTTATTTTCCTTCTTGTTCTTAATTT
ATTATAGTGTTTAAAGGCTGT
CTGGAATTACATATTGAAACTAATGTTGGGTTTATTCAACTGTTCTTCAGCAACTTGATC
AGATGATAGCGGATGTAGAC
AGAGATGGCAGTGGTGCAATTGATTTTGATGAATTTGTGTACATGATGAGTGATAAAAT
TGGGGAAAGGAGCAACAAACA

GGAGCTTACAAAAGCATTCAACATTATTGATCATGATAAAAATGTAACCTTCTGTATCAA
TGAAACTTTGTCTTACATTA
GAAACTAATGTTACTCTGGTGGAAAACAGGGAAAGATATCAATTTTGGACATCAAGAA
TATTGCTAAGGAGTTGAGTGTA
CGCTTTACCGATGCAGAAATTCACGCAATGGTGGAGGAAGCAGATCGTGATGGTAAGT
TCATGTAGTTTTCGCTACATGC
AACTTTACGTTTACTAGAGGTGATAAAATGGACATGTCAAATTGGGTAAATTAAAGTAC
AGGTCGGTCAACTCGCAACCC
TTTTTGGCTTTACAATTGATTGATGTTAACTAATATGATTATAGAGTTCTTGTATTGTGTT
AAGGAGTAACTTTTGACCC
ATTTGACCTGTAAGAATAAAGATATACTTCACATTGACCCATGTCCTGTTTAACCATTA
GCTGTCCTCTCAACCAAACA
ACAGAATAGAACTTGTGGTTTCAATATGTGTGTTCTGTTTACCAATATTCAATATGTTATT
TATGTTGATCAGATGATGG
AGAAGTTAGCAAAGAGGAGTTCATGAGAATGATGCAGACAACTTCTTATGGATATTAG
>CsCML39
GGGGGTTTACGGAGAAGCGTCCAGGAGAGACAAGCCCAGAGGGCGTCATCATGGGTT
GACTCAGCAGAAGAGGCAGGAGA
TCAAAGAAGCATTTGAGCTATTTGATACGGATGGCTCTGGTATGTTTGTTAATATTAAGG
CTTTGGTTTGGGGGTGTTTA
AGTGGATGTGTGATATTGATAATAAGTTGTAATAAAAATGGGCTGTAAAAACAATGTTT
GAATATGCTTATGTTGTTTGA
CGGTGTAATAGTAAGATAATCCGTTGTTTGAGTGATTGACAAGCTCTGATTATTCAAGA
CATTTTTTAAGGATAATGACA
AACAAGACTTGTTGTAAAATGAATGGAGATGGTTTGACATTTTTTAAGAAGGTGTAAAT
AAGTAATTTTGTTTTGCCCAA
ATGATTATTTTTGTTGTAGCATGTATGAAACTGATAACGTAGAATGATCAGAAGCAGAAT
CTGTTTATCCAAACATTAAA
AACTGAATCAGTTCGAAATGCAAATTCAAACACTTTTAATCTGTGGGATTTCAACTTGT
CTGATCTGAATCATTAACTAC
AGGCACCATTGATGCTAAGGAGTTAAATGTTGCGATGAGGTATGACCTTCAATCCTACC
TTACCATGAAAATCTTATATG
GGATTATGAGGTATTGAATTATGCTTACATTGATACCTTAATTTGATTTCATTTCAGGGCC
TTGGGTTTTGAAATGACGG
AAGAGGTAATATCTAACTGCCAATGGTTTTTTGTTTTTTTTAGAAAATACCGTCTTCATT
TATTAAAATCATTCCTACCA
GCTATCTTCTTTATTCTCTTTTCCCATGGAACTTGTGCCCCAATGATTTTCTAAACGACA
AAATTCTCTAGCAGACAGTA
TTAATTAATGGCTCTGCTTCCACCAGATTCCTCCAAAATATTGAACATATCTGAATCAAA
GAAAAGTGGTTGCCCCTTGT
TTTCCTTTTCTTAGCTTACTGAATCTTGTTTTGCTGAAAAAAGTTGCTCCTACGTGTAGC
TCGTTTTTGTTGCTATTCAA
CTGCAAGTCATTCCTTCGGATTTCATCTATTACACGTGTGAATGTCCTGCCTTCTTTTTTT
CAGCAAATCAATCAAATGA

TAGCAGACGTAGACAAAGATGGGAGCGGTGCAATTGATTTTGATGAATTTGCATACATG
ATGACTGCCAAAATTGGTGAG
AGGGACAGCAAACAAGAGCTTACAAAAGCATTCGAAATCATTGACCAAGATAAAAAT
GTAACCTTCTATCAGTTTTAAAA
TTAGTGACCATTGTTGTTATTTTACGTCAGAAACTAAATTTACATTGTTGCACTACAGGG
GAAGATATCCGTTGCTGACA
TTAAGAAAATAGCTAAGGAGTTGGGTGAACATTTTACCGATGATGAGATTCACGAGATG
GTCGAGGAAGCAGATCGTGAC
CGTGAGTTTCTCTAATCTTTCTGACTGCATCGTATGATTAACTGAAATTTAACTTTCAAC
TTGGGGTTTTTCTCTTTCCA
CCAATTACCAAGAAACCCTTTAAAATGCGTGGGGCTTTTGTGTTTGTGTGATGTTGCGT
TGCATTAAATCTTCAATGTTA
TGTTTGGTATCTGATTTGTTTGGGCTTAATGGTTGCATTTCTTTGCATTGGACAGATGAT
GGTGAAGTCAGTGCAGAAGA
GTTTATGAGAATGATGAAGAGAACTTCATATGGATATTAA
>CsCML40
ATGATCTATAATTATTATAACAAATCGTTCGAAAAACTATCATTTTTTCCTTCGAAACAAC
GTCTATTCTAAGATGGTGA
CATATTTTGAAAGTAGTAGTGACTGGTGAGGATTTTAGAAGGTATGGTGGGTTTTGAAT
AAATCGACGAACCCTAATCGG
AACCAAAGAGGGATATGCGGTTTACATGATTTTTTTATTTTTTTTTTCAGATCTATCCG
GTTCTTTCTTGCTTTTCTC
AACAAGGAGTGATATATTCGAACCTCCAGTTAATGATGGTGAGCCAGATGGAGTTGATA
AAATTAACTGATTTTTTATTT
TCTTTTTGCTATTGATATGGGCATTTTTAGTGATTTCTTTATTAGTCTTTGTTTATCTTTTT
TGCATAGCTAAGATGCAA
ACAGGTGTGATAGAGTGGTTGAATAGAAGTCTCCAAATCTTATTTGTTTGTTAATGCTTT
GGATGAAGAGTTTAGAATTC
TTTTAGTTGATGGTTCTATTCTTGGTTGATTCGATTTTGATCCAAGCCTTTTCAATTTTTC
GTAGATTCGGTTCTGGTTT
TTCATTCTTTAAACACTACACGTTTTTGTATAAATAATTAGTAATTACTTATTTTTGAATC
AATAGTTACACAAACAAAT
CGGTGTATCAATCTATTTGGAATTAGATCTGATATTTTTCTCGTTTCGGGTTGTGTATCAA
GCGGTTTTTGTTGACAACA
ATTGATGAATTCAAATGGGATTATTTTTATGAATGTATTTTTGTTTGTTGATTATTCATTTG
TATGTTATAAGAGTTGCT
CCTAGTTCTAATATTAATTAGTTAATTATGTATTTGTTTGTGTATTACATGGTTATAAATATA
GTGTCGCATTATTGATG
AAGCAGACAGAAGCCTTCTGACAAAGATGACAAAGTCAACAAACATATGCCATTACCA
AAACTACGTAGCATTTGTCTAT
AAAGAATATGTTATTTAATTATGAAACTAAATATTTCAAAAGAACTCATTCGTCACTAAA
AATATAAATATGTCATAATA
ATTAGTGTGTTAGTACAAACGTATTTAAAAAAAAGTTGTGTGTCATTATATATTTGTCAA
GAAAATTTGTGTGATAAATA

TAGCGTGTAATTACAAATGTATTAAAATTTTGTAGCGTGTAATTGAAAGTGACTAAAATT
TTAATGAGTCACTATAAATG
TATCAAAAAATTTAGTGTCACTACATATATACAAAAAATTTAGTTTGTCACTATATACTAT
ATATGTATCAAAAAATTTA
GTATTACATTACAATTTGTCAAAAAAAAAAAAAAAAAAAAAAAAAAAAAAAAAAAAAAAA
AAAAAAACTAGTGTGTCAATGCA
TTAGAAAGTGTGCACCAATATTTTAATTATTTCTTTATCGTGTAATAAATTGTGTATCATA
CGGAGTATGTTTTAGTTAA
CAAATTATGTATGGCCAATTAGAGCTTTTGAAGTCGTGTATTTTCTTTTAAATACAAAAG
TATTTATTACAGTTAGATGT
GTTATGTATCACTATTTAAAATGAAAAAATGGAATTACAAACTCTAGCTATACGTAAATC
TTAAAGGTGCATGCAATAAA
ATATTTATATTTTGGTTAGAAATTTAATTAAGCATATAATTTTTGAAATTAAATAAAATATA
CTGAATTTTGAAAAAAAA
AAAAAAAAACTGGGTCAACATTTATGTCTATTTCTCCATTCAAATTAGTTTTATTAAATT
GGAAGTTGGTACATAATACC
ATTAGTTTTTTAGTTTATTACAATTAGGCCATTACATTAATTAAATTTGATTATGATCTTTA
GTGGTTGATCCAATGGAC
CACAAGAGTTCTTATTGTTCTTATTATAAGGTGGTCATCACAATAACGTTACCCTATATAT
ATATATATATATATATATA
TATATATATATATATATATATATATATATATATATATATATATATATATATATATATATATATAT
ATATATAATTG
TTACAAGTGTCATAACTACTATTCATATCTAGCTACATTAGATTAAAATTTGATACAAAA
GATTTTTAAATCTTAAATAG
AAACGTATTCACCCCTCCTTTTTACGTTTATATATATAGAGAGAGAGAGTAAATTACACC
GTTCGTCCTTGTGGTTTGCG
TTGACCGCAACCTTTCGTCTTTTGACCCAAAAAAATGCGCCATTCGTTCTTAAGTGGCA
AACGTTATGCGCCGTTCGTCC
CTCCATCCAACTGGCCCCACTAACTCCCACTAACGCCCGTTAACTAAGGTTTTTAAAGG
GGTATATACGTCATTTCCCTA
GCAACTAGCATTTATTACCCTCATCTACCCCATCAGTTGTGATTAATTCCCAAAGTGTAA
AACTTTTGCATACACTAACC
CAAGTTTTCATCTTCTTCTTCCCCCATTTAAGTGTGAATCTTAACCCAGTAATATTGAATT
GATACTTCGCAAGATTCAG
TTTTTTTACTTTCTAACTACACCTATCTTTTTTTAATCACTAATAAATAAATAAATAAACA
AAATATCTCCCTACTTTTT
ACCAGATTCCTTATTTTCTTCAAACTAAAAAATCAAAAACACACCACATACACAAAAA
ACATCATCATCTTCAATAAATA
TAACACAACAACAACCCAAGAAACAAAACACACCACATACCCAGTTTTCCTTTTTATT
ATTTCCTGAAACCTAATTTCAC
ATTTCATTTTTACAGTGAAACATTAATTTTGAAAGCTTTACACTAAAAACCGAAAGATC
TAATTTTGAAATTATACCAAT
TTTTAAAGTAAAAATTACATAAATCGTCCAAAGTGGTATATCACCACTTACCACACACA
CACAAACATAAAACACACATC

ATCATCATCACCATTTCTCTCTCTCTCTTTAACCTAGCCGCCGACCACCTTAAACCACCA
CCACCGCCACACCGCCGCTA
CCGGTTCTCTTTTTGTGTTGTAAGCAAAGGTAGGTTAAAACCCACGAGTTTTGACTCGT
GTTTCTTGGTAACTCGTTGAT
GAGTCACGAGATGACTCGGTCACATGTCGGGTTTGATATTAAAAACATGGGAAATGATT
TCTTGATAATAAAATGTGGTA
TAAAACTTGTTGGTATCCGCGTTTAAGTCGTGCATCACGACACCACAGTCACCATCTCA
TCACCAACATGTGCAGCCGCT
GCCTTCCCTTCCTTTGGTCAAGAAACAGTGTGGGTTTTGGTTCGTGCCTTCATGGTTCG
TGATTATGATCGTGGGTTCAT
GTTTGTTTCGTTTTTATTCATCTCCTGCATCTCCTTCTCCTCGCGTTTTAGTGGTTATCAT
CAAGAGAGAGGTGAGTGAT
AGGTGTTGATGGCTGTGTTGGTTGGTCGGAGGTGATGGTGAACAGACGGCGGAAGAG
AGAGCAGGAGAGTGGTGGGTGGT
GGCTGAGATGTGGAGAGGTTTTAAGATGAAGAGAAGAAGATGATGTTTTGTGTTTATG
TGGAAGAGTATTGACCACAAAC
TATATAAGGTGGAGATGAAATGACATTTTTACCCTCATGTGCAAGTCACATGAGGGGAG
TTTAACAGTCAAACTTAACGT
CGTTTGGTCAAGGGACGAACGGCGCATAACGTTTGCCATTTAAAGACGAACGGCGCAT
TTTTTTTGGTCAAAGGACGAAA
GGCTGCGGTCAACACAAACCACAAGGACGAACGGTGTAATTTACTCATATATATATATAT
ATAAAGTAGGGTAATTAGTT
GATTAGTTGGAGAGTTTAAAATACAATATATATGTAAAAAAAAAAAAATTATTAAAACA
AGCATAGAATACCATATTCAA
ACAAGTATACATGTCTCCTTGTTTTAAAAAACAAGGGGAATAGACTCTGTCAACTTAAA
TCTTTTGACTTTCCGAGTTTC
CATTACATCTTACAATTCAGATCGTCCTTCACAAACTCGTTCAACGCGCACTTTCTATAG
AGGAAAAGGAGAAGCTGTTA
TTTTTATAAAGAATTCGTTGCAAAAAATGGTGAGTCGTCTTCTTAATTTCACATGCAATT
TTATCTCAAATATATATATA
CCTTTCCCCTTGTATTTGTATGTTATGCTTGTTTGTTATCTGCGTTATTTCATAAAGGTTTA
TCAACTTTATGGGTTGAC
CAGTTTTTTAAGTTTGCTTTGATCATGTTTTGTTTTTGTATGGTTGACCATAAAAATTTTG
ATGACGACGGGAGGTTTGA
AGAATTATTGTTTTTGTAACTCTGGTTCTGACCATTTGACCGACTAAATCACGGGATTTA
AACTCATCACCACACAGACT
CAAACCAGGTAAAGCACTCCATTTCTCAGAACGTTGGCCTAGCCACTTCAGGATTGTT
TGCGCCTAGCAGGAACATACTA
AACTAGAGATGTCTCACATTTTAGTGATATCGGTCACAAACCTTTTGCCACTAGGCCAC
CATGAGTGTTTTTTTCTGTAG
AATGTTGTTAAATAACTATTACTGATGTTTTGTGATGATTTAGGGAATAAGAAGGTTTAG
GCTTTGTTGATTAGTAATAT
ATATTAATATTATTGGATAGTTTAAACAATCCACATATATCGATCGCACAGGCTTCTGTTT
GAATTTGAAATTTGCACTA

TCGTAACATAGATGCAAATAATGTGCCAGCAAAGTAGATATGTAATAATCCCTTAACGG
CACTTGAACCTCCCAGTTATA

CCAGTTGCATATAATCTTGTGACATATCCCTAGAGTTAGATAGCCGATAGTAGTGGAAGC
GCTAAGGCCGCTATTCCTGT

TATCAACCAGCAATCACTTTATCACACCCAATGACCCCGATCACTTTTACTAAATTGCAA
TATTGGTTGTATAGTAGCTA

CAATATACGTAATTGACACAATTCTCATCGACACATAGATTGCATAAACTGTATTTGCAT
AAACTGAATTTGCATAAACT

GAATTATGGATACAAGAAGTAACTTATGCAAAAAGTACCTTGTGCAAATATGTTGACAA
TGGTCTGGAAAGAATTTAAAT

AAAAAGTAGAGGTGGTGGAAGTTTGTAATTTTTATTTTTTTTTCATAACTGCGATTCGAT
CAGTTTCATTTCGTGTTAAA

CTGATGAACAAAAAAAAGGTAATTAAGGGACGTGGATGTGAAACTTGAAAACAGAAAG
TGATTGCAAATAAGTAATCATGG

ATTTACTAACCTGATTAATTCAATCATTCGAAATGTATCCCTTTGTTGTGAAATGACAAG
AAGTTTTTTTCTTTTTTGGG

TGTGTTTATCAACGACAGTTGTCATCACCTCTTTCCGGAATAGACTTTGCAGGAAATGG
ATACTTTTTCTTTGATCCTCT

CTCTTATCAGCTAAAAAAATTTGTTCTCACCTTTTTTATACTTTTTGCAACTCATGATTTC
TTTGTATTTGGATTTTAGG

GAGTTCGCAGAGAATCATCAAGAAGCGACCAGCCCAGAGGACGTCATCATGGGTTAA
ATCCGCAGAAAAAGCAAGAGATC

AATGAAGCATTTGACCTATTTGATACTGATGGATCTGGTATGTTGCTATCCTATAAACCG
GGGGTGTTTGGATGTGCATT

TTTAAAGTGGTTATGCCAACAGTATAAAACGGACACTCAAAACTGATTATATTGAAATA
ATTAGACAATCAGAAGCAGAA

TCTGTTTATGCCAACAGTAAAAGTTCCCAAATGCAAATCCATGGATCCCATTATGTTACT
AGAGTGATTTGACATTTTGA

CTTGTGTGGTATATTCACTTATAATTGTAGGTACCATTGATGCCAAGGAGTTAAATGTTG
CAATGAGGTATGAGCTTCTT

TTCACTTAACAAAATTCCTATGACATTGGATTATGCAAACACTGATATCTTAAATTGATT
TCATTTCAGGGCCTTGGGAT

TTGAAATGACAGAGGAGGTACCATCTAATTGCCAATGATTTACCTTTTATTTTTTATTTTT
GGGGCTTCAGTTCTTTCTA

AATTATCTTCACTTGTTACTATGATTCCTACCATTTATCTTAGGGCCCTTTTTTTACTGTG
CCTCATTGCTTTCCTACAT

GACAAAATTATCAGATACAATAAATTTATTGTCTTAATTGTTATCTATTTGACATCTTCGA
ACATGTGTTTTAAAGAAAA

ATCTTCCCCTTGTTTTTCATAGCTAAGCTTTATGAATCCTGTGATATTAAAGTTTTGTCAT
ATTTGTATAACTCGTTTTG

TTATATATTACACATGTGTAAATTTCCCATATTCTTTTGTTTCTCAGCAAATTAATCAAAT
GATAGCGGATGTAGACAAG

GATGGCAGTGGTGCAATTGATCGTGATGAATTTGAGTACATGATGACTGCCAAAATTGG
TGAAAGGGACAGCAAACAAGA

GCTTAAAAAAGCATTTGAAATCATTGACCAAGATAAAAATGTAATCGTCTAATACACTG
ACCACCTTATAGTCTTTTAAA
TTTTAGAAACTAATGTTTCTCTGGTAAATTACAGGGCAAGATATCTTTTGCAGACATTAA
GAAAATTGCTAAGGAGTTGG
GTGAACATTTTACTGATGCAGAGATCCACGAGATGGTCGAGGAAGCCGATTGTGATGA
TCGTAAGTTTGTTTGTATATAT
TCTCTTTATTGCATATATTGAATCAATTTGTAGTTTGAGTTTTGGGGTTATTTTTTCAACC
AGTTACCGATATACCTTTA
AAACCCATTGGGCTTGTGTTTGAGTCATGTGCTTTTTATATCGCGTTTGACAGGTGATG
GTGAAGTCAGTTCAGAGGAGT
TCATGAAAATGATGAAGAGAACTTCTTATGGATATTAG
>CsCML41
ATGTCGAAAATGAGTTTTCTTGACATCCAATACAACATCTCTAAGCGTAAGTTCCTTAG
GAAACCATCTAGGATGTTCTC
TAGTAGCGAAAGACAACCTTCGGGCCTGCCTATGTTCCAACCAAATGTGAACGAGATG
AGGCGTGTTTTTGACAAGTTTG
ATCGTGATAAAGATGGCAAGATATCAAGAGGCGAATACAAGGCTATTCTTAGAGCACTG
AAACAAGGAGGTACAGAAAGA
GACATCCAAAAGATATTTGAGGTGGCGGATTTGGATGGAGATGGATTTATTGATTTCAA
GGAGTTTATGGAGGTGCAAAA
GAAAGGTGGTGCAGTTAAAGCGGTAGATGTGCAAAGTGCGTTTAAGACGTTTGATCTC
GATGGAGATGGGAAAATAAGCG
TCGAGGAAGTGTATGAGTTGATGAAGAGGCTTGGGGAGAGGTGTAGCTTGCAAGATTG
TCGAAAAATGGTGAGAGGTGTG
GATTCGAACCAAGATGGTGTGATTGATATTGATGAGTTTATGACGATGATGACACAAAA
CATGAAGATTTAA
>CsCML42
ATGAGCAATTCAAGTGAGAGAAAAGCTGAGCTAAAAAGTGTTTTTGCCACCTTTGACA
AGAACAAAGATGGGTTCATCAC
AAAACAAGAACTTAGTGATTCACTCAAGAATATAGGCATATCAACTAGTGAGAAAGAT
GTGGTAGAAATGGTTCAAAGGG
TTGATGTTAATGGTGATGGATTGATAGATTTTGATGAGTTTTGTGAGCTTTTTGAGTCCA
TGATGAGTAAAGAAGACATG
CAAGGGAGTAAAGTTGGTGATGATGATGATCATGAAGATGGTGATTTAAGAGATGCTTT
TAATGTATTTGATGGTGATAA
AAATGGGCTTATAAGTGTTGAAGAATTAGGGTTGGTTTTGGACTCATTGGGTTTTAAGG
AAGGGAAAAAGTTGGAAGATT
GTAAGAAGATGATTAGCAAAGTTGATATTGATGGTGATGGTATGATCAATTTTAATGAGT
TCAAGAGCATGATGAAAAGT
GGTGTTAGTCTTATTTCAGTTTCTTGA
>CsCML43
ATGTCTTCTCCTCCTAGCTACAACGATCTGTATCGCCTTTTCAAGAAGCTAGACCAAAA
TGGAGACGGTCTTGTCAGCCC
GCATGAGCTCCAATGGCTTCTTGATACCATGAAAGTGTCTTCAAGTGTCGATGACCTAA

GATACTTAACGGGTAAAACCA

ATATTAATTTTACTGAGTTCTTAGAGTTTTACGGTACCATTACAAAGGAAGAAAAGGTA

ACCTGTGATGATGAATCAGAA

AGTGACCTCTTCAAGGCGTTTGAGATGTTTGATAAGAACCGTGATGGATTCATTTGTAA

TGAGGAGCTAATGGAGGCGTT

AACAAGGTTGGGATTATGGGACGATAAGAGTAACATGGATGTTAAGAGTATGATCAAA

GCCTATGATGCCAATTGTGACG

GTTTTATTGATTTCCATGAGTTCAAGAAAATGATGGCTTAG

>CsCML44

ATGTCTCAGTGCTTAGAAGGGATCAAGCATCTATGCACTTCCCTACTTTCCTGTTGTGAT

CTTGAATTAACTAAACAATC

GCAGGGGCTCGATGACCCTGCAATTCTCGCTAGCCAGACAGTTTGTATGCATTTAGTCT

ACCATATTATGCTATATTTGT

GCTCATAACTTTACGGGTTAATACTTGTATCTTATAACTTTCAAATTTTTGGTGGTTTAGT

CCCGTTACTTTTTTGTGTC

AATTTTTCTAAGTTGCCGAAAAAAACCTTTGGAGAACGACATAAAAAGTCAAGGGACT

TGATGAGTGATTGAAGTTGGTG

TATTAAATCTAGTCGTTAAGGGAAACAAAACACTCGTAAAAACGTTTAAATCACCATTA

ATGAAAGTTTAGCGGTTTTAC

CATTATTAACCCTATATTTATTACTACTATTTTTATAAACATACAGAGATATCCTTATACAA

AGATTCATTTTGTTTAAT

GCAGTTAGTGTAAGTGAAATAGAAGCGCTTTACGAGCTATTTAAGAAGATTAGCAGTG

CAGTCAATGACGATGGGTTGAT

TAATAAGGTTTGTATTGAATCTGGAATTATCCTTATTTTGGTATGTTTATGCGAAATCGGT

TTTTAATTATTTGACTTCA

ATGCAGGAAAAGTTTCAGTTGGCATTGTTTAAGACCAATAAAAAAGAGAGCTTGTTTG

CTGATCGGGTATGGTTCAATTT

TAAGCATCTTTTGTTTTATGTAGTATATGATTTAGAAATGTTGTATCACGGTTATACTTAG

GTTGCTCGAATTCAGGTGT

TTGACTTATTCGACACCAAACACAATGGAATTTTGGGATTTGAGGAGTTTGCCCGTGCA

CTCTCGGTGTTTCATCCAAAC

GCCCCTATTGATGATAAGATCAACTGTAAGTCTACATAGTTTTCTCACGTTGTGTTTGTT

TGCTTCTTTAGTTCTCATCG

GTCATTGTCGATTTGCAGTTTCCTTTCAGCTTTACGATCTAAAGCAGCAAGGTTTCATTG

AGAGGCAAGAGGTAAGTTGT

GTACTATGCCTAAACATGTAAACATATACATGTACCTAGAACATTTAGTTTAATCGATTG

GATTAATGATTGTGTAGGTG

AAGCAAATGGTGGTAGCTACACTTGCTGAATCGGGGATGAATCTTTCAGACGATGTTAT

AGAGAGTATCATTGACAAGGT

ACTATTTCTTTTTTTTTTACTCTTTTAGGAAAAAAAATTTATCATTCTTATTGGAAACCCG

AGTGATAGTTGGTAACTTG

TTTATAGACCTTTGAGGAAGCTGATACGAAGCATGATGGTAAAATCGACCAAGAAGAA

TGGAGAAACCTCGTGCTACGAC

ATCCTTCTCTCTTGAAAAACATGACCCTGCAGTACCTCAAGTAAGTTCACTTTTCATAA

CAATTGTACACGATTTTTTA
GCCATACAACACCTAAAGAAGAGGTTAAAAAGATGGACGGTGAGATGGTCAAAATCC
TTAGTTGGTGTATGACTAAAAAA
AGATGTGCAACTTACCGCCTTTCTCAAAATGTTAGGAAGTTACCACGTACTAAGGTTAC
TTTTTCTGGTATTTGTTTACA
GGGACATTACGACTACATTCCCAAGCTTCGTTTTTCACTCGAGAGTCGAAGATATGTAA
>CsCML45
ATGGCTGCATCCAAGACACAACTAGAGTTTCATGATCATTTACCTTTGATAGAAGAAAA
GCTAGGCGGAGACGGTCTCAT
AGGTGAATTATGCAAAGGATTTGAGCTTATAATGGACCCGAATAAAGGGGTGATCACAT
TTGATAGCTTGAAGAATAACG
CGTCGTTTTTGGGCCTCCAAGACTTGAGTGATGATGATTTGATGAGCATGCTAAAGGAA
GGTGATTATGATGGTGATGGC
GCGTTAAATCAGATGGAATTTTGTGTTCTTATGTTTAGATTAAGTCCTGATTTGATGGAT
CAATCTGAGTTTTTGTTGGA
AGAAGCTCTTGAACAAGAGGTTAAGAATTCATATCAATAG
>CsCML46
ATGGTTCAGGTTTTGATTTTGCTATAAATGAATGAAGGACAATTAAGTAATTTACTAACT
TACGAATTTGAATTCATTGA
TATATTATCAGGTAACCAAACATGTTAAGAGATTTTGAAATAGAAATTCAAATACAATGG
CATTTGAAATTTCTTGGTAC
TTAAAGTCTCTTAATTTCAATTTACTTGAACCAAACGCCCAATTTAGGGTTATGTGGATC
GACCGAATTCTCTATGGGGA
TTAAGTTTGATAAGCTGTCAAATTTATATACTTAAAGTTAATTAAATGAAATCATAGTCCT
TCTAGGAATGTTCTATAGT
TTCCTATAAATACCAAAGAAATTCCGAAGGGATTCATAACAATCCTCTAAACTCTAACT
CCCTCTCTCGTTTGATCGACC
AACCGAAGGGTCGAAACCCTTTGGTTTGAACCCTAAATCAGCCAAAGCTGATTTTCAC
AAGTCGACTAAGCATAGTCGAC
TTGTTACTTGCTTTCGTACTCTCCTAATTCACATAGGTTCGTAGCCCTATTTATGCATGTG
CAATATTGATGGTTACCTA
ATGCAATCCAAGTAGTGATTCTTGCCCAAGAAACCCTAACATAATCGACTGAAACATTA
ATGAAAAAGAAGGCGATTACA
TGTTGATCAAGAAGGTTGTGGGGCTTACGAAACTGCAGGGACACAAGTCAGTAATCCT
AAACACCCTTTATATGTGTTAG
CAGGTTATATCACTGATTAGGAGATCTGTTGTTAGGGGTTTGGTCTTCATGGCCTTTATG
TATTTATTTTCTGACGTGTA
AGTTTTATAGGCCATACAGGTGACCACCAATACCCTACACTTATCAAGGGAGATACTCG
TATTAGATTAAGTAATAATCC
TATTTGCATATTCAGGAAGAGTTCCAGCTCGCACTATTCAGAAATAGAAACAAGCGTAA
TCTATTTGCGGACAGGGTATG
TTAATGCAATAAAAAATAACTATCAGGCATTTGCGGCTTCTTAAATACATCTTTTATTTTT
ACTAAAGAAAACAATGCCC
GGATTTGTTTTGTAGATATTCGATCTATTTGATTTGAAACGCAATGGAGTTATTGAGTGT

GGAGAATTTGTTCGATCATT
AGGTGTTTTCCATCCGGATGCACCAACAGAAGATAAAATAAAATGTGACTTTCGTCAAT
TAATTATGACACTCTTTTCAA
TAATTTGCTTGCATAGATTTTGTGTAGTTTCCTAATATTCTAAACCAAAACTCTTATGGAC
AGTTGCTTTTAGGCTTTAT
GATCTCAGACAAACTGGTTTTATCGAGAGAGAGGAGGTTAGTAGTAATTATTTATCGTT
CTTGATAATTAAGTTGATATT
AAATAAAAGGGTTCATTTATATGCTATGTTCATTTAAAAAATTTGTTTTTGTGCAACAGT
TGAACGAAATGGTAATAGCA
CTGTTAGACGAATCTGATTTGGTACTTTCAGAAGACGTTATTGAAATGATAGTCGACAA
AACATTTAGTGATGCGGATAT
TAAAGGGGATGGAAAGATAGATGAAGAGGAATGGAAGCAATTCGTAGCACATAATCCA
TCTCTTATAAAGAACATGACAC
TTCCCTATTTAAAGTATACCACTTTATTGACAATTAGAAATTTATCTTCCATTAAAATGCA
TGGTATTTTGATATTTTAA
TTAACCTGGCACCTACTGAATTTCAAGAACTAATAGACAATATATCTGTAGGGACATAA
CTTTGGCATTCCCGAGCTTTG
TTGTAACCTCTGAAGTAGAAGACTCGGAAGTATAG
>CsCML47
ATGGGCTGCATGTGCTCTAGTGGCATTAAGCATACTCCTGGGTATGAAGATCCTGCTATT
CTAGCTAACGAAACCCCGTG
TGAGTTCTCTAAATTATACTTTTCACATCATATTATCTTATGCACATTTACAAGTTCATTTG
CCGAGTTTTAATCATTGA
TATGATTTGGAAGAAAAATCTTTAAGTACTAGTTTCAGATTAAAAAAAAAAAAAAAAA
AAAAAAAAAAAAAAAAAAAAA
AAAAACTGAATTTGGCATCTTCTCTGGATGGATATATATATTGTACATGGAAAAAACTAG
TGATTGTTTTTTTTCTTTTA
CAGTCACCGTGAGCGAAGTTGAGTCATTGTATGAGCTATTTAACAAGTTGAGCAGTTC
CATTATCGATGATGGCCTTATT
CACAAGGTATTTTTAAATGATAATGATCTTTGAAACTTTTTAAGTAATCAAAATACCTAC
TTATCAAGGGAGATACTAGT
AGTAGATTAAGTACTAATCCTCTTAGCATATTCAGGAAGAGTTCCAGCTCGCGCTCTTC
AGAAACAGAAACAAGCGTAAT
CTTTTCGCAGACAGGGTATGTTAATGCAATAAACAATAACTATCTAGCATTTGCGGCTTC
TTAAATACGGAGTACATCTT
TTGTTTTTTACTAAAGAAAACGTTGCTCGGATTTTTCTTGTAGATATTCGATCTATTTGAT
GTGAAACACAATGGAGTTA
TTGAGCGTGGAGAATTTGTTCGATCATTAGGTGTTTTCCATCCGGATGCACCAACCGAA
GATAAAATTGAATGCGAGTTT
CGTCAATTAATTATGACATTCTTTTCAATAATCTGCTTGCATAGAGTTTGTGTAGTTTTCT
AATATTCTAAACCAAAACT
CTTATGGACAGTTGCTTTTAGGCTTTATGATCTCAGACAAACTGGTTTTATCGAGAGAG
AGGAGGTTAGTAGTAATTATT
TATCGTTCTTGATAATTAACCTGACATTAAATAAAATGGTTCATTTTTATGCTATATTCATT

TAAACAATTTGTTTTTGT
GCAACAGTTGAATGAGATGGTAATAGCATTGTTAGACGAATCAGATTTGGTACTTTCGG
AAGACGTTATAGAGATGATAG
TCGACAAAACATTTAGTGATGCGGATATTAAAGGGGATGGAAAGATAGATGAAGAGGA
ATGGAAGCAATTCGTAGCGCAT
AATCCATCTCTTATAAAGAACATGACCCTTCCCTATTTAAAGTATACCACTTTCTTGACA
ATCAGAAGTTTATCTTCCAT
TAAATTGCATGATAATTTGATATTTTAATTAACTTGGCACCTACTTAATTTCAAGACCTAA
TAGACAATATATCTGTAGG
GACATAAGTTTGGCATTCCCGAGCTTTGTTCTAACCTCTGAGTGTGAAGTAGAAGACTC
AGAAGTATAG
>CsCML48
ATGGAGCTTACGTCCGTAGCAACTGCAGCCACAACAGGAGGTATACTGTCCATGGAGG
TCAAATATTTCATAATCTTATA
CATGTTTGTAGAGTGGGTGAAACTTTTACATGATTGTTATTTCCTTTTGGCACGACCCAT
GTGGCGGTTTATCTCTACTA
CAGAAAACACTACTTGTAGACCAACTCCAAGCACAGTTGCTCAAGCCCACGACACTAT
ACAGGTTTTCCATGCATATAAT
AGCAGGAAGAAGTCATTAGGAGTAGATCTTGAAATAGTTTTGAAAAGATTAGGAATGT
TTTGTGATCATGATGACAAGGG
ACAAATTATTGGCTCTGATGAGATATTGGGTTTGTTTGACGAGGACGAGCCAAGCTTGG
ATGAAGTGAAAGAAGCATTTA
GTGCGTTTGACAGAAACAAAGACGGGTTCATAGACGCAAAGGAGTTGCAACACGCGC
TTTCAGAGATGGGGTGTATACAA
ATATCAGAAAGCAATTGTAGACTGATGATTGGAGGATATGATGTCGATCAAGATGATAA
GATTAGTTTTAGAGAGTTTTT
GAAGCTCATGGAAGACTGTTTTTGA
>CsCML49
ATGGGTTGTATAAGTTCAACTCCGAAGGAATTCAAAAAGTTACCAGCTTATGATCCGGT
TGCGCTTGCTGCTGAGACCCC
TTGTAAGTTTTCTATATTCTCCCATTTTTCTGATTATCTTTTTGGCTATTTTTTCATTTGTTT
ACATGTTTTATACGGAG
TATCATCTTTTGCCATAGTTGTATTGACGACCTTTTATACAGTTTAGCCTCTTAGGTAGTT
ACAAGTGTGGTAACAAGTA
TCACAAATTATTGTTATTTTCATTGCTGCGTTATCAATGTATGTGATGTGTCATTATTGAG
AGTCTTGAGCTATTAGTAC
CGTCCAAACAAATTTAACAAATAGAAAAACCGTTTTGGGATGTATTGGATTTTAAAATC
TGTGTGGCATTCGACACTTGC
AGTTACTGTGAATGAGGTGGAGGCTTTATACGAGCTGTTTGAGAAACTAAGCAGTTCC
GTGGTGGATGATGGGCTTATAG
GGAAGGTATACATTCCTTTTATGATGCCGTGAATGTGACATTTTCGGTTGGTTTAATATG
TCTTTGCTTGGAAGTAAATT
TTATTAAATAAATTTCACATTTGGCAGGACGAGTTTAACCTAGCGCTATTTCGAAATCAT
AGTAAACGAAACCTGTTTGC

CGATCGGGTAAGTATGGTTCAGCTTTTGTCCCTATCAAAGAAAAACGTACAACGGTAA
CTGTATGCTGCTCTTAGTTTGG
ATATGAATCATAGGATTGTCAACATTTTCCATATATAAACTAATCGTCTTTTTCATATCGTT
CCTATAGCAACTTATGTT
GTTACTTTTGTAGATATTTGATCTTTTTGACGTAAATAGGAGTGGGCATATTGACTTTAA
CGAGTTTGTCCGTTCGTTGA
GTGTATTTCATCCAAAAGCTCCTCAAGCCGACAAAGTTTTGTGTAAGAATAATGATATG
ATACTTTTTTTCGTGGTTTTA
TATTCACAATTATATGATAGGAATGATCATCTGATACATTATGCAATCAATCTTGTTTTAG
ATGCGTTCAGACTTTATGA
TTTAAGACGCACTGGCTTCATCGAACGTGAGGAGGTAACTTGTTCACTTTGTTGTTTTT
TACCAATTAAAATATGATTTG
TTTTCCATATCTAAAATATACTTAAGAATAATATAATGTAACAATGCTGATTCTGGTAACT
TTTTATAATGGGAAGCAGT
TGAAGGATATGGTTAAGGCTCTTCTGAGTGAATCTGATCTTGTTCTATCAGATGAAATAA
TTGAATCGATGGTAGACAAG
GTATGCAATACATGTTTGTTATTTTGTAGTGACAACTTTAAGGCTTGGGTTGTGATGATT
GTATCTTTTTGTTTCATATT
TAGACTTTCGCAGAAGCAGATTATAAAGGTGATGGTAAGATTGACCAAGAAGAATGGA
GGGAGTATGTCGACAAGAACCC
GTCACTATTGAAGAACATGACTCTTCCTCACTTAATGTAAGCCATACAACTTGCATCAAT
GCAATTTTTATATTATTTTT
TATTTTGAGTTTAAAGCATGCCAACACTTAAGATGGCAATTTTTAGGACCTTACATAATT
ATGCTCTTATCAAGTTATTT
TTACCTTTTTTAGACTCGTTTCAGATAATTGTTTTGTGTCCTTAAAATGTTAGTTTTATAC
ATACGGATGATATCTCGCA
ATCTGTAAAGTTAGAACAAAGCTAAGATGCAAAAACTATGACTGCAAAAGAGGAAAA
ACAAGTTGCTTAGTTTGGACGGA
TAATCTGATATGTACAATACCAAGTAATTGTTACAGTATAAACGTAGGTTATAGTATTGTG
CTTGTTTTACATATATGAT
GTACTGTATTAATCATGAATGACTATTTTCTTCTGCTGTGTGGTTGTAGATTTGCATATAA
AAATAGCTGCATAGAACTA
ATTTTTAAGAATATCTCTATCCTAGATGCGTATCTTTGCAATATGGGTATTCTTCAATATCA
GTTTACGTAAAAAAACTT
ATCTTCGAAATGACCCTTTGTGATGTTGCATTTTTGCCAATTTTTGAATTCAGTCTTGAT
TTTTAAGTATGGTATTTGCG
AAATGACTTTATATCTCTTGATGGTGCGTTTCAGGGACATAACTTTGAGGTTTCCAAGC
TTTGTGATGAACAGCCAGGTA
GAAGAGACTCAGTCAACAGACTAG
>CsCML50
ATGTCTTATAACCCATCAGGTTACAACTATGGTGCACCACCACCTTCTCAACCATACTAC
TCCACCCCTTACGCCGCACC
GTCACCTTACGGTCAGCCACAACAGCCCGCTTTCACGAGCCCCTACGGTTATGGAACA
TCCACATTTCCACCGGGGACGG

ATCCTAATGTGATTGCGTGTTTTCAAGTGGCTGACATAGACCGAAGTGGTGTCGTTGAT
GATAAGGAGTTGCAAATAGCG
TTGTCGTCGTATAATCAAAGTTTTAGTATCCGTACTGTTCGTCTACTTATGCATCATTTTA
CAAATACCAACACTAGAAA
GATTGGTAAATATTGAAATATGATTTTTATTTTTTTAGCTGCCTTAAATATAGTATTCGACT
AATATGTATAATCTTTAT
TGACTTGTTCTCTTGATGATATCAGGACCAAAGGAATTTACTCAAGTGTTTTATAGTCTA
CAAAATTGGAGAGTATGTTA
GAAATATAAGCTGCATTAATTTTGCCCTTTTAAATATGTTCAACCAAATGTTTTTATACCT
AAGTAACTTTGTGTTTACA
GGCAATTTTTGAGAAATTTGATCGAGATCGAAGTGGTCATATCGACACCTGGGAACTG
AGAGAGGCACTCATGAGCCTCG
GATTTACTGTTTCACCCGTTGTCTTGGATTTACTCGTCTCCAAGTTTGACAAGACTGGT
GGATATAAGAAGGCCATTGAG
TACGACAATTTCATCGAGTATGTCTTCGATCATAACAACTTTTATATTACGATCAAGTTCC
ATTAGTCTTTCCTAAATTT
CTAATAAGTTTTTTTTTGTTGTTTCTGGTAATTTCAGGTGTTGCCTCATTGTTAAGGTATG
TAATACAAGAAAATAAAAC
CTTATAACTAGTTAATGTCTATAAGCTAACATTTATGCAACAATCAAGATACATGTCTTAA
AACTAGGTTCTCGACTCTT
ATAAGATTGTGTGATCGATGTATGCAGGGGTTAACCGAAAAGTTCAAGGAGAAAGACA
CAATGTATTCAGGCAATGCGAC
ATTCACATACGAGGCTTTTATGTTAACAGTTCTTCCGTTTCTCATTGTTTAA
>CsCML51
ATGGGAAACACATCATCAATGCTGACACAATACGACATCGAAGAAGTACAAGAACATT
GTAACAACACATGTAAGTATTC
AAATCTGACTGAACCCATGTAAAATATATAAAGTTTTGATGTTTCTTTTTGGTTGATTTG
TAGTTTCACAACAAGAGATA
GTGTCATTGTACCAAAGGTTTTGTCAGTTGGATCGAAATAGTGGTGGATTTATCTCTGC
TGATGAGTTTCTTTCGGTCCC
CGAATTCGCTGTTAATCCCTTGTCTCAGGTTTAATTATCATACCCTTTTCAGCATTATGTT
GTTTGATTGTTGAAATTTA
ATGTTGTTAGAGTTTGCATGTTTGTGTTTTTTTTAGTGTGGGAATTTGAATGTTTGGATG
ATGTTGTTTGAAGTCGTGAT
GATCAGATAATCAGTTGTTTCAAAACGCGAAAGTGCAATACCAAACACCCTTTTAGTG
AATTGATGGATTCTGACCATTT
TGATGTGTTTACGTGTAAAGTGACTGCTAAGTGGTTATATAACAAGAATTAGTTTTGTTG
TGTCATCCAAACACTAGAAA
CTAGAAATTGATTATATGATACGTGAAAAGGGAATAATCTGATAATGACTTTCTAAATGC
AAGTCCAAACGCCCCTATTA
ACGTTTGTCAAATTTAAGGTTATTTAAGGCTTTTAATTTGTTTTTGTTTTTTTTTGGTTCT
GTCAGAGGTTGTTCAGGAT
GATTGATGGGTTGAATTTCAAGGAGTTTGTGGCATTTTTGTCTGCATTTAGTTCGCGTG
CCACCTTACAGCATAAAGTGG

AATGTAACTACCTTCTCCTTTGCTGTATGACATTCCGTAAAGATTTATATAATCTTACTTG
TTAAGAATGAATCTTACTC

TATTGCAGTTATATTTAAGGTGTATGATTCCGATGGCAATGGGAAGGTTGCATTCTCTGA
TCTGTTAGACGTTTTGCGGG

ACTTGACAGGGCAATTCATTTCTGAACAACAAAGAGAGGTAAGTGTTATTACTTGACA
ATGGTAGCATTTGGTTGTTTGG

TTAGTGTTTAAGAGACTACTGAAAATAGAGTACGTCAGCATGGGAGCACAAGCACTTG
TCAATCTAATGACCAACTGAAA

ATCTTGTCTGGAAGAAACCATGTACTCATGAAATCTACAATTCCTATATCATATGTCACA
AGAATTTGCAACATCTGACT

CTAGATACAAACTAGTTGACGTAGCTATATGTGTGAAATGTTTTCTAGCTTCATGTTCAA
GAAAATCCTGGGCATTGAAA

CTAGTTGTAACTACCAAGCGATAATTAGATCAACGAGTTGGTAATTAAAAGGGAAAGA
AATGCGTTAACTTTTATGGGAT

GAAAGGAGCATAGGAATACTGATCTATGATGGTCAGAGAATGGAGATTTAGACTACTTT
TTAAATTTAAAACTGAAGTGA

ATTTTATTTGATCGTCTTCTTTAAAAGATGGTTAACATAAATACTATTTAATTTACAAGTG
GAGAAAGTAATGTCAGCTC

CATTGTATACATCTTCAATATACTCGCCCAAATCTTATATATGTGAAAGAAAAAAAAAAA
GTGTTTTATAAATTATAACA

TGCATTTAGCTGCTTGCATCTGAAAATGTATGCAATGGTTTCGTTGATTAATGTGCTTTT
ACATGTCACCAAGGCTAGAA

GTAGAGGTTTCAGCTGTAGTTTAGCAACATAATGCATACAACTTTTAATTCTGAGAGTA
CTAGGTTTCGAATGTAAAAAA

TTAAGTGCATCTCTTCATTAAGTCAGAAATGTTTGTACTAAACTCGTTTAAGTCGCCTAC
CTTTTCTTGGCATTCCAAAA

CGTATTTTATTTGGCTTCATTTAAGTGAAAGTTGCATATGATTTGAAGTTGAAAAGCCTA
ATATGATCATTTGGATGACA

GAAAATCAAAACAAATATTATCTACAACTAGTTCCGTCACTCCGTGTAGACGGGAATTA
AAGGAAAAGAAAGGGAGTAAA

GTGATTATACACTGTGTTTGTATATATGTGAGGGAAATGCATTTCATATTGGGTACTATAT
AGTCTGGGAATATGCATCA

TCACCCTATTACCCCTAGGTGCATCCTTTATTTAAGTATAGTTATCAACACAAACCAAAT
TCACTAGTCAAGTAACGTGT

TATAAAGACTAACAAGAATAGCAACAGGTCGTAGTGAAAACTTTTAGAATTATGCATCA
GAAGTCAAAGTTGCTCAATGG

CATACAAAGACAAGTGAAATTTGCCGATACATAGTAGCATCAACATTTTAATTCGCAAG
ACTAGAATACTGAAGACTATA

AACAATCTGACTGTCTCAATGATTAAAAGGTTTGGGCAATGCATCAACATTTATACTTAT
GGATCTACAGCATGAACACT

TCCGAAGAGGCTCCTGAGATTATGACCACACTGGAGATCATAATCAAACAGAAACAAA
ACATATATTAAAAAGAGCACAA

TCATGATAAGAATCATTCTTATAACATTTGATCCGTTCAATGTTTATTTCACTTTCTATGT
GATTATGTTTATATTTTTG

TACTTCCGTCATGTGTTCATGTGATGTTTATGGTCATGTCTTTAATAAGTTCTGCAGCCC
CGCCTCCTCTCATGCTAACT
TTGTTGGCTACTGCCTACTGACGTATAGCTATATGTTCTTAGTATACAAATTCCTTTGCTA
TTAACTTGTTTAATTTGAT
TGCCTTGCATTTTTATTTACTTAAAGGACATGTTGAGGGTATATGGAAATTCTTGTGTTT
TTCTTTTTTGTCAACACTCG
CAAACAGAGCTATTTCTATTACTGCCTCTCTGACAATTTCAGATTGTATTTTTTTTTTAAC
ATATGTGATCTTAATCTCA
ATTCCACTTCTACAGCTTGTCCTGACGCAAGTTCTGGAAGAAGCTGGGTACAAGAAAG
ATTCGCTATTAGTTTTGTCCGA
CTTCATGAAGGTAATGAGTTTATCTTTTCTAACTCAACAACTTTAACTCCCTCTGTTTCA
CACTTAGGATGATGTTTTGG
TTCATTTCTATAGAAGGATATTCAGTGTATGATGAAACATTGTACTCAAAATTAGTGTTC
ATGGTTACATCTTACCTTCA
TATTTGCAAAATTTGGAATTTGGTACCCACTTGTGAACTATTCTGATTTGATCAGTTTTG
TCATTCAGTTACTGATAGTA
AATGGATTTGGTAGCTGTGATGGGCATAAGCATGGTAATGTCGTTGTAGAAGTTATTCAT
TTGCATATGATGCAGTACAT
CCACCTCTACATAGCCACAGCAATTTCTATTTCAGAATGTTCAATTTTGGTTACAAGTTT
CGTTTATGACACGTTTTCAT
GCTAAAAATACCTGAATTCCCAAATTCATGATGTATAGGCCATTAGTTATTCTTCAAGTC
TTTTACTAGGATGAGGATAT
TATTTCTTGTACGAGAATATTGAACTACCTTGTGTTGGATTTCGGATCCAGAAGACTTTA
GCATGTTTATAAAGTGATAG
GAGCATAATCTTGTGACAGTCATTCATGGCTTGAGGTTTGGTCACAAATATATCAGGCA
CACCAACACTGCATGAACATC
ATCTATAACTTTTCCATGTGCATATGAAGAGTTACATTTTAGGTAGTCCTTTTTTTTAATT
AATAGTAACATCCCATATG
GTAAACCTTGGTACACGGGAACTCACTTTTTCGAGGATACCCTTGTATGTGGTGTTTTC
TTTGCACTTTGAGCGGGGTTG
TTTCTGGGCTTTGAACCCTTATTTGTTTAGTACTTCATAATAAACATTTATTGCTGCTTGC
TGCAGATTCTTGGCAACAC
AGGCTTGAAGATGGAGGTTGAGGTTCCCGTGGACTAGTTGGAGACATTAAAGATACAT
AGATTATTATAATTGCTTGCTC
TCTTAATAGGGCCTTTAGCTGCTTGTATCTTGTAGTGTAAATGACCTATGTTCTCAGTTA
CCCTCTGTTTGATTCTGAAT
TTGGTCGTATTGATTAGCATGTGTATTCCACAGTTTATTAGCATTTCATGGATTGGTTTAA
AAAGAACTTCCATTTATTG
TGTTTGATCTAGCTTTAATGGTGACACAACTCATTCAGTATAACAAGAGATTTTGGTTAT
GTAGCTGTTTTAGTTATGAT
TTTGGGTGTGTGTGTGTGTTGGTATAGAATGTGTTAATGTGTAGATAGCATGTTTTGTTT
ATATGTAAGGTAACATGTTG
TTCTACAACTTATTTGTATCATTGTCTAGTTTAGACAACATGACCACATCTGTAACCTTG
GTACTCTATACAAACATTCT

GAATTATACACAGCTTAAACATGTCACTTGTATTAGGCCATTCAGCTTGAACTCTATAAC
ATAGCTCGCCTCTTAATGGA
CTAGCTAAATAATAATTAATAACACAACTTTTTTTTTACCCCAATGCTTTACAAAAAACT
CATTACAAAGCAAAAAGGGA
TCAAATCAACAAAAAGGGGGTATCACTGATGATACAAACACATGATGATGATGATTGTT
AATTTGTTAGTCAGAGTGATA
ATTTTGATTAGGGTTGACGATAACAACACATATGACGTGTGTTACCGGTCGTTAGGTGC
ACAAGCATATATGAGAGATGG
TGCGTGTAGTCAATACATTTAAAGCAAATATCGTACATACATACGCACATACTATATCATT
GTACACATGTAAAGTTCGT
CAAGTAGTTTTTAACGATTTTCAAATTTTATGGTCAAAGGCGGTACACGGCGGTGCGTG
AAGGTTTTTAAAGAGGTTACA
TTGGGGTGTTACACTGAGACTCTTAAGTGGATGGGGTTCAATTTTTGCAAGCAGCCTCT
TCGAAGGCTAGTTTTTGGGTG
CAAACGTCTTTGTGATGTCGGGAAGAGAAATTCTACAAATTGGTCATGTGTTTGAACTA
CCTTTGGTTCGTAAGTTACAA
ATGAACATTACACGAAATACGATTCTTAGAAGAAGGTCACATCAAATTCAAAATGAATC
ATGAAATAATGATTGCTCATT
GATTTTTATGAAGTACAGTAGTAATTAAAATGATAAGGATCCTCTAAAATTTACCTTCTC
ATCTCATGATGGTCATGTTG
AACAAATCAACAATAATAAAACAGAACCACAACTAATTAAAATAAGAGACTTATTATTT
TGATTTCTCTCACAATATCAA
TAACATAATAGAACATAAGAGAGTTAAACTTTAAAAAAGCTCAATCCTAATTAAAATAG
ATACAAACGAATACTATTTCT
TTAAAATTTATTGTTTACCTTTTTAAGGATTGTTAATTTTCACTACTAAAAGTTATTAAAA
AAAACCGCATCGAGTTTAT
CATATTAACTTTACTTGAATTTGTTACCCCTTGCGTCATTTAATAGGTGTCAACACCTTTA
TTTTTGTTTCTCTCAAAAT
AAGTGTCTAATTAATATGTCAACTAATCAAAAGAGACGTTAGTTAGTAGAGATGTTAATT
TATTTGTGTAGAATTTATAT
ATTTAAAGTTTTGTACATTTTTAAAAGAAAAACTTATTATAAAAACTTAAAAAGTATTAT
TAACTAACTGCTTGAGGACA
AAACTTTAATTCTACAAAATACATTAAAAAAAAAAAAAAAAAAAAATGAGTGCTTTTG
TTTTCCTCGTGAAGGGGGAATT
ATTTGACTTTTGATTGGTCGGATACCGCCAATTCCAATTCCAAATATGATTTTTCTAACTT
GTGCCTAAGCGCGCAAGTT
AAGCACACTAAAAGGACACTGAGACCCACAGAAAAGGACACATGAATTCACAGAAA
AGTACAATTTTTGTGGATCCCTGT
GTCTTTTTCTGTAGGTCCAGTGTCCTTTTAGTGTGTTTAACTTGTGCCTCAGGGCACATT
TTAGACAAACCCTTCCAAAT
ATGTTGGAATAATTTCAATTCTAAATGATGTCATTGGAACCTTAAAAATTCACAAACCCT
ACTCATCTTTATCACAAACA
CCTCTCTTCAACTACTACTTGACAACACAGGTATGTCCAACTACGAGTATAATGTTTACT
GTACTACTAATTTAGGAGTG

TTTGGATTAACCTATTTTTGGGCTTATTATAGAAAAAGTATAATCTTAACCCGAAATCAG
GTATTTAATTATTTATATAG
CCAATTCTTTTGTCCCAGTGGTATATAATTATGCCCACTTACGTAGAGGTCGTGCGTTTA
AGTCTCGTTAAAAAAACATT
AGTGAGATTTGTGGTACAGGAGTCCCAAAAGATTGCGGTTCTAAAAAATTAACTCATTT
TGGACGAAATGTGGCCTTTTT
CTAACGACGGCACCTCACTGTGTTTTCGAGTATCACTTAAGTAGGAAAAGGAAAGAAA
ATGAAGAGAAACAAAAAAAATT
GTGTTCTTGAGTGTCGCTCAAGGAGGAAAATAAAATGAAAAGGACTGAAAAATGTGA
TATTAGTCATTGAAAACCTTTTT
GATTGGTTGAAATATACAAGTGAACACTTATTTTGAGACAAAACAAAATAAAAAGATG
AACATATAATTTGGGACGGAAG
GAGTAATCAAGTCGAGTTCATGGACTTAAGTAAGCTGTATGCTTAAGGTGATAAGCTTT
TATGTGAAAAACGAAAAGTAA
GCTTGCAAAGCTTTTTTAGTTTCAAATAAGCCAGTAAGCCACAAAAACAAATTAATCCA
AATACCCTCTTTAATGTGTTA
AGTTTTATTATGATGACACTTTTGGTCAGTTAACTGACAGGAAATGGCTCGACTTAGTT
TAA
>CsCML52
ATGTCGTGCTTAGAGGGGATCAGGCATTTACTTGCTCCCCTGCTCCGTTGCTTTAATCTT
GAATCATTCACAAAAACAAA
CCCCCTTGAAGATCCTGAAGTTCTAGCCCGAGAGACTGTTTGTATGCTACCAATCTAAG
TAGTCTTTAAAGTAGTTATTT
GTGACATTGATTAATTGGGTTATTACTAACTAGAGTCGGTTGTGGTGTAATGCAGTTAGT
GTAAGCGAAATAGAAGCACT
CTATGAGTTGTTTAAGAAAATTAGCAGTGCTGTGACTGATGACGGGCTAATTAGTAAGG
TTTAAATCCTCTCTGTATTCT
TTTTTTTTTTTTTTTCATTCTTTTTCGATAATTATAGGATTGCAATATCAATATTAGATAATC
TATATGTACATCACAGG
AAGAGTTCCAGTTAGCCTTATTTAAGACAAATAAAAAGGAGAGCCTTTTTGCTGATCG
AGTGTTCGATCTGTTTGATACC
AAGCATAATGGAATCTTAGGTTTCGAAGAGTTTGCTCGTGCACTCTCTGTATTTCATCC
AAATGCCCCTGTTGATGAAAA
GATCGAGTGTAAGTTGAAATAGTTTTCTATTAATATATGACCTTGGATTTAGTATTAAAGC
GTATTCTCATTCTCCATTT
GAAAATTTGCAGTTTCCTTTCAACTTTATGATCTTAAGAAACAAGGTTTCATAGAGAGG
CAAGAGGTAACTTTTGTAATT
AAATTGACATCTTTTGTAGCGTCAGAATGATTTTATAGCATGTGGATAATTATATTGAATT
GTGACATGTAGGTGAAGCA
AATGGTGGTGGCGACTCTTGCTGAATCCAAAATGAAACTTACTGATGATGTTATTGAGA
ATATCATTAATACGGTACCTG
AACTAGTTAAATCTTATGCTGACTATATTCACAACAATTCGATAGTCTAACAAAACTGAA
TAATTGTGTACTTTGTTTAG
ACATTTCAGGAAGCTGATACGAAACATGATGGGAAGATTGACATGGAAGAATGGAGAA

GCCTTGCTCTACGACATCCGTC
CCTTTTGAAGAATATGACCCTTCATTACCTTAAGTAAGCTTCGTTGCTTCATAGGATTGA
TACTATTGTTCTGCATACAT
TTGGAACCGATGCTACCAAGAGGTTATTAGCCCAATAATCGATGATTGGTAGTTCTGCT
TCGTAATAGACGTTTGTGCTA
ATTATGAGTTATGGATATTGAGTTATCTTGAAATTTGACAGGGACATCACAACAACATTT
CCGAGCTTTGTTTTTCACTC
AAGAGTTGAGGATTCGTAA
>CsCML53
ATGGGTGGTAAGTTGACCAAGGCTGATGAATCACCTAAGACTTCGGTACCTACAACCA
AGCTTGAAGCTAAAATCTTGGA
GACAATCCGTCGCAGAGAATCTAAAGGAACTTCCATGAAATCGTTCAATACTATAATCC
TGAAGTTCCCAAAAATTGACG
CAAGCCTAAGAAAGTGCAAAGCTATATTTGAGCAATTTGGTAAGCACATAAAAATTTCG
TGATATAGTAAATTAATCTTC
AGCTTAGCTGTTTTAGTTTCTATTGTCTGTGAATTCTATGGCAGTGTTTGGATGTGCGTA
TAAACTTCAGTTTTGAGTTT
TATTAGTAGAAGTGTACAACTAATCAGTTCTGGAGAAGCATGTGCTAAGTTGTAAGAAA
ACTTATTATTGGGATAGAGGT
GAAATAGTGGTGACCCAGCGGTAAGAATACCAGCCTGGGATTACTCCGGGCTATCCAA
AAGGGTAGTCGCTCTGGGATTA
GTCGGCTCGCAATGTCGAGTCGGAAACCCAGGTAACCAAAAAAAATAAAATAAAAAA
TAGTTGTTCACTTTATTGCATAA
AAATTTAATGTGCCCTTTTTATATAATAATTATCATTTTTGAAAGTCTGGTTTTAAGAGCT
CTTGGTTTCAAAATGGAAT
GACTTAAAGGTTGTAAAAATCGCTAGGCGCTAGTGAGGCGGTCGGGTAGGGCCTAGCG
ATTAATCGGTTAGGCGGGGATT
AATCGGGGATTAATCGGATTGGAATTTTTATGCACAAAATTGTAATTTTAGATATCATATT
ATATTATATGCTTGTATGA
AATAAGCATAGTGCATCAAAGTTCAAATATTTTAAGTGTTCAAATATAAACATAATACAT
AATTCTTCAAAATTTAAAGT
CAAACATAGATATTACTCGGTTTGTTGACCATTTCTAAGCGATCTTTGACTGATTAATCA
GTTTTTTGACCAATTTTTAA
GCGAGTTTGTCCGATTTTTAAGCGATTTTGTTGACATATTTTTAAGCGATTTTGTTTGATT
AATCAGATTAGACCGATTT
TGTTCGATTTAGTCCGATTAATCGGTTTGTTGACTGATTTTTAAGCGATCTTTGACAAAC
CGATTAAATAGGCCGATTAT
GATAAATCAAGGCGCTTAGTGAGCGATCAGCGATTATGCGGGGATTAATCGGCCGCCTG
GACCGATTTTTACAACAGGTA
AGTTAAATGTACTAGACTTGAAAGAAACAAATGATAAAGTACATTGGTTATGGATGCAA
ATCACCCATAAAATAATCTAC
CTATCTTTAGCTTTAATTATATCAATAACTATTTGTAACTCTTCACATTGAATACATCCAA
ACACCCCTACATATTTATT
TCATCTTTCATTCAATTTTATCTTCAATTTTGCGAGTGCAGATGAAGATAAAAGTGGTAC

AATTGATCCGAAAGAGCTGA
ATCATTGTTTTCGTAAGCTGGAAATAGATTTTACAGATGAAGAAATCAGTGATTTATTTA
AAGAATGTGATATAAACCAT
GACATGGGAATAAACTTCAAAGAGTTCATTGTGCTCCTGTGCCTCGTTTATCTTCTGAA
GAATGACCCTGTTTCCCCCCA
TTCTGTATCCTTGCTAACGTCTACACAAATACAGTATATGGTTTACTCATTTTTTGGTGTG
CTTTTGTGTTCTTTTTTCC
ATCATTTTGTTATATTCTTGACACTGATAGAGTTCACGCATGGGGATGCCGGAACTACAA
GCTTTTGAAACTTTGGTGGA
CTCATTTGTGTTCTTGGACAAGAACAAAGATGGTTATGTTAGTCGAAGCGAGATGGTTG
ATGCCATAAATGAAACTACAA
CTGGCGAGCGGTCTTCTGGGCGCATAGCTATGAGAAGATTCGGTATCTCTTCCCCTCTT
TATTTTCACATGGGCGGGTTG
GGTAATGGTTCAAAACACGTTTGTTCAAATTTCTAAAATGTAGGGTGTTTTTGTTAAAT
AACCCACTCAGAATGGTTCAG
ATTAGTAAATCCCCATATTATTACAATGATAACCTGATTTTTTGAATAGTTCGATGTAGGA
GGTTAATCCTTATTTTCTC
GTAGCTATATTTTCTCTATGACCGATTCAACACCTTTAAAGCAATATAATACTTCAAAATT
ACCACCTGTTTTGCACCTA
GAATGCTTATAAAAGTTGCTAACATTGTGTTTTTTGTATTCGCAGAAGAGATGGATTGG
GATAAAAATGGAATGGTGAAC
TTCAAGGAGTTCATCTTTGCCTTCGAGAAATGGATTGGAATCGAGGATGGCGAGGAGG
ATGAAGAAGAGGTAGCAGACGA
GCAAGAATGA
>CsCML54
ATGGCATCAACCAATAATATGCAATCTGAGTTCCAAGATTACTTGCCATTAATGGCTGAT
AAGTTGGGTGGTGATGGTTT
AATACAAGAACTATGTAATGGGTTTCAGTTACTTATGGATCAAGATAAAGGGGTGATTA
CTTTTGATAGTTTGAAGAAGA
ATTCATCAGTTTTGGGGCTTGAAGGATTGAGTGATGATGAAGTCATGAGTATGCTGAAA
GAAGGTGATTTTGATGGTGAT
GGTGCTTTGAATCAAATGGAGTTTTGTGTTCTTATGTTTAGATTAAGTCCGAATTTGATG
GATCAATCTGAATACTTGTT
GGAAGAAGCTTTGGAACAAGAGTTAAATAATTTTCAATACTGA
>CsCML55
ATGACAACCAATTATAATTCTAGTTTTCATGATTTCTTGCCGTTAATGGCGGATAAGTTG
GGTGGTGATGGTTTGGTAGA
TGAACTATGCAAAGGGTTCGAGTTGCTGATGGATCAAGATAAAGGGGTGATTACTTTTG
ATAGTTTGAAGAAGAATTCAT
CAGTTTTGGGGCTTGAAGGATTGAGTGATGATGAAGTTATGAGTATGTTGAAAGAAGG
TGATTTTGATGGTGATGGTGCT
TTGAATCAAATGGAGTTTTGTGTTCTTATGTTTAGATTAAGTCCAAATTTAATGGATCAA
TCTGAATACTTGTTGGAAGC
AGCTCTTGAACAAGAATTTAATTATTTGTGA

>CsCML56

ATGAAAGCCGCTAGTAATAGCCCCATGGGCACATTATTTAGTACATCAATTAGAAATTT
TAAAAGTGGGAATCTTGATA
GCCGGATCATATATACTAATATGTTTCGTGTTTAACTACAGTTACCGGTCTGTTTAACACT
CAAGAGGTAGCTCAAGAGT
ATGTCGTGACTGGAGTTGGTTTGCTAGCCGGATCAACGATATTCCTTCTCACTTTATTAT
GGGGAACTTGTATCATTATT
GGAAGCCAAAAGTTTTCTTCAGAATCGGGTGCCAGTACTTCCGTGGATCCTACCCAAT
GCCCAAACAAGAAATTCTTCTC
ATTTCTTACTAGTTAGATTCAATCCTTCATCACAACCTACTACCCGGCCTTTATTTAAATT
CATAGACACGTAACGTAAG
CCATCATTTTTTAACGTGTTATTGTATGATTTCAGGTTCTGGTGTAACTACAGACCCCGA
GACATGTACAGCGGCGCAAA
TCATGCTTCTGTCAGTTATACCGTTTCTGTTTCTGTTGATCCCGAAGTTGTTTGGCATGA
CGTATGCACCTCATGGATAC
ATTTTTTTAATAGCACTTCCTGTTTCAGTTACCTTCTTGCTGGTATACTTCATTTATCAGG
TATGTATGTACATGTTTGT
CTGCTGTTTATGCGGTGAACCTGAATTTATAGAAAATAAAAGTAGCTAGATTATTTCATA
TGAGCTTAATCTGTTACTAT
AAGCAATAACTAGAATTGGATATGTGTCTTATCAGTTTGCTTCTTAAAGCTGTTGAAAAT
GGGTCGGATTGACGTCCCAT
TTTTATTTCCATATTGTAAAATTTTCTCTAGAAAAAGTATTTTATTTTGTATAATTTGGTTT
AGGAGGATGATGATACTA
GAATTAAACTTTGGGCGACATTCAATACATTTCATATCTAGCATTTTGTTTAAAAAACAA
ATCTCAAATCGATTCAATAC
ATTTCATATCTAGCATTTTGTTTAAAAAAAAGTTGGTGGAATGAAATTGCCTCCTCTACT
TTACAAAATATTCATGTTTT
TCACTGTTAGAGTCTTCTTCTTTCTGTTCTCCTTATTCATACACTGCACATAGCATATTAG
CATAGTGACTTTTGTCAAG
AAAATAACCACAATGATCCTTAGAAGATTAACTAATGAAAGTGGGTTCCTTTTTTGTAC
TTTCATCGTCATCATTTTGAA
TTTGTTCATGTGCAGGTGTTTGAGCCTTCAATTCAAAAGAGACGATTGTCATACGTCAA
ACACGAGCATTTAGTTTTAGA
CATACTAAAACATTTACAGGAGCAAATCCCAGAAAATATACTCGCTGAAGATGGTTCAG
TCAACCTGCCTGCTATAAAAA
GGTCCACAACCTTTTCCAAAAGAATATTTTTTAACGTGTGTTTCCAGTAAGATCAATTTA
ATTATGGATTTTCAGTTTGT
TTAAGAAGATTGATCAAGATGGAGATGACATCATATCTTTTTCTGAGCTCAAAGAACTT
CTCGAAAGTATTAAGTTCAGG
CAATTAAAATCGGACAAACAGAAAACATTCGATCAACTGATAAAAGAATTTGATTCTG
ATGGTAACGCACAAGTATCGCT
AGATGAATTCATCCATCGGTTCACAGAATGGCTTGATGAGGCTAAAAATGAATTATCTG
AGGTATTTATCTTATTATTAA
TTCGTGCTCACAATTTTTAGACATCTTCGCCAGCCTGACTGCAGAATGACATCAGTAAT

TAATAGTCTAATAAACTTTTT
CTAGTGAATCAAACTTTATATCATATATTAAAAAAGTTGTTGAATTTATTCAAAGGCAGT
ATCAAATACTAATCTGAAGA
TGACTAGGTTGACCCAGCGAAACTCTACATTTGCTCTCGGAGATCATCCAACTACTCAT
ATCTTTTCCATTACTAACATG
AATGATTGTAACTTACTTTTCTGCACGTTAGATGACAAAGATATGATAATGTTGAAATCT
TTTTAAAAAAGGCGATTGCT
TACTTTTACGTTCTGAAAGATTAGTAATTATTAAACAAGATAGGATACATACTTCCGTGG
CCTCTAGAGTAGTTTCTACT
GTTGGTGAGGGCTAATGAAAATAGTTCTGCCTCGCACATCATCCCATTTATATATAAAGG
GAGAATGATTCCTGTAATAT
CTTTTTTTCAGGTCGTCAAACCATTGGTTCAAACCAAAAGGAATGAAGATGACATGAC
GCAAGTCCTCGTATCTGAAATT
ATCGGCAATGCTAAAAGCTCTCCACTAGGGAAGTTTTACAAAGAAGATGGAACACCGG
ACATATCTGCCATAAAAAAGTA
AGACAATTATCGTTAGCATCTAGAAAGAAAAAACAAAGTCTTTTATCAACCACCTGATT
CCTGTGGAAATTAATTTTAGA
TTGTTTAGGAGCTTGGATGTCAACAAAGATGGTTCTGTGTCGTTAACCGAGTTGAAAA
AACTTACAATGCATGTCAACTT
GGGTGAGACATCATGGAATGTGGATGAAACAACATCTCGCATAATGCAAAATCTTGATA
CAAATGGAGATAAAGAGATCG
ATGAACAGGAATTCGTTGATGGATTTGAAAAGAAATTGGTGAACATAACTAATGACCG
ATCCAAGACATCTGGGCCTAAA
GATGTATCACGGGTAAGTAGGTCGACCTTTGCCGTTGAATAAGCTAAAATTACTAACTT
CAAAGTTGACGTGACTTTTTT
TTTTAATTCTAAACAAGTTTTAATAACTGCTTTGAATTTTTAAAGAAATGATTCAAATAC
AAAAATCGTTGGTGAAGAAC
CTGTAATGATATGGGTTTAACCTAAAAAGTCAGGGTGTTTATGACTATGCAGAAAGCAT
GCAAAAAATGGAAAGGTGATA
ATGTGGATAGATCTGTGTGGGGATGGACAAAGGCTATAATGCTACTGGTGCTCGGGATA
GCAATGTTGGCTCTGATGGCT
GAACCTCTTATACACAGTGTTCAAAATGTCTCCAACTCGGCTGCCATGCCGTCCTTTTT
CATATCGTTTATCTTGGTTCC
ATTAGCCACAAATGCTAGAGCAGCTATCTCTGCAATCCGAACTGCAAGTCAAGGGAAA
GAACGAACTACTTCATTAACAT
TTTCTGAGGTTTGTGTTTTATCATATAGCAATCTTGTTTGGGATTCATAAAGGCATTACAT
ATAAAAATTGTTAAAAGTC
TGTGTACTTTTCAGAACTTTTCTCATATATCATACGGTTATCCACATTCATATAGATAATAC
TCCGTAAATGAGAAGTCA
TAAATGCCATGCATGCATGCCTAATATTATGTGCTTTTTTCATATGTATAGTTTTAATGTTT
TATAAATAAAACATGAAA
GTTTCTATGCTATTTTCTTACAGCATGTACATTTGATCACTAAAACATCTGCGAGTCTGT
TACGGTTTGTTGCTTTTTTT
CATCTGTAGTGCGTATGCCAGCCTTGGCTGGCTTTTGACAAGCAAGTTTGATGATAGAA

CATGTCTTTCCTTTTATGCTT
TTTTCTATATGTACATCTTAGAGGCGGCGAGATGGTTGTGTTGGGGAAGGGTTGTTGGA
CTTAATGTTATAGAAGAAAAG
ATTAGGGTAGTTTAGTATAATTTTATGTTATGTATTCTGTAAGTTTAGGTATAAACCGACC
CATTGTATTGGGTTGGGTC
ATATAACCGTTTTAAAGAATTGTAACATTTAGTTTTAGTTGCATCGTTTACTTTTAGAGA
ACCGGTATGCCTCAAGTTCT
TGGCACCGATTCATTAGGTATATATAGGTTAGATTTCTGTAAACAAATTAATTCCAATATA
TCAATACAACATCAATCGT
TTATAATAATACAATATTCAGTTCTTATATTCAACTCTTTATATCTTTCTGCTGGTGCACAA
TACGCTTCTGTCCCAAAC
TTCTGTAAACACTCCGGTGTTAATCCAGACCGATTCAAGAGCCTGTACAATCCGAAGG
AAGAAAGCAACAATTGGCATCA
GAGCTAAAGGATAATCGACCCAGGGAAAGACGAAAACAACAATTCCTGTAACCGTTG
ATTTCAGAAGCAAGATAGAAGAA
AAACGATTTCTGTTTGGAAATCTATTTACCGAACCAAAAAAGGAACAACACAGAAATT
GATCAGAAGGAAGGATATTGGA
ATCACGAATCAGGAAAAGCATCTCGAATCTGTGAAAGGAACGCACGTCTGTTGTTTCC
CCTAATCGACCGGTTTTTTTAA
TTCTCTCAACCTTTTTACTTTTTTTACTACTCCATATAAATTAGGAAATGTTTTTTTATTG
ATTGTTAGTAAGATAGAAA
TTCCCATTAGCAAGAAAAAAAAACCCGATTGATTAGAGTAGCAAAGACCGATTTTTTTA
GATTCATAGTAAGACAAACGA
ATTCTTCAAAAAATTCGTTTCAGCAAATAGCAAGTTAATCCGAACCCTTGTGGTTCATA
GCAAGTTAATCCGAATCCTTG
TGGTTGATAGCAAGTTAATCCGAATCCTAGTGATTCTTAGCAAGTCAAAACTGAATCCT
TAGGTTCATAGCAAGTTTTTC
GAGCCTTAAGATTCAAATAGCAAGTTTCGAGCCTTTTCTTAGGTTCAAATAGCAAGTAG
AACCCTTGATTCAAACCAGCA
AGAAAGTAGAATCATTGGTTTTTAAATATCAGCAAGAAAGGATCAATTGTTTTGATCAA
AGCAAAGCAAGCATGGTAGAA
GATGACAAGCATGATAACGCATCAAGCAGTGCATCAATTCGAGAAGGGAATGTTTCAC
TTCAATGTCCAAAACTGACAGA
TACTAATTATACAACTTGGGCACTGATGATGAAAACAATATTAAAGGCGTATGGACTTT
GGAAAGTGATTGATGGCATGA
AAGAAACTAGCGGCTAG
>CsCML57
ATGGGTAAGAATTCTTACTTTATTATTCTTCATTAGGCTTTTAGCTCTCATATATGATATCC
TTGTTAAGTTGTAAAGAA
AGCCTGAGAAACTTATCTATTCAGTTTTATAACTTTCTTGTTTAATCTCATAATTTTCCTA
AATCTTAGCTTAAACATGC
TACTTTGTTAGCTTTTATTCGAATAAAAATCCGAAATGGATGTTACTCTAGTGCTAGAGT
TAAAGGGCGTTATATACGAG
ATTTTGAATATTTATATGTTCATGTATGTATGATGTATGGTGTTTTGAATCGGTTTCTTGAA

GGTACATCTCACACAAGC
CCTTCATTCAAGTCGTTATCCAACAAAGTCGGAGTTATGCTATGTTGTTGCAATTCACA
AAACCGATACGAGAGATTAGA
CAATAAGCTCGAAAGAAAAATGATGGAGGTCAAACAGAACAGCATACAAGGTCAAAC
CAGTTTCAGATCTATCGATAGCA
TTATCTTAAGGTTCCCGCGGTTCAAAGAAGGATTAAAGGAGATTCAAGGTGTTTTTGA
ACTATACGGTAAATCTAACTAG
CTTAAACAGATTAATACTTTTATCATAATTTTTGCGTTCTTCTGTGTGGTTTAAAGCTGCT
TGCGATTCTGCAGATGAAG
ATTCAAATGGAACCATTGATAACGAGGAGCTAAAGAGATGCTTACAGAAGCTGGAATT
TCATTGTACCGAGCAGGAGATT
AGAGATCTTTTCGAGTCTTGTGATGTGGATGGGAGCAATGGGATACAGTTTAACGAGTT
TATTGTTCTTCTGTGTCTCAT
TTATCTCTTAGACGGTCCTTCCTCATCATCTCATGTGGTAAATAACCATCTTCTCGTTTAT
AGTAATATTTAGTTTCTTG
ATAACTAAAGGTGACAATTTCAACCCATTTACTTATGAGTCGGTCGATTTTTGTCATGCG
TTATCTACAATGGGTCAAAT
GTGTCGAAAGTTGTTCAATTTTTTTTATATGCATATTAGGACTAGATAGTGTATTTATAAG
AAAGTTAAGTTTTCGACAA
CATAGTGTTTTGGGTCAACCCAACTCGTCCAAATATTACCCGTTTTGATATAATTGTCAA
TAAAATCAGACTAAAGAGAA
TTTTGTTTCAGACATCGACAGTGGGGTCACCCGAGCTCAAAGCAACATTTGATACCATC
ATCGAAGCTTTCTTGTTTCTT
GATAAAAACGGTGATGGAAAGCTGAACAAAAAGGACATGATGAAGGCAATGAATGAA
GACTTCCCAATGGAGAAATCACC
TACACATATCACCAAGACCCGATTCAGTAATTTACTTGATTTAACTACTACGAGTAAGTA
TTTTTGTGGATTACAATAAC
AAAAAATTTACATTTTTCTTTTTGGAAAATGTAGAAGAAATGGATTGGAACAAGGATGG
TAAGGTGAGCTTCAGGGAGTT
CTTGTTCTCTTTAATTAACTGGGTCGGGTTAGAATCCACTGATGAAGTACCTGAAACGG
TGTTTTGA
>CsCML58
ATGTCATACTCCGGTTACAATCCAAACACCACCATTCCATCAGCCCCACCCGCACCACC
CTCCCAATCCCACCAACCCGC
AACCGCATACCCTTACCAACAACCACCACCTCAAAATTACAACAACCAACAAACCTAC
AACCCCTCCGGGTATGGAGGCG
GCTACGGGTCCCAATACGGGTCGTACCCGCCTCAACAAACGGTGTCGTTTCCACCAGG
GACGCACCCCGAAGTGATTCGA
AGCTTCCAGGCTGTTGATTTGGATAGGAGTGGGTTTATTGATGCTAAGGAGCTTCAACA
GGCTTTGACTCAGGCGTATCT
CAAGTTTAGTTCCAGGACTATTAAGCTGCTCATGTTTCAGTTTAGGAACCCCACTGATC
CCACACGAACCGGTGAGTTTA
CGAGTTTTTAGGTTTTTTTAGCGCGTTTTTATTTACAATATAAATAATGTTTGTTTCGCAA
CTAGTGTTTACCTTTTGGA

ACGCTAATGTTAACTACAATTTTAGGGTGTCTTTGGCGCAGAGCGTTTAGGAGCAGTCA
GCGTCGAGCGTTATGATTTTT

AATGCTCGTTTGACAAACAACAAGTAGCTTTATCTAATTTTTCGGAATGCACCTTTTCC
ACGCTCCCTGAAGTAGCATTT

AGGAGCGTCGAGCATTTAGGAGCTGTTTTACATATTATGATTGAGGTGTAATCATAATGT
GTAAAACAACTCCTAAATGT

TCAACGCTCCTAAACGCTACTTCATGGAGTTTTGGAAAATGAGCATTCCGAAAAATTAG
ATAAATCTACTTGCTGCTTGC

TAAACGAGCATTAAAAATCATAACGCCCAACGCTTCTAAACGTTATGCGCCTACGTTAT
TATCTTCTTTCTTTTATCTAA

ATAATAAGTTTTTTGGAATCCATGTGACCTCATCACTGGAACTGGTTCATTTTCGTTTCG
CTTTTTTGATTTGGTTTCGT

TGTATTAGTTGTGATATTATAATAGGTCAAGTCCTAATAGTAGTAGTAGTAGTAGTAGTGT
GAGTAACTATCAGTTTCTG

TGTAATAATAGTAACTTTTTTGCAACCATCCAATTGTAATAATAGTAACTTTTTTGCATGT
TGATGTGTGTCAACATGTT

ATTATATAAAAGTCACTATTATCGCACAAAAAACTTGAAGTTACTTATACTATGGCTATG
TTTGGCAAACCTAGCAGTAT

TATGAAAGCGTGGCGTTTGTTGTATGTCTACTTATAAAGTGTAAAGTGCCTTAAAGCTA
AGCACATTTTTGTTAGCGTTA

CAAATAGGAGCTTTTAAACACAATCTAAAATCTAAACGCTTGTTAACAAGCTTTTTATT
CTAAATGCTCGTCACCAAACT

CAGACTATTTATGACGTTTTTTCTAGGGGCATTACGAGGATTAGGAATTGAGATATACTA
TTTGTTACTATATTATGCAA

AACAAAACATAACTATATGATTAGATGTGTCTATGGTAAATATATAATCTACCGGGTTGTA
TATAAAAGTGTTGGGATGT

GCATATGGTGTTTATTTGAAGTTGTGATAAACTGATAATCAGATGGTCTTACTTTTAAAT
GATCCTTAAATAAGGAGATA

ATCGGAATCGGTTTTATTTGCAACTACGCGTAAGTTGGTTATACAAAACTTATGGATAAG
TAGAATTAGTTTCACTATGC

ACAAACACTAGATACTTGCTGAGTTGTGGAGACTGATGGTTGCCACATGAGGAGGTGC
CCGCGCCTCAGTCGGTCTATAT

GCCATTTTAGGTCCAAACTAATTAATAATTAGGCGAGTAACGGCAAAAAAATTGTTTTT
AGGTTGCTTTACATGTACATT

GCTAGATTTGTTTAACTTATTTTGGAGCAAAGAAGTAATTAGAAACCACTTTTATATCTG
TTAAGCAGATTGAAATAAGC

CATCCGGTAGATGACCTTTTTTTGGGTAAAGGGTTTATACCCGGTGAATATAAATAAGCC
ATCCGGTACATGACCTTAGT

ATCTGATTATCACAACTTTGGACAGTTGCCTCCCGTTTTCTTGTAACCAACTATTATTTG
ATTATCGAAATTGTACGCAC

GGTTGCAGCGTTTTGTTTGTAGTGTTTCTATTTTGATGATTTGACATCACATAATTACTTT
AAGAACTCAATGCAAACAT

TCCTAAGTCTTGCTGAACCATGTACATGCACTCATGACTCATCTTATTTATTGCAATGAT
CGTGTTTTAGATTTTGATTA

TTTCGTTTAACTTGTTAAAGTAGGCATTGTCATTTCTCCTGATGTGCATTGTATATACTTA
TGTGACTTTCTTTTGGTGT
TTTACAGGTCCTAAAGAGTTTGCTGAGTTATGGAGTTGTCTTGGCCAATGGCGGGTGA
GTGATCGTATTTCATTATTGTA
TAAATATAAATATTGGGTGCACGATTTTTCCACATAGATAGCAGAAGAGTGAGCAGTC
TTAAAGGAACTTGTTTTACTG
GCTGGATTTGCAAATTGTGGCGTATAACTTCTATTTGGGGTAGTGAACGTTGTTCACTTT
ACCCTCAAAATAGGCTAATG
TGGCTTCATTATTTGTGAATTTAGGGTAATCCGACGGGTTATACGGTTAATGATGTGGCA
GATGATGCATTAAAACTTTT
TACTATGTTGACTTTCTTTTTACATGGCTCTGAATCGTTGATATCGCATAGTAAAGCAATT
AGCTTACTAGATTACCTCA
AATAAAAACTATTGGAACTTCATTAAGTACTTCTTGGGTAAAAAAAGGTTACCATCCAT
AAAAGAAATCAGACTTAAGTA
TGTTATCTTTGGTGGCGTTTGTTTATTGTTTTCTTTTGTGCACTGTAGGCGATCTTTGAG
AGGTTTGACAGGGATCGAAG
TGGAAAGATTGATCTAGCGGAACTAAGAGATGCCTTATACAGTCTTGGATATGCAATTC
CACCTTCTGTCCTACAACTCT
TGATTTCAAAATACGACGACCAAAGTGGAAGGAGGGTAGATCTGTCCTTTGACAGCTT
TGTTGAGTAAGTCTTTTACTTA
AAACTTAGTTTATCACATTCACATTTTACGTTTACAGCTTGTTATTTGTCTTAGTTCTAAT
GTTTTACCTAAAATTTTAA
TTTTCAGGTGCGGAATGATTATAAAGGTGAGCAGCAAGTTCATCCCTTATCCCTCACTC
TATTTCCATTTTTTTTTCTT
TATAATAATTATGTGATGATGCTCAGATTTAGAGTTGGCCTTATAAATGAGTGATATGGAT
TTGGTTTATAACTGGTCAT
ATGGGTCCAACGGGTTGATACTTGTCAAAAGTCTATCTTGTGATGCATACAACCTTCTA
AATTGTTTTTTTTAAGCAAAT
TTGATTTTATTGTACCTATAATCTTAATCTTAATTAACATTGTAACACGTCCATTACTTATA
GAAAATGAAAAATCATAC
AACGTTGGGCAATAAAGTATTGGCAGGTCAACTCAACCCAATTTGGCCCTTTTCACCCT
CACTAGAAATGACCCCTTTTA
GGCGTATAACCAGAACCATTTGAACCAGACGAAACCAAACCACATCTGCCTCTTCTAC
TCATAAATTAGTATTTATTTCT
TGATCCCTGTGAAATTTACATACCAAGTGTTATTGTATCCATGAATAATTGATGAAAAAG
ATCATCTTGTTGACTCGAAA
TCATTATCACCAAGGTTTTGTGACAGAGACAATTGGTAGATAGGTAAATTTTTCAGTTTT
CATAGGAGAAGAAACTAGAA
GCATTAGGGTCAACCTGTAAACTTAAATTGCTATCCCTTTTTTAGAGCATGAGTAGTGTT
TTTTTTATCGTTATCTTGTT
CTTTATGTGTTTGAAACACGTAATTTCAGGGTTTGACGGAGAAGTTCAAGGAGAAAGA
TACTAGGTACACGGGTTCAGCC
ACGCTTTCATACGAGACATTTATGACCATGGTCATCCCATTTCTTGTAGCAGAATAA
>CsCML59

ATGGCCTTCGGTTCTCAGTCGAATGTTCTCGATCCTCCTCCAAAGTTTGATATGTACAA

ATATACTTTTGTCATGACTGA

CGCTGACCTGGAGGGTGTTGTCGCTGGGTATGGTATATCTCTAACAGTGCTAAAGACTT

GGTCAGAAAAATGCTTATTAG

AGACCCCAGAAAGCGGATAACGGCTCATGGAGTTCTTTGTAAGTCTAACTTGTTCTTTG

TCATTTTTTTTTGTTTCAATT

AGGGCGTGCTTGGAAGTGTGTTTTGGATGTGATTATATATTTGATTATGCCATTATGGTG

TTTTAACTCACATAATTATT

TTCAAGCTTTGGGTATGCTAAAATTATTTATTCCATAGCACGTGCTCACATTCAAAATGC

AATGCCAAACGCTCTATTAG

ATTATTATGCACTATAGTTCTTCATCACTGTAGGCCACCCTTGGATAAGTGAAGATGGTG

TTGCTCCAGACAAGCCTCTT

GATTCTGCAGTCTTGAGCCGCTTAACTCAGTTTTCTGCCATGAACAAGCTCAAAAGAA

TGGCTCTTAGGGTAAGCAGCAC

TTCAAGAATTTATTTATCTATAAGGAAAACCAGTACTTTAAGAAATAACCCGATAACTTA

ACATATTACTGCCAAATGTC

AACTTTGCCCTTACCCTTCTTGTGTTCCTCACAGGTCATCGTGTCAAAACTCTCAGAAG

AAGAAACTGCAGGCTTAAAAC

AAATGTTCAAGATGATAGACACAGACAAGAGTGGTTATATTACATTTGAAGAACTAAA

GGCTGGACTCAAGCGATTCGGT

TCTACTCTTAATGAGTCTGAGATTTATGATCTAATGCAATCTGTAAGTAAATAATTTTTTT

ATAAATTTTTTTGCATTGG

TTTTCTTTGTTGCTGAATATAAATGATCTTTGATTCTTTAAACTGAAAATGCAGGCGGAT

ATTGATAATGGTACTATCGA

CTATGAAGAATTTGTAGCATCAACGTTACATATGAACAAAGTTGATAGAGACGATCATT

TGTTTGCTGCTTTTTCATATT

TTGACAAAGATGATAGTGGTTATATAACTCTTGATGAACTCCAACAAGCGTGCAAAGA

GTTTGGATTAGATGATGTTCAT

TTAGAAGAAATTATCAAAGAAGCTGACCAAAACAATGTACGTTTTTCACTACCATAA

>CsCML60

ATGGCGGAACAACTGACTGAAGAACAAATTGCTGAGTTCAAGGAAGCTTTCAGCCTC

TTTGACAAAGACGGTGATGGTAT

ATTTTTTTCTCGACATCTCAAATCCTTTTTTTTTTTTTTTTTTTTTAATAAAATGATTTCT

TGTTACTAGTTAGATTTG

TTTTTACTAGTTTGTTATAGTTTATTACTACCTTAGTATAAGTAAATAAACAAACATTTGA

ACATTTGTTTGGTCAAAAC

TCGGTCAACACACGGTCAAAAGTTGGTTTCAAATATATATCTATGAAATTTGTATATACA

AAATCAATCTGAGTATTTCA

CGCCGTGTGGAGTTTTACAACCTTGACTTGTATGTGTAGTATTCTAAGCATTTTTGGGCA

TCATAAATGATGGCTAACTA

TATGTGCCTTGAATCGTTTACTAGCCTAAATAGAAAATGAGACTTGTATGTAGGTTTCAC

TTGTTAATTTCGCCTTCTCT

TGGGTCCCCTGAATAAGTGTATGGATCCACGGGGATCGTTTAACTTTTGTTAGTTTTAG

GGTAATTGCAAAATTTACATG

TTAATTTTTTTAAGGCTTTTTAAACCTAAGTATTTGAAAGAAGTTTCAACAAAGCTGAT
TTATACTTTAACTCAACTAC
AAAGTAAAATAACATGTTCCAACAACTCCATACCCCGCTCTGGCGGGATTGGATATGTT
GTTGTTGTTGTCTACAGTCAT
GTTCATAAGGTTTCATTTCTACACTTTAAATTGCTAAACAAATGTTTTTTTGTTTATGAC
ATTCGTTGATATTCTAGCT
GTTTTCAAGTTTATATCTGATCCAATATGATCTTCTTATGATGCTATAGTGGTTCGATATAT
TATTTTTTCTGGTGCAAA
CTCTGTCCCCCTTTATAATTAAGTTCTACTGTTAGCGTTAAAAAAGTAATATAGTGACGG
GTTGTTTTCATATTGAATAG
TTGTTGGGATGTTTACACTTAGATACGCTTGTGAAGAACATTGACTGAACTGATTATCA
AATCTTGGCTAAATGTTCAAT
GGTAGATAATTACTACACGTCACTGTAACTGAAGTATCTCACAACGTTGATGAAAGAAA
TGGTTTCTCGCCTTTAACAGG
CTGAAATGTACGAGAAGGTTTAGTGTGTACCACCTGTCACCTCCTAGATGGTTTTATTT
ATAGATTCAAAATATTGTAGT
TAACAATTTAGCACATAAGCTTCTTTAGAAAAAATATTTTAAAGTTTGACAAGAAAAAA
GTCCTCTCTGTTTGATCTTCA
ACATGAACCCGTTTGACTCGTTCCCATCTTGGCCCAACATATTGAGAGTGTTCTGGCTA
CCTTATTTGTAAATATTTCTA
GTACTTAAGATATGCTGAGCTTTCTTTTGTTGGTAACTCGAATTCTGTTTTTTCATAGTTT
TATTAGAATAGACCTATCA
TGGATTCATCCAAACTCCGGCATCTTAACCTCCAATTTGCACATATGAAACTCCTATGTC
TTCTATGCAATTTGTTTTTT
GTTTTTTTTCCTTTACGGGGGAGTGCCTGCTATTTACATCGGTTGGCCTGTTTCTTTAAC
TGTTAATTACTTAGTTACAG
TGTTTAGCTTATTTTTTTGTTTTTATCTTGGAGCTCAATGGAAATTCAATATTGTAGGCTG
TATCACCACCAAAGAGTTG
GGGACAGTTATGAGATCATTGGGTCAGAATCCTACTGAAGCTGAACTGCAAGATATGAT
CAATGAAGTTGATGCTGATCA
GAATGGAACAATCGATTTCCCTGAGTTTCTGAACCTGATGGCCAGGAAAATGAAGGTG
GATATTAATAACTCTGTTGCTC
TAATATTCTATTGGAAATGTGCTCTGTTATTACTATATTTAGAGTTCAGGACTACTCATCA
TGATCTCGTAGGACTGATA
TGAAGCCTGTGTAGGTGGAAAACTTGGGAGGTGGGTAACAAGTAAAAATGGCTCATG
TCAAACAGAACAGAACGAACTGA
GCCGGTTTAAACAAAACACTTGTGCAGTATATGATCACAAAAAATATGTCATATTACAA
CATATATCTATTAATTTATTC
CAACAATTTAGTATTTGCATTACAAGATACACTTTGGGCGACATGTGAACTGTTTCTTTA
TAGCTATTATGTTTCCTTTT
ACCCATGACCTGTTAAAGATATAACATAAATTAATTCAGCCCACTAATTTGTTATTGGGC
GAAGTAAAAACTAAAAAAGA
TGAGCATAAAATAATGTTTGCAAAGTTGATCAAAATGCTGTGCTCTTTTTTTGAAGGAC
ACTGATTCGGAGGAGGAACTC

AAGGAAGCTTTCAAGGTCTTTGATAAGGATCAGAATGGATACATTTCTGCTGCTGAAGT
ATGTAACCTAACTACTCTTGC
TTATCTCTGTAATTATATGTGCCAAAGCTGGCAGGTCTTGGTGTTGACCCGAAGCACTT
TTTGATTATTTCTGTGAAGT
TATTAACAGTTAATCCGTGTTAGTTTGTGAAGTTGATAAGGGTTATGTTATTTCAGTAAA
ATTCTAACTTTTTAACATGT
GATTTTGGATGACTTGTTTAGTTTTCTCACATTTGACATGTGTGCAGCTTCGCCATGTGA
TGACAAACCTCGGGGAGAAG
TTAACTGATGAAGAAGTGGATGAAATGATCCGTGAAGCTGATATGGATGGTGATGGTCA
AGTGAATTACGAGGAGTTTGT
GAGGATGATGCTTGCCAAGTGA
>CsCaM3
ATGAGGTCTCTAGGACAAAACCCAACCGAGGCTGAACTTCAAGATATGATCAACGAAG
TCGATGCTGATGGCAATGGTAC
TATTGATTTCCCTGAGTTTCTCAACTTGATGGCCCGCAAGATGAAGGACACTGATTCTG
AGGAAGAGCTCAAGGAGGCTT
TCCGGGTTTTTGACAAGGACCAAAATGGTTTCATCTCTGCGGCTGAGCTTCGTCACGT
CATAACTAATCTTGGTGAGAAG
TTGACAGATGAGGAAGTCGATGAGATGATCCGTGAAGCTTGA
>CsCML61
ATGAGTAAATCGAACGATTACAAGCGTGTATTTGATCACTTTGATGAAGACAGCAATGG
CATGGTCTCACCATCCGAGCT
ACACCGCCGTGTAGGCATGATTTGTCACGAGCAAGTTTTAATAGAAGATGTACAAGTTA
TAGTCGAGTCATTGCATGGGA
GCAAAGTTGACGGGCATGAACTAGGATTTGACGATTTCGTTAGTTTAATGGAGAGCGA
TAATGAAGATGAGAAGGTTGAG
GATTTAAGGAAGGCTTTTAGATTGTATGAAAACGATGGAAACGATTGTATAACACCGAA
AAGCTTGAATCGGATGTTGGA
TCGATTAGGTGAGTCGAGAAGTGTTGATGAGTGTGTTGGTATGATTAATCAGTTTGATC
TGAATGGTGATGGTGTGCTTA
ACTTTGAAGAGTTTAAAGCAATGATGCTTTGA
>CsCML62
ATGAATAGTTGTGAAGTTTTGGTAAAATCCCTTCAAGCTTCGGTTCAATCGCTATTGAC
CCATGTTTCAATAACATGGAA
CAAGTTAGGATCCATTTCCACATCACTTGACCTAAATGAGACCGTAAAGAAAGAGTTC
ATGACCCTTGGAGAGATAAACG
TGATCATGGGGCAACTAGGGCTCCAACAACGTTGTAGTGATCAGGATAGCAATATTGAC
ATTTTATCGGTGTTTGATGAT
GAGGAACCAACTTTGGAAGAAGTTAAGGTGGCTTTTGACGTGTTTGATGAGAACTCG
GACGGTTTCATAGATGAGAATGA
GTTGCGTGACATGTTATGCAAGTTAGGAAAACAAGAAAACGCAATGTTAAAGGAATGC
CGGAGTATGATAAAAGGATTTG
ATGTCAATGGGGATGGGCTCATTGACTTTGATGAATTTGTTAGGCTCATGGAGACATGT
TCTTTTTAA

>CsCML63
ATGGGTTTAAAGAATCTATTCAACCGTAAAACCAAGAACACGACCAAAGACAATACTA
CTATGGTCGAAGAAACAATCGC
CACAAACACAACGTTGCCCGTTGTACCTCGTCAACAAACCAAGGAACAACAACTAGA
GCAAGTTTTCAAGAAACTCGACG
TGAACAACGATGGAAAAATCTCTTACTCCGAGCTAGGATCAGTGATGGGGAGCCTAGC
AGGAAACCAACCAACGGACGAC
GAGTTAAAGAAAATGATCATGGAAGTTGATAAAGACGGGGACGGGTTCATAGACTTGG
AAGAGTTTATTGAGTTGAACAC
AAAAGTCGATTCTAGTGAGTTGTTGGAGCTTATAGAAAAAGCGTTTTCCATGTTTGATG
TTGATAAAAACGGCTTGATTA
CGGTTGAAGAGTTGTTGAGGGTTATGCGGAGCTTGCATGAAGATTATAGTATTGAAGAG
TGCAAGAAGATGATTGCTGGA
GTTGATCAAGATGGTGATGGTATGATTAATTTGAATGAGTTTAAGGTTATGATGATGAGT
GGAGTAAGGTCAGATGTTAG
TGAATCTTGA
>CsCML64
ATGAATACAGGCGAACCATCAACTTCATCTTCTCCGGTCAAATTCTTCCGACCCCCCAC
CACCATATCACCTCCGTCATC
GTCTCCATTACCAACTCCGTGCACCGACGACATCCATCAACTCTTCAACTACTTCGATG
AAAATGGCGACGGTAAAATAA
CCGCAACGGAGCTTCAAAACCGGTTAAAAACCGTCGCCGGAGATGAAGTTCAGTTATC
CGACGAGGAGGCGGAGATGGCG
GTGAGGTCATCGGATGCTGACGGTGACGGTGTGTTAGGGTTTGATGATTTCACGAAGA
TGATGAAGGAAGGGGCGGAGGA
GGAGTTGCGAGAGGCGTTTCGGATGTATTCAGCGAAATCGGGGACTGTTATTACGGCC
AACAGTTTGAGAAGGATGTTGC
GGCGGTTGGGCCAAAGTACGGTTACGGTGGAGGAGTGTAAGGGGATGATTGGGAGAT
TTGATGTTAATGGTGACGCCGTG
TTGGATTACGATGAGTTTCGAGCTATGATGAGTTAG
>CsCML65
ATGGAAACACAAACACCTACATCTAAACACGCTCCTCTTCTTAAATCATGTTCCAACGG
TTCGTTTCGTCTGCGTTCACC
CAGTTTAAACTCCTTACGTCTTCGTCGAATCTTTGATCTATTCGACTCTAATCACGACTC
ATTTATCACTATAGAAGAAA
TCACACGTGCCTTGACCCTTCTAGGCCTCGACACCAATGCCTCGGATTTGGACACTATG
ATCAAATCATACGTGCATCCA
GGCAACGTTGGGCTAACCTATGAGGACTTTGTGACATTACATAGGTCCATAAATGACTT
GTTTTTCGGTATGGACGAGGT
AGAGGAAGCTGTGGGAAGTAAAGAGGACCAAGAGGAGGCGGACTTAAACGAGGCGT
TTAAGGTTTTTGATGAGAACGGGG
ACGGGTTTATATCGGCTGCAGAGTTGCAAATGGTGCTTGGAAAGCTCGGCTTTACTGA
AGCTATTGAGATGGGGAGAGTT
AAGATGATGATCTCGTCTGTTGATCTTAACCATGACGGATGCGTTGACTTTTCTGAGTTT

AAAGACATGATGCGGGTGCT
CCAATAG
>CsCML66
ATGTGTGATGACTCAGGCGTGCCACATAGATTCGTGCTTGATATACAAGTTTTGCAAGA
GATCTCATTGCTAGATTCTGT
TGCTGAAACGGCAGTTGAGATGATGAACATCCTAAAGAAAATAAAGAAAAATGTCAA
AACCGCATCAAAGCCTATTTCAA
ATAAAATAAAACGTATGTTTAAAATAGTCCGCCCCACGGTATCCTATTTGCTTGTTGTAC
ATAAATGGAATGACGTACAC
AGTATTGATATTCGTCACTCTTGAATAAACACACTTATTTTTCATCGTATAAATGAGCAA
CACACACACACACACGTTAC
ACACACCAAAGTAGAAACGTAAGAATTAAGTTTCAGAAAATGGAACAAAAGGTACTC
CTTGATCAAACAACCTGATACAT
GTTGCAAAGTTCATTTGTGAGAACAGAACCTAACTCAAGTAGAATGTATCAAATTCAC
CCGTTATGAAATGCATCCCAAA
GCAGCTTTATCCCTGTCAGAAGCGATATTCTCATACTCTTATGCCTCTTCTTCAATTGCCT
TAATTCCAGGTTGCAGCTT
TAGTCGTCCCATATTACACCTTCGTTCCAGTTTATGTTTGTTAAAAATGCTATCCTGCTAT
ACAAATTAGGATTTGCAAC
ATGACTGAGACCCTCCATTCACCACCTGTAAAGAGACGATATGAGTTACTATTTTACCC
ATAATGAGGACCTGTCTAACC
AAATATTGAAATGCAACATATGTAGTTAACATAATTATAAGCACAAAAGAATTGCTGGTA
AAACTTTTGATTAATTAAGG
CTTTGCTTGGACAATTGTATCTCATCATTAAAACAATATGTGTATCAATACTTATTTGGTA
AACAACAACTATAGTGCTT
AACCGAACAATGAAATGACAGCAACACATACATATCGTTACTCTCTAAATTCATAACAA
TTATCTAATGTGGAAATTCTT
GTGTATCCTAATACTGATGAAAGGACATATACCTAAGCACCCCATCCTAGACCAGTTGA
AATTATAGTAGAAGACTCAGA
CCATGAACCATCTGGCCTTCGAGCAATATACCTTTTAACTGCCGTTCGACTCTCTCAGC
TAGAAACCCTTAAAAATGTGA
TACCATCTTCAAAAGACTTGATTGGAGAATCATGTAAATACAAAGAATATAATTGTAAAT
AAAGAAAAACCAAAGACCCA
AGAATAAATGTTACCTGCATAACCTTTCACCAAAGATCAATAACATGGTCTGCAAGAAC
AACGGAATACATGCTATTTAA
GTAAGTGATGACTAAACTCAGGATAATTAGTAAAGACATTAACACCTTTACACAACCCA
AGAAACACAAGACAACATGAA
AAAAGAGTAATGAAGAGCAAAGATTTCTATAAAAAAAGGAAGACATATGAATACCATA
GAAGTGTTGGGTTCTGGACAAA
GTCTTGTTTATGTAATATCGAAAACGCCTCACATGCCCACGGCACATGACTATCTGCCA
CCCTGAAATCGTTACATGAGC
AACTATTAGCATAACATCATTCTTCTTAAAAGAATAGAAAAAACACTTTTTACATGACA
GAATGTTTCATATAAAATTGT
GGTGTTTGTGGCAAGTTCACAAATAAGTGGTTATTTTGTAGCGAACAGAAGCAAACTG

CTTAAACCAAAAATCCCATTGT
GATAAGACAATAATATATTATATACAGACGTAATGCAATGCAGGGAGCAAGTTTCGGATA
AAGGAATTCCAGAGATGCAT
CTTAATTGTGTGAACTTGAACATATTAGGGTTTTGAGTTTTTTGTTTTTTTTTTTAATCCG
AATATTCCTAATAATAATG
GATTTATGTTAATAATGGATTTATTCTCATTAAAATGGTGATAGGTTTATAAATTTGGACT
TTAAAGATATAATCAAAGA
CTTGCAAGATTTAATTCAAAACATGTGTTGGTCTAGTGGTTATCATGTTTCTTTGTGGAC
TAAGAGACCCAAGTTCAATT
CTTGTTAAGCTTAAATTTTTTGAAAGAATAAATCCGAAAATTTAAATAAATCCGAAAATT
GTCTAAAATTCAAAATTAAT
TAAATGTCTTCAAATAAAATAAATTTTAAAAATCCTTACATGGCATTAATTTGTGATGAC
ATGGCATAAAAATAATTAAA
TGTCTTCAAATAAAATAAATTTTAAAAATGCTTACATGGCAACAATGTGATGACATGGC
TAATAATGAATTACTGGGCAA
TGAGGTGGCGTTTACGTGGCATTATCTACGTGTAACGCCTCCTGTTATATATAATATAGA
GATATATATATATATATATA
TATATATATATATATATATATATATATATATATATATATATATATATATATATATATATCTATA
TTATATATAACA
GGAGGCATTTGGCATTAATGTCACCTCAATGACACCTCAATGCCACCTCAATGCCACAT
AAGCATTTTTGTGCCAACTAA
GCAATTGTTAATTTTCGTTTTTTTTTTTTTTTTTTGGTAATTTTTGGATTTTTTTTAAGCAT
TTTTTAAAATTGTTTATA
AAAAAAGTCCCTTGCAAGGATTGAACCTTGGTTGTTTGGATAAATAACAAGTGTGTTT
ACCACTTAGGCAACACGTTATT
TTGATAATTATGTTGCATATTATAATCTATAAATGCTTATAATTATATTGGAATCCTATACTA
AATACAATTCTTAGAAA
TCTGTAATATTGACCATCTTAATGAGATAAGTATCAAATCTTTTACAATAATAATCTCATA
CAAAACATTTTATCCATTA
TTAAAATATCCGGATGTATATTTTTTCACAAAATAAAAAAAACCCTAATCATCAAAATTC
TCTCCAAAACTCCATCACAA
TTTTAAGGTTTTAATGATTTTAATCTGATTTTTGCTTGTTATAATAAAAATAGCTGTTTGT
TTTGCAGTTCTTGAAGCTT
TGATAACATATGTTTTTGTTTTACTGAAGCTTTGTTATTCTCATGCAAAAACTCTATTTGA
GTTTAATAGGTCACTTTTT
TTTACAGGCACCTTAATCCCTAATCATCAAAACTCTCACCATGAAGAAAACCTAATCTT
GCATGGCTAAAGGTAAATATT
ATGTAGCACTGAACAAAAGAAACCAATGGACCTGACGTATGCTTTTGATATCATTTTTT
CTTAATTGATGTTTTGTATGA
ATTTTGTTGCAGATGATCCAGAAAAGAAACCAAAAAGTATAGTTGATGTTGGTTGTGG
AATAGGTGGTAGCTCAAGGTTT
CTAGCTAAAAAGTACGGAGCTAAATGTCGTGGAATTACTCTCAACCCTGTACAAGCTG
AGCGGGCTCAGGTACTAGCTGA
TGCTCAAGGATTGGGCGATAAGGTACCAAGCTTAATCGTAATTCTTAGGAATTTGGGGG

TAAATGTTTAATTTGTTTTCT
GAAGCAATAACCTTATCTTCGTATGAGTTAATTGTATGGAATGTAGTTAGCTCTAGCTCA
ACAAAATATTGTGTAATATT
TATATGCAATTGTGCTAACTTTTGAGGTGTATCTGGAAATTGTGGCAGTGAATGATCCTA
ACCTAACTTCTTGAGTATCG
ATTTCATAACAAAAGAGTTTTAGCAACTGATACAATAGTTAATTCTGAAGGTGGATAATT
TTTGTTGTTAGATGTCATCT
GATGAAGTTATGTGTCGACATGAAAATCTTTTTTTCGAATCCTCTTAACTCAGGAATTG
GTTTATTATCTTCGCATCCTC
TCAACATTTCATATTGGTTATTGATTCGCATTTTCCACTTTAACAAATATTTTTTTGTATAT
GTAGGGATACCTCATTAT
ACCCTCCACTAATGATACTGTATTATGGCCAATGCGAGCCACTATCTGAAGTACCTCATC
AAGGCATACCTCGGGTAAGT
ATCTTGGTTTTGGATTCTTTTCAAGCTCAACTCGATGCATTAATACAAAAAGGCGATAC
AAATAGCAATGGTTTGGTTGA
TTTTTCTGAGTTTGTAGCACTCGTGGCACCCGAGCTTCTTTCTGCTAAATTGCCTTATAC
AGATGATCAACTGAAACAGC
TGTTTAAGATGTTTGATAGGGACGGAAATGGTTATATAATGGCTGCTGAGTTGGCTCATT
CTATGGCGAAACTAGGACAT
CCTTTGACGGCTGAGGAACTTACTGGGATGATCAAGGAAGCTGATACGGATGGGGATG
GACGGATTAACTTTCAGGAGTT
TTCTTGCGCGATTACTTCAGCTGCTTTTGATAATTCTTTTTCATGA
>CsCML67
ATGTGTCCAACAGGAACATCCTTATTTCCATCAAGAAACATAACCAACCTACGTTCCGC
ATTCGACATCCTAGACGTGGA
CCATGATGGCAAAATCAGCCATGAAGATCTCAAAACATCTTACTCCCATGCAGACGAC
AACATCATAGGTACGATGATGA
AAGTAGCTGACTCAAACAACAATGGGTACGTAGAATACGATGAGTTTGAGAAGGTGGT
GTTGAAAACTGATGGTAGTAAT
GTTTATGGTGTTATGGAGGATGTGTTTAAGGCTATGGATTGTGATGGTGATGGAAAAGT
TGGATATGGAGATTTAAGAAG
CTATTTGAATATGGCTGGTTTAGATGTTAATGATGATGAGATTAAGGCCATGATAAGATT
TGGTGGTGGTGGTGATTATG
ATGATGGTGTTACTTTTGATGGATTTATCAAGATATTGTCTCTTTGA
>CsCML68
ATGGCGAGAGTACTGGCCGTCCAACAACGTAAACAACTACTTGAAATTTTTAAACAGT
TTGACATGGACTCAGATGGATC
CTTAACATATCTTGAGCTAGCAGCCCTCCTTCGCTCTATAGGCCTTAATCTCTCGGGTGA
CCAAATCTATACGCTCTTTA
ATAAAATAGATTCTGATGGAAATGGGAAGGTTACATTTGAAGCATTTGTTGATGCAATG
ACGATTGATGTGAAGACAGAA
GAGATTGTTATTGATCAGAGACAACTTTTTGAAGCTTTTCGCTCATTTGATAGAGAGGG
AAATGGATTCATCACACCCAC
ACAACTAGCTATATCAATGGCTAAGATGGGTTATCCATTGACATACCATGAGCTTGTAGC

GTTGTTTAAAAAGCCGGATA
GAGATGAGGATGGTGTTATTAGTTTTAAAGAATTCTCAACTATCATGGCGAAATCAGTA
GCTGATATATTTGGGATGCAA
GTTTCATAGAGGGTAAGAATTATACTTCATAATACTCTTTTTTTTTTAAATAATAGTAAGT
AACATCAATATATTTTCTG
AAATACAAATTTATAAGAGAGATCTAAAAAGTTATTTTTTGGTTGTAATCTGATTGTGAG
GGGGTCTAAAATTATACGCT
TTATGAACGTCTTTATTAACGACGGTATTAGTACAGTGACGATGCTGTTAATATGAACCC
TTTTACTAATAACTTTTCAC
CATGATTAACAAATAATGTGTGGGTTACTGGATCACTATGGTGTTTTAGGCCTGGGAAA
TTGCAGATAGTCGTTACTATT
AATCGGTTGGACTCATACATCTAACGCACATATTGGTTTTATCTTTGTCTAGGTCTTTTTG
TAGGACTCACTAGGACGTT
GGAAAGATTGGTATCGGAGCTCGCTGATTTCTGTATAGAACTGTGTAGGACGTTAGAAG
TTAAAGTGGGGATGCAGAGAT
TGACATCCCACCCCACGTATGTAGTATTTTGA
>CsCML69
ATGTCATCAAATAACGAAGCCAACTTTCATGATTTCTTACCCATAATGGCGGATAAACTA
GGTGGTGAACGACTAATGGA
TGAGTTATGTAACGGGTTCCGAATGCTAATGGACCCTATCAAACGTGTTGTCACGTTTG
ATAGCTTAAAGAAAAACTCGG
CCGTATTGGGTATTGGAGACTTGACTAATGATGATGTGTTGAGTATGTTGAAAGAAGGC
GATTTAGACGGTGATGGTGTT
TTGAATCAGATGGAGTTTTGTGTTCTTATGTTTAGATTAAGTCCTGATTTGATGAAACAA
TCTTGGTTTTTGTTAGAAGA
AGCTTTAAGAACAATAGTTGAATTTGAATCTAATTAA
>CsCML70
ATGCTTACACTTGACCCGAAGAAGCGCATAACCTCTGCTCAAGTCCTTGGTATGTTTAA
GTCTTATTTCACATGTGCAAT
CTTTAATCGTCAAAATAATTATTTATTCATAAATTCCGTGTCATTTTGAATCGTATAGAGC
ATCCATGGATTAGAGAAGA
CGGAGAAGCATCAGACAAACCAATCGACAGTGCGGTTCTTTCAAGGATGAAGCAATTT
AGAGCTATGACTAAACTCAAAA
AACTTGCACTCAAGGTTTAATTTCTTCTTTTAACATTGCCATTTTAAATGGGTGGGTCGA
GTGGGTTTGCAATGGGTGAT
TTTATGACACATTGCCATTTTAGCTAAACTTAAATTTCCCTCATTTTACTCGGTGAATATG
ACCGGATGATCTGTTTAGG
TAAACAAGTTGAAGTTGCATCTTAAATAAAGGCGGAAAAGTGGGCCGGTTTGGGTATT
GGGTCTGAGCGGTTAAACTTTT
ATGCATTATACTGGCCAATACATTTTGGTGTGACCGAAGCAATTTATGTGTCAGTTCTTT
TCTGTCAGTCAATTCTTAAA
ACATAGTTACGAAATCATATAATCTCCACTTTTTCGGAAAATAATGGTCTAAGAAGTTAT
TGGGTGTCTTTCGATTTGTT
TGACCAGTTCCCTTTGAAGCTGATATCTCTTATTTTACCTTATTTAACCGGTTAAAGTGT

TTGACCCATTTCTATTTCCA

TTCTAGCTAATTTATACATCTTTTTATTAACAACCCGTTGAACAATAATAAGTATAAATCG

AAACCACTTTTTTTAAGGC

AAACAAGTCTAAATTGTCACCTATTAATTAAAGCCAAAAAGGGTATCATTTCATACACT

CATCGTGGATTTTACAAAAAA

AATTTAGGTGATTGCTCAAAATCTAACAACAGAGGAAATTCAAGGGTTAAAATCGATG

TTCATGAACATGGACACAGACA

AAAGTGGCACAATCACCATCGAAGAACTTAAAACCGGGTTGGCTAGACTCGGGTCAA

AGCTCACAGAATCTGAAGTTAGA

CAGCTCATGGACGCCGTAAGTTGTTTTGGTTTTCAAAATAGGGAAAATGATAAATTTAG

GCATCAAAGTTGCCACGTTTT

CCTTTTCTGGGCATCGAACTTTTTTTTTTCCTATTGTAGCACTGAACTTGCCTAATTTTC

CTTTTGTGACACAAAATCAA

ACTTCGATGCCCATAAAAGGAAAAATAGGCAAGTTCAGTGCTACAATAGGAAAAAAAA

AAGTTCGATGCCCAGAAAAGGA

AAAAAAGTTCGATGCCTAGAAAAAAAAAGTTCGACCGTTAATTTTTTATTGCACCGTT

AGTCCTTTTCGTCCGTTTTTG

CACGTTTTCGTCCGCTTTTGCACGTTTTCGTCTGTTTTTGCACAGTTTCGTCCGTTTTTA

CACATTTTCTTCCGTTTTCG

TCCGTTTTTGCACATTTTCGTCCTTTTTTGCACGTTTTCGTCCCGTTTTTGAACGTTTTC

GTCCGCTTTTGCACGTTTTC

GTATGTTTTTGCACAATTTCGTCCGTTTTTACACATTTTCTTTCGTTTTTGCACAGTTTCG

TCCGTTTTTGCACGTTTTC

GTCCGCTTTTGCACGTGCAAAAACAGACGAAAACGTGCAAAAGCGGACGAAAACGT

GCAAAAACAGACGAAACTGTGCAA

AAACGGAAGAAAATGTGTAAAAACGGACGAAACTGTGCAAAAACATACGAAAACATA

CAAAAGCGGATGAAAACGTGCAA

AAACGGACGAAAACCGTGCAAAAGCAGACGAAAACGTGCAAAAACGGACGAAAACC

GTGCAAAAGCGGACGAAAACGTGC

AAGACCGGACGAAAACGCGCAGAAACAGACGAAAATGTGAAAAAACGGATGAAAAC

GTGTAAAAACGGACAAAAACCGTG

TAAGAACGGACGAAAACGTGTAAGAACGGGCGAAAAGGACTAACGGTGCAATAATAT

AATAACGGTCGTTAAAAACAGAC

GAAAAGGACTAACGGTGCAATAAAAAAATTAACGGTCGAACTTTTTTTTCTGGGCAT

CGAACTTTTTTTCCTTTTCTGG

GCATCAAACTTTTTTTTTTCCTATTGTAGCACTGAACTTGCCTAATTTTCCTTTTGTGAC

ACAAAATCAAACTTCGATGC

CCAGAAAAGGAAAATTAGGCAAGTTCAGTGCTACAATAGGAAAAAAAAAAGTTCGAT

GCCCAGAAAAGGAAAACGTGGCA

ACTTTGATGTCTAAATTTATCATTTTCCCTTCAAAATATGCTTTTCTATATATCAAACATTT

TTTGAGACTCAGAAATAA

TGTTTATGTTTCAGGCTGATGTTGATGGAAATGGGTCGATTGATTACATTGAGTTCATTA

CGGCAAGAATGCATCGACAC

AAACTTGAACGTGAAGAAGATTTGTACAAAGCGTTTCAGCATTTTGATACAGATGGTA

GCGGGTATACGTTTGAACCTTT
TTCTAATGTGAGAATCTCATAAAATGTTATTTCTCCAACTTACTTATGCTCTGAATTCAG
GTTTATTACAAGAGACGAAC
TAGAAAATGCAATGAAAGAAAATGGATTGGGCGATGAAGCTACCATAAAAGATATCAT
ATCAGAAGTTGACACCGATAAC
GTGAGTTCTTCTACATCGTCATCCTCTTGCTCATCTTCTTCTTCTTCAAGTGTTCAGTTT
ATAACGGCAAACATTTTTTT
TTTCTTTTGCAGGACGGGAAGATAAACTATGAAGAGTTTTGTACAATGATGAGAAGTG
GAACCCAAGGAGCAAAGCTGTT
TTAA
>CsCML71
ATGCTTACACTTGACCCGAAGAAGCGTATAACCTCTGCTCAAGTCCTTGGTATGTTTAA
GTCTTATTTCACATGTTGCAA
TCTTAATCGTCAAATCGTCATAATAATCATTTATTTATAAATTTCGTGTCATTTTGAATCGT
ATAGAGCATCCATGGATT
AGAGAAGACGGAGAAGCATCAGACAAACCAATCGACAGTGCGGTTCTTTCAAGGATG
AAGCAATTTAGAGCTATGAATAA
ACTCAAAAAACTTGCACTCAAGGTTTAATTTCCTTTTTAACAGCATTAACTAACTCGTA
TATGGCTGTGTTGTATGAATG
TTAAATAAATGGGCCGGTCGAGTGGGTTTGCAACGGGTGATTTTAGCTAAACTTAAATT
TCCCTCATTTTACACTGTGAG
TATGACCGGAAGATATGTTTAGGTAAACATGTTGAAGTTGCATCTTTAATAAGGGCAGA
AAAGTGGGCGGTTTGGGTAGT
GGGTCTGAGCGGTTAAACTTTTATACATTATACTGGCCAATACAGTTTGTTGTGACCAA
AGCAATTTAAGTGTCAGTTCT
TTTCTGTCAATCAATTCTTAAAATATAGTTACGATATCATATAATCTATGCTTTTTCAGAA
AATAATGATCTAAGAAGTT
AATTGGGTGTCTTTCGATTTGTTTGACCAGTTCCCTTTGAAGCTGATATCTCTTATTTTA
CCTTATTTAACCAGTTAAAG
TGTTTGACCCATTTCCATTTCCATTCTAGCTAAGTTATAGTAGTACATCTTTTTATTAACA
ACCTGTTGAACAATAATAA
GTATAAATCGAAGCCACTTTTTTTAAGGCAAACAAGTCTAAATTGTCACCTATTAATTAA
AGCCAAAAAGGGTATCATTT
CATACACTCATCGTGGATTTTACAAAAAAAATTAGGTGATTGCTGAAAATCTAACAACA
GAGGAAATTCAAGGGTTAAAA
TCGATGTTCATGAACATGGACACAGACAAAAGTGGCACAATCACCTACGAAGAACTTA
AAACCGGGTTGGCTAGACTCGG
GTCAAAGCTCACAGAAGCTGAAGTTAGACAACTCATGGACGCCGTAAGTTGTTTTAGC
TTTCAAATTCTAAAGATCATTC
TTTATATACCAAACATTTTTAAGACTTCAAAAGATTGTTTGTGTTTCAGGCTGATGTTGA
TGGAAATGGGTCGATTGATT
ACATTGAGTTCATCACAGCAACAATGCATCGACACAAACTTGAACGTGAAGAAGATTT
GTACAAAGCTTTTCAGCATTTT
GATACAGATGGTAGCGGGTACGCGTTTGAACCTTATTCTAATGTGAGAGTCATATAATAT

GTTATTTCTCCAACTTACCT
TATGCTCTGAATTCAGGTTTATTACAAGAGACGAACTAGAAAATGCAATGAAAGAAAA
TGGATTGGGCGATGAAGCTACC
ATAAAAGATATCATATCAGAAGTTGACACCGATAATGTGAGTTTTTCTACATCGTCATCC
TCTTGTTCTTGTTCTTCTTC
TTCAAGTGTTCAGTTTGTAAACGCAAACATTTTTTACTCTTTTGTAGGACGGGAAGATA
AACTATGAAGAGTTTTGTACA
ATGATGAGAAGTGGAACCCAAGGAGCAAAGCTGTTTTAA
>CsCML72
ATGACTCAGTGCTTAGAGGGGATCAGGCATTTACTTGCTTCCATATTGCGCTGCTGTGA
TCTTGAATTGTACAAACAATC
AAGAGGCCTCGACGATCCTGAAATTTTAGCTAGAGAGACAGTGTGTATGTGACTAGTC
TAACATATTACATTCCAGAATT
TGATAGTTTTGAAATATTGTACTTTTAACATATAAACCACTGATAGTTTTGAAATAATCCA
GAAGTTGACGTCATACTGA
AAAAGGCTATGTTTTGCTCAATGCAGTTAGTGTAAGTGAAATTGAGGCACTTTATGAGT
TATTTAAGAAGATTAGCAGTG
CAGTTATTGATGATGGTTTAATAAATAAGGTTCATACTTTGTTTTATTCCTTTTTACTTATT
CTTTAAATCCGTGCAATA
TCAGCCCTGTGAAATTACCATTGATGTTCATCTTACGTTGCTACACTTTGTAGGAAGAG
TTCCAGTTGGCGTTGTTTAAG
ACTAATAAAAAAGAAAGTTTGTTTGCTGATCGGGTATGTCCTCTTTAAGATAAATAAAA
CAGTTTAGCATTATAAATAAG
TGTTCTATAAAACTCTCTTTCTGTTGCTCCACTCAGGTGTTTGACCTATTTGACACAAA
GCATAATGGAATCCTGGGATT
TGAGGAGTTTGCTCGTGCACTATCAGTATTTCATCCTAATGCTCCTATTGATGATAAGAT
TGAATGTGAGTTAAATTTTA
TTTCTTCTGGTACTAGATATAGTCATGTAGACTTCGGCTTGCTGTTTGTGATTCCTTATTG
CATTTGTTATCCTTTTGTT
GGCAATCTTTTAAGCTAATGTTAAAATTTGCCCGCAATATGTTATTGATTTTGGTCCACA
AAATTCACTTTAGTTTGAAA
ATTAACTGAAGGTATTTAGACATTTGGCCAATTTTTTAACACTTGCATAAGTGATTGCTG
CAAATTTCGTGAGTTCTTGG
TATATGGTATGATTAAGAATCTCTTATTCATTTCTTCTTTCTTTGCAGTTTCCTTTCAATTA
TATGATCTTAAGCAACAA
GGTTTCATAGAGAGGCAAGAGGTAAAATCATTTGTTCTTTTGTACATGCAACATAAACC
GTTTAAAGGTTGGCTTTTCTG
GTGTTAAGTAAGATTTGCTCTGTTCACAAAGCTATCCTAGATATGCTTATTATACGATTTA
ATTTATAGGAATGAAAGTA
AATGGTTTTGAATATATATTAATCAACAAGGCTTATCACAGCTTAGTGGTTCAAGCCTGA
CATGGATTAGAGCCAACTCA
ACTGCCTGCCAAGCCAAATTCGAGCTATGATTCTCGTTAAATTTTATATTCCGTTTTCCC
TAGAGAAAGGGTCACTTTAC
AGTCCTGAGAATATAAATAGAAACTATTTAAAATTTTTATATTAAATAGGATAATATTTAC

ACATAGTAGTAAGACTTTA
TGCTGTTAGTATTGAGTTCAATTGCCAATTTGACAAGTTTAATTCTTTTATATATTAGTA
ATTGTCAACACACTCATCA
TTCTGTCAACATGTAGGTAAAGCAAATGGTGGTAGCTACCCTTGCTGAGTCTGGCATGA
ATCTCTCAGACGATGTCATAG
AGAGCATTATTGACAAGGTACTGTAGCTGATCAGGTTTTTTGGTTCTAACGACAAGTGT
GTTTATTCCATGTTATGGCTT
ATTTCCCAAATGCGTACAGTAATTTTTGAGACATTTTATAAGGGAGGTTAACTCATTAAC
GCTATTTTTCTATTCATTTT
ATTGACTTAGCACACCAAATGAATTTAAATGATAGTATTTTTTTGCTGACGTGATTTCAA
AACAAATTAAATGTTACCCA
TTTGACCCGTGGTCAATGATATATAACCCATTTTCAGATAACTGGGATGAATTTGTCCTC
TCTAGGTTTCAAGACAATTG
AGATGAATCTTGTAGATATAACATAGTTTTAGTTTCAGAAATATACAGTTCCCACAGAAT
GACTAATGTTTTTTACTTTG
TTTAGACGTTTGAGGAAGCTGATACAAAACATGATGGGAAGATTGACAAGGAAGAATG
GAGGAGTCTTGTATTACGACAT
CCATCGCTTTTGAAGAATATGACGCTTCAGTACCTTAAGTAAGTTCCATTTCTTATTAAA
GTGTTGTTTTTTTACTCTGT
AAATGTTTGGCAGTTCACACAAACTTTCGTTGTCCTATAAAGACCTACATGTGAAATAA
CCTCGAGATAATCAAAAGTAC
AGAATCAGTTCTGTTGCAACTACTCCTAAAATGATTATCCGAAACAGAATCGTTCTTCA
AGTAATCACTAATCAGATAAT
CATAATCATCTTGACTGTAACCAAACACGAGAAACTCATTATCTGCACCCACTTGAAAA
CTGGTATGATCATAATTATAA
TCGCTTTCAATACACACTGCAGAAAAGAACGCAATGCCAAACATCCCTGAACCCTAAC
GGCCTAAAGTCATTGACTCCCA
TTCATTCATTATCAGTGAGTCAACTGGACTCATCCGGCACCACAGCCATATGAATGCTG
CAAAAAGGGCACTTGGCCTAA
TAAGTAGTTAGCCATTAGCATTTGAAGCCATTATATGTTTTTCCTCACCAAACAACCAAC
GTATTTGCATAAAGGAAATA
GTTGCTTCTACTGGCAAAAGAAACTGACTAGTTCTTCTTTCTTACCCTTTTAGCCAGAA
AATAAGAAAGCTGTTAAATCT
TCAATCTTACTTGCAAAGTTTGAGTTGATGGACTGCACTCATTTAAGTTTTGTAATTATC
TGCAGGGATATCACGACGAC
ATTTCCAAGCTTTGTTTTTCACTCGAGAGTTGAGGATACATAA
>CsCML73
ATGGGTTTGAAAAATCTCTTAAATCGAAAGAAGAAAAAGAAAACAGGAGATGATAAC
AGCACTCACGAATCTGAACCTCA
ATCTGAACCCGCGTCACAAACAACAAACACGTCGAATAATATCAATGCCGAGGCATCG
CAAGCCAAGTCTTTGGATTCAC
GCGTACGTATTGAAGAGGAGCTAGAGCAAGTTTTTAACAAATTTGACGTAAATGCTGAT
GGAAAAATATGCGCGGCAGAG
CTAGGATCCATAATGGGAAGCCTTGGACACCACCCGTCACAAGAGGAGCTAAAAAAC

ATGATCAAGGAGGTGGATGCAGA
CGGGGACGGCTTCATAAATTTGCAAGAATTTATTGAGTTGAACACGAAAGATATTGATT
CGTCTGAGGTGTTGGAAAATC
TAAAAGACGCGTTTTCTGTTTTTGATATTGATAAAAATGGCTTGATCACTGCAGAGGAG
TTGTTGAATGTTTTGAGTAGT
TTAGGAGAAAATTGCACTATCACCGAGAGCAAGAAAATGATAGCTGGTGCTGATCGTG
ATGGCGATGGTATGATTAACTT
TGACGAGTTTAAGGATATGATGATGTCTGGTTCTCGGTTTGATTCTGGGTTGCAAAAAC
ATGAGATAGCGAAGGAAGATT
AA
>CsCML74
ATGTCACCCGAAACATCCAACCAATCGCCATCCGTATTCCCAACGGACAAAGAAGAAA
TCAAGACCATCTTTAACCGTTT
CGACACAAATGGTGATGGAAAGATCTCTGAGGACGAGCTAATCAATGTCTTGAAATCA
CTAGGATCCGACACGTCTCCTG
AAGAGGTCAAGCGCATATTGACAGAAACTGATACCAATTCTGATGGTTTCATTAGCCTA
GACGAGTTTGTTGTATTTTGC
AAAGGAATTGCGGGTGAATGTGATGGTGACGGGCTTAATGATCTTAAGGAAGCATTTA
AGCTATACGATCAAGATAATAA
TGGAGTCATTTCAGCTAGCGAGTTGCATCAAATATTGAGCGGAATGGGGTTGAATTACA
CGCTTAAGGATTGCGAGAATA
TGATTAACTCGGTTGATTCAGATGGTGATGGTTGCGTCGATTTTGAAGAATTTAGGAAA
ATGATGTCGAAGAATTAA
>CsCML75
ATGGCCGAAAAATTGACAGACGATCAAATTACCGAGTTCCGACAAGCATTTTCAATGA
TCGATAAAGATTCTGATGGTAA
CTTTCTATCTTTTAGCTAACTATATTTAACACCTAATAATGTTTCATGTAACAAACAAGAG
GTAATAGGGTCCGTACCGC
ACTAGCTACACATTTGAGGGTGTTCAGAGAAAACGTCGAACATAAATGAAGAATGCAC
TTAAGTTAGATGATTGATAGCT
TTACGTGAACATGTTTACTTTATTGTAGTTTTATAAAATAATTAACGTTGCGTTTTATACT
GGCAGGATTGATTAGTACA
GAGGATTTAATAGGCGTGATTCAAACATTGAACGAAAACGCTACTAATGAAGAGGTTA
AAGAAATGATGAACGAAGTAGA
TACAAATGAAGAAGGCACAATCGATTTCCATGATTTTCTTAATATCATGTCCAAAAGAG
TTAAAGTAAGTACTCGAAACA
CCCTTGAGATATACATATCATGCAAACATATGGAATGCAGTTTGTATTTACAAAAAATAT
AAATAATGAAAAGTTCGTAG
GAATGACTTGTGGTTTCATATAGCATGACCTCTAGCTTTGTAATAATCGTTACTAATTTAT
GGTAATCAACAGGAAAATG
CGAGTGACGAGCTAAAAGAAGCTTTCAAAGTATTCGATCGGAATCAAGATGGTTACAT
TTCACCTGATGAGGTACTTGTC
ACTCTCTAACCTCCCTCAAGTTTGTATGTGCTCTTCATCATAATATATGGTTTCATAGCGT
TAGTTATAAATATGAAAAT

AATGAGTTCATTAAATGTTATTTTTACAAAAATGATAAGGCTTAACCATAACCCTAGCTA
GCAAAACCCTAAACTTAAAC
ACATGGCTAATATTAGCTTAGCCGGCCCAAATTAACATAAATGGGCTAATATCTATGGAT
TGTATTGAAACTTTGTAAGA
AAATGCAACCGTAGAATATGCAAAAGTTGACATTTTTATTGACGTACTCCGTTTCATATG
ATGTGCAGTTGCGAAATGTT
ATGATAAACTTGGGGGAGCGATTAAAAGATGAAGAGCTGGAGCAAATGATGCGAGAA
GCTGATCTTGATGGAGACGGTGT
TATAAGTTATGATGAGTTCGTTCGGGTTATGATGAACTCCTCCTGA
>CsCML76
ATGTTTGATCATGATGGTGATGGTAACATCACCATACAAGAGCTATCAAAGTCTCTTGA
AAGCCTTGGTATGGTTATACC
CGAGAAAGATCTTGAAAACATGATCAAACATATTGACACAAATGGTGATGGTTCCGTG
AACATGGAGGAGTTTAAAGGGT
TGTATGAAACAATAATGGAGGAGAAGGACGAAGAAGAGGATATTAAAGAAGCGTTTA
ATGTTTTTGACAAAAACGGAGAT
GGGTTCATCTCGGTTGAGGAGCTTATGTCCGTTTTAACTTCACTTGGATTTAGACAAGG
TCGAACCATCGAGGATTGCCA
ACTTATGGTCAAGAAGGTGGATGAGGATGGCGACGGGATGGTTAACTATAAAGAGTTC
AGGCAAATGATGAAAGGTGGCG
GCTTTGCATCAATGTAA
>CsCML77
ATGGGAAACGATGATCATCTTAAAGATGCCTTTGCATTCTTTGATAAAAACAAAAGTGG
GTACATAGAGATTGAAGAACT
AAGGGAAGCCTTATCCGATGAAGATGAAGCCAACAGTGAAGAAGTAATTTCGGCAATC
ATTCATGATGTAGACACCGACA
AGGTTTGATTTTGCATCTTTCATTTCTCATTTGTTCGCTCTTTTATCTAATTTAAGAACTT
AGACTAAATTAGCATCACA
AAAATTGGAAGGGGTGATTGTTCCGGGTAGCCTCTATGTGCTTCCCCCACTATATATTTG
TGAAGCCTTTTACTAGTCTA
GTTTACCTTTTATCTGTATGTTATTCTATGGTGAGTCTATTTTAAGATACCGAACTTACCT
AGAGAATCAGTGGGCATAA
ATGGGGTTTGCTTCAATTATAAATGAGTCCTTGGGTTGCTGTGTGCATGTTGAGTTTGCT
ACTTCTAATAGTTAGGGACT
TACAGTAATAATCACTGACTATCTTTATAACATCTCAAGTTCAAGTATCCAATTTATACCT
ACCCACATCATTAGTTAGG
CATGCACATGCATAACCAAGTTCTTTTTTGTAGTACAGTGTATAAGTTTATAATTATTACG
AGATTTAATCATAGTTTCA
AGTCCATGTGGTTTGGCCATATACCTGCCTTAGTCATTAAGTTTGAAGTGTTACAATCAA
AATCCCTACTTCTGTAACTA
AATACGTGTTTTATCCTTCAGTCACTGTTAATCCCTTTCAGTTAATGCTCGCATGGGCGT
TGCACTCGAGGGTAATCTAG
TCCTTTCCGCTTCCATCTTTAACAAATATGAATTGTCTTTCTTCACTTAAGGGTATGATGC
GAACCGAAACGACGTTTTT

AAAAAAGTTTATCATGAAACTTTAATTTTAACAAATCAAGATTGCTACTATTGTCAGATT
CTCCATCATATGAAACAAGA
TATTTATTAAACCTTTCATACCTATGAACATGTCTACTTAACACCTAAAACCTTGCAAAT
TTAAGATTGCAACAACAGTT
ACCAAATTTTTAAGATGGAAATGCATGCACCTTGTCTTAATAAATGAACTCGACCACGT
ATGTAAAGTGTATATATTGTT
TATTCCTTGTTTGATATGTTCAGTTTCGACCATATCAAAATTACACTTACACATGTCATTA
AGATAAGGCACATGTGTTT
TTCGTTCTAAAAGGCCATTAAGATGATGCACATGCATTTTTGTCTTAAAATCTTGGATTG
TTGTTTTTGTTACTTTGAAC
TTGATTTGCAAGGGTTTGCGGTTACTATGTTCAAGTAACTATGTTTTTAGTATGGAAGGT
TTGTAAAATATATTGATACA
AATACTCTAGAATACGAAAATAAGTGCAATTTTAGATTGTTATCATTAGAGTTTCAGTAT
TCATATCATAATCCAAGACT
GCTTAAGTTTGAATAATACCGCTTAACTGGAACACAACTCAGATGGTTGTAAAAAATAC
GAGGGGAAGAGACAAGATTGT
CTCGTGCGTAACACACGTAAGGACGGAAATGCGGAACTGATAGGGATAGCGGTTGCCA
ACTCTTGGATCGAGAGGTGCAT
CATTTTAAAATTAGGGTCTTGACTTAGGGACTCTGTCCCAAACCTTAATCAACTATATGT
ATTATTATTTCAACCCTGAT
CATGCATACCCTAGTTAGAAGGTCACAGAACATCAGTCTTCTACCCGAACAAGTAGGTT
GACTAATTAGAAACTCTTGGC
TGATTTGTGCAGGACGGAAAGATTAGTTTTGAGGAATTCACGGCAATGATGAAGGCGG
GAACGGATTGGAGAAAAGCATC
ACGACAATATTCGAGAGAAAGATATAATAATTTGAGCTTGAAATTGTTCCAAGATGGAT
CATTGGTTTCAGCAAATGAGG
GAAGATGA
>CsCaM4
ATGGCGGATCAGTTGACCGATGATCAGATCTCTGAGTTTAAGGAAGCTTTCAGTTTGTT
TGACAAGGATGGCGATGGTAA
CATCCCCTTATCTTTATTGTTTGTGTTTGAGATTTGTTTATTGATTTGTGTTTTAATGTTTG
TGTTTTCATCTGATGTAA
ATGATGATCTGTTTGTTTAACTGTGTTGAGAATCTCATAAGGGGTGTTTGGATAAGTTTA
TTTATGTGCTATTAGGTTAT
TCTGATATTGTGCTTTGATTCTTTTCAAAACACAATAACCAAACACACCCTTATTGGAA
ATAATGTTAAGAATCAAATAA
TTTAGTACCTGAAGGCTTTTTTTGTAGTTGAGAATTGATTTCATTATTTGCCGTAACGTT
AAAAAATTTATTCCACCCAA
TCTCACCTTTCAAACAACGTTAAAAAAATTACTCAGTTATAACCGTTATCACGGGAACC
AGCCTCTTCAAAGGGATGGGT
GTAGTAATGTGGGTTTGTGCGCTTCGAGCTGTTTTCAGCGTCACATCTCATCTCATAATT
GTTTTTGAACACCATTTTTA
TGTTTGACGGTAACGATGTCTCTTGCTCCACTCTACATTCATGAAACATGAGAAAATAA
TTATTACATGTGCATCAATAG

ATCAAGTAATTGTGCATTATAGTTAGGCTGATCCTTGGTCAAACGCGTAATTCCATTCCC
TAGGATATTCCCTTTCATAA
TTGTTGTTCTAGTCTCGTGAATGCATCAAATGTTTCCTCATGCGATGCCTCCACTTTTTA
CAATGGCATTTGACGTGTAA
CAAAAACATAACTACCTTACCCTAGCTCTCATTACACATGTATTTATGGCCATTATGAAC
ACATTACAATGGCAGTGTAC
TTTATAGTTAGACGCTTGACACGTCGCTGTTAACTAACAGTTAACCACCTTGTACTTTG
CATATAAAAGAGAATGTCTCA
AAGGAGTAAATGCCGTCTCGTGCAATTAATGTGATACACATTCACGAGAGCATTTACTC
CTCCAAAGAACTAAGTCCCTG
AATCTATTCGAAGTGTTAGCATAGTTCTTACTTAAACACCTACTTTTGTTAGTGTTTTAA
GTTAGTGAGGAGATCGCCAT
ACATTGTCATTCAACTTAGATTTTATTCCGCAAACAATTTTAGTAAATTCTTTGATTGTTT
TCTAATAAAAATTTAGGGG
CAATTGCATATATATCCCTCCAAAGACTAAAAATTGCATATATATCCAACATAAACTTAAA
ATTGCGTATATATCCTTCT
TAAACAAAAAAAAATATCACATATATTCAAATAGTTATTTTATTTATAATTACAACACAAA
AATGCAAGATAATTAAAATT
TAAATACATAATGACCATTATACCCTTTTATTTATCATTTCTCATAATTAAAAAATATCATC
AAACATTTTTTAATTTTT
TTTTAAAAAACAAAAAAAGAAAAAAAAAAAACTTCACTGAACACCAAAATGACACA
AAAAAAAAATCATTAATAATTGAC
TCATTTGAGCGTATATCATTAAGAATTAATTTTTAATTCATCAAAAAATTAGATGATATAT
CAAGATGTTTGATGTGGAC
TCATATACAGTTTATGTTTTCATTCAAGTATTAATTTTTAATAAAGATCGTATGGTTGTAA
ATATCTATAGGTTGTGTGT
GTTTTTGGCAGTTGGTTATAATCAATTAAAATTCAACAATAGGAAACATACGAACAAGC
GAAAACAAGAACAATAATGAA
AAAATTAGTTTGAAAAGTTAGTACAGTCAAACCAATTTACGGGATTCAATCTAGAGTGC
AGAGTTAATCTGTGTGTGGAG
ATTGTTATATTCAAACTTGTGTATAGATTTATTATAAGTAGAGGATATAATGGTCATTTTGT
ACTTTAATTACAATTATT
ATGTGTTTTTTGTTTTTAAATATATAGAAAATAATTATTTGGATATATATGACAATTTTTGT
GTTTGGGAAGGATATATA
CGCAATTTTAAGTTTATGTTGGATATATATGCAATTTTTAGTCTTTGCAGGGATATATATGC
AATTCCCCCAAAAATTTA
AACGTTGAAAAACACTTGTGCAATTCTTTCAACTAAACGGGAACAACAATATGTTTAAT
CGTTATTGCGGGATAACATTG
CTTAGTTGGCTTTATACTGTGATAATATGATTTGCTTTTAATTTTATACTTAAAATGCTACT
TAATACTTACTCCTTGCA
ACACCTTTTGTTAGATCATTAAATTAAATGGTCTGATCATTCATTTGTGAGGCTATGACT
ATCCCCTTGTGTCATGCGTG
TACATTTTGACCCGTTTACATAGAATATAGTGATTGTTGGGAAAGTTCCTCAATGTCAAC
CTTCTATTTTGCCGTATTCA

AAGATTTAAATTATTATTATTTTTCTTGCAATCATAATTAACTCACGAGTTATTTATACCCT
TTCTATAAATATCGGATC
AAGTGTTTGGTGGGTCAACCCAACCTGGCTTGTTCGAAATGTACTAAAGATTTACTGTT
TAAACCCAAACCTGTTTTTGG
ATTTGTATTTTGGGGGAGATATTATCTGCGATATATGTGGAGAGAACTAAAATTATAAAA
AAATAGGTTGAGACTTGATT
TTTGAAAATTTGGTTTTGTAAAATCCAATCCCAATATCCTCGAGCTCATTATACGGCTGC
GTTTAATCAGAAAACAGTTC
TATTCTTTTTTTAGAACTTGTGAGTTGACAGTGTTATGTTTTTTAATGGTTCTCGTGGAA
TTACCTATGTTCAGGTTGCA
TCACCACAAAGGAGCTTGGGACTGTGATGAGATCCCTAGGGCAGAATCCCACAGAAG
CTGAACTTCAGGACATGATTAAT
GAAGTTGATGCTGATGGAAACGGAACCATTGATTTCCCTGAGTTTCTAAATCTTATGGC
CAGGAAGATGAAAGATACTGA
TTCAGAGGAAGAACTTAAGGAAGCATTCAGAGTTTTTGACAAGGACCAAAATGGCTT
CATTTCTGCTGCTGAATTGCGTC
ACGTTATGACAAATCTTGGTGAAAAGCTTACTGATGAAGAAGTTGATGAGATGATCCG
AGAGGCTGATGTTGATGGTGAT
GGTCAAATCAACTACGAGGAGTTTGTTAAGGTTATGATGGCCAAGTAA
>CsCML78
ATGAAGCTTCCCGCCAAAATCAACCCTAAGCACATCTTCCGATCAAGAAAACACAAAA
CAGTCACCAGATCTGATCAATC
TTCATTCAGTTCCTCCAATACGACGTCGTCAGACTCGCCAGAATCCAGTCATCACCGTC
GCAAGGCCAATACTTCCGGCG
TCACTACTCCGACGAGCGTCCTTCCTTCCTCCGCCGACGACTACTCCGACCTTCAACTC
GACCTAATCCAAGCCTTCCGA
TTCATCGACACCGACGGCGACGGAAAAATCACAACGCAAGAACTCGAAACGATCCTA
AACCGTATCGTAAGATCTGAGCC
GTTGATTCAATCAGAGCTGAAATCGATGTTAACCGAGATAGACAGTAACGGCGACGGC
GTTATCACTTTAGAAGAATTTG
GAGCGGTTAGTGAAGCTTTTGGACCGGCGGTTGGCGACGGAGAGTTAAAAGAAGTGT
TTGAGTTTTTTGACAGAGACGGT
GACGGAAAGATAACGGCGGATGAGCTGTATGAGGTTTTTGTTTCGCTAGGTGATGGGA
AGGTGACTGTGGAGGAATGTGT
TGGCATGATTAAAAGTGTTGATGTTAATGGAGATGGGTTTGTTTGTTTTGATGATTTTAG
GAGTATGATGGAACAAAGAT
GA
>CsCML79
ATGAGCACATACAAAGACCGAAGTTGCCGCGGTAAGATACCCTTTTATTTAAATTTTAC
GTCTTTGTTGTACAGTATCGT
TTTGATAGTTTTGGGGCCTTGAAAGTATCAATAATTGATAGAAATTTCACTCGTTTATAA
CATTCTTCGTTTATAACATT
CTTCGGTTATTTTTGAAAACAACATCGATTTTTCCTAAATGCATCTTAAATGCACACAGT
CCTTTAAAATGTGTTTCCAA

TATTACAGTTCAAAAGATTTTGTGCACCCGTTCATATTTGTTCCAGGCTCCGTCCTATGA
TTAGGACATTTGACCGTATT
GCTTGTGTTTGACAAGGGATGTTTTTTCCCCACTTAATGAGTAGGCTTAAATCTTTGTTT
CTTTTTATCGGGGAGGTTCA
CAAAACTTTTCTTTTCAATTAAAACGCTAAAATAGAAATGTTTCAGAACCGCTTAATTT
TAAAAACAATCTTTTAAAGTT
TCATCACTCCTCAAGCCATGTATCTAACTAAATGCCATTCACAAATTATAAATTTTCAAA
TTTTTGTGTGGTTATATTTT
AAGTTTCTTTTTGTTTTCACAAGGGAGTCGTGTATAATTTTCCTTGCTTTCCCTATTTGTT
TTACCTTAAAAACAAGAAA
TTATATGATTGAGAACATGGTCTAACATGCTTTGCTAATTCAGTAATTCTAGCTCTAGAA
ATGAAATTGATAAAAAGTGT
AGTTAGCGTGAATTTTTTAAACTACGTGCATTCTGAAAGTGGACTAATAAATTAAGACG
GAGGGAGTAGAATTGCTTCCC
CAATATATATTGTTCATTTTGTAGAGCGTTTTCTTGATCCTTTTCACATATACTCAGTAATA
AATAATTGGTATAATTAG
ACTTTCAGTTTCTATCTCACCAAATACATAGGATAACAATATTATCATGTCGATTGAGGT
AGAAAATAGACAATTATATG
AAAAAGTCAAGAAGTACATGTAAACATGGCATGCCCACTTTTTTGCTTTGCCCTTTTCT
CATAATAATGTTTCAACATCT
ACCACAACTTGTCTTCAATACAATTCTTGATAATCATTTAGTTTCTTGCTTTTTCTCTAGC
TACATCTCCAATGCCTAAA
TCCTTGCAAACACGCATAAGGAATCTCCTTAAGAGAGTCAACTTCTTAACCAAAGTCA
ATAACTTCAAGAAAAAACCACC
CAAGAATCCTTGTGTTCTAGATGCCATCTCATCATTTATAGCCATGGATGTCTCAAACCA
ACTCAAGCAAGTCTTCAAAT
TCTTCGATATCGACGGTGATGGCAAGATTTCTCAAGTGGAGCTCACAAATGTTTTGTTG
ACTTTTGGTCAAGAGAAGTCA
ATGGCCACAAAAGAAGCTCAAGGAATACTTAAAGAAGTTGATTTTAATGGAGATGGTT
TCATTGACTTGGATGAATTTAT
GACCATTATGGATGGTTCCAAGCCGGTTTTCGCTAGCTCAAAGGAAGATAATGGTGATG
ATGATCTTAGAAATGCTTTTA
TGGTTTTTGATAGTGATAAAAATGGACTCATTTCTGCAAAGGAGTTGCAAAGTGTGCTC
ACTAGTCTTGGATGTAGTAAT
TCCAAACTTGGACAATGTAGAAAAATGATTAAAGGTGTTGATAAGGATGGTGATGGATT
TGTAGACTTTGATGAATTTAA
GTCAATGATGTCCATCGGGATTAAGTAG
>CsCML80
ATGGCAGAAAAAAGCTACCAAGACTTGCTTCCTATCATGGCTGAAAAGCTAGAGTTAA
CAACCTTCATGGAGGAGTTATG
TAGCGGTTTTCGTCTGCTAGCTGACGAAAACACCGGCCTGATCACCCCTGAAAGTTTG
AGAAAAAATTCAAGTATATTGG
GTATGGAGGGAATGAGCAAAGAAGATTCAGAAGGTATGGTGATCGAAGGAGACCTTG
ATGGAGATGGTTTCTTGAACGAA

ACCGAGTTTTGCATACTCATGGTGAGACTTAGTCCCGAAATGATGCAAGATGCTGAGAT
GTGGTTGGACAAAGCGATTGA
AGATGAGATCAAGAACGTCTCTACTTCATTACCAGATAATAAAGTTTAA
>CsCML81
ATGGCATCAACCAATAATATGCAATCTGAGTTCCAAGATTACTTGCCATTAATGGCTGAT
AAGTTGGGTGGTGATGGTTT
AATACAAGAACTATGTAATGGGTTTCAGTTACTTATGGATCAAGATAAAGGGGTGATTA
CTTTTGATAGTTTGAAGAAGA
ATTCATCAGTTTTGGGGCTTGAAGGATTGAGTGATGATGAAGTCATGAGTATGCTGAAA
GAAGGTGATTTTGATGGTGAT
GGTGCTTTGAATCAAATGGAGTTTTGTGTTCTTATGTTTAGATTAAGTCCGAATTTGATG
GATCAATCTGAATACTTGTT
GGAAGAAGCTTTGGAACAAGAGTTAAATAATTTTCAATACTGA
>CsCML82
ATGAGTCAACTAAAATCATCGTCGTTTCGTCTTAGATCACCCTCGTTGAATTCTGTCCGT
TTACGTCGTATTTTTGATTT
GTTTGATACCAACCATGATGAGTTAATCACGGTTGATGAACTTAGCCGCGCTTTGATAC
TTTTGGGACTTGATACTAATA
TGAATGAATTGGATTCGATGATTAATACGTTTATTCAACCAGGGAATGCTGGGCTTACGT
TTGATGATTTCCATGCTTTG
CATAAGGAAATTGATGATTTGTTCTTTCGTCTTGATGATAATGATGATTTAGGTACGTTG
TATATATGTCAATTATTAGT
TTTTATGATACGAGTCGTTATATAACTTCTAATGTTTTCGTTTATTTGCTAAACGAACGTG
AACAAGATTTTATGTTGGT
TAGCAAATCAAATAAACTTACGAAATAGTTATAAGCATCGTTCCTTCATTTATGTTTATG
AAAGGTGGCTAAGGTTTTCA
TGCTTTTATATTATATCGATATCACATCTTTTAATCATAATTGAATTTATATGTTCATTTAAA
TGTTTATGTTCGTTCAT
TTTTTCGACTAAACTTTAACTAGTTGACACAGAGGCGGATCCATGTGTTATCAAGCGGT
TTTCGGGAACCACTTTGGACA
ACGAAAACTTCTAAAAGCGTTATAGAAATTTTCTTAGGAACTACCTTTTAAAATATTTG
GGAACTAATTTTAAAAAAATT
GGAAAAATACTTCACTTTTAGTATAAAATATTCTTAACCCAAATCTTAAATAGCCCAAAA
AAATTAGTCCATTTAACAAT
TACTCCGTAATAATTAACTTTAAGGGCATAAAAAATCCATTAATCGGCAATTCTAGTACT
GTAGAAGGAATTAGGGAAAC
GAACTCGGATAGTCAACTCACTTCATTCAACAATCCATATGTACGCTATTTGTCTTTTAC
GAGTAGTTTATTTATTCATT
CTAGTTTTTATGAATAATTAAAAGGAATAAACAATGTTATGTAATGTACACTGTATTAGTA
TAATTGTTTGAAGATTATT
TAGATTTTAGGGTTTGTTGTTTAGGACTTTAGTCTTTAGGTTAATATGTTATGCATCATGT
GATTGTTGATTAAAAGTTT
GAATTTTGGTATTTGTTTTAGTTACAATGTTGAAGTTCTTAACCGAAAAAGGAGCATTT
AGTTCAATGTTTTATTATCGC

ATCTTGTTAAATTGGACCCGATCCGACCCGAAAATTTGACCGGTTGGTTAGTTGACCTA
TTGCAATTTTTAATTTGATCA
AACCAAGGAACCACTTTGAATTTCCGGCTAGATCCGCCTAACTGTTGACACAAACAAG
TTTCGTTCACGATTTTTCATTT
CGTTTACATTGATAACTATTTATAAAACATGTTCTAGAATTTTAGGGTGGAAAAACTAAT
TAACTATGTATCCTTTTTAT
GAAAATAGGTAATCAAGATGAAGATAACGACGAGGCTAATGGCGATAAGCAAGAGGA
GGCGGATCTAACTGAGGCATTTA
AGGTTTTCGATGAAGATGGAGATGGCTACATATCAGCGACCGAGTTACAAACTGTGCTT
GTGAAGTTAGGGTTTGCCGAA
GGTAATGAGATTGGAAGTGTGGAGAGAATGATATCATCCGTTGATCGAAATCATGATGG
ACGTGTTGATTTCACAGAGTT
TAAAGACATGATGCGTAATGTGATCGTTCTCAAGTAA
