# Supplementary material for: Genome-Wide Identification and Expression Analysis of CsCaM/CML Gene Family in Response to Low-Temperature and Salt Stresses in Chrysanthemum seticuspe
Source: Plants (Basel). 2022 Jul 1;11(13):1760. doi: 10.3390/plants11131760 (PMC9268918; doi:10.3390/plants11131760)
Supplement: Supplementary file 1 [file plants-11-01760-s001.zip › Supplementary File S4 Promoter sequences of CsCaM_CML.pdf]

>CsCML1

TAGTGGATAACCGATCAAACCGAGAAACCGACCAAAAACCGAAACCAAGAAAAACCA
ACGATTTCGGTTATGGTTTTTAA
AAAACCGAACATTCGATTTTCGGTTTCGATTTTATGTATAAAACCGAACTAAACCGAAC
CATGCTCACCCCTAATGGAAA
GTTACAGTATCCAATATAAAATTCAACTCAATATATAACGTGGTAATAAGACTAAAAAGT
CCTTATTGGAAAGTGATATG
ATAGGTTGTCACACACCACAAATCTATTATTTTTATTTAAATAAAATAATGACAGTTGAA
GGATATTTTAATGAAAATAA
AATGTACAGAAATCTCGTCCAGCTCAATCGTATAACTGTAACCTTTTCTGTCTCGTACTT
TAAACTTTATACCAGAAAAA
AAAGTCTACAAGGCCGATTAATCACCGATTAGGTGCTACTGGGCCACCGACCTTGCCG
ATTTTTGTAAACCGTTCAAACA
AATCGGTCAACCAGGGTCAACGGCGGTTAACGATGGTCAAACTTGCCAAAAACTTATA
AATCTTGTTGGAAATACCCTAA
ACTCACTGTAAATTAGTTTTAGTTACTTATAGTCACTATTTATATGTATTACAAGATGTTTT
TCAAATAAAATCTTTCTG
GAAATACCAAAACTCGCCGCAAATACCCAAAGCTTACAAGAAATGCCAATATGATTTAT
CACCAATTCGCTATTTTTGCA
ACCCTGAACAAATGTAATCACTTATAGTCGTTGTTTATATCTATTACAAGATGTTTTTAAG
GAAAATCTTTCAGGGAATA
CCCAAAAGTCGACGGAAATACCCAAAACTCGCCAAAAATGTCAATCCGATTAATCACC
GATTAATCTCGCCTAGACCGAT
TACAAGCTACAGGGGGTCGACCGCCTCACTACCGATTCGTGATTTTTGCAACCTTGCTT
TATACAACTCATTTTTTTTTT
CCTACACATACAATCTTTAAATTTAACCTAGTGCTGTGTGTAATCGCAAACGTTCGTTAC
ACAAACAAGCCGTAGATTTA
ACATAGGGATTGTTTCTGTAACCACAAGTAACGAGACTACGAGAGGAGCGTATTTTCG
CAAATTTAAAGACGTTTTTGAA
AACATACGTTAACCGAAAGGACTTTTGTAATCAAGAGTAAACGATAAAAATAGTCTTTG
CAATTTTTCAAAAGACTATTT
TTCTAATCGTAAGTCTACCACACACAGGGTCCATTTTTGCTAGATGTTCTTTATTTGACA
TATATTTATATTTAAAATTA
AAATTAACATGATAGTTTTTTTTTTTTTTTTTTTTTTTTTTTTTTTTTTTTTTAATCTGGGTT
TTCGATTCGATATTACGA
GCCAACTACCCTTTTGGATAGCCCGAAGCACAAGTGAACCTCCGTAGCCAATGAAAGA
TTAATTTTTGATACGCGCCAAT
TTGGAAACTACTGATAAGCTTCAGTGGAAACCACTGATAAGCCAAAAGTTAGTTGTCG
AAAGGTTGGGCTAACTCTGAAA
CCAATTGATACGAACCTATCCCATACAAGGCTCGTATCGATGTTCATAAGTCCAAATGTA
AAACTCACTCAAAAAGCTAG
CTTGATGAGGAGGGGACACTTAGACATTATAAACCACCGCCCAATCCCATACTTGGGCG
ATGTGGGATCGTAACAATTTT
CTCCGTAGAGTTCGAACCCAATACCTCTAAACATTAAAATGATAGGTTGTCACAACACA

AGTCACAACTTACAATATCAT
ATACATATTTATTTAATATTATTTCTAGAATAAAATAATGACATTATTTTAGAAGGACAATA
TATATGAGTAACTCAAAT
TGATGTTTTTAATGAAAACAAACATCTGGTTCCTGAGTTCCTGTCTGACTACAAAACAA
AAATGCTTCTTCTTATAACAA
GACTCTTTCTAACAATTTAGTATTCTTTAGTAAAAAAAAAAAAAAAAAAAAAAGAGCTC
AAAAACATTCTATCTTGCATGC
>CsCML2
GCCTTTCCCTTTTTATTAAAGAGAATTGATTTGCACATTTAAGGTTTTTGTTAAGATTTA
AGATCATTTAAGGAGAAAAA
CTTTTTTATATACTAAGGGATCAATCTTCACCTTCTATTTTTATTAGATCTAATGGATGTGA
TTAATCTAAACAATTAAA
AAAACTCACTTTTATCATTTTTCTTTAAATATATATATCTTTCAAACCGTTAATCGTCAAG
CGAAAAAAAAAATACCATT
ACGACGAGCATTTAATTATCTTTTTATAGATAGGCACTTTGATATACTTTCGACAAAAAA
ATTTTTTCGTGTTTTTCACG
TTTTTTTTTAAGTTTTTTTCATTTTTTTACGTTACTGAAATTTGCATGAAATATATGATTTA
ACACTAATTTGAAGATCA
CATGTGATATTAGTGTTAAATCACATATGATTTGTAATATCATATGTGATACTAGTGTTATT
ATCATATATGATTTAACA
CTAGTATCACATATGATATTACATATCATATGTGATTTAACACTAATATCACATGTGATCTT
CAAATTAGTGTTAAATCA
CATATAATATTTCATGCAAATTACAGTTTCGAAGAAAAACGAAAAAAAGACGAAAAAA
GTGAAAAAAACGATTTTTTTT
TCTTTTTTCGAAAGTATATCAAAGTGACCGTCTATAAAAAGATAATTAAATGCTCGTCGC
GATGGTGTAATTATTTTTGC
TTAACGATTAACGGTTTAAGAGATATTTAAATTATAAAAAATAGATGAAATAATAAAATG
ATTTTATTCTCAACCATTGT
TTTATTTTTAGATAGATGATGAAGATGAGTACCTTGGTATGTAAGAAAAGTTTGGTATTT
GATTAAGATACTCTAAATTA
TGCATAATATAATTATATAATATTTACAACGTGTTTTGTATAATTATACAATGAATGTCCCA
ATTAAAATAAAAAATATG
CAAATCCCTTTTACTAAAGAATAAATAAATTGTTATTAACTATAACAAATTAACAATAATT
CTTTAAAAATAAAATCTTT
GACAGAAACAATTAAGTCAAATATTATCAAAGGTATATTCCCTTCAACCCCAAGAAAAG
CAAATCACAGACACACACACA
CACACTTTTTCAACACACGTGCATGTCACGTGACCCTATTTTCACCATATATAAACCCAA
ACCGTTTAATACCCTCCTCA
CATAACCAAACCAAACCCCTCTTTTTTATAACTCTCTGTGTGCTGTATTTTCTTACACAA
TTTTATAAAATCAAAACCCT
AATTCCTTCAATCACAATAACATAAACTAAATCCACACACAGTTACACTTTCACTGTGT
GTTTGTTGACCAGATCTTTGA
CTTTTAGACCGCTTTTTTAGCTGGTATAAATAATGAACTTGTTTAATTTTAGTTTAATTTG
ATTAAATTCAGTTGACAGT

TGATAATATCATATTTAGGTTAAATTTTTGATTTTTGATGGTTAGTTAGTGATTTATGCATG
TTTAATTTGTTTGTGTTT
GGGATTATTGAGTGAAAAATTGATTTAGGGTTTGTTAATTTTAGTTAGATCTGTGATAGA
TTAAGTTTAAAGCCTTTTTT
TTTGTTGTTATAATTAGTTTATGTAGATTGGTTAATTTGAGCACTAATTTAAGTCTAAGTA
CATTGTGGTTGGAAAACTA
TGATGAGGTTATTAGGTGTGCGTAAACTAGTATGAGTATGTATGTTGTTAATCCTAGAAT
CCTCGATTTTAGATGAACGG
CAAAAATTGGAAGTTCGTTTTTATCCTTTTTTACTTTCCTGTGATTCTATAGATGTACGTG
TTTTCCATGTATCAAATGA
TTCTATTTAGCTAAGTTGGTAATTATCCGTGTGGTTTATATTTTATATTGGTTAATTGATTA
GTTTTGTGATTTACCATT
GTGGCAGTTAAGGTTGAGACATGTTTCGTAGCAGCAATAACGTTGCTGTGCTTTTAGCA
GTGTAAAGCTCGTGTTGTCTA
>CsCML3
AGAATGTATGCTAGTATGTCAATTTTGAACCCAATTTTATATATCACTACATCTGTAGCAA
GATTTTAAATGAGGAGTAC
AAAAATGTACAAAGACATGGCTAGGAATTTGAATTGAAGATGATATGTTCGGCTTTAAT
TGCCAAAGATAACAACTTGCC
AACTAACTGATTATCTGACGCAAATTTGTAGAAATTCTCTAAGAACTTCTCTCATTGTCA
TATACGGATATGTCATAAGC
TAAAAGCTGTTTTTTTAGCAAATTATTCTATGAGTAACTAAGATCATATATTTCTTAATTA
AGAACTTCCTCTTAATTAT
CGACGCATTAAAAAAAATGCTAAACAAGTAACGAGTAGATCGAAGACTATAATGCTAA
TTGATCATGTTACAACTAAGTA
ATATACCAATAAATGTCTTGTTTGACAAAAAAACGCCGTTTTCAGCTACACAAATATAA
ACAATTAAAACAAATTAATTA
CGAAACATCACATCTTATTTAGCATGCCCGCTTGCCCTATCCACAAAATATAGAATCGTA
AAAGGTTAGTGATTACCAAG
CTCTTAAATAATCTGTAAGTTTAAACATTTACCCGTTATGTTACATAAATAACCTGGATAA
GTTCAAAGAAAAAACCTAT
ACAAAATAACTTGCATTACACTAGTCTTTAACAAAAGGGATTAAGTCGTAGAATAACAA
TATACTTTTTAAAATATACCA
TTTTGGTACGTGTACTTTTTAAAGTTATAGAATAGTAATGTACTTTAATAAGTGTTTTAAT
TTGGTACATGACGATGACG
TGTCATGCTGTAAATGCTGCCACGTGTCAAACTTTTGGATATGACGCGTCATCGTCCCG
TACCAAATTAAAACACTTATG
AAAATACGTTGCTATTCTACAACTTTATAAAAATACACATCCCAAAACGATACATTTTGA
AAAGCATAGAAGGAACTATT
ATTTCGCCTTTTTTACTAAAATATAAATAAATAAATTGTAATTATTAAAAATAAAATCTTT
GACAAAAACAATTAAGTCA
AATATTATCAAAGGTATATTCCATTCAACCCCAAGAAAAGCAAATCAAAGACACACACA
CACACTTTTTCAACACACGTG
CATGTCACGTGACCATATTTTCACCATATATAAACCCAAACCGTTTAATACCCTCCTCAC

ATAACCAAACCCCAACCCCT
CTTTTTTATAACTCTCTGTGTGCTGTATTTTCTTACACAATTTTATAAAATCAAAACCCTA
ATTCCTTCAATCACAATAA
CATAAACTAAATCCACACACAGTTACACTTTCACTGTGTGTTTGTTGACCAGATCTTTG
ACTTTTAGACCGCTTTTTTAG
CTGGTATAAATAATCAACTTGTTTAATTTTAGTTTAATTTGATTAAATTCAGTTGAGAGTT
GATAATATCATATTTAGGT
TAATTTTTTGATGGTTAGTTAGTGATTTGTGCATGTTTAATTTGTTTGTGTTTGGGATTAT
TGAGTAAAAAATTGATTTG
GGGTTTGTTAATTTTAGTTAGATCTGTGATAGATTAAGTTTGAAGCTTTTTTTTTTTTTT
TTTTTTTTTTTTTTTTTT
TTTTTTTTTAATTAATTAGTTTATGTAGATTGGTTAATTTGAGCACTAATTTAAGTCTAAG
TACATTGTGGTTGGAAAAC
TATGAGGAGGTTATTTGGTGTGCGTAAACTAGTATGAGCATGTATGTTGTTAATCCTAGA
ATCCTCGATTTTAGATGAAC
GGCAAATTTTGGAAGTTCGTTTTTATTCATTTTAACCCCCCTGTGATTCTATAGCTGTAC
GTGTTTTCTACGTATCAAAT
GATTCTAGTTAGCTAAGTTGGTAATTATCCCTGTGGTTTATGTTTTATTAGTTAATTGATT
AGTTTTGTGGTTTACAATT
TTGGCAGTTAAGGTTGAGACATGTTTCGTAGCAGCAATAACGTTGCTGTGCTTTTAGCA
TTGTAAAGCTCGTGTTGTCTA
>CsCML4
ATATATCTCTCTCTATGAGTTGATAGATATCAAAAGTTTTTAGATCTAGATCTAAAAACTT
TTTAGATCGTGATGTTGCT
ATTGTGGTGATGTTGCAATTGTGGTGGTATCTGTTTTGTGGTGGTATCTGTTGTGGTGGT
ATCTGTTTTGGTGGTATCTG
TTTCGTTTAAGTTTTTAGATCTAAAATTTTTTAGATCTTGAAAAAAAATAGAGGTTGGTT
TAAAAAAATCTGAGGTTGGT
TTTCTTTGATTATCTAAAAACATTTTAGATCTAAAAACTTGATCTTGAAAAAAATTTGTG
GTAGTATCTGTTGTGGTGGT
ATCTGTTTTGGTAGTATCTGTTGTGGTGATATCTGTTTCGTTCAACTTTTTAGATCTAACA
TTTTTTTAGAGCTTGAATC
TTTTTAATCTCTTGAAAAAAATTAGAGGTTGGTTTAAAAAATGTTGAGGTTGGTTTTCT
TTGATTATCTAAAAACATTTT
AGATCTAAAAACTTGATCTTGAAAAAAATTTGTGGTGGTATCTGTTTGTGGTGCTATCT
GTTGTGGTGGTATCTGTTTTG
GTGGTATCTGTTGTGGTGGTATCTGTTTCGTTCAAGTTTTTAGATCTAAAAATTTTTTAG
AGCTTGAATCTTTTTAATCT
CTTGAAAAAAAAATTAGAGGTTGGTTTACAAAAATTTGAGGTTGGTTTTCTTTGATTTTC
TAAAAACATTTTACATCTAAA
AACTTGATCTTGAAAAAAATTTGTGGTGGTATCTGTTTGTGGTGGTATCTGTTGTGGTG
GTATCTGTTTTGGTGGTATCT
GTTGTGGTGGTATCTGTTTCGTTCAAGTTTTTAGATCTAAAAACTTTTTAGATCTAAAAC
GCGTACACACCACAAGAATG

```
GAAAGCGTACAAACCACAACAATGGAACACGTATACTACTCTTTTATAAATAAAAGTTA
ACTCTTTTATTTATAAAAGAG
TATTATAAATAAAGTTTATAAAATTTGGTGGTATCTGTTTCGTTCAAGTTTTTGTGGTATA
AAACTTGGTCTCTCATTGT
TATCTGTTTGGTTATGTTTTGCAGGTTTGGGGTGCCTCAATAGCATGGCCCTTGCAGTTG
TTTCATTACCATTACTGGAT
GGTAAAGTGATCATTTTTAGCACTTTGCAACTTTAATGAAAAAAACTTGAATGTTTTGG
CATGGCACGCTTCATTACAGG
ATGGTAAAGTGATCATTTTAGCTCACTTTGCAACTTTAATGAAAAAAACTTGAGCTAAA
AAAAGGCAATGATGATTTTTT
TATAAAAAGCTTAAGAGTTTTGGCATGGCAAAACTGAATTTGCAGCCAACATGAGCTA
AAAAGCTAATAATCTGAGCCTA
TTCGATAAGTTTACCTCTTGTGTGTCAAATGGTGACCTAGACATGGTTGTTTGTTGGTG
CAGTTGTTGCTTTTTGTTTGT
TTGGTGCTATTGGTGTTGTTGTGTTGTAGCTTTCTGTTGGTGCTAATGGTGTTGTGGTGT
TGTTGCTTTCTGCTAGCTTA
TGAGACTTTGTGTAGTTTGTGTTGTAGCTTTCTTGTTGGTGTTGTGGTGATGTTGCTTTC
ATGTGGCATTATAAGCCTGT
GCCTGCTATGCTGGTACCCCTTTGAGAATCTTAGTTACTTTCTTGTGAATGTTTGCAGTT
TTGGAATCCTACTTACCACA
TTCATGTGGTTGAACATGTGCCTGCTATGTTGAAAACGTGTAATTTGTGAATATTGTTGA
ACTTGTGAGGCTTGTGTCCT
TGTGAAGTTTGAGACTTGTGAGACTTTGTGCAGTTTGAGACTTGTGAGACTTTTGCAG
TTGTGGACTTGTGAGACTTGTG
CAGTTCCTGTGAACTAGTGACATTGTGCAGTTCCTGTGAAATTGTNNNNNNNNNNNNN
NNNNNNNNNNNNNNNNNNNNNN
NNNNNNNNNNNNNNNNNNNNNNNNNNNNNNNNNNNNNNNNNNNNNNNNNNNNNNNNNNNN
NNNNNNNNNNNAATGATCTTGTACAC
>CsCML5
CCTAATTTATTAAAACTAGTGCTAGTGAATCAAAATTTAAAATTTTAAACTTGATTCGAA
TCAAAATTAAAAGATGATCC
AGTTGGTTTCATTTTAGGTGACAAAGAAATTGGTTTGAGTGCAAACTTAAGTTCTCGA
GTTTTAGTCTTGTCGACAAAGC
AACCACTAGTCATTTAACAATTATAAATGTTATGTTCAAGTTATAAATTATGCATTTAACG
AGGGTTGTACTATATAAAC
CCTCTCTATGGGGTTACATCCATAGGGAATATTCCTTTTGTCTCCTTGTTTTTTCAGTATA
ACGCAGTTTTTTGTCAGTT
TCAGTTCCGTATTTCTGACAGTTTTAAGCGTTTGGCTTATTAAACTTTATATTTACAGTTC
CGCATTTTCAGGTTTATAG
CTTTTTGATATAAACAAAATTATAAAAATAAAAAAAAAATAAAAAAACAAAAAAACTA
AGGGGGGTCAATCTAAAAAACT
AGGTACCGTCCCAATAACCCAACCCTTTAATGATTCTACAACATTTTGTGGTTATTTTCT
TAATAAAATCACTTAATACT
TCTTAGTAACTTATAAATCTGAACTATTAGAAGCCTTTAACCATTAAGGGTATTTGTTGC
```

TTTTTTCTTTCGTTTCGACA
CATTAAGATGATTTAACCCTTTGCTTAACAAGACTTTGAAGATGATCTTATTACTTCTTA
ATGATATATTAACTAGTTTT
TTTCCGTGTTATAATCATTTAGACTTTTAAAAAACAAACAATATCATTTGATTCAGATTC
AAAATTTTCCGCCACAGATT
TACATTCAGATGTACGAATCAGATTCATACTCATAGCCACTTAACAAACAGCACCACAT
ATCTTAAAAGGGAAATGATTT
GCACACATTATGTTTTAATTTGCCCACTCATTAGTACAACCTGTTAAAGCAGTGTTATAC
ACAGTACAACAGTTTAATAC
ATATTCTTGAGTGTCCCTATCAAATAATAAATTGTTCAAATCATTTCCCATATTAAAATAT
CCGATATATTTTTTTCCTC
ATTATGGAGTCACACATCCCAAGGTTTTCAAAATCGGGATCCTACTCAGGATCGTTTTT
TGTTCTTGCAAGATCAGAATC
GTAATATCGGATCGGGATACTAAGATCTTACTATAATGTTATTTTCTTAATAATCTGTGTA
ATATTACTCAAATCTACAA
ATGTTTATATTCCATAAACATAAAACTATATGAATGTTTCGAAGAAATGTTTTCTTATGTA
TGAATATTGAATGTTTAAG
AATATTCACATATTGTTTTTTAAATTCTATAAAAATATAATCAACAACTTTGTAGTGTTTT
AATTTGCAGTTCGAGAAAA
GTAAGATTGAATCGGTTATATCTTAAGATTTTATAGTTAAGATCGGGATTCGATAGGATCT
TAAGATTGTTAAGATTTTA
TTTAAGATTTGAATCATTTTTGCTTTATAATATCATAAAATCGTAAAATACTAAGATTATTA
TCGGGATCTCATCAGCTA
GGACGCATTCATTTAAGCACACGCATTCAACGTATAATACTACCCAATTGGATTCTCCGT
ATCCTAGAAATAATGATTTT
TGCACAAATTTTAAGAGAATTACTCCCCTCACCCTCACCACATGACCCCACCTCACCCA
ACGGCACTTTCTTTGCATTAT
AAAACTCACCACACCCCTATTCATTTCCCACAATTCATCTTTGCACTCACCTCAAAAAA
CACACATAAAAAACACTTGTG
TACTTTTTTGCAACATCTACAACAATGCCTAGCATCATTTTAAGAATATGTCATTTCTACA
ACATCATATACTCAATCTT
CCTATACTTACTTCCAAAAAAACTCAAACATTATTTCTCTAAATCATATAACCAAATCCA
ACAACCAATAAACGACGCGA
AAAGTACAATCTCTCACCCACTAGCACAACCAGCTCGTTCTGGTCGCATGGACCAGAA
CGAGCTCATGCGCATTTTCCAA
>CsCaM1
CATAATAAGTTATAGTCGTTTAAGAGCCGAGTTCGTGAAGTGGGTTCAAATTTTGAAAT
GAAAAAACAAAACTTATAAGC
CCAAAATAATAGGTTGACCTTTGGTGAAAAGGTAGCCCATCATTTGGAATTTGAAGAG
GGCCTTCGTACTAAATAATCGA
ATTTGTATTGTTCTCGTCGTGAACAAAAGTCAGTCTAACGATGTGTTTGACGACTAGCT
TATAAAAATATTATCTTTTTA
AAAACTTTTTAAAAGTTATGAGTTTTCATAAGTTCAAGCTAATATAAATAAGTTTAACAA
AATGCTTTTTTTTTTTTTAT

CAGTCAAATTATTTAAATATATTTTAAAATATCATAAATGAATATACAAAAATTATAATATA
TGTGAACTTCATTTGATT
TATTTTTGTGTCATATTATAATAATAAGTTGTAACTCACGTCTAACTCGCCAAACACATTT
GTGAAAATTAAAACTACAA
CTTACTGTTTATTACGCTCTAGTTTCAACTAGTTTTTGAATACACACCTTGAATGTTGTTT
GAAATTTTATTTCAAAAGA
TATTTTGGCTAGTTGATGTGTTTAAATGTAAAAAATTAGCATTTGATAAACTTATTCGAA
AAATAACCATCGTCATTTTT
GTAAAGACATAATCGGTTTTGAAAAATTTAGGTTAGTGTTACATTCAGATAATCGCTTTT
GGAAAACACAATCCTAAACA
CCCTTTAATAACATTTTTCTAACAGACTATAAGCTCAATAGTTCATCAACAAACATGCAA
CCATGCATATACTAGTTCAT
CACGAAAAACATGCAAATACGTGCTCTCGTATATTGAACTTAGAACCACTTTATGGATA
CAACACCATCGTGTTGATAAC
TAGCATGGTACTCAAGCAAAACATTGACAAAAGATAAATCATGACGAACTTTTAACGG
TACACAACGGGTTTTCGTTGAC
AGTTATAATGGTTATGATGTTAAAAAGAGTAGAAAAGTGGCCTGACAGAATATTTGATG
GGTGTTTATAACCTGAATACT
GATAAAATTAACTTTTTCCACCGACATTTGTTAGAACTACCATCCGATCCTACCTTTGAA
TATCATCGCAACGTAACACG
ATTACTCCATGGTTGCCATGAAATTTATATTGAATAACATCACTAAATGGGTAAATAGAA
AAGTATTCCTACTCTGGTTA
CTCAGAAAATCAAGCAATTTTTGAACATGATATACACGACTTAACTAATATTTTCTTAAC
GAGGTTTGACATGATATCTT
TTAAGACTAAATGTAACGGGGACCAAACCGTTCGATTGGTCCAAATTTTGTTTGGTATC
CTTACTACACAAACCAAAATG
CACGCGTCCCCCCCCCCTACCCCATAATTTTGCATTGGTTTGGTCCGGTTGTGGACCAA
ATGAAATGGTTTGGTTTGTGT
GTATTTAAGTTTGTGAGGCATGTTTTTGGTTGTGAGTGTGGTCTTTTTACTATGAGTATT
TTGTCTTTTTAGTCTTGAAA
AAAGAAAATGAAGTAACGCTGATGTGACAGTGTTGGGTTTGGCGTTCGTGTGGTCTTA
TTACTATCAAGACTTTGAGAAC
ACTAATCTGAGGATAATGATCATCTCATGTTCATTTGAATATGAGAGGTAATGCTATGCT
TGTTGTGATAGTATATTGTC
ATTATTGTAAAATATTTATTTTATTTTTAGTTCAAATGTACAATCACATTAAGCATAATATT
GCTTCTCCTAATCTGAAC
ACCTCAATTTACAGAACCTTGTAACATCTCCAAGTGAAAAACATTACAAACCCAAAGG
TCCATATCACCCAAAACACAAA
ACCAAAAGGAGAAAAAAGTAAAAACACAGCACGATGGGAGGAGGAAGCATCTCAAA
TGACATGATATATGGAGGACCACT
CTTTTTATATATATTCCCACACCCACCCCCTCTCATCATCATTTTCTTCATTCATTTTTACT
TTTTATAAAAAAACAACA
>CsCML6
AGGAGGTAACCATTCGGTTTTTGCGCATTTGTAGGACCTATGGGATGATTTTATTATTAA

TTACTTCATCCGTCCCATTT

TATTGTCCATTTTGACTTTGATCACTCTTTCTTATTCAACTTTGACCATTAATATATTTATT

TGTGTTATATAATACTTG

ATGAAAATTATACCAATGAAAATACATTTAAAACCCAATACATTCATATATTTTTCATCAA

GTATTGTATAACACAAACA

AAAATAATTATGGTAAAAGTTGAAGAAGAAAGACTAAAAAAGTCAATAGGAGACAATA

ATTTTGGGACGGAGTTAGTATT

TGATTTGTGAGTATGTATGAGTTATTTTGTGAGGTATGTTTGTCTAAAAGTGAGTATTTT

AATGAAAGTTGTCGGAGTGG

TGACAACTAAGAGTGAAAAGTGTGGGAGGTGAATAGATAGAGAAAGCTGAGATGGCG

TTAAGGTGAGTGGTGAAGTGGTA

CAACCACTAAGAGTAGTCTTACTATATTTTTTACTATTCAAATATTATTTCTGCTTACTAA

GAGCATGCCCAACAATAAC

GGCATAACGCCGTTATATAGCTAATAACGCCGTTAACGGGCGTTATGTCGTTGGAACCA

TGGCGTTATTTAGGCGTTAGT

CCCTCAATATTATGAAAGCAACGCGCGTTAAAAAAAAAAATCATTTCACCCCCCTAAAC

CATTTCAAATAAATTTTCACT

TACACCCCCTCAACCTATTCAAATTCAAATTTAAATTATACTGGTCAATTCTAGGCGGGA

TAAATTCAAATTCAAATTTG

AAATTTAAAAATCGAATATGAGCGGAATTTATTCAAACTCAAAATCAACTATGCCTATAT

ATACAACTCTACCAGAGTTG

TTTAGAACACACAACACACACAAGTTACACACACAACTCAGACACACAAAAAATGTC

TCGCAAAGATTTTTCTCAGCAAT

TTCCAAGTCCAGAAAAGCTGCCGGAAACAAGCTTTTCAGCTTCACCACAGTTTCCTCC

AACTAGCTTTTCAGCTTCACCA

CAGTTTCCTCCAACTAGCTTTCCACCTGAACTTGAAGTTTACTCAACACCACCGGAAA

ATGTCTTCCCGGTGGGTTTTGA

AAATATCCCATACCTCCCAATTGTTTCAACTCCACCTGTGAACAGTCCACAGGTGGATC

TTCAAATTCACCCCAGCGGGT

TAAGTGAGGCTTCCCTCCGTCAACTAGAACAAGATATTGAAAACGGTAGGGAGATTAT

GGAAGAGCATGCACGTGAGGAA

GCTGCTCGTCGTGAGCAAGAAGCACGGGAAGCTCGGTATCTTGAAGACAACTACTTCG

ACATAATGGAGCGTCGGCTCTT

TAATGGGGAGTTTGATTCCCCTTCAAGTTCTGAGGGTTGGGTCTCAGATGATTCATTTT

TTTAAGTTTTAGTAGTTTTAT

TTTCATCAGTATGCGTTGTATCTTTAATTTTTAATGTAATTCGTGTTTAATGTTTAATGTAC

TTTGTGTTTTAATGTTAA

ATGTGATATGTTTTTTAATAAATGAAGTGTTTGTTTTAGTTTAGGGGTTGAAATTGAAAA

TATGAAATGTTATTGATGTT

ATTGGTATGTTATTGTTATGGGTGTTTTTTGAGGATGTTAAGGTGAAATGTTAAAGCTGA

TGTGGAGCTGACGTGGACGG

TGTTATTTTATAACTTGAGTTATTGTTGTGATTGCTCTAAAGTCTCACTTGATTGGCTAAA

TATTAATATGAAAAAATAT

TTTGAGACAACCCAAAATAACGATCTAAACAAATAAATTGGGAAGGAGAATAGATATTT

GTGGGTCAACAAAGAACAACA
ACTACATCATAACACGTTCATAATTATTCAATCATATATACGGAGTATATTCTAACAAACA
CGGGAGTCGTCCGTCGTCG
ACCCCTATAAATCCATCAACTTCATTCGCAACAAGGTCATAGACTCATGGTTATTCAATC
ATATATCTAAACTATATATA
>CsCML7
GCTCGTGAGTGTGTGACTGTGTTTGAGCACAATTTGTTAGAATAGCCCAAACGGTCAA
AATTTATAAGGTCACCAAACTG
GTCAAATAGATTTTATCAAAAGCTGAAAACACGTCAAATGGGTAAAATATGCCAAACAT
ACTCAGTGCGCACAACTTCTG
AATTCTTATAAAGAACTTAGCTTCTATAAACCATATGTAATACATCAATTAGTTACAACAT
TCTTAAACCAGAAAAAAAAA
AAAAAAAAAAAAAAAAAAAAAAAAAATTGGGTCAACCCAACCAAAACAAATGAGGT
TTTCCCTGTTGAGGTTACCCGGGG
AGGTCGAGCCTTCTTGACTCAGATGTACGCGGCCTCACCGAGTTAATCCCATGACCGTT
TCCGGCCCTTCTCTGGCCACC
CTCAAATGTACACACCTAGCATAGTTTGAACCCTTGACTTGTGAGGAGACCAAGGCCT
TACCACTAGGCCATTTGGTGGT
GGTTTGGGTCAACCCAACCTATTCTGACAAAATTCAGTGGCCACATTTATATCACACCA
ACACTTGGCACATGTTTCAGG
TTCAACAACGTCTCCAACTTCGGATCGAATCACAAGGAAAGTATCTTCAATCAATCCTG
GAAAAAGCGTGCAAAGCTCTA
AACGATCAAGCAATAGCAACTGCTGGATTAGATGCGGCTAGAGAAGAGTTATCTGATCT
TGCAATCAAGGTAGCCAATGA
GTGTCCTTCATCTGTCACCCCGACCTCTTCACTCACCCACGTTTCCGCCCACCCTGAAA
ACGAACATGCTCCAAATGTTG
ACAGTTGCTTGAGTTCTCAGGCTGATGCTGCAAAGAAGAGACAAAGGGCTATGTTTAT
GCAGTCGGAGTGGAGGGGCGTC
TAACTTTAGATAAGCTATTGGTCTATTTTGTTTTCATCCCGATGACATTTTCCTCGGCCTC
TAGCCTAATGCAGGGGGAA
GCGGGTTTTATTAAGTAGGGATTATCTTTAATTAGTCCGTATCTTGGCATGTATGGCTGAT
TTGTATAGTCGGCAGCAGT
TGGTAGGAACACAAGAAAGACTGGTTATTAGTTCTTTGGTTATGTTCATTCGAAGTTTA
GTTCTACATCTGTGATGGTTA
AACCTGGTCCGGGACTTGTATTAGTCATGGACAAGTCGCACAGTCAGAACCCTTTTCT
CTTAACCGTACGCAACCTTTCG
TCTCATGAATCATAATTTGTGAGTTTTAGTTACGTGCGTTTCACGTCGTTCAAATAGTAA
TAACATTAATTCCAAAATAA
CACCTTTTACATATACAAATATCAAGTTTCGAGCTCTTTAGGGTCAACAAGAGTTATTTA
GCTTCTCAAATAAGTCCTTA
TCAGCTGAAACTCGACTCATTGTATATATCTATTCCGTTGTCTTGGATTCCAGCTTGATCT
CAACCTCAATAAAATATGA
GAAATGATATTTCTTCGTGCTTAACATTCCCACGTATCCTCCTACTCACAAGATCTTGAC
ATGTGGAAAAACTAACGGTT

TAGATGAAGATAGTAATTGAAAAAGTGTTATATTTTTGTGTGGGTAGAAGGATGAGTAG
GAAGAAAACGTAACCGGGCAG
CTCCACTCGTTGCAAAATTACGATCCTAGTTATCTAGGCGTACGTTTTTCAACTTTTTTT
AACGTGTTTGGAGTTGCATT
TGCAAAACTGCGCGTTCTACTTTTTCAAACTGTGTTTTCTCTCTATAACAACACAAAGA
GCCATATTATAACAACTATTT
AGCAATTATTTGCGTTGTACAAAAGCAATAACGAAATAATCACTCCTTAAAACTCAATC
CCAAACACTAGAAAAAGCGAA
AAAAAGAGGGAAACTGTTTCATTTAATTTAACTGCAGTCTCCATCAGAACCTGATTCTT
TTCCACAATGCTTTCCATTTA
TCTTCTTTTTCAATCATTCTCTACCCATTATTCCTGCACCCTTATAACAACATTGCCTCGA
TCAAACAATCCAATCCTCA
>CsCaM2
AACACGCATTTCTCGTTAGGGTATTTAATAAGATGGCCAAGTATTTAGAATATTTATTTTA
TTTCCTCTTCCACTGATAA
GAATTCATTTCCAGTTCCAGTTTCTATTTAAATAGAAACACAACATTCCATTGCTCATTT
CAATTGGTGGGGAAACCAAG
AAAGGAAGAAACAGATTAAAAAAAAAAAGTCAAAAGAGTAAATTACACCGTTCGTCCT
TATGGTGTTGAAATGACGAATTT
ACCCTCATGTGCAAGTCACATGAGGGGAGTTTAACGGTCAAACTTAACGTCGTTTGGT
CAAAGGACGAACCATGCATAAC
GTTTCACACTTAAGGACGTACGGTGCAATTTTTTGGGTCAAAGGACTAAACCCTGCGG
TCAACGCAAACCACAAGGACGA
ACGGTGTAATTTACTCTTAATACTATTATACACCATCATCGTTAGACGTGAATAATCGTCT
AATAGTATAAACAATTTAT
CTATGTGTTTCGCATAAACTATCATTAAGTCATATTTTTTAATCTTCAAATAGATAACTATC
ATTATTTCACAAAAGGAA
AAGTTCTCTATCAACTTGCTACTTAAAACCTTTACGTATTAAAAGTTTTCAAGTAGTTTT
TAACTTTTTAGTTAACAACA
ATTATGACGAATTCTTTCAATGTCGGTGTCAGCTAAAAAGATGTCGAGAAATTAATACG
TCATGGTTTTAATAAATAGAA
AAACATCCCTTGTATTATGATTACTATCATTTCAAAATATTCACCGAATAAAGTTAAAAG
GGTGCCCGCGGGTTTCTAGA
AACATCATGAATTTTTTTAAGATAGTGTTAATTTTTGATAAAAAAAATCGATTTTTTTTA
AATGCATATAGTTTATCAA
AATGTGTTTCTAGCGTTAATTAGTTAGTATTTAAAAAAAAAAAAATTTATACACCAGTCAT
ATTATATTCTGTTTCCGTCC
CTTACTATGATTTTTTCCATGTTTAATAAATACCTGTCATTCAGGTTTTGAGTATATATAAA
ATTAATTATGAAAATAAT
TATTATTAAACTTTTTAGTAAAACTAGACGAGTACCCGTATATAGTAACGAGATTTTATG
GCGGCAAATAGATATATTTT
GGTTACACAATACACTTAACCAAAATATAATCGTAACTATATGCCACTTTTAAAATGTCG
TTGCTATGCACGTGTACGCG
TCTAATCTTTTGTACCACAAACGTCGAATTTGCAGAGAGTTTTCTTTTTATCTTTTTTTT

CAACAAACGTTTTCTTATAA
ATACACAAATTATATAAGGATGTCATCATATATTAGTTGACAAAATATTATTTTTTGCCAC
TTCTATGAATTTAGATAAT
GACATTTACAAAGAGGTGGACGGCAAATAGGCAACAAATTGCGCCGCTAGTTCTTTTT
GAATAACAAAACCAATCTTTTT
GAAAAGTACATCTATTGATCTAGGTGTTATAATATTATTATGCATAACCCGTTGAACTCTG
TTGTGGAATTTCACTACCT
AGGTGCAAGGAAACCAAAAGTATCAAATGTGAAAGGGATAAAAATATGTTGATTTCAA
TGCGCGTTTTGTCGTATTTTGC
CACTTTGTACAAAGCGGCTTTCAAAGCAGCCTACTGTAAAACCTCGACTGCTTAAACC
CACGAAAGAAGAAACTAAACTT
GGTAGAATACAATTGTCTTAAGCTTTACATGTATTCTCAATTTAACTTTTATATTCTTAAG
TCAACTTTTATATTTTTGT
GTGTTTCTTTTAAATCGTGTGTTTTCGTTTTAAGTTTTGTAAGATTGTTAGAATTTTCCA
AAAATACATCAAAACCTAAT
AAATAAGTTTTAAGAAAAATAAATAAACAGTCCGAATTCGCAAACACCCGATCTTCTAT
ATATGTAGTCACCATCCCAAT
CCCAAGAAACAAACAAACAAACACACACTATTGAATACAAAGAAACAAAATTTTCATC
TTGAATTAAGAAAGAAAAAAACA
>CsCML8
TCTTTTGACTTCAAATCCTAAGCCAAAGGGGAATATCAAACGGGCTCTAACTAGTTAAA
AGGATTTGGTATTAGAGCCGT
TCTCGAGGATGGGTGGAATGGTTGGCTGCCCAAGAACCCCAATTTTTAGAGCCATACA
ATTTATGTTCGATGTATAATAC
TATAATGTATAAGTGCATTTAAATTTTAAATAAATATGAATAAAATAAAGACATTTTCATA
AGGATCTTTGAAAAATTTG
TGTGAGTGATCTTCATTGTATCTTTGAAAATCAATATATTTTTCATTTAACCTTCACTTTG
AGGCTTTATTATCAATTTT
CGCCCAAGGTTTCAATAACTTAAAAATGGCTTTATTATCAATTTTCGCCCAAGGCTCCA
ATAACCTAAAAATGGCTTTAT
TATCAATTTTCGTCCAGGACTCCAATAACCCAAAAATGGCTCTGTCCAATATGGGCGTG
TTATAAGGCATATTTTGTGTC
TACAATTAAACGCATCGTACCTGTACTAGAGTCGACGTTATTCGTTGTTATATGTTAGAC
ACCATCAACAAGGTTTTTGT
ATTTTTAGTGTTTTTTTCTTATAAATTATTTCTTATCTTTTATGTTATGTATTTGTGTTTGTT
TTAATGTGTGTCGGTTG
CATGTGGCTAAGATGTGTATTTATGTTTTCTATTTTCTTTATTTCTTTATAAGATTATTAGT
CTTGATTAGTCTTTTAGC
TTGTTAGTTTTTAGCGGGTGTTTTCTTATATTATTAATCTCTGGGTGGGTATTTTTTTCTTA
AGTTGTTAGTCTTTGTTT
TTGGTTTACTATTTTAGTTAGGGGTGTGCATTGTTTGGGTAATAACTGAATAAACCAAAA
ATCGAAACCGAACCAAACCG
TACCAAGAAGGCCACTTCCAAACTTCGTTGATAAGAGCTTGACGAACCACAATTAGAG
ATTAATTTTGTTCTCAATAAAA

AAAACAGTCTTATGATAGTCAACTATGATCATGTTTGGCAAAAAGCTTTTGAAAGTGTT
TAGGAGCGACAAGCGGTAAAT
ATTTGTTTGTTTGACAACATCTCAATAAGCGTTTGAAACATGATCAACTGTTCCAATCTT
AAATGCTTTCTGAAGAAGCT
GCAAGTGATAAGCGGTAAGCATTTGTTTACACTTTATTATTTCAATTACTGCACGTTACC
AAACACGTTTTTAAACAAA
CAACTACTGTTTATTGCTAATTATTGCTGCTTACTACAAACTGCTACTGTTATTGCTACGT
GTCATCAAACGTAAATACA
CACGATAAAGACTTTTTAACTTTTTGAGTATACGTTATACACAAAAAATAATAAATTCAA
ACACATAATATTTTCAATAC
TTGCAAGTAGTTGCAACACCTTTACCATTCGAAAGAAACATCGCCCTTATTTCTTTGTA
CATTAATTACCAAAAAGTTAA
GTAGAAAAAACGAACCTTCAAAGTAAGTGGTTAACTTCCAAACAAAAAATAATGCTTA
ACCCCGTGTATCACGAAATCGA
TATACACCTTCAACGAAGATTCCAATAAAACAAAAAAATACCTTTTTTTCCAAACTCAT
AATCAACTAATCCAAAACAAT
AAACAACTTTCATGCATAACGTACAAGTCATCGCAGCATGACTACTCTAAACTTATAAC
CTTTTTTTCTTGGAGTCAAGG
TCGGCTGCTTAAATCAAGTATTTAAGTAAGAATTCTTCTTCCTACTTTCCTTAAAGAATT
TTTTTGCCGCAAACTTTTGA
CAATTGACCATATCTTTCGTACACACTTTGAACACAAATCTTATATAAACTCGATCGATC
ACAAATCTATCACAACACAT
ATTCCTTATTTTAAACTCATAAAACTTTCTTAGCAAGTAATCTAACACGTTCTTTTTTCAT
ATATATACCATAACAATAT
TCTTAACCATGAGCTTCATGATACTCGAATTGATCCAATACTTTTTCTTGCATATAATTCT
CAACAGGGTAACCCTAATC
>CsCML9
TGATGATATTTATCTCTGGTTTACTTCATAATGACAAGGAAGCTGCCATTGCTGCCATGA
ATATTCTGCTACAAGCAACC
TCCTTTATCTACATATTTGCATCATCTATAAGCATGGGTGTTTCGGCTCGTGTTGGACAC
GAGTTAGGGGCCAACATGCC
AAAGCAAGCTGAATTTGCATCATATGTGGCCTTATTATGTGCTGTTGTGACTAGCCTTAT
CGCGGTGGCCTTAGCCATTG
TTACTAGAGACCTTTGGGGGTCACTGTTCACCTCAGATTTGAAGATCGATTCACTAATA
TCGGCAGCCATGCTTGTATTG
GGGGTGTGTGAGCTCGGAAACTGTTCTCAAACTGTCATCTCTGGGGTGATAAGAGGTT
GCGCTAGACCAAGGTTCGTTGC
ATGGGTTAACGCCGGCTCATTTTATCTTATTGGGCTTCCCATGGCGTTGCTTTTGGGCTT
TCATTTTGACATGGGCTTTG
TGGGCTTCTGGAAAGGCTTATTCGTGGCTCAGATGGCCTGTTTGCTATGTATGGGTGTG
TCATTGTGGAAGATAGATTGG
GAGAAAGAGGCTAGTGAAGCACAAACTCTTGTAAACTGAATTGCTAATGAAGATGTTA
AATAATATGTTTCAAAACATTT
GCCATTTGTTTTTAGTTATGAATATAAATTTGGTGGATGACTCTTTGTTCTCGTATGTCTC

TAACATTTTGTTAAGATTT
TTGCAAGATAAACATAACACTAGTTGTCTAGTTGACATGGTGGTACCTAACAACCCAAC
TCTTTGGTATGTTCGGTTCTG
TACGGATCAAATTTATACCATGAGTTCCATGACTATTTTGTCCACAAATTATGGTCATAA
ACTTTTCTAAAAATAGAGGG
AATATATTCTTGAGTGGGGTTATTTCTCCTACTCATTCTAGAAAGAGAATCCATCATTC
CATCTCACAATAAGTAACAA
AAACATGATAGATATAAACAATATTACAGTGGGATTACATACTTTTTTGATACATCTACTC
TAACTTTTTGTACCATAGG
TTTGAATGAACGTATTTATTCGTATCTATAAGAATAAAGGGGTAGGTTATGTACCACTCC
TTTTTCACAAGTGATACCCC
CTTGATTTATTTATTTATTTATTAATTTTGTTTATATTAAAAAAATTATAAAATTGAAAATAT
GAAACATAAAATATAAA
GTTTAAAAAGTAAATAGTATTAAACTGCTAGAAAATACGAAACAGAAACTTGTACTGTA
AATAGAGTGAGTTAGAAGCTC
GAAGATGTAAAAAGTACAAAAAAAGTGGGCCAGAGGGTACCAATAACATATCCGTAAA
TAAAAGTGGGCCAAAGATGTAA
ATAGATATGATATCCCTAAGAATAAATGGTTACAAGAATAAATGGTTACATCATACACCA
AATTCTTGATTCTAGCTTTG
CTTGTTGATAAGAACATTGCCACTTACAATATTCTTCCTTATAAAAAGGTAACATCTTTT
TTGGCATTTCCAACTTAACA
GAATTCAGAATACCACACAACTATTTTAAGGTCCAATGTATATGTACAGATGGAATAATC
TTTAAGAAATGAATAATATT
TGCACTTTTAAGTTTGTTTTCTTTAACATGATAACATGGGCAAATCCTGATAGACTAAAC
TCAGAAGTCGAACATTTTCT
AACCTTTTTTATGTTTTATAACGCGTATAATGAAAGCTGTATATTCTCTACAATGGGCAA
ATATATAATGTAATACTAGT
CTGTAATGAGATTCGTTTGAGTATAAACTACAAAGCCAAAAGAATACAGCTTTGCTGGT
TTTGTGCAATATTATAAAAAG
AGAACAACCCATACTTCATTTTAGCACATCAAAACTTGTAAACTTTACTACAATGTCTA
GCAAAAGTTTCTTTAATTTCA
AATATGGTATTTCAAGAAAATCATCAACCCCTAAAACCAAACCAACTCAATCACTTCAA
CCATCAAAATTCGGTTCAAGC
>CsCML10
ACTTCTAAAACCTTCTTCTCAGAAACCTGTATACAACATATTCTGTGAAGATTTACTGGT
CGATGTGATGTCTAACGGTC
TTGATTGTTGTTCCTAATCAGATTAGTGAAGAGGAGATCGTTTTTTTGACAAATGGAGA
AGATCCATATGATGATAACGT
TGTCCGTAAATTATACCATGAAAATCTTAAGTTGCTGCTTGTTACCGAAGGACCAGATG
GTTGCAGATATTACACAAAGG
TATTGTACTAGTGGTTTTTAAGATATGTTTATGTATAAATGATGGTAATTACCCATCGTTT
CTTTAACCATGTATTTAAC
TTTATTGTTAATTTTGTTGAATTATATAGGAGTTTAGTGGAAGAGTGAAAGGTATAAAGG
TGGATCCTGTGGACACAACG

GGTGCTGGCGATGCTTTTGTAGCTGGTGTATTATCAAAGATTGCCGCGGATACCTCATTA
CTCAAGGTATGACTTTACAT
CAACAACATTTAAATTAACCAGATGTCATACCACCGCGCTATAAATCACATTTTCTTCTT
AAATTTATAAATATATTAAA
AAACCAGTTTTCTGAACTCTGCAATGCATATAAAGAGGGTTATTAATATGGATATAATAT
ATAGGATGAAAAGAGGCTAC
GAGAAGCCCTTAGCTTTGCAAATGCCTGTGGTGCGATTACTGTGACAGAAAGAGGTGC
GATTCCAGCATTACCGACTACC
GAAGCTGTAATGAATGCTCTGCTCAAGACTGTTGCATAGTTCTCCTTTTGCAGATCCAC
GCTGTATATTATTCAACTCTC
ATTTTTCCACAACGGTGAGCCATTAGGCGTGTTTGTTGTTTAGAAAAATAAGCTTACTG
TTATTGTAACGTTAATTTGCC
ATCTAGTTGAATAAGTTAAATTTGTATGATTCATACCGCCAAGGTGCTAATTAGTTTGAA
TGACACTTAATAAACTTATG
GGTTAGACTCCCTATGTGTTCATATCTAGTTATCATTTATGTGAAGATGTTTAATATTTGA
ATCTTTAGTATTTTGAGAA
TAGGAAGAACATGTCCCTTAGTTCTAAATGGCCTTTCACAAAAAAGACATAATATTGAT
GGTTGTCTGTCACTCTGTAGC
AAATCAATTATTATGTGGCTTTTTGATCTCTATGTGATTTCATTTTCTGTTGCACTTCACT
ATCATACAAGGGCAATGGG
CCATTGACAATGGCTTGTGATGTCTGAATATTAGTATGCTCCCAAAATAGTTACTAGCAT
ACAACTACTATAATTCTATC
ATAACATTTTTGACTAAAATCAAGTTGCTTTTATTTTGTTTGTTTAGTAGTTTCACATCGT
GATATTCAGAAAGAGTTAA
ATACTTGATTCTGGGTCATACTCATTCTATAGTTACTAGAGGCAAAATAGATGAAGCCTA
TAAAGTAACTAATGAAGTGC
GGAATGGTAATCGTCACTGGTATTAAATCGCAACAAGGAGTTTGATTTTACATGATAATT
TTCAGACTTGTATGATACTA
AGATTTCATAGTTTGATTGTTAGATTTTCAGGAATTTTCTTTGTCATTTGGCAGTCTCTCT
TGATCTTATGTCTTTATAG
TTTGTACTCGGCATCTGAGTAATTGTATAAATAGAATTTGGTTATAAGACAGCCCAGAAC
AAGCATTCAGTTAAACTAAA
AAATAAATTAAGGTTCTCAAAGAGCAAAAGAATGAGTCGTAAGATACCCTGTGATATAC
AAAACACAAAGTCGTTAAAGA
TGACATTTTTCAGATCCGGCTTAAAAACATGCGTTTTAAGATGTTAAGGACCATTTCTA
ACCCCCTCATTTAGGACACTC
TAGTGATGCATTCTTTGTTTACACTCCTAACTCTAAGGCCAACCACAATGTTTATCCTAC
ACAACCATTTTATAACCCCA
TATCATCATTAATCTTTTAACCAAAAACTACAAACTACACAAAAAAGCAAAAGGCTCG
GTACTACTACTAGGAACCCAGA
>CsCML11
GTATGAAAAGCGACGAAAACGTAAGAAAACGGACGAAAACGTAAGAAAACTGATGA
AAACGTATAAAAAGCGAAAAAAAC
GGACAAAAATGTATGAAAACTGACGAAAACGAATAAAAAAAAAGTGCAATTAAAAAA

GTGGTTATTAAAAATACAAATAA
TAAGAAAATAAATAAGAAAGACAAAAAAAAACATGGACTTAGGTTTAGACTTATTAAA
TCTTAGGTTAATCACCACCATT
AGATCAAACTAGATGGAATGTGGAGATTTATCCCTTAGTATGCAAGGATTTTTGAGTATG
CAAAATAAAGTATCTCTGGC
ACACCAATGACACCACATAGTCACATACCGTATGCTCTAACATTTTTTATAATTAAAGTT
TGAAAGTGCCATAAAGTGCC
AAAATAAGAAAACACATAAATATTCGTTGTAACTTACCCTAAACCTTAAGTAAGAATAA
ATAACAAAATCACATTTAGTA
ACTGAAACATTGACATTTTCAATAAAAGGCCCACGGGGCCACGACAGCAGCATAAATA
AGCTATTCTCCTAACAGCTTCC
AGTTTTCAGAGAACAAACTTACTTAATTCCACATTAAAAAAAAAAAAAAAAAAAAAAA
AAAAAAAAAAAAAAAAAAAAGGAT
AAATCACGTTTGTAGAGAAAACAAAATAAATTTCTCATGATCATTTGCAGCCTTGAAAT
GCCATAAATAAATCATGACAT
ATTTTTGTTTTGGCAAAACAAACACAGTGAACAGTTTTTTTGCATTTGCATTATTAGATT
ATGTAAGGCTAGCAAAATAA
GTATCACTTTTGGCATACAATTTTAGAGGAGAGTTATTTTACATGTCAAAATTTTCTCAC
ATACCAATTTAAAATAAAAT
CAATTGTTGAAATTAAAAGCATTTTATATTTTTTATAATTTAAATATTTCTTAAATCGTTAA
TCGTTAAGCGGAAATAAT
TACACCATCGCGACGAGTATTTAATTATTTTTCTATAAACGGGCACTTTGATATACTTTCG
AAAAAAGAAAAAGAAATAA
CTTTTCTCATGTTTTTTCGTCTTTTTTTTTTCGAATATGAGATTTGCATGAAAAATCACAT
GTGATTAAAATAGTAATTT
GAATATCACATTGGATATTAGTGTTAAATCACATATGCTATGTAATATCACATGTAATATTA
GTGTTAAATCACATATGA
TATTAGTGTTAAATCACATATGATATTCACATGTGATATTAGTGTAATATCATATGTGATTT
AACACTAATATCACATGT
GATATTCAAATTAGTGTTTAATCATATGTAATTTTTTTCAGGCAAATTTCAGTAAAAAAA
AAAAAAAAAAAAAAAAAAAA
AAAAAAAAAAAAAAAACGTGAAATGTACGAAAAAAAAAAATTGTCGAAAGTATATCAA
AGTGTTTATCTATAGAAAGATAA
TTCAATGCATGTCGTAATGGTATTTTTTTTTTTCGCTTAACGATTAACGGTTTGAAAGAT
ATTAATATTTCAAAAAAAAA
TGATGAAAGTGTGTTTTTTTTTAAATTGTTGAGATTAATCTCATATATTATATCAAATAAA
AATGAAGGGAAGATGAAAG
GTAAAGATTGATTCGTAAAGAATATTTTATCTTCAAACGATCTTAAATCTACGTTTTATAG
TATTTAAAAGTGTTTAAAA
AGACAAGTTATTTTCACTTGGACACCTAATTTCGTGTAACCTTTTAGAGAATGTTGATA
AATCATGAAAGATTGTGTAAT
CTGTTCTTTGTTTACTAATGCTGATTTAAACTTTGTTAATTTGTTGCCCATAATTATCTCC
TAACATATGATAGAATTTT
TTTCACCTTAGAAACAAAAGGTAATCCGTATTCCAAGGACATCCATATTTCTTTTGTTTT

CTACAACCATTTTATAAACA
TTCCTAAAGGTATTAGAAATCAAATAATCAAGCATAACACAGGAATCAAAATTGTTGAT
TGCTACCACTAGGCATATATA
>CsCML12
GTTTTCCACAACCACAAGTTTCCTGGGTTTTATATTTACTCAACTTTTCCACTTTGTTCG
CCTAAGTTACCAAACTTTCA
TTTTGTTCACTTAGGTTACTGAACTTTAGTTAAGCGTTCAACCTCCATTTTTCAGTGTAA
TCAAAGTAACCCAAATGCAT
TTTTTAGGTAAGTTAAGTAACCTAAATGCCTCATTTTACAAATTCAGTAACCTGAATGTC
AACTCCGTGTAGTCCAATAC
CCTGTTTGCACAAAAAAAAAGTTTGATAACTTAAGCGAATAAAGTTGGAAATATGGGT
AACCTATATGTCTATAAACCCC
AAGTTTCCACAACCACAAGTTAGGCAACCAAACCAATATTTCTGTTGGTAAAAATTTC
ATTATTTAATGCTTTATATAAT
ACTTCTACATGTTTTAAAGCAGACAAAAGTATTCATATGAAATATTCATATTAATTAAAA
CTATACAAACTACAACAAAT
TACCATTCCATTTATTAAAGTAGAAACTAATTGATGATACCTGCATCAAAACCATCAGTG
AAAATCAGTCACTGTTGTGA
GAACTTCATACACCTTTTTCCGAATTTGATTATTTTGCCCCTGTATCGTAAAATTATAAGG
AGATTTGTTCTGATAAATA
ACACAAATATGACATGATGCAGAGCATACTGATAACCAACTTCAAAAGGCAACTCCAC
AAATTGTGGGGTGTTTAGGTTT
CTTTGCGATTTATGGACTTTATTTTACTTTGTTTCTTAAGTAAATCACATGAAATCAGCC
GTTGATGATTTTTACATGCT
TGTTAGACACATATCTGCTACAGTATTAGTCAGCTTTTTAACAATTGAAGTTATGTACTA
ACTTTGTAAGAGCTCCGGGT
ACGGTGGCTGAAGGCTCAAGCCATCACCTTCGGCGCCACCCTATGGCACGAGACACC
ACACCTAGGCTGGGCTGGTGGGT
CTTGGCGCCGTGGCCACCAGCTCACGCCGAAGCAATATTTTTTTTGTCGTTGGGTTTGT
TCTTTTTTGTTTGTGTTTGTG
TTTGTGATGTGTTTGTGTTAGTGATATGTTTGAGTGACACTGTGATGTGTGTTTTTTTTT
CCCCTGTGTTTTGCAAGTTT
TGCTTGAGGAAGAAGACGATCCAATTTAATATGCGTGAAATAGTACATTTCTTGTTGGA
ATAGTATCTTTTAAGTTCTAG
TTGTATTAATTATGCCACATGCAACTTTTCTTATTGGACCTCACATCCAATCTCTTGTCTC
AAAAAAGTTTCACATCTGA
TTTGTTTTAATTGATATTTAATGTTTCAGTATTACTCATAATATAAGATTATATTGTAGTTA
ATTAAGATGATAATACAT
TTAGAAAACAGGAGGCTCTCGAATAGGTAATGGGTTAATTAGGTAATTAGTTTTTATATT
TTTATGTTTAATTTAAAAAT
GAAAAAAAAAAAAAAAAATGACGGCACTAGAAAACTGGTGTTATGCCATGGAGGGGTG
TTTTGGTGCCATAGACAAAACTG
GTGTCATGAAAAAAATGTGCTGAGGTGGCACTACTTTTTCCTACGGCACAACCCATGG
CACGACATTGTACCTAGCCCTC

TAAGAGAAAGACAATTGAACAATATAATGATGTCATAAAGAAATAATAATATGTTTATAC
ACTTTGTCATTAGAAAACAA
AAACAGGTCAATTTGTCCCACTTCCTATTACCTAAAGTCTACAGGAAACAAAGCAACT
TCCTTTGGATTTTCCACTTCAA
ACCACCATTTTTTCCAACTACATTCCAGACACTACCTTCAACATATTCTCTTTGACTTAT
GACTTTCTTACACACACAAA
CACCTTCATACACGCACAAAAAATTTAGCAACGCACACTTCTTATAAAGCCACACTTCA
TTCTTCATTCAAACTCACAAG
CTCTCTTACTTGACTTATAACATTCATATTCTTTAGTTTCATTTCACTAACAAAAAAAAAT
ACATCATTAACCAATAACC
>CsCML13
CTAAATTTGTCGACAAATCAATTAATTTAAATTCATTTTTTACAGAGAGGTGAATGATCC
AAATTTGATGACCACACTTT
TATGATGGATAACTCAACTTAAATCTATAAGAAATAAAAATAATCAAGATTACATACAGC
AAATAGGATTAAGGAAGACC
AAAAGAAAGGGAAAGTCTAATAAATAAATGAGGCACATGCTGTGTTTAGAGGAAGCTG
CATGAGAAAGTTAGAGCATTGG
GTGTGTTATGTATATCATGGCTCTTCATCACCGCCACCTCAACGTCACATCATCATAACA
AACTTTCACAACTTATAACC
ACAACAAACCCTCTCCCTCATAACAAATCTCAATATCTTCATTTGGTACCCACCACTAA
ATAAAACATTACATATTAAAA
AAAATAATAATAATAAAAAAAAAACTTGACAAAAGTACACAATATACCCTCGTCGTGGC
CACAAAAAGGGTTGTGATGAA
GAGGGACGAAGAGGGGGGCGGTGTGGTGAAGAGGCACGACAAGGTGCCATGTCACC
TCCACGGCGAGCCACAACAAGCCA
CACCAATACGCGTTGCATGCAGTGTTATGATTCATACCGGTAGATATCCATAGTCCACTT
GGACAGTTGGTAGTTAGGAT
TGTCATATGGTATAGTTTGTTTGGTTAACTGTATCAATCATAATTGAAAGTTGGAAAGAA
AAATAGAATCATAAGATGTT
AGCTAGTTTTCTATGATTGTATTGACTTTTATACCAACAAATATATGTAGAAAGTTAAGTA
AATCTGGTTAAGATTTTTG
CATGGTGTTCATGAACTTTGATGTTTTATAAACACAATAACTTTTTGTAAGAGGTTAAAG
TATTTAGGAAAACATCTATA
AATTCATAACGTACTTTATAAAAAAATTCATTATGCTTTATCTACTTCACTTATTGTCATA
ATACATAACCGTTTTCATT
TTATTCGGTTATTTTCTGTCCGGTTTTATCCGTTTTCGTAAATCGTCCATTTTTATCCGTTT
TCAATTTAATATGTCCGG
ATAAAAACAGATAAAAACTGTTTTGACATGGATAATAAAAACGGTTTTATTCGTTTTTAT
CCAGACAGATTAAAATGAAA
ACGGATAAAAATTGACGATTTACAAAAACGGATAAAAACGGACAGAAAATAACCGAAT
AAAATGAAAACGGTTATGTGTT
ATGACAATAAGTGAAATAGATAGAGCATAATGAGTTTTTTTTTTGTAAAGTACGTTATGA
ATTTATAGCTTTTTTTCAAG
TATTTAATCAAACTTGAAATAACAGTCTAAGTATACTTGGTCAACGGTCGTAGACTTGTA

GTTTTAGGTAATGTAATAAC
GAAACTCTAAGATGTTATCAACATAAATTAACTACTATTTCATTAAATTTGATTAATTATT
ATTTTCAATAGGTTCATAA
TAAGATGATGCGTACCTTATTTGTAGAAAATTAAATATTTGATCAAATGCAAATTGCAAA
TTGCAAAGCATAAAAATGGA
GTATCATTTGTACACGTTGCTTTTTTAATATATGATAGTTGTACTTTTGATGTTGATGAC
GGTGTCATATTAAACTATC
ATCTAGAGATGATGAGATATGACCAGGAATAATTACGTACAGCCAAGGGTATATCTAGC
AGATTCTTGAAATATCGAGGT
GCTAAAATGATATAATCTTGGGTAGTACTTTCTGAGACATTTCCTATTTTTGCTCCTTCAA
CATCTAAGTTTGGTCAAAA
GTCGCCCTACGGAGAAAACGCGGGAAAAAATGCATGATCTCTATATATATATCTTGCAAT
TTCAACTCCATTTTGCAATC
ACAACAAATATTTCTCCCTAAAACATATCACACACTCCATAAGTAATCTAACTATATATCT
CTATAGTTACTCAATTGTG
TTATAACGCTAATAACCCTCAACGCTGCAACTATATTTCTCTGGTTACTCACTTCTATTTG
ATTGTGTTTGTTAATTTTC
>CsCML14
TTAACTTGTGTTAGTTATAATAAAGAGTGTTTTTTTGTTGAGTTAATGTGAAATGTTAAA
GTTGATGTGACGCTGACGTG
GCAAGTGTTAAACTTTAACACAAGATTACCACACCTTGTGCTCTTAGACTATGTTTACC
AATAGGTTGACAACCCAACCG
GTTGGATATATCATACATAAATAATAATATTTTAATCAAAATTCTCAAATCCTCCCATGAC
AATGACCAAGGTCACATGA
CAACCTAGATTGTCAACCTCCAATCTGCCAAATGGCGGCTAGTGAATGGCTATTTGCTT
TATTTTTCTTTTTTCTTAAAG
AATAGATTGGTTGTATATATAGGTTGGGTTGTTGGTAAAAAGGTATAAATTGATTGTGTA
AGTGAAGAAAAGAAAAAATA
ATATTTTAGTAGAGATTGAATCGTGATACATTGTTAATAAACATAGTCTTAGAGGAATCC
GTATGACTGCATGAGAAATA
TTTATAAAATCTGAATACGCGTTGTTGCATGAAGTGTTTTGATTCATACCGGTATAACCC
ACGTGAACTGTTGGTAGTTA
GGATTGTCATATGGTTAGTTTGGTCGGTCAACTATATATATATCAATCATAATTGAAAGTT
CGGAAAGAAAAATAGAATC
ATAAGATGTTTACCTAGTTTTCTATGATTGTACTGACTTTTATATCAACAAATATTTGTAG
AAAGTTAAGCAAAGTTGGT
TAAGATTTTTGCATGGTGTTGCATGAACTTTGATGATTTACAAACACAGTAACTTTTTGT
AAGAGGTTAAAGTTTTTAAT
CAAGTTTGAAATAATAGTTTAAGTATAGTTGGTCAACGGTCGTAGACTTCTAGTTTTAG
GTAATGTAATAACGAAACTCT
AAGATGTTATCAACTTAAAATAACTACCATTTCATTGAATTTGATTAATACTATTTTCAAT
TATGGATGGCAATTCTTGA
CACGATATGATAAACAACACGAACCTGACATGAAGTTAAACAAATTTAGGTTGAGGAT
AAAAAAGTTTGGGTAAAAAACA

AGTCAACCCGTCTAACCCGTTTAATAAGCGGGTCGTGTTTGGGTTGAGTAAGTATTAAA
CAGGCCAATCCGTGAACTCGT
TTAAGTATAAAATACTATAAATAGAATATATAACTTAGATTTTATGTTAGATTTAGATTAGA
TGTATTTTATTATGTACT
TAGTATGAGTGAATTATTATTATAACTCACGTATTACTGAAATCGTCGTATAGTTTTTTTAT
GTTATTTTTATTATTTTT
TAGTTTATTATATCATTTGCATTTAAATTAGTTAAATCGACAAACCACTTATCATATTTACC
ACTTGACCTCTTACTTAA
ACAGACTAAACGTATCACAAACGGGTTACACATACGTGTTATGCACATGTTCAGTTTTT
TTGACACAATTAATAAATGAG
TTTTGTTTAGGTAGAGGTTATAATAACCCGCCAATTCATTAATTCAAATAAGTCAACTCA
AACACGACTCGATTGTCACC
ACTTTATTCAATAGGTTCATACTAAGATGATGCGTATACTAAGATGGTGCGTGTCTTATTA
GTTGTAGAAAATTAATTAT
TCGATCAACTACAAATTGCAAAGCATACAAAATTGAGTATCATTTGTACAACAAGTTGC
ATTATTAAATAATAGCCAAGG
GAGTATCTAGCAATTTTTGGAGATGTTTCCTATATTTTGTTCCTTCAACTTCCAAGTTTG
GTCAAAAGTTACCCTACGGA
GAAAACACGGGAAACATGCATGGTCTCTCTATATATATCTTGGAATTTCAGCTCCATTGG
GCAATCAGAACAAATATTTC
TCCCTAAGACATATCACACACTCCATAAGTAATATAACTACATATATCTCTATATTTACTC
AATTGTGTTATTTCTAGCA
AAACGATAATAACCACAACACTGCAACTATCTCCTGGTTACTCACGTACTTGTGCTATTT
GAACGTATTTGCTAATTTTT
>CsCML15
AGTCAAATACATAATGCCACTATGTCAGAAATTGAAGAAGGGGCGGGTGTAGGCCGTA
GGGGTGAGGGAAATGAAGATTT
TTTGTCAGTCGATCATGTTTCATTTATGTTAATTGTTTTACAAGTTAGAAATGAACATATC
TGTTGGTTAAATCGGAGTA
CAAATATTTATAAGATGCATGGGACAAAAATTCCCAAGCTTTACAAGATTTTATGAGAA
AGGTGAAAGAGGTTGCACATC
TATAATAACAAAGTTTCAAACATGATATAATATTATTAAATTGAATTATATGATTGCATTTA
TTGAGAACCGGTTATGTA
CTTTTTTTTAACATAAGGCTGGTCCCAGTCGCAACCTGGGACAGCCTGCGCCACGTCA
GCTTTCTCTCTACAGCGACCTG
GGACAACCCGGATACCCCGGTCGACAACCCGAGGAATTTGTATTAAATGTTGCTTTGG
AAAGTAGGAAAACAAGAGTTAA
CATCGGTCTTTATTGCAACCGATATTCCAAAAATTGTTCACTCGACCGATGTCACACTC
GATTGCCGCATTCGTTTGAAC
CGATGTTAACATCGGTTGTTGCATGGATTTTTTGTTGGCAACCGATCTCAACCTACTCG
ACTGCGACAAGCCTAATATAC
TTGTTTTAAGTAAGAGAGTTTTACAAATATTACTAATACCTTATTATATTATGATCATTGTC
ATTATTTGAGTTTTATAT
TATAAAATTTTAGGGTGAGCAAGTCGATCCAAGAACTGACAACCGATGAACCAAACCT

TAGAACCAACCAAAATGAGAAA
GATGGAGTCGGTTCGTTTCGGACCTTGGTCCTAAATTTATTGATATCGCTTTTGATCCA
AGCTCGGAGGAATAGGTCAA
TGTTTAAAATTTGGAACACAAAATGACATGGGATAGTGTTTTGTTGATATATAATTATGG
TTTAGTTAGGATGATCAACT
TGTGTGCCCTCCTTGTAGTATTTTATAAAAATGGATGACACAATGTGTTTTGTGTATATTT
TATAAAAATGGATGACACA
ATGTGTTTTGACACGGTACTATTGCACGATTTTTAATAAAACGACTTAACAGTTTTTTT
ATTAATATGACGGCAGTCCT
AGCTATGACCATACATTATAAGGTCGTGGGTGGTGGTCTGGTTCAAAATTACAAAACCA
TGACAGACAATTAAGTTCAGT
TCGCGGTTCAATTCTATGGAGGTTTATGTCTGGTTTTGGAGAAAACCTTGTACATGCTTA
ATTTTTCATATAAATTGTAG
CATATCCTTATAGAAAAGAATATCAACTTTTTGGGCCATGGTTAGCACGGCCAAAAAGG
CTTACTAGTGACTTAATAAAT
TCGTTGCTTCTTCCAAAAACTACGTTAAAAATGATTATTTTTACTACGTATTTTCAACA
AGTTCAAACTAAGGCGCGCG
ATATGTACCTTAGTTGTAGAAAATTAATTATTCGCTCAATTGCAAAATTGCAAAGCATAA
AAAATTGAGCATCATTTGTA
CACGTTGTCTTTTTAAATATATAATACTTGTACTTCGATGTTGATGACGGTGTCATATTAA
ACTATCATCTAGAGATGAT
GAGTTTATGACCACGAATAATTAAAGCCAAGGGTGTATCTAACACATTCTCGAAATATT
GAGGTGCTAAAACGATACTTA
CTAATTTAGGAGAGCAATTTCTGTGATAATTCCTATACGTTGCCCCTTCAACTTCTAAGT
TTGGTCAAAAGTCCCCCAAC
TTAGAAAACACGGGAAACATACATGATATCCATATATATATATATATATATTGCAATTTCAA
CTCCATTTGGCAATCACA
ACAAATATTTCTCGCTACAACATATCACACACTCCAAAAGTAATTATATATCTCTATAGTT
GATTGTGTTATTTCTTGCA
AAACGCTAATAACCAGCAACGCTTTAACTATATCTCTCTGATTACTCACTTGTGCTATTT
AATTGTATTTGTTAATTTTT
>CsCML16
ATGTCATCAGTTTTGTAATTACGTCAATATCATTCTCACGCTTTTGTAATTACATCAATAT
CATTCCTAAACGTTTGTAA
TTACATCAATATTTTTCACAAAGTTTCTCATATATATCCGAAAATCAACATGTTCTTTCAT
CACTTCATCACTTGAGGTC
AAAAAATATATATGAACAAATAAATATATATGAGCAATGTATTTTTCATATCTATATCCAA
ACTTTTGTAGTTCTATCAT
ATGTTCTGTCATCACTCGTGGCCGAAAAGAAAACATGAACAATATAATTATGATCAATAT
AAGAGATAAATAATAGTTTC
AAATTTAAAATCAATATAATTATGATTTAATTGATTTTTTGATATATATGAGAATTTAAAAG
TTTAAAAATGATATTAAT
GTAATTACATAAGTTTGAGAATGATATTTATGTAATTACAAAACAAGAAAAATATTTATG
TAATATTAAACTTTATGAAG

TATACATATGTAATTTTCTCAATATAAAGTATATTTAAATTTTAGGAGAGAAAGTATGAAA
TTACATTCTTTTTTTAAAA
AAAATAGGAATCCTATTTTTCATTTGCATTAGCATGTAAAATGATGCTAATTTTTATTGTC
CATTTGTTGTCAAACTTCA
ATTAATCTTTATCAGTTATTATATTCATTGTAACCTTTTATGAATCAATATTATTGAAAATG
CCTATCAAACTATTATTT
TTATAAATACCAAATAATTCTATACAAAATAAACATCTAAACATCAGTTTTGGAAGTATT
TATTTTTACGCAACTTATAA
ATACTTATCGTACGTTAAGCAAAGCACAAAAACAAAAATAAAGATAAAATAATAATAAG
GACTATCAAACAACCTCAAAG
TTCGTAAACTCGTAGTTTTAGACTTTTAGGAAATGTAACATGAACTTAGATAGGGAAAC
ATTTTTTCTTTCTAAAACGGC
ATAAAATTAAAAGGCATAGCAACAAGCAAAGCAAAACAATTAAAAAGAATCGGAGAT
GGCTTTACCAGCTGAATTAGTTA
GATAGCCTCTCAATCCAATTAATAGAAGGCTTACGAAATCAAATGAAAATACAATCAAA
CTAAAGGTGTTTAACAACGCG
TTAACAATGAGACTTTTCTTCGGTCTTGAGGAGGCAAAAATTAAGTCGTTCCTAAACTT
TCAAAGAATCCAAACAGTGGA
ACGGACAACCGCATCAAGTACTTTGGAGAGAATGGGGACATGTTCACCGAATATGCAA
TGGAGAAAATGTCATTATTTGT
ATATGAGTATCGTGTAGGTTCCACCATTTGAGGACATCTCGCCATGTTTCGGAAGCCAC
ATGATATGTTTTTTCGTTTCA
ATGCCTTCATCACAAACCGGGCATTTAACGAAACTCAAAAAGTTCTCAACTTAAATTAA
CAATTTTTATTGATTTTGACT
TTGATTTTCATTTATTATTATTTTCAATAAGTTCATACTAAGATGATATGACGTTAATTAGT
TGTTGACAATTAGATATT
CGATCAGTTGGCAAATTGCAAAGCGTTAATATAAAGGATCGTTTATATATACACGTTGTC
TTTTTAACTAAATTATTGGT
TGTATAGTGATGTACATGACTGTGTCATATTAAACTAAGATCTAGAGAGGATAGCCACG
AATAATTATTAAGAAGGGTGT
GCGTAACATATTCTCGAAAGAGGTACAGCAAGAATATTTTAAGGTTACATTTCTTAGAT
GCTTCCTAAATTTTTTTCTTC
TTTCAAACTTTCATTGTGTCTTACCCCCCTTGAACTTCTAAGTATGGTCAAAAGTCACC
CCGTGGGAAAAACGCGGGAAA
CATGCTTATGATCACTATATATATGATGCAATTTCATCCATTTTGCAATTCAACCTTCTTAA
TTTATTTTTCTCTACAAG
ATATCACACAGTTCGGAAGTTATATAACTATATTTCTATAGTTACTCACTTGTGCTCATTG
ATTGCATTTGTTAATTTTT
>CsCML17
ACCAAATGGGCCCCACAAGGCTTTTGGGGACGAATTTTGTAGTTGCTAAAAGACTCGA
TTCAACATTTGAAACCAACAAA
GAAGAAAAGCAAAATTGTGGCGCGTTCTAAAATGCTCCATGTTTTTTTTAAGTCACGT
GTTAGACTTGTTTAAGAATGA
ACAACACATCCTACTATGAACGTAAATTGAATATAACATATCCAAAATGAACAACATGT

GAACCGGTTTAACTTATCTAG
AAGATGAATCCAATATTTTTGAATATCGATCAATTACACCTTTCAAATTTTTATCTAGTT
CTTGTCTAATTAGATATAC
TTGCACAATTACACTACTACACATAAAACTCATTAGATACGGTTTTTAAGACTATTTTTT
ACTGTTTTAAAATCGTACTA
GATGGGAGCTTACAAAAAAATGAAATACTTTCTAGTATGGTTTTGAAATGTAATAATTAT
TTGGTACGGTTTTCTTATAC
CGTACCAAATGTCTCCAGTCAAGCCACATCACATTTTTCTTTTTTGGTACGGTTTCTAAA
TAAAACGTATCAAAATTTGT
TGGTTCAAAATAAAAATAGATTCCTTTTTCTTTTATAAAACCTGTAATCAAACACTAGCG
CCATTCATAGCCGACCCAAA
CAATATCATTTGCATTATACACAAACAATATTCAAGAAACATAACATTAATTCTATATATA
CATCAACCAACGCAACAAT
ATAGATTAGACAAGTGTTATATATTACAATTTTAAACATCATTATCATGAAATTATAAAAC
TTTAACTATGAAGGATCCG
TTGTATCTAAGAGAGACCCTACAACCCTATGAAAATATGTTTTGTAGCAGTATCAATTTG
GCGTATCAAAATATGTTTTA
TGTACTAGTGTTAGCATCATTAACCAAATATGAGAAAGATCTACAAAAGGTGTCCCATA
AGCCATTTGCTCGAGAGCATG
TCTGACCTCGTCGTTACGCATCATGTCACCCTGGCCCCGCCTGCCGTTTAAAAAAAATA
GAGACCAAAAATATTAAAAGG
ATGCAAACTGAACTACTATAAAAGTAACCAGCTAATTACATACCATTGCGTATATAATAT
AAGGGAGGTGATTTGCACAC
TTTTTATTTTGATTGGGATAGAGCACACTGTAAATACTGTATAACAATATTATACACAAA
ATAAAATGTCCCAATAAAAA
CCTCGAATGTGCAAATTAGAGGTGGCAAAACGGGCGGTCGGGCGGGCACGGGCTATG
GGCTAAACAGGCACAGGCTAAAT
GGGCACGCGCTAAACGGGCACGGGCTAAAGGGGCGCATGTTATACGAATCACTTTAGA
AAAGACCTAATATGACGAAATA
AACTCATCAAACGTAACATCTCATTTATATTTAACAAAATAAGTATATTCTATTTATAAGT
TTTTCTTTAAAACAATTGT
AGTGTTCTCCAATTGATAGAAAATATATGTCCGTTTGTGCCCATTTAACTTTTATACGGG
CAATATATGCCCATTTCAAA
AAAAAAATATGCAAAATGTACTCACATCTTTTAAAAATTTACCCAAACTAGCCAGTCTT
TTTTAATTCTGAACACAAATT
ACCACCTCTAATACAAATCAATTCCTATAATATAATGCCAGTAGCAATTAGCCAACTTGA
TGACAACATACATATGTTAA
ATTGTTAAGTTGCACTGAAAATGATTTGTTACTCTCCCACTTTCCTGGATGTATCCTAAA
ATTTGCTTCACAATGAAAGT
TCGTCATTGCTTACCCCCTTCAACTTCAAATATTGGTCAGAATACACCCCCGTGTGCAA
AACGCGGTGGCTTGTACGGCT
GTAACTAAATTCTATATGGCTATATATGTTGCAAATTCAACTCTATCAATATACCTTAGCA
CTTCTATCTTTCTTTAAAC
TTCTTATCAACAAGATACCACACTCCCGTAAGCAATATATATATACTTATATTTAATTACTC

ATACTTTTTAATACTTTG

>CsCML18

ACAATGATTTGTGAATCAAATTTGTGATTTATAGATTTCATTCACCATAAAAAGATGTTT
ACTTGTGAATCAAATCAAAA

ACATGAATCAATTTGCTCAAACGAAGATTTCTCAATCTTCTCAAAATAAAAACCTTCTC
AATGGAACCGGAGCCTATATA

TATATATATATATATATATATATAGAGTTAGGATCAAATAAGAAGAATAATAAAAATAAGAA
GGATAGGAAGTAACCACA

ACCACTCATTCTAGATGCACCTACAAAAATGGATGCATCTTCATTCAAATTTTCCCTCCC
ATATTAAAATGTGTGGCTAG

AATTACTTCTTACCCTTCTCATTTTAATTATTCTTCTTATTGGATCCCTTCCCTATATATATA
TATATATATATATATAT

ATATATATATATATATATATATATATATATATATATATATATTTATATATATATGCATGCATGCTA
AAATCGCATCTCAA

AATCCAGCATTCTTTCTCGCATTACCTACAACGCTATCTTCAGATATACGAACCCAAGCT
TTACACTTGTGAATGAATCG

GTACTCAATGAAATTAATAAATGACTTACTCCCAAATTTAGTATATCTAAAAAGATTTTAT
TTTTCTCGTTAAATCAAGT

TTCAGGTTTCAGATTTGCATCAGATTCATTTGTGTTTCAAATTTTTTTATAAAATTCGGGT
TTAGGTTCTAACCTTGACA

CTTGATATGGGTTTTCATTGAGGTTTGCATGCATGTTCTCTAATAAATTAGTAGATATACT
TGGGTTTTGATTCAATTAA

CGGTAGAAGTTTGAAAGCAACAATTACTATGTTTGGGACTTGTTTGGTAAATATGTATG
GAGTGAGTGGATAGAGGTTGA

CGATGATCAAAATTTGTATGTGTGCGTGTTTTGATAAATTTGATACTTTAGGATTAATATG
ATGAAAAAAAAAGCAAGGG

GATCGATACTTCAACTAGAGGAGTGACCTGTTGGTTCATCAAACCTACATAATTTTTTAT
TTATTTTATTTAATCGATTT

TATTTGAAGATCTTGAGGCGTTCCTCAATTTTTATATAAAAAGAATTGAAATTCGATGGA
AAAAAGAAATGAGGAAGAAG

ACGAGGGCACGTTAGTCATTTCACTTTGGCGAAGGATGAAATCGGACCTAAGATTTCC
TTAGCGGAAGCCCGCCTGCACT

TTCCGAACCACACGGATGAAATATGCAATTTGGCCAAACCACGCTGAGGTTTTTTGCA
ATTTACTCTAAACTTTATATAG

GAAAAAAATAAAAATCTAATACCTAAAAAAGACAAATACAAACTGAATGTCTTAAACT
AGAATTGGCCCTTGAATGGATT

CAAAACATTAGTATTTTTTATGGATACAAGCCCTGTCTGATTCATATCGAAAATACACTA
TGGATTAACCACCATCTAAA

GAGACAGTGATGCCAATAGCATGATATATCAAATACAATATCATTAACGTGCACAAGCTA
ATGTTACTATTACATAAAAT

TAATGCAACAAGCAATTAAATGACTAAAACAACGAACATGACACAATGACAAAGAATG
CAAAGTACAACAACAATATCAA

TCATATTCATGTTGAGGTGAGGCATGATGTAAACAATCATATACTTATCCGGGGATGATG
ATAGATAATCTGCTTCTAGA

AAAAATATGTGACAAAACAATGTATGCAACTAAAAATATTTAACTCTTGGAGGAATGAG
GATATATATTTCCTATACGTT
GCCATCACTTTGAAACTTTCCCCTTGAACTTCTAGTTTTGGTCAGAAAGCATCCCGTGT
GCAAAGCGTGGGGGCTTGTAC
GGCTTTAAAATTCCTATATTTGTTGCAATTTGAACTCTATCCTCGTGGCTATTCACTTCTT
AGCATATTCAATTTATCAA
GAGACTTTTCTTCTCTACAACATATATATTACACACCCATACATACTTATCTTTAGTTAAT
CGTTTTCTTGGATACTTTG
>CsCML19
AATGGTTAGTCCAACACTTTTTAGGACTAGACGTAAATGGCAGGGGATAGATAGGAAA
AAAATGCAGGGGAGTGGAAGCA
AGACACCAACAGTGAAAGGTGGTGTTGTTATAAAAAAGGAAGAAAGGGTTGTAGATA
AAAAAGAAGAGAAAAACAAGGGA
AAAGAAAAGCTGTTTAAGGAAGAGGATGTTTTACACAAAAAAGGGCAAAGAAGTTGT
TCAACATTTTAAGCTAGGTTTTG
ATGAGAGGGGAAGGCTAAAATGGTTGAGTTTGCAGGTTTTGATGTTAAAAACATGGCT
GGTGCTGCATGTGTTGAAACCA
GGGTTGGTGGTGGTGCTGCTGATGTGTTTAGCGGTGTTAAAACACCCTTTAGAAGCAG
AGAAAGGATTACAAACTGCGTC
CTTGCACTTTTCGCCCAAAGGCCATCCTAGGTGATGTTTCAATGATGAATACCATGTCAT
GTGTTTAATTTTTTAATTTG
TAATAGTACAATACCTAACTTAAATGTTTATTGTTTGCATACTTGTTTTTGCAGACAAGG
TAGCAGTATCATCATTACTT
TAAGGTTCATGTTTTGTGTGTTCATCAGTTTATGGATATCCACGACTAGTGTGTAGATCA
TTTGAAAGTTTAGCAGTTGA
AGGTTTTGCAGTGGAAACGGCATCATCTTTTATATTTTGTGAAATGTCAGTAGAATCTTG
TATCAGACAATGACTCACTA
GTTTCAGTTGTTTCAGAATTGAATCTATAGTTGACTGTTATAAGTTAACTTGCTAAATAG
TTAGTTATGTATAATATTTG
TTTGTTTGTCTTATAGGTTAAATTCTGTGTGATTATACTAGTTTATCAGTTGACCTTTATA
AACTTATACTTAAAATGTT
GGGTGTAATGGTATAATCAGCTCAAACAATCACCCATTTATGTGTTGGTAGTTAAAATAA
GAAATTAGATTGTGTTAGAT
TTAACTAAGGTATGGTATAAATAGGTATGGACTATAAATATAACACCCTCGACTAACTAA
TAAAAATCAGACGTTGACTT
GGGCATTACGGGTCAAGGGACGAGTTTAGCCTCAAAAACTCATACTTTGGACTACTGA
AAACTAAAATGTCATGTTTTGG
TCCAGGACGACATTTTTGACCAAGTTTAGGGACCAACAATGTATATATATATTTTTAAAC
AAGTGTTGAATATTAAAATT
TTAAGCTTCCAATTACAAACAAGTCTTAAATATTTTATAAAATAGTATAGATTTCGAGCT
ATTCAAGTTAACTCGAATAA
CCCGAATTTAAAATTTGACTCGAGTAACCTGACTCAAATAAACTTAATCGAGTTCATGT
TCAAGTCGGGGTCGGGTGGCT
TTTTTTACGCCTACTTGTCTGCTTTAGATTCCATTATACTCCCTCCGTCTCATATTTATTGT

CCATATTTCCTTTTACGG
ATGTCTCAAATTAATTGTCCACTTTCCATATTTAGTAAGAAAAAGAGAATATAATTGAGG
AATATTCCATTTGAGTGGTT
GAGATTTATAAAGGTCCCACTTTTATTAAACTTTGTGATTCTTGTCCCCTGACAATAATT
TTGAGACGGAGGGAGTATAT
ATATACTAAACATGGGGAGGAAAGTTGCTGCTACAGTAACTTCCTCCCCATTAATTGCT
AAGTTTACCAAAAAGGCAAAA
ATGGTCCCTCACCATTAATTGTTATGTTTACCAAAATGGCAAAAATAGTCCCTCAAGTTT
AGTTATCATGCAAACTAAGT
CTTTTGTAAAATTCACGTCTACAAAAGTAATTCCGCTGCATCGCGCGGGTTCCCCCACT
CGTTACCAACCTAATAGGAAT
ACTCTTAAATACCAATCACGAAAACTTTTTAAAAAGACCAAAACTAAAATATTTACGTA
GTATTCCAAATCACATCTTCC
TAAACGCGTCTCACGCGTCTTCCATCTCTATATCAACACACCCATCCACCACATTTCAA
AACAACACAAACTTTCTAAAA
>CsCML20
ATTCTTATATGGAATATATTTTTCTCTAAATAGAAAGAAATTCCAAATATTGATGGTACAA
AAATACGTTAGTTTCTCGA
AATCAAAGAAATCTAGCATTCTTTATTGGGTTGAAGTTAGAAAGGTAGCCACATACTTG
CCGTAATGAGTCCATTTTAAT
ATGTTAACTAAAAAGTGTTTTATGTTTGTTACTAGTACAAACTTTCAAATTTTGTCTAAG
AGTTATGATATTTAGACATA
CAACACAAGTATAAATATATACTTAATGAATAAATCACTTGTACAATTACATTAAGTACTT
TCTCTCTCTAAATTTTCAC
CAATCAATTTTTTAAATCAACTAATCAAATCATTAGGTTCATTTGTAGGCATGATTAGTA
GGTCTAACAATCATTTTTCT
TTGTCTAAATATGCACACCGTCATGTCACACAACTCAAACCGGACAAAAACTCTCTTTT
AAGATATTGGCATGACTTTCA
GGGTTTTTTTTACAATTAGCAGAAATTGAACTTGAGATCTAGTAGTCACAAAATTTTTA
ATATTTTGTAGCGTTATAAAC
ACATGTTTATTGTAATTTGTTATAAACACATGTGTGCGTGAAATGTGCTTTTGTTAGACA
AACTTGTATGTAATAGAGAC
AAAATAACAATATTTTGTGTTTTTATCGAATCAAAGTTTTTACATTTAGTATATCGTGTAA
AGATGAAAACGTAACTGAA
GAGAGAGGTTCAGAGCATATTGTACGGAAGTATGTGGTTGGCCGTCGGTGTCACCACA
CCCAAAAAAGCCGGTGTAGAGA
GAAGAATGGGCGAGAACTTTTCACCAGTTATGTTATGGGATAATAATTGGTGTTGCTTG
GTCGAGTAGCTGCTCTCAGTG
AAAAGAGGAAATATTTTCCGCACCACGTTTAGTTTTGACATGTACATGCTACTATGAAA
TTTTGACTTGTAATTGTCAGT
AAACAAACTTGTTTGCGGCCGGAACAAAATTAGGTTGTTAATTGTTTTCCTTATTTATAA
GGAAAGCAATTTGAGTGTTT
ATTGACTTCATGTGATTTTATTCGTTTTACCCTGCAAAACTTCTTTATTTAGGAGAAACA
AGTTTTCTTCCTAACGTCTG

ACAGGTCTCTAACAATGCTTCGTATTCAATGATGCTTTCTAGAATTCTTTCAAACCACA
ACGTGCACACGCATTATCGCC
AAGATTCATCTACCTTACAATGATAATGAAAAGACAAAAGATAACGTGCACGTCCATGA
TATAATAAGCACTCAGTTAAT
TTTTTGCGTAATTATCTGCGTAACGTTGATGTTAAAAATAACAAACTACTCTCACACTCA
TGGGCGGAGATAAAGGGGGG
GGGGGGGGGGGGTCACAAAGGGCATGTACCCCTTTAGCTAAAAATATTGTAGTGTAAA
TTTTCAAAAATATTTAGCTTTT
TTTTATAATTTGACCCTCTAAGAACTAATTTATAGTGTAGTTTTTAATTTTATGAAAGTGT
GTCCCCTTAAAGCAAAAAA
TTCTGGCTCCCCCATGGTTACACTATAACTAACCAATTACCCCCAACTAATCACTCATTC
ACTTCACATTCCTAATCACA
CATCTAAGATAGCTCACTGGCACACTTGGTCAACTTGTAATCTTTGAACCTTCCTAGAA
TGCAGGACTCATGTTACACCT
TTCACAAACAATATTTCACCTATTTAAATGGACTTAAACTTTTCATTAAATAAAGAAAA
GAAAGCATTAATTACACCGT
GTAATTGAACACATTATTTTTCATATAAATAATACATCTTTGTTAGGCTTCATATGACAAG
TAACATACTTATAAAATCT
TGTTTGGTTTTTTTTTACATGAGTTGGGCGGGGGTCTTTCATCGTGCATTCACGCACAC
CTCCTGACTCCTCCAAACCCT
TTAAATTAGGGTTCCATCTTTTCATTCCCCAAATTTCTGTCAAATCCTAAGATCTGTTTTT
TTTTTATCCAAAACCTAAG
>CsCML21
AATGCATACAACTATGTTGGAATGTGGTTTAAATAGACCTAAGAATCCTAATTGTAAACC
GACTAGAAGTAATAGTTTCC
TAACTGCCTACTAATCCTAATTAACAAATAAAAGATAAACATAAGAAAAGATTCCTAAT
AAAACTAAAAAATCTTATGTG
ACTTGGATTCCTTGCTCTCCAAGTTTCCTATTCCTTGTTTCCCAAGTTCAAATCTGTTTC
CATATTATCCACTTCATCAC
AAATTCAACTTCAAAATTAACCATGACCCAAAAATACGAAAATGATACATCTTCTTACA
TTTTGTTCATACATATCCTCC
TACTCATGTTACTGGTGCATATGAAAGAGAGAAAATGAATTTCACGTATCATCTTCATGT
ATGGGTAGGAGGATAAGTAG
GAAGAAAATGTAGGAAAATATAACAGTGTTTTTCAAAAATAACTAAATTGCAAGTATAT
AAACAATGTTTTTGAGTTTTA
TAAAAAGAAAAAATAAAATAATATGTAGCCAGAAAAGAAAAAAAGTATACTTAGATAT
CTAAAGGAAAAAGACAAGAAGT
GTAGGGTTATTGATAGGTTCTGCCTCGACAGGAAGTGTTTTGATATCGGTGATTTGGGG
CAATCTACTGTCGAAGTAGAC
ATTGAGATAACAGGTCTTAATCGTGATCTGGAAGGGATTGTTCTAAGGGAGGTCGGCG
ACAAATGGGAATGGAAAGGAAG
GAACCATAAAGCTTTTAGCGTTAAAGGGGTGAAAGAATTTATCCAAAGCCATTACGAC
TACAGTAGGAGTTTCGTGTTCA
AGTGGTCGAAATGGATTCCGAAAAAATATAATATTTTCATGTGGAGAGTAGGGATGGAT

AGAATCCCCACATGTATGGCC
CTTAGTTCCAGAAACTGTTTTTTCGGTTCAGTGACTTGCGTCCTTTGTGGCATTGCGGA
TGAATCTGTTGATCACATGTT
TTGCTCTTGTGAACTGGCCATGGAAGTTTGGTATTGAATAGGCTGTTGGATCAAATCTC
CCCCTCTTTACATGTTCTCGG
TTCTTGATATCTTTGACATGCACAGGTGGGGAAATATGGATAAGAAAAAAAGGAAGAT
TGTTAAAGGGATCATCTTCATT
ACGTGTTGGTATATTTGGAAAGCTAGGAATTCGAAGAAGTTCGACAATGGATCCATCGA
TGTCAAAAGAATTGTCCTAGA
TATTAAGGGTTTCGGTTTTTTGTGTTACAAAAACCGTGAGGGGAATCCTAAGTTAAATT
GGGGTAATTGGTGTAAGTTTG
AGTTGATGTAATTGTGTTGCTCTCTTGTTGCCCCTTCGTTAGTTGTCGTTGGTTGGGCGT
GTTGTGAATAATAGTAAACT
TTCGAAAAAAAAAAAAAGATATCTAATCTTAATAAGTGGGATTAAATGTATGCTTACTTT
TTTAACTAAATTTTGTGAAA
CTAATTTATAAGTCTGTGTATAAATGCGTCAATAAAATGTTACTCCGTATCACACAAGTT
TAGACATGGAAAATGATTAA
AAGGATTAAACATGTGATTATTTTGTGTCTAAATAGATGCGAAAGTGGTTTAATGATTT
GTGAAATTGTAACTTTCACC
AACACTTTTTATGCATGTTTAGGCATATAGACATAAAACTATGTATACTTCTAGCTCTTTC
AATCATCTATTCGTCCCAC
GATGGAAAAAACTCAAACACAAGAATATAAAATATAAAATAGACAAGACAGATGTCAT
TCCATGATTGGTTGAAACAACG
TCACCCAAATAACCACGTCTCATAATCTCACTTTTCACTTCCATTAATCTTTCACTTCTT
CTCTAGAAAACAAACAGATC
TACCAACACAAACACAACATAAAAAAACACTTCTTAACCCCTTCCTTCCTACATTAAAA
AACACAACACCCAAACACCAT
CTCTTCAAAACCTTAAATTAAATCATCAAACAAAAACAACAACATTAAACACAGTTGT
TGTTGTTGTTGTGTTTAATGCA
>CsCML22
CCCAAAATATGGAAGTACACATTTTGGCCCTTCCAAAAAATATTGAAGCTCCGCCACTG
CACGCCGCCTTTTCTAAAAAG
TTTTACCCTATTTGTGTCAGTGTGGCGCCGTGGCGGTAGGCTCAAAACCACAGGATTAG
TCGGCCAGACAATCAGAAATC
ATCGTTAAGAAAATTTGACAAGTCATTGCTTTTTAGACCGAACTTTATGATGATCAAGA
GTAAACACAGACAACAAAGAA
TGTTGGCTACGCTTGATTGTCTATAAAATCCTCCAAACTAAGCCTACTTAAGCTTCAACT
TAGATTTGGAAATTGGGAAA
GACGTTTTATTCAAGTCTTTTCCGATATCCAAAATCATAAAATAGGATTTAAATTTTGGT
ATAAATGTGATCAAGTCTTC
ATCATTTAGGTGCCACAGGGGCCACCAATAATGTGATCAAGGTCAAATAGGATTTAAAT
TTTCGGTATATACTACAGTAT
GCAACTAGGCACAAATACATTCAATGACTCCTTGCCAGAGTACAAAACATCATACTTTT
GTCTAAAATTGCATCATCATT

TAACATTTAACAGTATATCTTTATGATACCTTGACGACTGATATTGCTAAAACGTTGATAT
AATGTTATAGCTGTGCTGT
ATCCTTACACTTATCGCAGAACTTTATCTATACAAAACTTTTTCATATAAAACAGTAAGT
GTCTATGTAAATGCTAAAAC
CGAATAAAGTATGGCAGGGAGAACTAGAGAAGAGAATATGCTTATACGGAAAACACCT
CAGTATGACTTTTTGATACCTT
ATTCCGCTTACTACGTAACGAGGAAACAATTAAAGTCTAGAAAATTATATTTCTGAAAA
TTTGTGTACTTTTTACTTAGT
TATAAGATTTATTTCAGATATAAAATTATGATGAGTCGTCCTTTGTAAACTTGAATAATAA
AGAAATATAGAGTGGAAAC
CGTGTATCGAGTAGTTGACCTTGATAGCACATTATTGGCTTAATTAAATCGAGTTTATAG
TAAATGAACAGAAACATGTA
TTGTCTAGTTGGCATTAGTATAATGATGCTGAAAGTGTAAACCTACTCACATGATTTAGG
CATAACCGCGTGATTTACCT
CTTAGGTACTGTACGTCTAATATATATGAAACCGGTGTGATTTGGAGTTAACCTTATTTT
GTAACAAATTAATGTTTAAA
AAGGTCAATGTGGATTAATTTACTGTCAAGAAAAGATTTTGATCTAGTTTGGACTTTGG
ATCTAGCTAGGAGATTGATGA
ATATACAATTCGTCAAGCTCAAGAAAGTCATCGTATTATTATAAAACTTCAATAATTGAA
AAACTGATTCAAAGTATTAG
TACCTTGTATATATTATAATCTACGTGACAAGTGATCTTTTAAGGATTATAAAATGGGATT
GACAGATTCACAGAACATG
ACAGTTCCTGATCTGATTTCATCAGCCATAAGCACACTAATCATATAAAATCAACCTTGT
TTTCTCTTATTGCTTGACAC
CATTTTCAAACACAGGTCTGTTTATCCATATTCATATTCTTCTGCAATTTGTTATATTCATG
CAAGTATGTGTGTCGTGT
GTAGATAGATTTATTAAGTCAACGCTTTTTCATAGCTTTAACGATGCAATCATGATCATGT
ATTTCTGTTTATGTATATT
GTTGTCTTATATCATTTTGATTTATGAAATATTGTTAACGTGCTTTCAGTTGAAGTGCGTT
CGAGGGTTGAAATGAGCTT
TCTTTTGGATATTTGCTAAAAATTATCCATTTTGACCCAAAAGAGCTAGTTATAACTGCC
AAATGCCAAAAAGAATACAA
AATATCGAGCTTTTTTTTTTTTTTTTTTTTTTTAAATAACAAGAATGTGAAAGATTCATAC
TGAAACTTCCTTTTTGTGA
TTGATTTCAGATATTTATCTGCATTTTGTTCTTGTTTCTGCTGTGTTTATAGGTGCATACA
CATTGGTAGTTGACCAGCC
>CsCML23
GAATAAACCCACGATGGTAAAAGGATAAGTTGACCTACTAACTAACTTGTTGAGTAAG
AGGGTTGCAAACCCATCAATAC
TGGATCACGCATCCTTGTTAAACACCTTGTTCTCAAGACGTTGGAATAAGATATGTGCG
CATCCCACGACGGTTGGGCAG
TAATGCACACGTGCACGTTTAACAACTGGGGAACCAAGATACTTGCTTCCTTAATAAGA
TATTTACAGCACGCTGAATAT
CATTACATACACGGTTATGATTAAATACTTAGAATTATTTTCCGAAGTATACTATAATTTTC

ATACACGACTATGAATTT
CCTACATTGGCAGACAGATATGTATACATTACAAATGCCTACAAGCAACAAGCAAAAAA
GAAATTTTATTTATAACTAC
GAGTTATTACAAACGATATCAAACATATCCGCTACAAGCTACTACAACGCCTAAGTGAC
GAAGTGCCAACATGACTTGTC
GCTTCTTTAACTTCGCTGATGCTCTTCAACACATCTGAATCATTAGGCGACACGGTGGC
CACACTGACTGGCATTCCATC
AGACCTTGGCAAGTGAGCCGCACCAAGTCCACACAAAATGGCCTTACCTGCAATACCT
TTTTTCTTGGCAAGACGGGTGG
CATGCGCTTCCAAGACCTCCTTTAGAGGTACCTCTTTCTCCAATGCAAAGGGATTCTGA
CTATCCCTAGGACGCGCAAAG
ACGGGTACTTGGAGCTGCGACTCATGGGGTTTCAAAAAATAATCCGGCTGTGTGTCGG
CATCCTCAGCACGATGACGTGC
CAATGTCAACCCGGCCATCATAACACTTATCGGATAATCTTGGTCGCGCTCAAGCTCCG
AAATCATTGGGAGCTCTAGGT
CTTTCATTTTTTGCATGGCAGCGACCAAATGATCATAGGATTCAGCATTGTAACCCGGTA
CCTTATCAACAGGTGCTTTC
AACAGTTTTGCTTCATGGAGTTGTTCCACTGCCTCTGACTTACCACGCTCTAAAGCGCA
CTGCACAACCTCCCCAAATGC
ATTCACTACCTCTGTTGACTCTAGGGTACTAAGCGCAGCCAATCGGAGACCGTGGCCTA
TGAGCCACCTGCGTTCAGCTA
TCACTGACATAAGCTGCGGATACAATTCTTCATCATACTCGATGCTCAGCTTACGCAGCT
TTGCCTCATGTACCTCCAAG
GACCAGCTCACAGTATTCCCCAGCTCCCCCAACTGCGCAAACAAACTTTCTTTACCCT
GTTCCAAATGTTTTAGTGCCCA
GCAGCACGCAGACTTATACCTGGCAGCATCGGCACGCTCTTGATTCATTTGGCACATTA
GCGCCATTTTATCCTCGACCT
GTTTTTGCGCATATGCGCTGAGAACAGTAGCCTCTGCACGGGCGCTATCCAGTTGCGTT
TGGGTGCTTGAAAGCTGCGCC
TCCAACAGAGCTATTGCTTGGTCCTTCTTTGAGCTTTGGTCCTCTCTACCAGTTACTTG
ATCTTTCAGCTTCACAATCAA
CGAAGCCTGATGGGCGGAGACTTGAACCAATTCTTCAACTAATTGATCCAGGGGCACG
TCTGCATCAGCTTGGTTTTCAT
TCTCCTGGCTCGGCGGCTCAGAATGCGCTGACTGTGCTGGACTAGCCTTGGGAGGTAA
TTTATCGTATCATTAGAAGATA
ATTTATCATATTTTCAGTAACTATCAACCATATTATTAATACTATTATTTAAAAAAAAATAA
AAATAAAATAGAAATTAT
GTTATAAAACCCGTCAACGGTCCTCTCCACATTTTGATCGATCCATTAATTGTGGAGAA
GGTTAACTACCTTATTCCTAA
ATCCATTTGTCAGAAACACAAACATCCTATGATTTCATCTAACTAGATTTATCATTTTGTT
CTCTTTTTCGAATGTTTGT
TGGATTGGTTCATTTGTTCATGAAACATTAGAACACACAAACAAATATTAATCTCCATAC
ACGAAAAAAAAAAAAAAAAA
>CsCML24

GGTTAACTAGATTATAAATCCCAACAATATCCGACTCACCCGGAGCAACAATAACATCA
CCCAGAGCAACATTAACACAG

GTAAACTACCATGCGCTATATACTATCCGTTACTCACGTCGTGTTTGATTTTAGGCGTTC
CTTATGCTTTCCACTTGACC

TCCATAATCACCTAAACACATAATATCATTCTATCATCATTAGAATCACAACTTCATCTTT
AAGTACTATACTTAATTAC

TTGAAGCCTTATTCCAAACACTCATTTGACATTTGAACTAAACGTAATCATAATCATAAT
CTTAGAGCATGAACTTTACT

TTCCATAACATAATTATCCTTAAATATAATCATAAAACATACTAATTATGTCTAGAATCATC
AATTATATATGTAACATC

ATAACTATGATTATGTAACATAATAACTATGATTAAAATCATCATTTATACACGTATCATCC
ATAACATAATCATCCTTA

AATACATTTGCATTGTTTATGATTTTGTAAAAATTAGGAAGAAGAGCTAAAAAGTCGAA
TGAAGTATCACGTGACAATCA

CGTGGGTTCACAACTATATATATTTGTGCATGCTAAGACCAAAGAGTAGGCAAAAATTA
ACGGGCTTCATCTAAATACAC

ACAACTTTTTTTACGAGTCTCCGCATTTATAATTTCTGTCCCGGCTCCGTCGTCCACCCC
ATCTGCATCTCTACGATCGG

GCGGCTTCTTTCATCCCCCGCCGGTATTCAATCTATAGAATTAATACGTATTCAATTTTAT
ATATTAGATTGCTATATGT

TCATCGTCCTTGTAATGTTAATTAATTATGGTTTTATTTGGTCGTGCATTGAATTGGATAT
CAACATGCAATGGATTCAT

GAGCATGTACGTTGTTGTTTTGTAATTGTTACTATCACCATATATTGTTGTGATTTTGTTT
ATTTGAATGTTTGTGTTTT

GATGTTTTTTTGGATGCATGCGCGTTGTGAAAACGATGAAGGTTATTGCACGGATCTGT
GAGAATAATGAAATGGCTAAT

GATTCGTGAAATGTTGATAATTGGCAATGCTCACGGTTCTAGCTAGCTACACGAGCTAC
CACCCGCACGACAATTAATAT

GTGAATCGAAAGTGTTATGCTCAAACATATAACTATCTAAGTAGCAATAAGCTTATTTAC
TACTCTCAGATATTGATGTA

CTCTCTTATCCAAGGCTATAGCTGAGCTAACAGAGGTATATTGAAAGAATAAAAAAACA
CCATAGTATTGAATGTAAAAA

AATGACAGATCAAAGATATATCATCAACTATTAAAAGAAAAAAACGCGCAATATTCTAA
TGATCTAGCTTCGATCAGTGT

TAACACATGCGGAGTATCTAGTATATAAAATCATATTGTGAACATTTTTTTGTTTAAAGTA
ATTTTTGAGGTTCCAAATA

TATAAAGATACGTAGTATGTATATTCTGAAGCCTCCAAAACGTTGAGTCTCTATGCGGAT
TCACACCTTGAACACCTCAT

ACGGATCAATTTTTATCGTGTAGTAAAAAGTTCATATTTGTTGTTAGGGGAGAAAACAG
TTTAGATTAATCTGTTCCTAT

GCTTGAAGCTTGATCAATTTTTCTTTTGTGTCCTTTCTCGTATAAAACATTCTATTTTACT
TCAATGTAGACCTCTTTAG

TGTGCAGTTGTAAGAACGATGAATATCAATTGTTGATGCTTCAACTGACTATTTGTATTA
TCTTGTAGCTCTCATACCCG

CATAACTTTACAAGTCATTTAGAATGGATGTTAATTTAATGTAGGATTTCAGTTTGTCATT
TTTTTAATCACTTTAATGA
ACGTAACGTCTTCTGTTTGGATAGTGATCGTCTGTCTGATTAGCTTTGTTTTCTTTGTTT
TTTATTGTGATTCTGTGAAG
GTTGTGTGAGATGATTTTGGTATTAAAACAGCAGTGAATGCTTGCGATGCTTGTAATCC
TGCACCGCCCTTCCAGTCTCA
>CsCML25
TAAAGAGAAACAGACAATAAACCAGAAAAACAGTAGAAAAAAAAACTAACCAAACA
GATAAGCAAACAGCAAACAAATTA
CTCAATCAACCACAAACAACAATCCTCAACTTCCAAGACTGCCCAAAAACCTATCCTC
CAGCAATCTAGACTCAATAAAT
AAAAAACTAAAACAATAGCTAAACTCTCACACCCAAAACTAACTTGGATCAAGCCAAG
TGGACTAGGGTTGGCCAAAGCA
GGTATTCGTACCACTCACGATGGTAGATCAAGAAGTTAACCAGCACGTTTGCACCCACT
TTGACTCAAAACTAGTTATCT
TAAAGGAGAAATTTGACTTGCAGTGCTTTAAACGAGTTGCAGCGGAAGAAACAATTAG
TTCTTCTAGATTCACTTAGTCT
AGAATAACCAATCAAAGAAACAGCCCAGAAATCTACAAAACCACCAGATCTTCGAAA
AAACCCTAAATCCAAAATTAGGG
TTTCAAAGAAAACCAACCTAGAACCAACAATAAACCACCAAATTCTTCGATCAATCGT
CACCAAAACCACTGGATCGTAC
CAAAAACAAGAATCCCCAAACTCTAGGGTTTCAAGAAAAAACCTGGATCTTCAATTGA
ACTCCTAAATCTTCGATTAACC
CAACAATCAACAAGATTCAACAATTTAATCGTTGAATCAACAACTCTAAACCAAGATC
GATCAATAAACCAAGAAACCAC
CAAAAACCAACTAATCAGAACCTAGGGCTCTAATACCATGTGAAAATGGTACCAAAAC
AACACAAGTATCTAATCACCCA
ACCAACCACCCTTTGGTGATCAACTTTTATTATAACAATATACACAGTAGACTAGTCCCT
AGCACATGTGCTAGCCAAAC
AAGATACAAAGAAAACTGATAGTCCCCAAACTGGATGTTATCACTACAAATCAACTCA
CATTTACAAAACCCAGAAACCA
GTAGATCAAGATGACCTAAACTAACCAAAAAATATACTAAAAACTGAGAGTACAAAGA
CTCAAGAATATGAGAGCACTAG
AGTGAATTTGATGTGATGAAAACTTGTATCATGAATAAGTGAGAAACCCTAGCTGTGAT
GATAGCTTTTATAGCACCAAA
TTTCACTTCAAACCCTGCACATGATAAACTTACAAAAACAACATCAAAACTTATTAAGT
GTAAAGTTAGTTATAAAATAA
TTTCATAACAACCCCTGAAGTATCATAAGCTTGCTGAAGTGCTTTATACATTTAACAAGG
TTCAACTTGTCGATGCATCA
GTGATCATCAAATTACGAGAACCAAATATCATTTACTATAGCCTAGTGTCGTGTAACATT
CGTTTTTCTTTTAAACTGTG
TCTTAATTTCATTTAGAAATCACATCATGTTTTACATCAAAAGGTTTTCTCCCTTCTTTTT
GTTACTCACCCGCTTCTCT
TATACGAAGCATGAGGCTCATAAAATTAAATATGGGATGTCAATATCCAGTCTATTTTAA

TCTCTACTCTTACTTAGAAA
AATATATGACTTTCCAACATTTTAAGATAGATATTCTAGTACCTTCTAAGACGTGAAGAG
CCATTACAAATTTACAATAA
GATTTTTCACGACTTAAATATTCTAAAATGACGTACACCAGCCGTACCGAATAACAAGA
AAACGTTGAAGACTTTTCGTC
TAGAACTCTAACTGCATTTCCTAGCAGTTTCTAAATTTCAATGGTAAACTGTACGGCAG
CTCCTACATTATATAAGTTCT
TGCTTTGTCAACACGCGCAAAAAACACGGACCAACCAATTTATATAACTAACTCTTCGC
TCTATAGTCTAGAAATAAACA
AAGTACTGATATATATATACTCCAATTTCTCATAAAGGAGGCTCATTACCGGTTTAACTTT
CTACTTTTCCATTCCTTTA
CATACATAAATAGCTATACAAACACATTAACTAACACTTTACTCACACATATTCGGTGTA
TTGCGAGTAAACAACCTTTT
>CsCML26
TTACGCTAACAAAAAACAACAAACAAATAATTATAAGTCATGCCCGCCCATTGGGCGG
GCTTCGAATACTAGTATGTATG
TATATATATATATATATATATATATATATATATATATATATATATATATATATATATAT
ATCCAAACTTT
TGTAATTACATCATCATGTTCTTTCATCAATCGAGGCCAAAAATATATATAAACAATACA
ATTATGATGAATATCAGAGA
TAAATAATGGTTTTTGAATTTAAAATCAGTATAATTATAATTTAATTGATTTTATATGAGAA
TTTAAAAGTTCGGGAATT
TCTATCGTTAAGGTCTGTTGGAACTCTCGAGAAGGTTCCAACTGCACTGTCAACGAGA
AACTGAAATCAAGCGCAAGCAT
CTTCGTTTCCTAGAACCCTATTAGGTAATTTTAAGTGATATTGGTGTAATTACAAAAGTT
TAAGAAGGTCATTGATGTAA
TTATAAAAATGGGAAGAATATTTATGTAAATGAAAATGATATTTATGTAATATTGAACTTT
ATCAAAGATATACATGTAA
TTCAACATTTGTTTATTTGATTTTTAAAGAATTTGTGATCTCTATTAATCTCATCTATCTTA
TAGTACTAGCTATTGTTC
CCTTGCAATTTTTTTTCAAAGTATATTTTTATTTCATGTAAAAATCAATTTGAATATAAGA
GGTAATGTTGAGGTTTTTG
TGAGTGGAAAAAATGAGTCATCTTATTTAAGACCTCTAATTTCTTACAAAATAAGTTTTC
CTTCACAACAAACTCAACAT
GACCTCTCATGTTCAAATAAAACATGAGAGGATCTTATTAATTTGTTTATAATCACCCGT
TTTATCTAAAAGGAGAAAAT
GCATTTGTTTGAAACTTAAAAACTTAAAAATTGAGTAATATAGTTAAACCAAAAACTTA
ACTAAAATTTAAAATACTAGT
GTAATTTTCGTCACTCTCCTTCATACTAAATGTAAATGGCTAATTACTTAAAAAGTCATC
AAACTTGACACTTTTTGTCT
TTGTGTGTACTGAACTTTTTTTTTTTCTCATTGAGTGTATTGAACTTCAAATTCGATCATT
GTGTGTATTTTGTGACCAT
TTTACCCTTACTTTTTTTAAATTTCAAAGGGTAAAATGGTCATTTTACATGGCTGAATAG
TCACAAAATAAACACAATTA

TCGAAATTTGAAAGTTCAGTATACATAATGAAAAAAAAAAAAAGGTAAGTGACACAAT
AACAAAAAGTGTCAAGTTCGAT
GATTTTTTAAGTAATTAGCCCAAATGTAAATGGTTATAAAACTTGAACATGAATAAGGTT
TTTAATTATTTATATACAAT
CCGTTTTCACCTATTATCAGTTTAAATTTTGTGACTTTGTGATTGGGGTGAGTTTTCCAC
ACCCTCTCATAATGAGGGTT
TGCAATGATTGGACCCATCATCAGATTGTACGGTATGCATCACGGGTTGTACTGTTTGAT
TAAACCTTTATGATGTGACA
ATATAAAGAGGGTATGTATAGGGTATTCGACTATCTAAAATTTTAGGATACACATGTGGT
ACCTTCCAGGACGTCAAAAG
CCAATAAGATTTGTTCACGACTTAAATATTCTTAAATGACGTGCACCGGCCTTACTGAAT
AAGAAAAAAACGTTGAATAC
TTGTTGGTCTAGAACTCCAATTGTACAAATTAAGAGGGTGCATTTCGTAGTAGTTTCTA
AATTTCAATGGTAAACTGTAC
GGCACCTCGTATTACAATTATCGTTTTGTCAACACGCGCAAAACACATGGACTTTTAGT
TGATTATCATCGTTTAAGTCC
AAAAATATGAGAAGTATATAAATAGCTCAAATGTGTCACAAAGGAGGCTCATTACCGGT
TTAACTTCCAATATCTTACAT
ATATAAATAGCTATACAAACACATTTACCAACACTAACTTACTCACACATATTCGGTGTA
TTGCGAGTAAACAACCTTTA
>CsCML27
TTATGTGTTTATTGGAAGTAAATGCGAGTTGCAAACTATTTCTTGGACCTGCAACTCTTC
TTTTAATTTGCTTATGTGTT
GACTAGCGAAGGGTAGATATGTGGTAGATATTGACTTAGGCTAGGGTAGCAATCCTTTG
CTATAATTACGATACCTAGCT
AAGAATAGATATCTACTTCCGACCTAGACCATGGTAGCATCGAATTATGCTATGCTATGG
CCGTGTTACCTGGCTAGAGT
CGCTAGGGCCCTACTGAGATCTCGATGCGTTATTGGAAGTAATATTGATGGAATGTTATT
GGAATATTGTTGGAATGTTG
TTTGTTGGAATGTATAGCATATATATATGCCTTAGTTCCACATAGCAAATAAACTCTATCA
GGTCCATTACTAATTTTGT
GATTAAACTATATGAACTCACCCGCGTAAGCTGACCTTTTTAGCATGTGTTCTCAGGTTT
AAGGCGACAACTCACTATTT
GAAGACGCTTGGCAATGGGCATGGTCATAGGCGACTCTGATAAACAATTGGGACCGCT
TTGAGTTTCTTTATGGGCCAAT
TTCCGCACTATTTTCATATAAAGAAAACTTAATGTTTTTGATAATGAATTTTATTCTAAAT
GAATGAAATAATAACCATT
CTTTGTACCTTATGTTTTGATACCGATATGGTAGCACCCCAATGCATGTATGCTTTTGCTG
GACCCTATTTTCGGGGTGT
TACAGTTTTACTCTATGAAACGTTAGAGTACAACCTTTTTTTGATGACCATGTCAAAGA
ACAAAACTCTACAATGTGGAG
ATATGGTAAGTTTTTTGCTTATAATCGTTATCAATGATATGTTCGTACGATTGCTTAAGGT
ATTTTTCTTTTAGGTAAGA
TAATGTTAACAGTGTTTTGTTATGTGTTTCAACAGCTGGTCGATCGGGTCAGTTTAGCAT

GAGGTTTCATCACAATGGTA
TTTTCAGAAACTGATCAGGTAGAATATACGAAGGAGGTAAGGAATGTATTGTTGTTTAT
GTTGATATGAATGAGGTCTCT
GTTGATGAAATAGATGCTCTTATGATCCAAATTGGTTATGCTAAAGGACAAACCATTTAC
TACTGCTTTGTTAAACCTGG
TAGTACACTGGATGATGGGTTGTATAGCTTCCATAAGCCTCTAAATCTTAAGTTTTTGAA
AATATATTGTCAAAGAGCAT
AAGTTGATAGATGTTTATGTTAAAATATGTAAGACAAGATTAGAGTTGAATGAAATGTC
CCCGTTAAGACTGTTTTTAGA
GAAATTGATGAAGAAAGTAGAAAAGTAGCTAGTGATGCAATCCAATTGTGTTATAGAC
GATTGGTTTTAGATGACCTTGA
TGAGCTGAAAGTAGGAGCATTTGAGTGAACCGACCCTAGTTTCCTTTATCATTTTAGAC
TCATTTTTGAGAATATTTGTT
TTAATATTTCATTTCTTTTAGACATGTTGGATTATGTTTTTTCTTTTGAAGTCATTCATTTA
TATGACTTGGTATTTTAT
TTAATATGACTTCCGTCTGCGTTTATATGCTTTGTATGATACCATAGTATGACATTTTGAAT
CGTGTCAGGTTGGCGTGT
TACACCTTTAGTGTTACTGTGTTAGGAATTAAAGTTACCTATGCAATATGCATCCTTCAC
GAATCCGAATTTACTTTTTC
ATAAATAAACTATATATATCTACCTCATGCAAAAACAAATGGAAAAAACGGTTCTAAAG
GTCGCGACAATATACACAATA
TAACTTCAAAAGTCACGAACGAAGTTGCCATGCAAATTCCTTGTGAACCAAACTTCAC
GCAATTTGCTCTCGTTACACCT
TCTAGGAAATTTCCATTAGCATTTTTCTTCCAACATTATATAACTGTTTTCAAAATCATGT
CTTGTCCCAAGTGTCTTTG
TTTATGTATATATAGGTATCAACGTTAGGTCATTCCATTCTTAAAATTTAGTAGAAGCTTT
ATTATATCTTAGCACTATA
>CsCML28
CAAGTTAGAGACTTATGAGCCATTGGATTTAATCCAATGGCTCAAATCAAAAGAACTTC
TTTTGATTTGAACCATTAAAT
TAAATCCAATGGCTCACAATCATCTAACTTGACTCAAGTTGTGTTATGCAACTTGAGAG
GATCTTGATCCGTCTACTAAA
CAACAACATTACGTAAGCTTTGCATCTCACAAACGACTTCTAAGTGCACGTGTAACGAT
ATAGTTAAAGGGCCACTTTCG
TGTTTCACTTTGCATAGATAAGTAATTTGACTTTTATTAATTTAATAGTTAATACAACTAG
AAGTAACAATGTCAACCCA
CTTAAAAGTCATATAAAAATGCACACAATGACACAAAGGTAGGAATATATCTGGTAGCG
CTAATTTGTGACATAATATTG
TTAATTTAATTCATCAAAATACCTTGTCTAAGTGGACTTGCTTCAGGCGTAGTAAAAAC
ATGTATGCTCGCACTATATCC
GATGGGCATTAGCTGTCGATATCTTTTTCTTTCTGTAAATAGACTTGTTTGCAATGTACTA
AGTCGTACTTCATTATTTT
GGGAATCCATATTTATCAATTCATGTGCGTATGTTCAATAGACAAGTTATACGTCCTTGA
CTCTTCAAAATTGTATTTAC

TTTTCAACATTGTGAAACACTAACTTTTACTTGTTGGCCTGTAAATGCAAAAAATTAGA
AAAAGCTAACATTCTTTTTTC
AAAACTTATTCATATGAATAGCTAGTTTTTTTACATGTAAATATGAATAACTTGCATTTAT
AATATATTAAACCGTTGGA
GTATTTTTGCATTTTTCATGATATTACACTAGTGTTCGTCCCCAAATAACTATCCATCCTA
TTTTCATGATATTACATTA
GAGACGTTATTTATTCTCTTTGTCCCCAAATAAATCTTTCTTTTTAATTTTGCATTTTTCAT
GATATTAGGTGGTGTCTG
AATGCAACTTAATTGACTTAATATGAAAAAACTTAATTTATTAAATCATATAAAAAGTTG
TTTGTTTTTAACTTAATCAA
AATAAATGACTTAAAATAATAAGTGATAAAAGTGAGGTCATCCACATTAAATCATTTGTT
AATTAATAGGACTTAATAAA
AAAATTAAACAAACAAGCCCTTACATTAGCGACGTTATTTATTCTCTTCGTCCCCAAATA
ACTACCCATCTTTCTTTTTA
ATGTTGTCATAAATTAATTGTTCACTACTAAAGTTAAATAATAAAATTTAATTGAATTTAG
GATATTATTCTTACTTAAA
ACTTAAAACTATTATTATGAACATTAAAGGAAAACAATAATTACTTAGTTAAACATAAAA
CTCATATTCTGTAAAATGTG
ACAAAAATATAAAATAGTAATCGTCGAGTGTTTTTTGTCAAAAGACAACAATTTTTGAA
TGGAAAAATTAATATCCGTAT
TGTTATTTAAATAATTAAGTTGTTCATAAGTCTTAATTTATGAAAGTAATGGCTTGTTTGT
TACAATTTTTGACTGAGTT
ATGTATATATAATTCTATATAAGAGATGACTTAATATAAACATCTTTTATTTTATCATTTTAT
ATATTTAGTCAATATTT
TTTATTAAGTCACAAACAAACGTACAACTTTAACATGATTTTGAGAGTCCAATTTTGTT
ATATTAAATTTAACTCTAAAT
TCTTTTACAAAAAGTTAAATTTAAGAAAGTAAATTATACATATCTTCACCCAAAAATAAA
AATCCCTAAACTACATACCC
AAACACCCTCTAAATTACAGTTATTTACAACATACCACAACGTTCCACAATAGGCGCGA
GCACAATATCTCACCTTCCAA
AAATTAGTTACAACTTACAAAACCGCGTGCAGTCATTCTCACAATCAGTCACCGAACT
GTCACTCTACCGTGTCTCAATT
CACGCGTCTTTCTCCCATATAAATACCAACCCCATTCACACATAATAACAATAACCAAAC
CCTAACCCTAATTCTTCACA
>CsCML29
GTATTGGACACACACCTTATACCCTTGAGGTAGTTTATATAATGCATTTTTTTAAAAAAA
AATCGGTTCCAATCGATTAG
ACCCGCAAAGAACCGAACCGGACTGAGAACCGAACCGGAACAAAGACATGTTACTCG
GTCCGGTTCTCGGTTATTTGGCC
ATCCGTTTTTCGGTTCTCGGTTCTTGCGGTTCCGGGTCGGTTCCGTGCGGTTCCGGAAC
CGCGCTCACCCTATTCAAGGG
TAAACACATGAAATTATTAAGCGACACCTTTTTACTTGTAGTGACACTTTCTGACGTGT
CACTAAGTGGCATTCTTGTAG
TACACAGCTTTCACGTTTCAAGCTATTTGTATTGCATCAATGTGAGGTGGAAAAGAAAA

TGAATTAGATAAGAAACAAGA
TATAAACTCCTTATAGAATGAAGATAATTAATGATGTAAACTACGAAACGCCAGCTAGCT
TTTAGGATCACATATGAGCT
CGTCCAAAAGCTTGGATGAAACTATTGGGCCGGTTATCGCATACTTTAGTGATGTCTCT
ATGTCTCAAATCAGTATCAAG
TTATCATATGAGCTTGCTCAAGTGGCTAGTCAGGATTTCTTGACCCCAAATCTTATGCTT
TGGATGGATGACAGAAGTTT
ATCCATCGGATGGTTTTCAACTCTCGAAAGAAATTCAAGTCAATAGACAACCACCATGT
TACAAGAGGACCATATGAATT
CCTTCTCATATGCCCATCACATATGTTCAAGGCATCTTCCTAAAAGTAAGAAATAAAGC
AATCGATTACTTGGATGGATG
ACACAAGTTTACCTATCGAATGGTTTTCAACTCTTGAAAGAAATTCAAGTCTTTACAAG
AGGATCAACTGAATTCGTTCT
CATATGCCCATTACATATGTTCAAGGCATCTTCCTAAAAGTAGAAATACAGTATGGTGAA
TATACCCGTTCTTTGTTCAA
AAAAAAAAAAGTATCTATATATATACCAATTCTTTTTCTTTTTAAAAAATACTTGTTCTA
TATGCGTGCTAATCTATCT
AAATCGTGTAATCCGTGTTATATGTAGTTGCATTAAAAAACTATATCTTGTAGAGCAAGA
ATCTAGCTTGTGATGATCAA
GTTCTTGAGTTTCCGCCACGATGATCAAGTTCTTGAGTTTCCGCCACGGAAGTGTTTTC
TAGATATATATGATTTCAAAT
ATAGCGATAGTATAGTGGTAAAGGAACATGTTCTTTAATTTCATTTTCAAGTTTGGCTTA
GATTCAAGCATAAATTGATT
TGTACTTTTTATTTGATTCTTAACTGTCAAGTTTGGGTTCGATGCCAACATAAATTAATTC
GTTCCATGTAAAACCGACT
AAAGGTGAAGTTGGGTCAATCAACTCTCGATCTTGTGGAGTCATGGAGGTTACAAATT
TTGGGGATAATAACAAAAAATT
GTATATTAACTTGTCCATTTTTGTTGTTCTGTTTATCATTTTTTTTTAATTAAAACACAAAT
ATCTCAAATTTCAACTTT
TGAGAGTATTTTATAACCATTCTATCCTTACGTTTTTTAACCACCCTGTTAACATCAAAG
GGTAATTGTACACGTACATA
ATATACACAAAGGTCGAAATTTGAAAGGTTTGTGTTTCGATCGAAAAAAGAAAGGCTA
CACAAAATAACAAAAAATGGAC
AAGTTAGATAATTTTTATTTTCTTATTATCCCAAAGTTTTAATATTTTACAGAGAACCCAA
TATATCCTTTTGGTACTGC
AAAGTGGTGAAGATAGACCGGAATTTGAGGAGAATCTCGATTTCACTGATATGTACGA
ACCAGTTAGTATGAAATAAAGA
TAAAAATTTCACAAAAAAATCTTAAATAGCTGCAGTTTGACGAGCTTTCTTCCTAGCAA
TATGCATGTGAATTGATGTGA
GTGCTTACAATCAAATGGTTGTGGCTTGAAGTCTCAACTTGGATAAGTGTGTGGGAGT
GAGTTTGTCTTTAAAATATTGA
>CsCML30
ACCGTCTCTTACCGATAAACTCATCTACCACTTTTTCCACACACACACAACTTTCCAAT
GGCAGATTTCCCCATTTTAAA

CAAACTCATGGAGGTGGCTGATTCGCCAAATCTTAATGAACGGATGGCTATCTGGTTTG
AAGATGAGGCTGCCCAAGAAG
GGATTCTAAGGAAGCTACTTCTTCAGTGCTGCCAGCATCTGAAGACAGGAATCATCAA
CCGTAGGGAGCTGATACAAGAG
AATCAAGCTCTGGGACCTCGTGGAATTGCGCTGAACAAAATCCAGTCTAGGCACACAA
ACCAACTTGCCCAACTCATACA
GATTGTTGGTCAAACAGATGAAGGTATTCGGTTCAACCAAGAGTTTGTTAAGAAAATG
GCTGAACATAATGCCTGAATTT
TTGGTTTTCCATGGGGTAGATAAAGGCGTCTTTTGGGTGATAGTTTTGCTAAAAATGGT
ATGTAAATTAGATTTGATGTA
GTTGGTTATGATGTTTAACAGACTTTATTAATGATAAAATATCATTTGGGTTATGTGTTGT
TTAATCTGTTCTTGTATTG
TGATATTGGGTGTATTTTAGTGCTTTTAGTATCAACAAAACCAAACATCCCCTTATATCA
GCAAAACCAAACAGGCCCTT
GTATCATCAAAACCAAACAGGACCTTGTATCAGCAAAAGTTTAATCAGTAGAACCAAA
CACCCCCTTGTATCCGCAAAAG
CAAAGGCAATGGTTTAAAAAGATGTGGTGAAAATGCAAATACTTTGGCACTAAATCAT
AATGTATCAAATAAAACATCCG
CTTGTATCAGCTAAACCAAACGACCCCTTTTATCAGCAACAATTGTTACAGCAAAACCA
AACACCCCCCTTTCAGAAAAA
ATACAGGCAGTGGTTTCAAAATACGGGTTCAAAATGCAAACGCTTTAGCATTAAACAA
CAATGTATTGACAATGGTGTAT
TGTAACATCTTGAATTTTGACTTACCAAAGTCAAACAAACTTATGAAAACCAACAAATT
GTAACTTACTTGGGTAAGTGA
AACCTATTAAGAAATGCAAAACGATTTCAAACACAATTACTTAAATAAGATCACAAAAC
TTTATTCATCGATTACATGTT
CTCAAGAACTACAAATGTTTACAATCTACTAAACATACACTTAGCTAGACCCACAATTC
TCCCTATCATCGTTTTCCTTC
TTTTCCTTCCTTTCCTTAAGATATTTAAACAAAATTTCCAGTTCTGTAATCCTTTCTCCCA
AATCGTTAAACTTTGATAA
CAAGATGTCATCCTCTTCCTTCTCCTTCTTAACACCCAACCGCTTTTCATTTGCGCAGTC
TGCTTTCCTCAACGAACTCA
ACAAACCCTTCCCCTTATCGTTTTGTGCCATTATTGTTTTTCTCTTTGCTTTGGGAAGAA
TGTGTGTTGATTGTATTTTA
GGGTTCAGTCTTGCTCGTTCATACATATGGTTGAAGGTCGGGTTACATTTTAACTAGGTT
GGGTAAAAAAAAGTGAATGG
TCTATAATACCCTCGAAGGAAATCGGTTTGACTGTCCCAATTCTGGTCAAGGGACGAAT
CTGGCATTTAATGGCCACTGT
TTGGACCCGTGGCGATGAAAAGGGCGTGGTTCGTCCTATCGCGATCCTCCAAGACAAG
TTTAGGGACCAACGGTGTATTT
TAGTCAAAAAATTATTAATACCCAAACCCACATCCAAACCTACCAACCTGCCCCAAAAC
CCGTCCCAACTGGTTCGAGTT
TCGGTTTCCCCATCGGTTTCGGATGCTTTTGCCATCCTTAGATATGATTCTGGTTATTGCC
GGATCTTAACATCCAAATG

ATAAGGAAAATACAAAGAATATTAAGCACAAAAGAAAGTGCAACCCCACGCCAACTC
CCTGTGCCCCGTGTATATATATG
CAAAGCCTCATAGTGATGCAAAGCACCTTCTCACACTATTAACACAAGGCCTTCTATTT
CCTAATATCTTTTTGTTGAAT
>CsCML31
GGGTCTAATCTGCGATCGGCAGTAGTCATGTTAGACGGGACATTATCAAGGCCAAAAG
CGCCTTTGGAAAGAAGATGATC
TATTATCTGTCATCACTTTAATTCTTATAGTTTTGATGTTTTTCGTTCAGTTGGTATATTGT
AGAGATTTATGTATTTCA
TAACGATTTCTTTTAGCTCAGCTGAATTAATATATGCTTGTAGTATTAATGATAATCGAAT
GCTTATTTAAGTCATTAAA
CAGCTAAAAAATATTAAAAATAAATAAATAAACAAATCAAAAAAGAGGGGAAAGGGG
ACAAATTTACAAAACTTACCCTA
AAAAATAAAGAAAGTAACAAATAGGCCCCTTGAGTTGAGGGGAAAAAAGGAAGGGG
AAGAGAGGGATGATCTAACGGTTG
AGATCACTTCTCTCACTTTTCTCCTGAGATGAACTTTTTAAATGATCCCATATATATATAT
ATATATATATATATATATA
TATATATATATATATATATATATATATATATATATATATATATATATATATATATATAAAATTA
ATGCAAATCCCC
CCCCCCCAAATTAGGGTTTTAATTTTCAAACCCTAGAATTCGACATATATATAAACATAA
TGAAAATATTCGTTTTTTAC
GTGTTTCAGTAATTAATTACACATTATAAAGGCTCGTGATGCTACTATTGGAATTAAATA
ACATAACAATCATAAATTTA
AATACAATAAACATCAAAGTACATGCAGAAGCATAGGAACATATAATTTCAATTTTAAA
ATGTATGCGTACCTTTTTTAG
CAATTCAAGAATCAGATCAAAGATGAACAAGAACCTTAATTAGGGTTTATGTGGATTAC
TGAATTGACCATTACCTTTTT
TCAATTCAATCCAACGTACAAACAATAGTATGTTTGTACCACGTTTTTGTACACACGTAC
TAATAACGACTTAAATAGAA
TGCTAGTTCTTAATACACGAAAAACCAAAACAAGTTTTGTTTTTGAAAATCAAAGTCG
AAAAAACCTTAATGGGTGTTGT
CAACCGGTTATGTAAAAGAAGAAAAAGATGAACAAGTTTAGTGTATTAAGAGTGCACA
AGAGATGACAAATAATGACCCT
AAGCCTTGATTATATACTAATGAAATCTTAGAGGACAAGTTAGTTTAAGAATGGGCTTA
ACCCATGCATTACCGATCGGT
GCTCATCCCATAGGGGATTCTAGAACCTTCCCGACCTCCAATTTTCATTCTAGTCATTTA
CTTTTACATTTAACGATCGT
TAGTCAATTTTCGTTAATTACTTTAACGCTCGTTGGTCGTTATTTTTGTATTTAATAATTA
ACCGTAACATTTACATTAA
TTATTTGAACATTATTTTTCATACGTAAAATGTGCAATCCCGTGATCTCGAGATTAATTCT
AGAAATACGCTATTAAATT
TGTCCATTAAAACTAACAAAAAGAACATATGGCCCTGTCATCATGGTACCACCAAACAT
GACAAAGCCATGCCAAACACA
CCTCAGCCAATTCGTTGGCTCACGCCAAATGAAAAAAATGTTATTTTATTTATTTATTTAT

TTATTTTTTTGTGCATTGT
GAGTGAGTGAGTATATTTGGTGAGGTAGTGGAACCTACATGTGATGTTATTGGTGTGTT
ATGGATGAGGGATGTTTAGTC
ATTGGTGTGTTATTGGGAGGGGATGATGTGGCGTTGATGTGACATACCTTTGGCATGCA
ATAATACGTGCTCTAAGACTA
TCCGAATTTTAAATAATGTACGTAACTTTGAGGATAATAAAAGAATTTGATTCTTTTAAT
CCCGGCTCTTAATTAACATC
CAAATTAAAGGGGAAGAATAACGAATATTTTTATAAAACAAAGTGCATCAAACACGC
ACCTGTGTTCGTGTATATATAT
AAATGAATCGACTACTTGGTCCTAGCATTTCTAAGACAGTGAGCATAAAACTTTCTTAG
TTCTATTATCTTTTGTTAAGT
>CsCML32
GTTTCTTGGATGAAAGAGAAAACCAAAAAAATGAAGCTGTGAAAAAAGAAAATAATT
CGGAAGAATAAATGGTGGTGGTT
GGTGTAATAATTTGTTGTAGCAAATGAAGGCATCATTAGAGTGGTGTTTGCAAGTTCAA
AAAAAAACCCATCCTTGCAAA
CAAATTAAAAATGACGTAAACACCACTTATTGAAACAAATTTTTATGTAAAATCCAGAA
AATGAAGGTGGTGTTTGCAAA
TTAAAAAAAAAGTCATCATTTTTTTTTAAGATCCAGGAACTTATAAAAACGCCACTTA
TTGTAAGTTGACCTTTTTTAA
AAATTTTCATTCATTTTAGTTGAAACTTATGTAAACACTACTTACTGAAACAGATTTTT
GTGTAAAACCTGAAAAATGA
TGGTTTGATCACTGAATTTGTGAAGAACACATAGATATAATTAGGTTCAACAAGACAAA
AAGTGTTATAGATTTATAAAA
GATAGTATATGAAAAGTAGTTTTATAGATGTCATTAGTTTGTTTGAGTTGTACAATAAGC
TAACTTTATTGTAAGTTGAT
TTTTTTAGTTGAAAGGAGTGTGCAATAATGAAAAAGAATGTTTGTTAAAAATAAAAAA
ATGAAAGAGAAAATGAAATGAC
AATAATACCCTCATACGACTGTCACATGAGAGCCATATATAGTTAAATTTAACGTCCGTT
TGGTTAAAGGACTCTTAGCG
TTAAGAGTTTTGGAGTTAGGGATTATCTATGCAAAAAAAAGTCAAAAGACCAAACATG
TGAGTCAACGCAAAACACAAGA
CCATCTTTGTAATTTACTCTTTAGTGAACTACTTTTGTAGCTCAAAATACTTTCACAAGG
CCGACCTTAAACTTTGACTT
GTTCTTCTATCTTAAGTTAAAGGGCTGCTACTGTGCATCTCGGATATTGTTATGAGGAAA
TTTCTTGGCACAAGTCAATT
TTAGGAACGTAGCAATCAAGAAAATTGTATCCCGTACGTTAATATCTAGTTAACAAATTC
ACTGACATAATCTTTATTGT
TAATCAACTGATGTAGATACGTCACGAAAATAAAACTGTAATGGTTACAGTAAGATACA
TAGTTTTAATAAACGAAAATA
GTAATTTTTAAAGAGATTTCAACACTTACATAAAGAAGAAAAATATGAGAGGAAAATA
GATAATCTCATGATCTCATCGA
CAATTAAAAGATTTTTGTTAACAATACTTAAATGGTCACGTCTTTGTGACAATGAGTTC
AAACCATGTAGATTAATCGGT

CAAAAAGGTCGGAAACTAATGTTACCCAAAAACAAATATTAATTTTTGAAAATTGATAT
TGTAAAATGATTAGACAAAGA
CAATAAAAAAGGACGATTCTCACATTCATTTCCTAGAAATTTCTGCTGAAAATGTTAGC
TGAACCAACTTTGTCCCATCT
AACCATGTATGATGTATCCTAAAGTACTCACAATCACAAGTAGGCTTGAAACTTTCATC
TAGTGACATATGGAACGGGTT
TAAGAGATGTATCTTTAAAACGTTATTATGATTGGCATGATTTCTACTTTTATAATAAGAA
GAAATCGTGATGAAAAGTC
GTCATGATCGAAAAGCACGTAGATTTTTTTAGGAACAAGTTACACATCTAATGAGTCAA
TAATTAAAGAGATGAAAGAAA
TTTAACCATCATACATGCATGCTAGCTTTTGATGTTTTACTGCCCACAACATATCCAAAG
ATAGCAAATGCACGTAATCA
CCTACATCATCTCATTTTTTCTAAGAAATAGAAAATCTGCTAACAAATCATTTTCGTTTTT
TTCTTTATGCCCATCGTAT
AAATATATATACTACCGGCCATTATGTCAATGAAAACTTTAAATTTAATTATTAGTTATGG
ACTATATATGCCATCCACA
TGCCAACTTTATTGGTTAATAAATCACCGGGATATTGCCAGGAAGCTCACCTTAATACTT
TTGCATAAGAATAAATCAAA
>CsCML33
TTGATATAGAATCCAAACAAGGTCCCGAAGGTGGTCCAGTTCAGGATCCTACTTTATAT
TACAGTCTTGCAGGTGGTCTT
CAGTATCTCACATTTACCAGACCGGATCTCACCTATGTTGTCCAACAGATTTGTCTTTAC
ATGCATGATCCTCGCGAGCC
ACATTTAGCTGCCCTTAAAAGTATTTTATGATACGTTCGAGGGACTTTGGATTTTGGTCT
ACACCTATATTCATCCTCTA
CCACTTCTCTTGTTTGATATACAGATGCAGATTGGGCAGGTTTCCCATCCACACGATGG
TCTACTTCTAGATACTGTGTT
TTTCTGGGCGATAATCTACTTTCTTGGTCTTCTAAGCGACAACACACCATCTCCCGTTCT
AGTGCCGAAGCCGAGTATCG
GGGTGTTGCTAATGTTGTAGCTGAGACTGCATGGCTTCGTAACCTTCTTCGTGAGTTGT
ATTCTCCTCTCTCTACTGTCA
CTCTTGTATACTATGATAATATTAGTGCCATTTATCTGTCTGCCAACCATGTTCAACATCA
ACGGACCAAACATATTGAG
ATTGATATACACTTGTCCGTGACATGGTTACTGCTTGCCAGGTTTGTGTTCTTCATGTAC
CCTCCCGTTATCAGTACACA
GATATCTTCACCAAGGGACTTCCTTCATCCTTATTTGAAGAGTTTAGATCCAGTTTGAGC
GTCCGGCTTCCTCCCGCTCC
AACTGCGAGGGAGTATTGGTCGACATTATGTACATAGGCCCGATATGGATTGGTATAGA
AAAAGTTCTTTTTTAGGCCTC
CCCCTCCCTTTTTTGTCTCTGCAACAACCTCATTGCAGGTTCCACTACCACTTCCAATG
GTAGGAATAGATATCGCATCA
CTACCACCCATATTTGGTTCGTTGTAAGTTTTGTTCCATATCGATTCAAGAATAACTTCTA
TCATCTCATTCCCTATGTT
CTTATACGAACCATACTTGGCAATAACTTGATCACCAATTGTAGCCATAACTTCCTCAAA

GACTTCATCATTTAATAACA
CTTTCCCTCACGATTTTCCCGATGATTGCCCCCCCCCCCCCCCCCCCCCCCCCCCCCCAG
TTAACAAAGACTTTCCCTTAT
CACTATTTTCCATAGCTGGTTTGGATGTGTGTAGCGAATGGATAAATGGGTTGTGTCGAT
TTTTTGTTTTGTTTTAGATA
GGGTTTTACTGTTGTTTATTGATTTGAAAGTATATTAAATGAGGTTGTTTTGGCACTTATT
GTTTTCTATAAAAGCCTAC
TCTAGTTGATTTAATGAAGTTAGAGTTATATTTGCAAATATTTCTTTGAAATAAGTTAA
GAGGGAAATGAAGTATAAT
TGGCAGGAGAGAGTGAGACGTCAAAAACAAAACAATGAAATGGCGATAATACCCTCA
GGGGTGGGGTCATGTAACCCGAG
AACTAACGCTCGTTAGCAAACAATGCTTTTCAGTGGCTGTTTTGCAAAAAACGAAAAT
ATTAATGGCTGTTTTGCAACAA
ATTTCGTCTCTGAGTATTCCTTACATTTTCGGAAAACATTAAAAACATAATTTGCAATTT
ACCCATATTAAAAATATCAA
ATTGCTAAAAGAATTAGAAAATAGATATTACGGTTATGGAATATAGAATAAGAATATGAA
GATTATGCTATGAAATTGGG
AAGGCAAATGTGAAGAGAGAAATTTTGTTTTTTCCATTTATATTACACAACCACCGAAT
TTACAAACCATAATATTCTTT
TAACTCTTTGCATTAAACTTTGTATCATCACCTATTTAATCACAACATATTGAATGGTGAT
CATCAAACGCATATATCTC
CATGCAATACATGCAATTCTCTTCTCTTCCATTATTCACTAAACCAACCATTAACCACCC
TCTATTGCCACCGTATCCAA
CCACACCTTAATTTGCCACCTATCTCCTGATATCAAATCAAAGATCACAACCATTGCCAC
CACGTACGCGATACACGACA
>CsCML34
CAACCCACTCCCACTCCCACCGACCACCCATCCGTTGCCACTCTCACTTCGGCACCAC
AACCACACCCTAATCCCACGTC
AACTCACCCCATGGTCACCCGCTATCGTGTTGGTTCTAATCGTCCTACACAACGATTCA
CCTTCAATGTGTTTACTCTTT
CTCCACTCCCTAAGTCGTACACCCATGCGTTTCGTGATCCCAATTGGTTACATGCTATGC
TAGATGAATATAATGCTTTA
ATTAAAAATAATACTTGGATTCTTGTGCCTCGACCACCGGACGCGAACATTGTTCGTTC
TATGTGGCTTTTTAGACACAA
ACATAATGCAGATGGTAGTCTTAATCGCTACAAGGCACGCCTTGTTGCGAATGGCAGTA
CTCAACTTGCAGGTATTGATG
TTGATGAGACTTTCAGCCCGGTTGTTAAACCAGCTACTATTCGCACGGTACTTAGTCTT
GCTATTTCTCGACACTGGCCT
GTTCATCAGCTGGATGTCAAGAATGCTTTTTTACATGGTTCGTTGTCAGAGACTGTTTAT
ATGTATCAGCCACCTGGTTT
TCGGGATCCGGATCATCCCGATCATGTTTGTCTCTTGCAGCGATCACTTTATGGGCTTAA
ACAAGCCCCTCGAGCTTGGT
TTCAGAGGTTTGCTGCATATGCTACTCGGGTTGGGTTTCAGCATAGCCGTTGTGACTCA
TCGCTATTCATTTATCGTCAG

GGGACAGATATAGCTTATTTACTATTATATGTTGATGATATTGTCTTGACTGCATCTTCTA
CAGCACTTTTACAGCAGAT
CATTACTTCTTTGCACACGGAGTTCTCCATGACGGATCTTGGCCCCCTTAATTATTTTTT
GGGAATATCTGTGACTCGTA
CTTCTTCAGGCATGTTTTTGTCACAACAAAAGTATGCTACTGAGGTTCTAGAGCGTGCT
GGCATGCTTACTTGCAATCCG
TGCCGTACTCCTGTTGACACGGATTCCAAGCTTGTTGCTGATGGTGATCCTGTTACTGA
CCCTACATTATATCGCAGTCT
TGCCGGTGCACTACAGTATCTTACTTTTACTAGGCCGGACATCTCTTATGCCGTTCAACA
GATTTGTCTCTTCATGCATG
ATCCTCGGGAGCCCCATTTTGCTGCTCTCAAGCGGGTTCTACGTTATGTTCGCGGAACC
CTTGCCTATGGTTTACAGTTG
TATTCATCATCGACCTCATCCTTAGTTGCATATTCGGATGCCGATTGGGCTGGGTGTCCG
GTTACACGTCGATCTACCTC
TGGATATTGTGTATTTCTTGGCAACAATCTGTTATCCTGGTCTTCTAAGCGTCAGGCTAC
TATTTCTAGGTCAAGTGCTG
AAGCTGAATATCGAGGTGTTGCCAATGCGGTAGCTGAGACCTGCTGGACACGCAATTT
ACTGCGTGAGCTACATATACCA
CTTTCGACAGCTACGATTGTATATTGTGATAATGTCAGTGCCGTTTATCTCTCATCTAATC
CCGTGCAACATCAACGCAC
GAAGCATATTGAGATTGACATCCACTTTGTACGTGATCTGGTTGCTACTGGACAAGTTC
GTGTTCTTCATGTTCCGTCTC
GTTATCAGTATGCGGATATCTTCACCAAAGGGTTACCTACTGCTCTGTTTGATGAGTTTC
GCTCCAGCTTGAGTGTTCGA
TCCTCTCCCGCTCAAACTGCGGGGGGATGTTAGCGATAGGCTTCATGGGCCTAGCCCGT
TTATGTAGTTGTGTTGTATTG
TAGTCCACTAATACTCTATATAGGGATGATATGTATTGAGTTGTACAGACACAGAATATA
ATCGATTCATTCATTATCAA
TTGTTAGCGATTTTGGACCCGCGGCGTAACAATCAGGAACTTGCCAGGTGATTTTCTCC
GGAAAATGGACTAAACATGAA
ATCTGGCAATACCCCAAGGACTAACGGTGTATTTTACTAAAAAATAAATAGCAAATATTT
TATTCGTAACTACTCGGGAA
>CsCML35
GTACGTTTTAGGTTTTAAACGTTCATCACTTTAAATATAGTGATAGCGTTTATTTAATAGG
TTTTATCTTTTTAATTTTC
TTTAATTTGCATTTAAGGCGTTTTTAATTTGTTGGCTTTTCTCGTAGTTATTATATCATTCA
TAATCCTTTTTCTGTTCA
AACTTCTCGATCACTAGACGTTGATTTATCCATTGCACAGCTCGCTGGGACATAACGAC
CCGGGGAGACTCTATACTCCT
CTGATCTTAATTGCATTGGTGTTTGAGCAGTTTTTAGTCTTTTAGGCTTGACTGTTTAGC
ACCGATCATGACTCATCAGA
ATATGCAAAGGTGGAAGATGACACAACTTCAGGTAACACAGGTGGGACATGGCCAGG
GTATTTTCCACCATTGCAGTATG
TAGTAGCACCTGATGAGCTAGAGTTTTATCAACCAAAGGTGGGCTCAAGCTCACATTAT

GTTGAAACTACTTCTGATGAT
GCAGAGGATGATGAGCCACTAACCCAAAAACAGATTGATAAGCTTCTGTTTGATGCAA
GGCCAAAAGTTCTAGGTTTGCC
TGCAAAGGCCACTCTTGAAGCATTTGGTGTCAAACCTTATGTTGCCAAAACTCAAATT
GCAGCTCCAAAAGGGAAGGGGA
AGGGAAAGGGAAAAAGGATGGGTTAGAAACACCTACTACCCTAGCACTGGTCTATGGT
CTTATCAGCTCTTGGTCTTTCA
GTTTAGTTAATGTAGGCCTTTTATGGCCCTATAATAATGTGTTTTCTATCAACTATCAATG
AATGCTATGCTCACTGTTT
TACTATGGTCTACAAATCACACTGGATTGTAACAGATATTAGAATGCAACATAAATATAA
AAAAATGTAAGCAAAAGACA
ACATTTTCATTCCATAACATAAAACCATTACAACAAGACCCTATTTACTTGGCAATTGTC
ATACCTAATACAACACAAAT
TAAACACAAGCATCCTATCATAATGTAAAATAACTTATCATACAACTGCAACTTCGACTT
TGTAGCCTTCACCTCATCTT
CCAACAGTGACTTTTCCATAGCAAGACCTTCAACCATGTGTTGAGAATGTGGATTGTTG
CTCATGTGTTTCTGCCTTTCA
AAGAACCCAAACACCAACTCTTTGTAGTACTCAGTAGGAAGCTCTACATCAAGAAACT
CGTACACTTTGCAATCATTACA
TTTGACAAATCTCTTGCCCGGATTCCTCCTTGTGGTTGAAGTATGAACCTTTAGTAGAC
GGTTACAGTTACAGTAGTATT
GAATCTTACCACCAATTAGTCGTGATTGTGCTGAGCTTGAGAAACTCATTGATTAGGGT
TTATACTACTTGGGTTTGTAG
CAGATTTGGGGAAGAACAAGTTTTTGGTTTTTCATTTTAATAAAAAGCGTTTTTTTTA
AATATTAGTCCACATGTTTGG
TAACTTGACATAAATGGTCCCCCAATATAATGCAACGAGTAAAACGGGTTTTTTTGGAT
TAACTCTTAATTAATTGTGCC
ACGTGTCCAATTTGGTTACAATAAGAGTCATTAAGAAAGTTTTTTGTTTTAATAGGAAA
ATTAAAAGTTTATTGTCACAA
TAGTAAAAAGTGGAAAGTTTGTGTATTTTTTATACCATTAACCCTACTATAAATGGCATT
ACCTTCAAAATGACTAAATC
AAGAGCAATATTGTTACCAAGCAAACAAATTGACAATTTACGAAATTAGAGGGTGTCC
ACTAATATATTCCCTCATATAT
TTTCTCCTTATTTTTCTCTTAACCACACACACCCTTGATTATAGTAATTCATCCTCAATAA
ACCCTTCACTCTACCTTTA
TCACAATTTTTACAAATTTTCTTAATCTTTTTTTAATACCCCTTTAAACCCAAATCATATT
TTGATCGCTTTAGTTTCCA
TTTTATCGCAACTAATTTTACCATCAATCATCAAACATTTCATTGTGATTTGCTTGATATA
TGTGGCATTTTCATGCAAA
>CsCML36
AAGCAGCTGCGAAACCCTGTCAAGGAGATTCTTCAGAATTATACCTTATCACAGGCAG
TACTTGTACTGATCAACGGGGA
ACCGTGGGCCGTGTACCTCAATTCAACGAAAGTGATCAAAGAAACTTTCGTGCGCTTT
ATTGCTAACTGTTGTATGCAGG

TACACACAACATTTGCAGCTGATCAGAATTGAGAGGTCAATCCATAGCCGCATGCTCAC
ACATCACATTCATTTCAAAAG
AAATCATGATTAGTGCATCAATGACTCTGATATTCTCACTCCATGCTTCTGCTACGCTGC
TGCAAATATCATGAATCAAC
TAAAGCTTCCGCTACTCTGATGAAAAGAAATCATGATCCAGAATGTGAGCATGTCGGTC
CACTGTCACAACATCACATAA
TGTCTACACTACAATGGAAGAGTCAGAGATTTATGCAAGCTGATGATCTCAAAACTGG
CTCAAAACTCATCATCACGTGG
ATCAGTTAAAGACCTCCACTTGAATTGTCCAAACAAGCAAAACATGAAGAATTAAAGA
CTACGAGCTACAGAGCAATGAA
CTTAAACAAGAATCCAAGAACAATCTTAATGTAATATCTGTTTGTATTATGTGTAATTTG
AGGTGACCTCCCTTAAGGGG
AGATTGTTAGGATTTAGAAAATTCAGTCGTTAAAGAGTCGGTCACCTCAAGTATATGTA
TACGTTAGAAAGCTCGTAGTC
GTAGTCAAGTTATGTTAATAAGTAGTTAGTTAAGTAACACCGTGGAGGACGGACGGGT
CTTTACCCACTGTATGCCTGAT
CCACAGCCCGTAGGTGGTGTTTATTGTAAGACACTATAAATACCCTCTACCTCCCAAGG
TAGAGGGTTGGAGCTTCAATA
CCATTTTTCTTTGTTTCACATAGTTCCCAACTCTCTTCCCTTTCTCTCTCTAGAACTTTTT
CTCTCTAAAACTTGTAAAC
GCTCCATATGTTATTGGTTTGATTTGTTTGTGAAATCGTATATCTTACATATGTACTAACG
GGTACAATACAAGAGTAAA
ATAAAAGATCCCAATGAGTCGCCTTGCTTTATTACTTGTAAAAGCGACTATAGATTATAT
TGCTTTTACAGCAAACGGTA
TCGTAACCATTAACACGTTATTCTACTTGATTCTTGCACAATGGCGCGCCATCTCCATAC
CAGCCCAGGCGCTATTCGTG
AGTCATTCGTGTTTGAGCCAGTGAAATAACTTACGCCACCGTTTATTTGTGCTGTTTTTT
TAGGACCACCTTGGGCTTAA
GTTGAAAGAAGAAAGGAAAAAAAATGAAATCGGAATAGCAGGTCGATGACATTTTAC
TAACGGAATAAATGAGAAAGATG
AAGTGAGTAGGTGTGCATTTTTATTCTTGATATTGCATCAAAAATAGTACTTTTATTATTG
GAAATAGCAATCTAGGTGT
TTGGTGCCACTTATTTAATTTACATTAATTTTAATAAAAGTGTGTTTGTTTATGTATTGTA
ATTTTATTTTATGTTTTGT
AATTTTATTTTATTTTTAATAAAATGTGTTATTTTTAAATAAAATAAAAAATAAAATAGTG
TGTTCTAATAACATGTTAT
TGGTGTGTTATGAGGGAAGGTGTTTGAGTTATTAATGAGTTATTGGGAGGTGTATGATG
TGGTGATGAGGTGTTATTTGT
ATCCCTGATAACTTGTGCTCTTATAAGTGATTGTATTCTCACGTTTTAAGTTTTGAGAAA
TTTTGTTTTCTGTTACTAAA
GATAAAGTTTAAAGTTTATAAAACTTTCATTTTTTAAGAATAAAGCATGTATTTTGATAA
AGTTGTGAGCCAAAAAAACG
TTTTATTAATCGGTTTCACGATTTTGCTCGTTTTTACAAAGCGGTGGCGACTGTTTTTCA
TTAATGAGGTAGGACAATCG

TATATAAATCCCCATTCATTCAACACCTCCTACATCACCCTCACCAAAAACAAACTCCTT
CAATAATAAAATAAAAAAGC
>CsCML37
AATCTCAAATACAAAACAACGTTATCGGAAAAACAATATAATCATTCAAAATATATTCCT
TAACGCTTAAAATACTTATT
AATGAAACAAACTTAATCGCCGCTAAATTTTTGTCGAAACTAGATACCGCTGCCGCTAA
GCACGGGTACTCGTCTAGTAT
ATATATATTATTGTTAAAATTCTATTCCATAGCTATTAAATAAGTGTTTATACATGTTGCAA
CGTAGGGGATATATACCT
CGCTATTCACAACAATCAGTTACATTTTCAGTCTAATTCTTTTTTATTACATTTAAGTTGC
CGGATAAGTTAGTCACTAA
TAGAAGATCATGTGCATAACAAAGATCTCTATTTACTTGGTTTTTTATAGCTCATTTTACT
TAACTAGGCAGTTACCCGT
GCATAAATACGAAATTTTATAATGACAAACGATTATAATTATAATATTATGGTTTAGTGTT
TCGTGTAATGTTTATGCAT
ACAACGTAAAATAATCAAAATAGAATTGAAAAAGTTGTAATACGCGAAATTCTACCCAA
AGAACAATTCTAAAATGTAAA
AGAGCGTATAAAAATATTTAGTAAAAATAAAACATTATATCAATTCTAAAATGGCATGCA
TATTTTTTATGTTTCGGTAG
TAATATTGTAAATACAACTATTCATTAAAACTAATTGTTTGAATTTTATATTAAAAAGTAA
ATCAAAAATTAAAATTTAA
GTTTTAAAGTAGGATCCGAGATTTTGTTTAGGTTATAAAGAACCCTCCAAATCTATAAG
GCTAGCCCCAATAGAGGCGAT
TCACACTCGATCGTGCTATCTGGCGATCGATTTCATATCGATCGTGTTGTTGTGGAAGAC
ATTCGATCGCGTCCCATGTT
AACCAAATCAATCGCATGCAAGATCGATATTTATTGTTGTATCGTTGCATATGAAACAAA
AAAAAAAAGAATCATACCCT
TACTGGGGCACCTAATCGCCTGAAGGTCGCTGGAGAGAGAAAGCTGACGTGGCAGGG
AAAATGAGGGGGATTGAATCCCC
CTACTGGGGGCAGCCTAAGTAGTACCCTTCTTGTTTTATCATTTCTTTTTATTTTTATAAT
TTTGTTTATATTATATAAT
TGTAAAATGAAAAGTACAAAATTAAAAAAAGTACAAAATTTAATAAATCAAAAGAATT
AAACTAGCAAAAAGTACAAAAC
ATAAACCGATAAAAAAACAAAAAAAAAATAATTACAAAATATTTGTATTTATGTGAGCA
GGAGGGTAGAACCGTTTTTAC
AGTAAGGGTCCGTTTGGTTTGCTTATAGAAATCACAATGGATATTGGTATGATAAATTGT
GGTATGGAAATAAGTTTGAT
AGTTCAAAAAATGTTGTTTGATAAATATGTAAGAAAAGAGTGGGGTGAGTGAAATTAA
CGGTGAAATATGGCTCACATGA
ATGGTTTGTCAAAATCAACCAAGTTTAAATGGTATGGATGTATGGGTTTCAACTATGGG
CCCTATAGAAATGGTTTGTGA
AAACTCCTCAACCAAATAAACATAAATGGTATCAAACTGATTCTCACACCTTTATACAA
AATGGACCCTAAGTTTTTTAA
GAAAAAATGTTAAGAAATGATACATGGACCTAAATTTAAGCCTAAACTTAGACCTAAAA

CTACTTGACATCATTTTGATG
GATTATAAAATAAGTAAGTCTCTGTCTGCAGCTTTGGGACGATGAAATTTAGATACATTT
GTATTATTTCTCAAAAATGT
TTTATGTGCGATAGAGACATTAATTAAAAATTAAATAAACAACCCTACCACACCACCAC
AGCATATAAATAGTCTCTCTA
CCTCTCCTACGAACCTTCGTGGAAGCTCACTCACAATGCCTCTAATTTCTCCATATAACC
ACCCTCACACCTTCCTCTCT
TTTTCCATCAAACAACAATCTCCATTGCCAAATACTAATTACTTTTCTTTTCATTTCCCTT
CCTTTCACTCCCAAAAATC
>CsCML38
ACACCCCCTCAACCTATTCAAATTCAAATTTAAATTGTACTGGTCAATTCTTGGCGGGAT
AAATTCAAATTCAAATTTAT
TCAAACTCAAAATCAACTCTGCCTATATATATAACTCTACCAGAGTTGTTTAGAACACAC
AACACACACAAGTTACACAC
ACAACTCACACACACAAAAATGTCTCGCAAAGATTTTTCTCAGCAATTCTCCAGCCCA
GAAAAGTTGCCGGAAACCAGCT
TTCCAAATCCAAATCACTTGCCGGAAATCAGCTTTCCAACTCTACCAGAACATGATGTT
TACTCTACCCCACCAGAAAAT
GTCTTTCCGGTGGGTTTTGAAAACATCCCGTACCTCCCAATTGTTTCTACTCCACCTGT
GAACAGCCCACAGGTGGATTT
TCAAAACCACCGAGCGGGTTAAGTGAGGCTTCCCTCCGGCAATTGGAACGAGATGAA
GAAACCGTAAGGGAGATTATGGA
AGAGCAGACACGTGAAGAGGCTGCTCGCCGTGAGCAAGAAGCACGTGAAGCTCAGT
ACCTGCAAGACAACTTCTTCGACA
TAATGGAGAGTCGATACTTTAATGGGGAGTTTTATTCTCCGCCAACCAGTTCTGATGGT
TGGGACTCAGACGACTCGTTT
TTTTAAGTTTTAGTAGTTTTATTTTCGTCTGTATTTTTAATTGCAGTTTTTTTTTTTTTTT
TTTTTTTTTTTTTAATCT
CGTATTGTAATATGTTTTTAGTTTTAATGAAATGTTTGTTTTAGTTTAATAAATGAAGTGT
TTGTTTTAGTTTAGGGGTT
GAAATTGAAAATATGAAATATTATTGGTGTTATGGTGTTATTGGGATGTTATGGATGAGA
GTGTTTTTTGGGGATGTTAA
TGTTAAATGTTAAAGCTGATGTGGCGCTGACGTGGACGGTGTTATTGGTGTTATGGTGT
TATGAATGGGAGTGCTCTAAA
TGCTAAAAAAGACTTTAAATTTTCTTTAATGCTTTTATCTAAATTCTTGCTCCAACATGA
TGTCGAATGTTCTCGGTTGC
ACTAACGACATAAAAGATATATTCTTAGAAAACGATTTTCAAGACACTCACCGTTCACC
ACCAAGACTGTATTTCAGGAT
GAATTCAAGCTTATCCTCAACGCATGCGGTAACAAAACATTGACGAAAACACAACATT
ATCTTAAATAAACATTGCAAGA
CTAGAATACAACTACAATGGTTTGTCTTCATATTTCCATATGCAATTATATTTCACATCTAT
TTGCTTTTGCATGTATAT
CTTTATTTGTTTTATGCAATTGTGATATCTTATGATATACGAAAACTACTGGTTCTTCTGT
TTGCGTCACGCGAGATTGT

TTTTTTCTCGAGAATTTATCGATGCTATTGAATTTTATTCATGATTTAGTGAAATATAGTAT
GTAAAATGGGAGGTTAGA
ACTTGTCGATTAGTTTAAGGAGATGATGTTAATACTATTATTAATTAAAACAACCCTAAC
CCAAGGCCTTTCGTCTAGTG
GTATTTGAGTTGTCCACAAACTTAAAAGTTGTAGGTTCAAGCCTCGCTGCATGCATTCG
TGAGATTATATTGTGAGATAA
GTGGTATATGGTGTGAAAAAAAATGACTTAAAAAAAAACCCTTCCCAAAGTGTGTTGC
GAATTTTTTTGGTATGGATCAC
GATAACTTAGCACAAGTTGATTCATCTCACAGTTAAATTTGCCGATAGTGGAATATAACT
AGTGACTTATTATAAAAAAG
ATGTTGGAATGCTTAATACAAATAAGTGAGTCAATCGAACCCATTTCAACCACGGTTAT
TAAAAGTAAATGGTTTTAACT
AGAACCCATTTTAACCACTAGCCTAAAGTGTATCATTCTTATTTTTTTTTGGCAACCAT
GATTTCCGACCTTTTGGCCG
ACCAATCCACTTACGCATGTAAAGCCTATGTTTTCCTTTTCACTTTTGGTAATTCATTTAC
ATCTTCACTTGGGTTGTCA
>CsCML39
CAGGCGGCACATGAATTAAAGTATATGGCTTTTTATGTCCTAAATCCTTGTGTCTTTTTT
TAATAGTGAAGTTTTCGTTA
TTAAACTGATAACATAAGTCAAGTGGCTTACCCAATGCCACGAAAAATCAATCTGAATG
AACCAGCCAATATCTCTGAAT
TCGTGCAGCAAAAAAGATTCAAGTAGTACATATTGCACATTATTTGTAGTTAACCATCTA
TGAGCCAACTCAGCATTTGG
AAACCGTAAATTCTAGTCCACTTCTTTACTACTTCCGTAGTTATTTAACCAAAGTAAGTC
CCTTTTGGACTTTGCGTGCC
AAACCTTTATAGAGAAGTTGACAAGGCAAGCTAGTAGAGTACTTGTTATTTGAATTTAT
ATCGAAATGACAACTTTTTTG
AACAAGACTGCAATGACAATTGACAGTCATGAAAATAATATTGGGCGTATCATCTGCAG
TAATTGCTTAAGGAAAACTGA
GAATCTTGACTTGTTGTAAGCTGTCACAGCAACCCAATGTATGTGATTTGGCTCTTGTG
ATTGTATGTTTTCTATAAGGG
GGAATTGCATATATACCCACTAAAACTCAAATATTTGCATATTTCCCCAAAATACAAGTT
TTCAATGCATGATGTACAAT
GCAAGTTAAGCATGTTTTGGGTATATATGCAAATATGCAAGTTTTAGTGGGTATATATGC
AATTGCCCCTTTCTATAAAA
CGTATCACATGACTCTCAGTAACAAACAATTATGTCACTTTCTAAAACTATGATGCATAG
AGATTTCGTTTTGTGTGTAG
ATCAACATACGTAGGAATCACATGAACCGCTTTATCATTGATAACGATAATCTTTTTTAA
AAACATCTTTGTGCTTGAGC
ATTTAGCCTCGTTCAAAATAGATCCCCAGCATACTGACCCATCAACAAGCAGTCAAAAT
TATCTCATCCAAGGATCCTGT
TCCAATATGTCTTACTAGAGCGTTTGTCCTTGTTCCAAATAAGTAGCTTATCATTGCAGC
AAATCATTGTGTGAAGATGG
TCATAATGATTAAACACCGGCCATTCCTGGTTACAGGAATGAGTAACTACAAAATTTTT

GGACTAAAGAGTTTAAGGCTT
CTTTTTCTTGATAACTCATAGTTATAAAAGATTTTTAAATTAGGATTAAGGTACTGATAA
TTTTATTTCTTCATATTTT
GCTCATTTTTAATGTTTATATGTCAAACAGTGACATATACACTACCTAGAAACTTGTACT
AGCCTACTTCACTTGGGTCC
TAGCCTTTTTGCACTTTTCGCCTGTGGCGAGATGCAAGTTTGGCTGCCTCAGTTCTGTT
TTCGTCTTTAACTACCCTGCC
TTTTATCAAATATCGTTTCTGTTTGGAAAACAGTAGCTCATATACATAATCAATGCCACTT
CCATGATCCATCAAATATG
GATTTTATAATTACTAAAGTGGGAGTTGGTTCATTGGAGAGTTGTATCAGCCAGATACG
AATGCAATTAAGGTAAATGTT
GTGGAAAAATGTTTCTGGGATATTGAAATGAATTTTGATTTGAACGAACCAATCAAAGT
GAATTACTGATCTGTCTTAAG
TAGATCGGTTAAGTGAATCTAATTTATCTAACTAAATAGGTCGATGCAGAAATCATCATC
TGCTGTATTGAGAGACCCAA
GTTACTTTTGATCTTCAGTACATGCGCTGAAATACAGGAAGGGACATAATATACAGGAT
TACTACCAAGGGTTTGGTTAT
CTATAAAAGGTGTAAGGATGTTATATGCAGAGAAGTCAAATTGAGTTGGGACTTTATAC
TTCTCTCATTTCTTAATCTTT
TGTATATAGGGGTAGTAAATTAGCGGTTACTCTTTTGACCTCTGTTAGAGGGAAAATTTG
TTTAACTGGATATTTGTCTT
GTAGCTTTAGATTTATCTTCAATTTACTGTTTTCGTTTTGTATTTTCTGCAAGTGGTTCTT
ACATTTGATTTCTTTCTCA
>CsCML40
AACCCTCGCTTTTATTTCTTTAGTAGTTAATTTTATCGAAATCATATTAAAATATGTGACA
CCTAGACTTAATTTAAATC
CGCACACATAGCTCTTGGGACGACCTCGGTTTTACCGAATACTACACTACACACGGCC
GGGTACACTTCCCCGTTAGTGC
ATTAGGGTAGACTATAGGTATAACGAGTAATTATAAATATTAAAACCTGGTCAGTAAAAC
CTGCATCAGTCTCCCACTTG
AAACTCCAGGGTCTTACGTCTCTTATCGGCGTAGCTCTTTTGTCTATCACGTGCAGCTT
GCAAACGATTACGAATTTGGA
CTACTTTCTCAGTAATTTCCAGCACGAGTTCGGGAGTGGTAAGCTGGTTGTCGCCAACT
TCCGCCCAACAGATCAGAGAA
CGGTGCGGCCTTAATGCTGGTATGATAGCTATTGTTGTATGAGAATTCGGCCAAAGGTG
GGTACTTCTCCCAGCCTTTGT
CGAAATCGATAACACAGGCACGAAGCATATCTTCTAGCGTCTGGATGGTCCTTTCGCTC
TGAATGTCGATTTGAGGATGA
TAGGCAGTGCTCATGTCAAGCTTCGGTCCCAAAGCCATTTGCAGTGACTGCCAAACGT
TTGACGTGAAACGGCTATCTCA
GTCGGAAATGATAGATATGGGCAAAACTACTATTCATATATATATATATATATATATATAT
ATATATATATATATATA
TATATATATATATATATATATATAGTGAGGATTAGGTGCGGATTTTTTAATTCGTGCGGAT
GTGCGGATAGATCTGGAC

CACCCATTCCAGATCTGATCTTGACTTTTTGTAAAAAAAGTCAAGATCTGCGCTAACTT
CACGGTACCAATTGACCATCT
AAACGATTTTTTTAATTTTTTTTCCTTTTGAGGGATCGTTACTATTGTTATCTTCTTTCATT
TTTTTATTTTTCCAGATT
GTTCTGAGCAATTTTTTGTTTTTTTGATTATGAGTCCTACCGTGATATTAACGATATTATG
AGTCCTACTGTGATATTAA
AAAACTATGATATTATAAAATGGTGATATTATAAAACCAACTGTGATATTAAGCTTTTTTA
CATGTAAAAAATAAACCGT
GATATTAAGAAACATATGATAGTTACTATGATATTAACAATTTTATTAAAAAAACATATGA
TAGTTACGGTGATATTAAC
AATTATATTAAAAAAAGAAAAAACGTTTCTGTGATATAAAAAGTAACCGTGATATTAAA
ATTTTTTTATTAAAAAAGTTA
CGATGATATTAATAATATTAGTAAAAAAACAAAAAAGTACTGTGATATAAAAAAAGTTA
GTGTGATATTAACAATTTATA
TTTAAAAAAAAATTATGTGATATTATGATCAGATCTTGACTTTTTTACAAAAGTCAAGAT
CTGATCTTGACAAAGATCAG
ATCTTGACTTTTGTAAAAAAGTCAAAATCGACCTTAATATCACAATACCTTTAATCATGA
AAACGATTTTTTTTTTGAA
AATTTTTTCAACGATTCTTAATAACTTATCATCCTCTTTATTTTTTTAATTTTTTCAGATAT
TTTTGATTTTTTTTTTAA
AAGAAAAAGCTACTGTGATATTTCCGCACCATCCCTACTAAAAGCCTAAAACCTACCCG
ATCCCTTCTTTATATATATAT
ATATATATATATATATATATATATATATATATATATATATATATATATATATATATATAGATTT
GCGTTCATGTAG
CGACCAATCTTAAAATAAAAACTATAAGAACCAATGAAAGTGTGACAAGTGTTAATTA
AAAGTTTAATTTAAATAAAAAT
GATAATCTTAAATATAAAGATATTATGGGTATTTGATGCATCTTAAATTCCCACATCCTTA
AATCACTCCTTTATTGCAA
CGACCTCCATCAGAAATCTCTTAAAAGAATTACATCTCTCCATTATCTTCATCACACAAG
TCTTCATGTACAATATGAAG
>CsCML41
ACCCAACCAAGATGATTCAAGCTTACCAAATGAAAACCTAAACACTTGTGATGATTCTA
AACATAAAAGTACCAACAAAA
TTCAAACTTTAACACAAGAAATCACCCAAATGATGCAAAAACCACTAAACTTTTTCAA
ACTCAAAAACCACCTAAAAACC
ACTATAAATCCAAAGATGTGCAAGATATGATCTAACCTTGGCTCTTGATACCAATTGTAA
GATATAACTTTTCACATGCA
CATCAAACCATAACACAAAGTAACATATAGATGAAATTCTAGAGAGAGAAAGTTAGAG
GTTTAGGGAACTTAGTGAGAGA
AAGTGAAAGATATTATTATGAGCTCCAACTCTCTACCTTGGAAGGTAGAGAGTATTTATA
GTCTTACATAACTCGCCACA
CTTTGGGCTGTGGATCAGGCATACAGTGGGTAAAGACCCGTCCGTCCTCCACGGTGTT
ACTTAACTAAATACTACTTAAC
TTAAGAATTACTACGAATCCGAGCTTTCTAATGATTACATATACTTGAGGTGACCGACTC

TTTAATACCGGGAATTTCTA
ATTCCTAACAGTTCTTGCCCACGATTACGCCAACCGTCTTCCTGGAGCCGATTTAACGT
TCACATTAGAAAACATCCCAA
ACCCTAAAACAACAACACATATCAAAAAACACCCAAAAACATCAAACATTTGAAATAA
GGTTGGTCAATTTCCCTTACTA
TAGACCCTAATTACATGAACATAATACACCTAATGCACTAATAAACTAAAAACCTGCAA
CACTAACTAACCAAAAACCTG
ATTGGTTGCAAAACTAAGATTGGTTGCAAAACACATTTTCACTTGCCATCAAGTTATTA
GTACTACCCACACAAAAAGCT
TAAACAAACATAAATACACATCTATAAATCTTCTTCAAAAATATGGAAAGATCATGACA
ACCATCAAGTTTTTAATCCCT
CTAAGCCGACGGCTCGGCTCGGCTCGGCGTGTGAATAGTTTCGGCTCGAACCGCGGCT
CCACATTGCACCCGAGCCATCA
TCCTCCCTGGAGCCGAACGACGAAGGGACGAACGACGCTGGAGCCTGAATGGTGGTG
CAGTATGGCGCCATTGGTCCCTA
AAAAACCCAATGGCTAGCCATTGGTATCCTTATACCGTGCACTCTAATAGTGTTGACGG
TCAATGACATGTCAAAACATA
TGATATTAGATCAAATTTTAAAAGTGTTGTATATCCGTACTTTTTTTAAAATTATTACTCC
CTCCGTCCCAAAATTATTG
TCCCCGTTTGACTTTTTCGGTTTTTTTTTTACATCTTTGACCTTAAATATTTTTCTTTGTG
TTATATGAAACTTGATGCA
AAATATATGAATGGATTTCATTTTACATGTATTTTCATTGATATAATTTTTATCAACTATTG
TATAACACAAACAAAAAT
ATTTAAGGTCAAAGTTGAGAAAGAAAGACCGAAAAAGTCAAAATGGGACAATAATTT
TGGGACGGAGGTAGTATTATTAC
TTTTACCTAGAGTTTATGCCAACCAGCGTACTATGTACCATATAAAAATGTAACGTTATG
AGTATCACAAAGGATTTATA
TACATAAAAGAAATAAGAAAAAAAAAAATTGTCAATTAAGGTTATAACATGAAAGTTTG
GCTGTACCAAAGTACTTGACAA
CTTGGTTTACTTAGAAAGAATTTTATAGGTACCAAATAGATGATAAGGAAGACTTGCTT
TCCTAAAGCACACAAAGAATC
AAGAATAACTTTAATCATGCACGTAGACCATTTGTCATGCCACAAGCCTTAAATTGTTC
CCAACTTTATATCTCCTAACA
AGTTGCTTCACACAATATTCTTTCCTTTGATACCATTTAGGGCAGGCATCTAAACATCTA
CCCCAACATTTGGTATATTA
GGTACTCTTGTTCATAGATAGCCATCTAAACATTTAAAAAAAAAAAAAAAAAAAAAAA
AAAAAAAAAAAAAAAAAAAACA
>CsCML42
CTAGTTTTCTTGCAAAGTACATTTAGGATATGATAATAACTTGATATCCTTCCTTAATGGA
CAAACTTACACACATATAT
TGAAAGACACTATTCTAGACATACACACGCAACCTTAAATAAAACACACTAATACACGT
ACACCCGGCTCTTAAGTGAGT
CCGTACTTTATCGCTCGAATAAGGCTTTAAACCAACTTCACAGAATGTTGATAACTAAC
AATGCTAATAGATCTAGACGC

CTGCTTTAATTCTTCAAATTAAGTGATGATTCATTTCATTTCCATTTTATTATCAAAGCTA
CACCATCTTATATGAAGGA

AATGCCACTTTAAAGAATTTATGCCAAACAATCTTGAAAAATACATTATTGCTTTATTAA
AATACTACTTGATGCTTTCT

AAATCCATGAAATTAACTTAAATATGAGATGTAGCTTTGGATCTCACGACAAGGTCATT
ATCATAACACAACGCGCTTAT

GCCTTATGGGGCATACGCATTCTTCAAAACCTGACACGTTATAAAAGCACAAAACATTG
AATATCACTTTGTTTATAAAG

AAAGTTAAAGAACAATAATAACTTAAAAGAGTAAATTGCAAGTTTGGTCAAATTGCAG
GTTTCATTCCTAATTTCCTAAA

AAGTGTAGGCACCATTTTTGTGGGAAACTTTGGCTTGTATTTTCGTTCCTGAGTCACAG
TTTTTGACAAGGGCATTTTTG

TCATTTTACTTTGGACAAGGACGAAAACTGCGACACAAAGTTCCTCATAGAGACGGTA
CATGCACTTTCGAAAGGTTAAA

GACGAAACCTGTAATTTAGCAAAACCACAAAGAAAATTTTTGCAATTTACTCTAACTTA
AATTATTCTTGTATATTCAAA

GTTAAATAAAATGGTTGAAGTCTATTTTTTTTTTTTTTTTTTTAAATGATTCGAGTCATGT
TCTGTCGCTTGAGATATAT

TAAACAGATCAACTAATCAAACCTTAATCGATTAAACATGTCGATTAATCTAACTGTTGT
TGATTAATCAACCTTTAAAG

TTTTCTCAAAGCGTATAATCTATAAAACGAGTTAAGTGGGACGATCAAACACCCGTTTA
ACAACCCATTTAAATAACTGA

GTTGAAGGTCGTGTTCAGGATTGTGTAGACAGCTTGTGGATGACACATGATATGTCTAG
TTATGTTTTTTTTCTTTCCTT

AGAAACAACATGTCTGAACACATTTATATCATTCATCTAACAAATACTTTTAATGAGACT
GCTTGAAGTCACAAAACTAT

CGATTGATGATTGATGAACATATCACTTTGTACGAAGGCTTGTGTATGTTCTAGTCAACC
ATTTAGGAGTGACACCGCGG

AAAAGTTTGTGACTCTCATTACTAAAATGTGACTGATCTTAAGTTCAATTCATGTTAGGT
GCAAATAACTCTGAAGTGCA

CATGACAACCTCTTTAAACGATAGGCTTTATCCATTTTTCGTCTGAATAGCGGTGAATTC
AAACCTTATAAATTAGTCGG

TCATCCTAATTTTAACATTAATTTAGTAACTGATTTTTTGTGTCCGCATAATATATTTTGAA
ACAAAGAAAATAATATCC

TTATATATTAGCTTAAAAATCACTTTCCAGTTATTTCAGTTTACGCGTTTTGTGGGTACTA
CTTACAAGTCATTACTCAT

CACAACCGAATTTATTATTCCAAAGCAATAAACAATGAAAAAGATACCAAATAACGCGT
GAAAACTCACCTTTTGTATTC

ATAATCATCAAAAAACACTCACAAACACACATACACAACTCTTTTTCTTTGAACCTTCT
TCTTCCTAGCTACAAAAAAAT

GGCTATATTAACCACACTTTTTCTTGTTCTTCTTTTCATTATTGGCCTTGTTTCAACTCTT
TTAAGAATACCAACAAAAA

AAGTTGCAAGTTTATCTAAGCAAGATATACCCAAGGCTCAAACCCTAGAAGAAAATTG
CATGAATACTAACAAGGTAGTG

>CsCML43

TACTTTTTGAAATTTGACAAAAGTAAGGGTAAAATAGTCACAAAATACACAAAATAATC
AAAATTTGAAAGTTCAGTACA
CACAATAAGAAAATAAAAAGTAAAGTACACATAATAACAAAATTGACAAGTATTATACA
TTACACTTTTACATAATAATA
ATTATCTAAAAAACATGACATTAAGTTGATTCAATTGTGGAAGAAATTAGACTTAACTC
CATCATAATAATCAAAATCAA
AAGGTAAATACAAATTTCAACATACAAACTTCATATTACCATAATAATACAATTATACCA
AAAACAATCTTTTAAAAACA
GAAATTCATAGTTTCATTCCAATAATAAACATAAGTTAAGCTGTTAATCCAAAATCCAAT
ACAAAAACTGCATATGCTTA
AATACTATACGAACTAGCCATTTTTCGTCGCAACTCGATCAAACTCATCTGAAAGGTTC
CTCACAAGCCACTTCTTCCCA
CCACCTGCCTCCTCATCAGCCAGCCCTGCAAATCGCCTCTTTCTCTTTTATGCTATCGGC
AGCAGGACTACTTAAACTCT
TTCCCGTCTTTTTGGAACCTGATTTTTTAAACTTAGGGCTGCTTAAGGACTCTTCTCTTT
TCTTAATACTTGTTTCAATG
TGGTTTCGAATATTTTCAACATCGCCTTCGATACAGCCTTGCAACCTGTGGCGATCAAA
ACTAAACCCACCCATTGGTTT
TGAATGCACACGTACGTATAAGGGCTCGATCTCAAACGCTTGTTTATTTAATTTGAGAA
GTAGAAAAGAATGCGGTACGA
CTTTTATAGGATGATGATACAAGGAAAAAAAGAAAGTCGATTTACGTTTGGAAAATCTT
GCCATGCATGACATGCAAACG
TACTATGACCGACTAATTAGCTTTTTTCTTCAGCCCATGACATAAACGGGTCAAGTGCTA
AAACGGGTGCCACTACAACA
AATATGTCATTTCTACACGCTTTTTTCTTCGCACTTTATAAAATGTGAACTTTTTTTTAAA
GATATTCACACTTAAGAGT
GTGAATAATTGTTTCAAAATCCTCGCACCTAAAAATTGTGAAAATTTTCAATCTCACAC
TTAAAAGTGTGAAGAATTATT
TCAAAATCAAAATCATCACACTTAAAAGTGTGAAGAATTATTTCAAAATTAAAATCATC
ACACTTCAAAGTGTGAAGAAT
TGTTTTAAAATCATCACACTTAAAAGTGTGAAAATATTCATAAAGCATCACACATAAAA
GTGTGAAAATATTTGCTACAC
CCAATTGGTTCACACGCATGCTAGTGTGAAGTTTTTTATTCTTCACACTTTAAAATGTAG
CAAGATGCTCTAATAAGTGT
GAATATGCCGCTATTTTGTTGTAGTGGTGGGTCGACCCGGTCAGTGTTGTATGGTATAGA
TTCTAGTCCAAAATAGTTTT
AACCCGTAGGATATAAATATAGTTATACCTTTTTTTGAACAATCCTAAAGAAACAAAATA
AACAATGAGAGAGTTTTGAA
ATATTCTTTTCCTAGTCAAAGTAAAAGACTAAACATTTGTCTTAACTAGGTTGTTTTATAT
TTTTTGCATAAAATTCCTG
ATGTATCTTATTTTATGCGGGTTTTAGTTTGGTAGTCTTGTTGGTTGTAGGTAGTTGTCAT
TTATGGTCTGTCATAAATG
TTTTGCTGGTTGTGTCAAGCGATTGCTTGCGGTTAATGTGGTACTTCAGTCACATTCCAT

TGTACTTAAATGTTTTTTGG
AGTAATATATTTTACCGGGTGGAATACCCTTTACCAAAAAAAAACTAACCGGGTGCAAG
GATATTGATCAATAGAAAGAT
TTCAACAAAACAAAATATGGCACCAAATGGCTATGGATACGACTTCTACATTTTTCCCA
TACATATGCCCATTTTTTGTA
CCACTACTAACCAGAATCTTATATATATCCAGCACAAAATATATTTATCATAAACTCATAC
ATATATTTGTCTTTCTATA
>CsCML44
CTTATGTGATCTGGGCTGTTGAGATTTGTATTCAATTATTGAGTTTAATTGTTTCTTTGAC
AATATTGATCTATGATATA
TGATATTCTTTTATAAGGTTAGTCTATGTTTGAGTGTATAAAGATTTAATCTTTTTGGATT
GTTTTCGATTTATATGATC
TGGGCAATTAAGGTTTAATTTTGGTTTTGGAGTTGGGGGGTGTTTGTTTTGGCTTATATA
GCGGGCTTATGACTTATGTG
GTTTTCGGCTTATGTTTCGTAAGTTTAGTTTTTGTGGCTTATTTTGAAACTTTTCGGCTTA
TATAAGTTTATTATGACAT
GATAAGCTTATATAAGCCTGCAAAGTTTCAAAATAAGTTACAAAAAGTGAGCTTATGAA
GCATAAGCCGAAAACCACATA
AGCCATAAGCCTGTTATATAAGCCGAAACAAACACCCCATTAATTAGCTCTTTGATAATC
TTGATGTATGCTAGTTTATT
GTAAGGTTAGTTAGATATTGTCTCATTTCATGTTGTGTGTAAAGTTTTGATCTTTAAGATA
TTGTGATGTATTCAATTGT
TTATAATCGAGTTATAGACCATCTCCAAGGTGTCCCCAAAGTGTCGGAAGAGAGTAGTG
TTCAAACTTCTTCGGGTGAAC
ATCTTGAGATGGCCAGGGAAAAGTATGGACACGGAATACCGGTTAAGCCGCCTACATC
GGAGCCAAAAGGGCTCGACTTC
CCTGGGTTACTTAAACACGGATCCTTCTCCGTTTACTTGCTTACTTGAATTGTCATTGAG
TTATGAACTTAATCAGCACT
TTGATAATCTTCATGTATGCTAGTCTTTCATAAGGTCTTGTGTCTTAGCCGATAAAGGTT
CTATCTTTTCGATATTGTGA
GGTCTATAGTCACTTATTAGCTTTAGGTTTTTTGATAATCTTGATCTTTGCTAGTCTTTT
CACAAGGCTAGTGTATGTC
ATATGTCTATGAACTTGTTATTTAGCCCATAAAGATGCATGTTTTTGAGATTTTGAGGGT
ATGAACTTGTTTGTAATCAG
TTATTGTCGATTTATTTGATATGGGTAGCCAAAGTTGTGATCAAATATTGAAACTTAAAT
CAGTTACCTGACAATCTTAA
TCTATGCTAGTATTTCTTAAGGTTACTCCACATTTTGGTTACTTATCGTGTACGCAGTGA
ATATTTTGTTATATTTTGAG
GTCTACAATTGTTTATAATCATATTATTGACGATTTATTTGATACTGGTATGTGAGTTATGA
TTGGATCTATGAACTTCA
ATAGTTCTTTCACTAAGTTGACCTATGTTGGTCTTTAACAAAGGTTCTTGTTTCTTAAAC
ATTAAAGATTTAATGGTTTT
TGATATCTTGAGGGCTATAATTGTTTTTTATCAGATCTTTGGTTTATCTGATCTGTGTACT
AGATTAATTATTGAATATT

TGAACTTAATCGGTTTTTTTGACAATTTTTATATGTGCTGATTCCTAAATATCTAGCATTA
TATTTTGTTCTGAGATATG
AATCCTCTTGGTTGACTTTTGTTCCTGCACCCTTATGTGGCATCAAAATTTGACGGTTAC
TAGTATAAGATGCTTTCTAT
GACGATTTTTATAGCTGATAAAACTTTTTAAAGCTAGCCAATTTTTGAATTCTTGAATGG
TTATGTATAATGTGTCAAGT
TTGTATTGAACGAGATCACCAATGGTGAAATCAACTTTTTGGCTGCAATGCATTCTGGT
CTTTTGGATTGACAGAGACAT
TGAATGTGAAATTTTTGTTTTTGGCATTAAATGATTCTTATTTGGTTTGTGTACTGTTAAC
AGAAATAGTTGGACGTCAG
TCATTTGATATCAGCAGCATCAGGAAATGGTCAAAAGGTTATTCCTATATTTCATAAAGC
TTTTATCCTTGTTTTCATGT
TCATACGGAGGTAATCTAACTCATCTATAACCGTTGTTTTCAGGAATAATAATGAAGCGT
TTTGATTTGCAACAAGTGCC
>CsCML45
ACACATATTAAGATCAAATGTGAATGTTTAAAAAGAGAAAGTGGCAAAAACATGAATC
AATAAAATACGTGAATTAGACA
TGTTTTTAGTCATGCTTTTTAGATCTTCACATATTGCTCTTAAATCAACACCGATGCACCT
TCATCCTAAGAGCACTAGA
TATGGTGACTCAAGCCAAAGGAAAAAAAGTGCGTCACGTCATGCCACTTCAACATTTA
TTCTTTTCACTTCTTATCACTC
ACGCATCAAAAAACCTCCTCACTAATCACTCAAGCCAAAACTAGGTCTCACCATACCC
ATCAAAACACAAAAAAAACAAGG
GGCAGTTACATTTACGTCTTTACTAAAGTTGAATATTACATCAATGTCCTTGTCATTTTTG
TAATTACGTCAATATCCTT
ATCAAACTTTTTTAATTACATCAATATCCTTTTCACTTTTGTAATTACGTCAATATCCTTCT
CAAACATTTTTAATTACA
TCAATATTCTTCTCAAACTTTTAAATTCTCATACATATATCTCAAAATCAACATGTTCTTT
CATCACTTGAGGCTAAATA
ATAATTTTTATATATATTTTTTGGGAATATCAGAGATGAATATCAGAGATAAATAATAGTTT
TTGAATTGAAAATCAATA
TAATTATAATTTAATTGATTTCTAAATATACGTGAGAATTTAAAGTTCAGGAAAGATATTG
ATGTAACTACAAAAATTTG
AGAATGACTAATTATAAAAGTGAGAAGGATATTTATATAAATGAGAATGATATTTACGTA
ATATTGAACATTAAAAAAGA
TATACATGTAATTCTCAAAAAAAAAAAAAAAAAAAAAAAAAAAAAAAAAAAAAAAA
AAAAAACACCCCACTTTTAGCGCA
AATCAATGGTTTTGCTTGCACAATGACACAGGAAAGAGAGGTATGATAGCTTGGTTTTG
GCTTAAGGAATAAAATGTCAC
CTTGGTTGGCTTTGGTGGAATGTGACCACACATGGTCATGTAATAAGCCAAAAACTCA
ACAAGTAGTAGTATTATATTAT
CCAATTAAAATATGGCCCCATGAAGAAAACAAGAAGAAAATGTACTAATAAACAAATAT
TAATATCTTCAAATTTTGTCA
ATAGCAATACCTCACTTTTCTTGGTATATTGGGTCTATTCGTAATTTCACCTAACTTAATT

TCATTGGAATTTCCCCTAC
AAAGAAGCATCATTCCCTCCTACCTTTGTAACAGAAATAAGACTTGGTCTTTACAATTG
GAAAATGACATATGAAAAAAA
GAAAAAAAAAAGAAAAAGAAAAATACAACTTTTCCAATTGCCTTTCTAAAAAGAACA
ACAAACTTTTCCAATTATAGTAT
GTTATCATCTTATAGAAAGATTTTTAGTGAAAATTATTTCATTTTTCACATGTAACACCAT
TTACATGGCAATCAAAGCG
TTGGCGAACATTTTATGCAAGTGAGGCGGCTGCATAGGGCCAGTATTTCTATGAAACCT
CCCTTTTATAGATTATCAAGT
TATTTGTGCATGAATTTAAGCTTTTGGTTAAATTTATTATGATACAATGTTATATGATCACA
ACCAAAATACAAGATTAA
GAAAACATCAACAATGAAAAAGTCGACAATTCTTTATTTTTTGCATTAAAATTCGTCAT
ACATGTGGCCCACTTATCATT
ACCGCATAGGGTCTTCGAAATTCATTGGACAGCCCTTAATCCAAAGTTTATACCTTTTCC
ATTAAGTTAATCTTACTACG
TTTTATTTCTACGACATGTACATGTTGCATACATCGTTACATTTGTTAAAGTACGTTGCTA
TTAATATTATCCTAGTCGA
CATTGTCGTACCAAATGGTCAATTCCAAAAGCAAACACCCTCCCCTTTCCATGAATATG
CCACTCATGGAATTGGTCAGC
TTCCTCTTCATATATATAAACAATCCATACATCAATTCTCTTCATCACAAAAACAACATTC
AACAATATCTTAAATTCCA
>CsCML46
ACGCTCTGATACCAATGTTTTCCAGGTTCTTTGCCAACGTTTGAGGATTTCAAGATGTG
ATTGTAATTAACCATGTTTGG
TTCTGGTTATTCGAATGTAAAACTAGGGTTTGTATTGAGATAAGAATGATAGAGATACA
ATGAATATAAAAGACTTGTAT
TGATATAAAACGTGTTGATATGCTTCTATTAAGAAGGCATATTTATACATACTGATACGAC
ACTATCTAATCTAAACGAC
ATGACTAGTACTTACAACGGTTACAAACAAACACAACGGTTTGTTTGTTGATAACCGTT
TATCCTACTTAAGTTACAATA
ACAAACGGGCTGAGTTTGGATCAAGTTTGTGATCCAAACAGTAAATAAGTTCGACCCA
CATAAACTAAACATAACTAAAA
GTTTTAAACTTAAAACATGATACTGGTTCTTGATTCTTTGTCGACATTAACATTATTAGA
GGTTCTACAGTTTTAATCAT
TGATATGATTTGGAAAAAAGAAAATTGATAAGTACATGTTTTAGATAAGAAACACTGAA
TTTGGCATCTTCACTGTATGG
ATATATTGTACATGGAAAAAACCAGTGATTATTATTTTTTTTTCTTTTCTTTTGCAGTCA
CCGTGAGCGAAGTTGAGTC
ATTGTATGAGCTATTTAACAAGTTGAGCAGTTCCATTATCGATGATGGCCTTATTCACAA
GGTATTTTTAAGTGATGATG
ATCTTCGAAACTTTTTAGGTAATCAAACTACCTACTTATTGAAAGAAATAGCGTGCAAA
GAACAACGTCTCTCACAACCG
GCCTTCATAGAATTGCCGAGGTCACACTAGTGCATAGTAAAAGGAACACGCGAGCGGA
AATGAGAAAAGAGTGAAGAAAT

GAAAAAAAACGAAGAGAAAATTGGAACATGTTAATATTATCATAGAGGAGTAAAACGG
TAATTAACCAGAATATATATTT
ATCAAAGATAAATATATTTAAAATATAAAAAATAAAACCACAAACCCTAGGACGTTTTT
GGATCGGGTAAATTGAAAGAA
ATTGGAGTAATCTGGAATTAGAAATCCACCTCATGCATGGTTTGATGACAACTCGGAGA
AATTTAAAATGTTAAAAAGGC
TTTATGTAAAGCAAATATAAATGCACAGATTGAGCAAGGAATTTTAAACGACACGGAA
CACATAAGTTTTATAGTGAGAT
ATTGGACAAAATTTCCATTTCTCATTTTATTCGAATGATTTTAAATCACTATTTTGACTTC
TTTCTTCTTCATTAATATG
CTTTTTAATTTTCATAACTGCTCCATCATCTTCTTCGGCTAGACCCCGTTCCTTGAAATTC
TTGCTTTACATCTCAATAT
CTATTCTTATAGTTTCAATTTTCTTTATGAACTAAACTTTGGAGTTCACCCAAAACAATT
TTGGGTTTAGGTAAAATAAC
ATTTTTTTGTTTAAAACAAAAACGATTTTATGTTTACATATAAGTGAAATCGGTTTTATAG
AGAAGTGAATCTGGATTTG
CAAATAAACATATTTGTGTTTACATATTTGAATTTTGGTTCCTAAAAACTCGATTTATGGT
TTAAAAAATCAATTTTTCT
CTACATCATACAACAATCGTATTTGGTTTACATACTGATGTTATAGATCGAACGGAAATG
AGAAATTGTTTGTTGTAACT
AATTCTTAAAGTGCTTGGTGTGGTCGTGTTATTCCTTCGCCAAAAGACCCAACAACAC
ACCAACATGTTATACCACGGCC
ACGCTACCTATATAGTCATTTGTCTGCCATTGGTGAATTAATGGGTGCAATGGCTGGTGC
CATTTGTCCCACTTTTTTTC
TTTTTCTTTTTTTTTTTTTTTTTTTTTTTTTTTTTGTGCCAATAAGTGTTATTGGGTGGGAGA
TAATGTGGCACTGAGATGA
CAGACGAATAATGTATTGTTGGTATCTCCCACATCAAGCTCTCTTATCAATGTTTTTATTG
GTTCTTGCGGATGAAGATA
>CsCML47
AACGAAAATAAACCTGGTTGACAAAGGTATTGACCTTCACATTGTTTTATGTTTCTTTT
GTGAAGATAATTGTGAATCGT
CAAACCACATTTTCATTGAGTATAAGATGGTGAACCCGATTTAGGACTCTATAATAGATG
GTGTAAAGTCCATGTTGCTT
TGGTTAGATCCCCTGATTTGTTGGAGTTATCGGGTTCAGTTGGTCTAGCCTCGGTGTTC
ACAAAAGCCTTTGACGTTGTT
ATTCGTATTACAGTATAGTATTAGTATTACGTATTACGTTATGGGTGATATGGAAATATCG
AAATGGAAAGATTTTTAAA
AACAAAGATCTTGGCATCAAAGCTGTGGTAACGGAGATCCAAGCTATATCTCACCTTTG
GATTTATAGCAGAAGTCGCGG
GAATATTGCTAACTGAATGATTGGGTGTTATACCCTTGTGTAGGTCCTATCTTTCATGAC
TGTTTGGTTTCATGTCTTTG
GTGTTGTACTTTTTCTGTGGTGTATGTTTGGGTTGTTTCTCTTGTTTGTTTGTCTAGCTTC
TTGCCACTCATTTTGTGTT
TTTATAAAATTTGTTGTTCGAAAAAAAAGATTTATGTTTTTTCTTATTTTTTCTTTCTTG

TTTTACAAAGATGCAACGA
AAATGTGCTACAAATTTGTGACTTTAACACAGATATATCTATAATAAACATATTCATACAC
ACATTTATCGATTTAACAT
AAATATACGATTGACATTATTGAAACATTTAGTGAGTTTTTTTTTTTTTTTTTTTTTATAA
CACCGTTCGTACATTTTA
CCAAATGAAAAAACGTTTCATGATTCAGATTCAGACTTCTCAAGCAATTTTTAACAGTA
AAATATGGTAGTTAGATTTAG
AATAAAAACGTTTTATTGAAACAAACAACAAGTTTGACACTTGACGGTTACATGGTTT
GATGTATACATATGTTTGCTAG
CTTTTATAATGATAAAAACAAATGAAATAGAGCTTTCAGTACAGGTTTATTATTAACTTT
ATTAAACGTTCATCATTCAT
GGGCCTCAAACCTTTCCTTTTTCGTTTTCTTCCTAGCAAAGTTGCAATACATTGAAAAT
GATTGCTTTTTTGTTATTGCT
TAAATGGTTGTTTTCGTTTGAACTTTTTGATATTAATCTCATATAATTTTTAGATTGAGAA
TAAAAGTGATTATTGCAAA
CTTTTTTTTTTTTTTTTTTTTTTTTTTTTTTTTTTAAGAATAAGTTATTAAATTGATGAGAA
GTTCATGCTGTCTTTTTC
TTATCAATTTTAGTAAATGTAGGTTTCCATTATTGAAACAAGATGGTGGCTTATGATAAA
TTCAAAGTTAGGCCCATAAT
ATGTTGACAAATATTCTGCAAGATGTAAATTATGTGGTCCTTGATAGTTGATCTCTTCTT
AATGTCTAGTTTTATCCAAT
GTTTTAAAAACTGGGTTTTAATCGACTCGGTTGAGTCATTGGCCACCGGTTCAACCGTG
GGTTAACCCAATTTTCGATTT
ATTATTATTTTTTTCGACCAGGTCAACCGATTTTATCATCAAATTAACCTTGTTTCTATA
AATAAAATTGAAAAAAATC
AAACCTGACTGGGTTTCAAGACCTGAGTCAATCAAAACCCGACGGGTCTAATCGATTA
ACCCGGTTTTAAAACCCGGGTT
TTTTTTTACTTGTTTTTGTGGCCGGTACCGATCTGACCGGTTAAACCGGCCGGGTCAAC
CCGGGTTTGAAAACATTGGTT
TTATCAATTGTCTATGATTTGGTAAAGAGTGAGCCACGTCTTCCATATTTCTCTTGAATG
TCCGTGTCTTTATAACACGT
GCTTATATCCCAACTTTATATAGTTATATCTTACACAATATATCCAAAGTGATCAAAGGCC
CTTTAGTGATTGACTTATT
TCAGTTGAATATCATCAAAAGGGTTATTTATATTAAGTGGATCTATATATCAATCTTTGAA
ATTTATATATTTCAGCTGG
>CsCML48
TCAATGAATTGAATATAGGACTTATCCCAACTACATGATTTTACTTTATGGATATTGTTAT
TAAGTAAATCATAAATAGT
TATCCTCAGACTTGAGATACCAGATAAGTGTCATGAATGTAGTGCACTTCTGTGGAACA
ACTTAACGCTATACGTAACTG
GTAGTCATAAGGGTTGTTTCCTGAAGTGTTGCAAAGTTCATGAGTTATTCTTGTAGTCA
AGATAGAATTTGTTCCTTCAA
AATTTATTTTGGAGTTTAATACTGCTGGGCCCCTCGTAGGGTTGACAAGATGTTTGCGT
GGCCACGCCCAAAGTTCTCCC

CTGAATATGTTCAGAAGATTTGGTAATCTTGCTCAATCACTAATAACGAGAAACAGAAT
AGTTGGATAAAGAATGACTTA
ATTCCATATCTTTATTTAACATGGTATTAGAAACAAAAGGATAATAATAATGAGAAATCA
TTAAAAGGTTTTCCAGGAGC
CTTGCTCGAGTTGTACTAGAGGCATCGAAGTGTTTCTAGACGCTAGCCGAATGTGATCA
CTCTATTAAGATATATCCAAG
TGGGAGCTGTTGGTTTTATGGATATAAATTAGTAGACGTGAGTTAGGATCGAACTCGAG
ACTATGGGATTGCACACTTTA
CGTATGAGAATTTAAAGTTAAATAATTAAATATAAACGTTGTATTTATTTATTTATTAAAC
GAAATAACTAACGGACGTT
AAGTAATTAATGTAATGGACTAACGGGCGTTAAAGGTATTTGGAAAAGGACTTAATTGC
AAAAGTTGGGAAGGTTCCGGA
ATCCCTATGGGATATCCAAACCGATCGGCCAAGGAGAGAAAGAAGGAAAGAAAAACC
AACTTGTTCACCAAGTTTGCTTG
GGTATATAAACAAGGCTAGGGGTTTTATTTTTCCATCTCTTGTGCTCATTAGCTAAACCC
TAAAAACGTTTTGTGTGTGT
TTTCTTCTCCAATTCGATCGTACATCACCTTTGGGTGTCGATCCAAAACTTTTCCACTAA
CCAAGTTATTTTGTTTGATA
AAACTAGCATTACTTTAATTCGGTTTAGTACGTGTGTATAAGAACATAGTAATAACATAC
TTTTGTTATTATCTTGGATT
GAATTATAAAACGGTAAGGGGTAATTCACATAATCCTATAAACCCTAATTAGGGTTCATC
CTAATTGCTAGTTTGTGGAT
TTGATTCTTGTTCGGCTAGAATCAAGAAAGGTACGCATATTCTTCTTATGTTAATTAATTA
TTCGTTAATCTTTCCTATA
CGCTTCCGCTTGAATACTTTGATGTTTTGTGGTTATTTATTTGAATGTCATGTTATATAATT
CCAACATAGTCAACTATT
TTCATTATTTAAACGTATTGCATAGCTTTCACTGCTGACTAAGTTCGTGGATTGAGTTGA
TGCTTCTAGCATATTTATTG
AAACTAAAAGGCAGTTGAGGTGCTAAACTGAACCAATTGTTGACGGGTCTATATATGA
GTTCGGAGAAAACGAGTCAAAT
ATTTGCATAGATAAGAAATTTGTTCATTTGTAAGAACTTGCAGAAAAACTCTTTTATTTC
TCCAAAGTAAGAAAGAAGTT
ACTCTAAATGGTGATGCGCTAAATACTCAATGATATGATTCTTACTCATCATATATTGAAT
TTTCTTTGTCCATATTTGT
TTAATTTTATAGAACATGCATGTTTATGGTGTGATCATGAACACAACAGCTTTTTTTATTT
TAATATTTTCATGGAAGTT
ACCAAGAAGCTCTCTAGGCAACTTCGTTCCCTTAGAAAGTTAGCAAAAAGGACGACA
ACAAGTCAATTTCCATCAATTTA
CTTGGAATATAATTTAAACAAGTGAGGCGGTATGTATCTTCCTCAATACTATGATTGTTA
GGCCATCTTTTTACAATGAA
TTATCTACAAATAGCACGAGTTACCAATCCAGAGACACAATTAGACTAATTAACTCTCT
CTTGCATTATTTTCATCTTAT
>CsCML49
ATAGAACAAGTGCTACAAGTTATGAGGTAAATCAAATCGTCACAAGTGCATTAAAATCT

TCGTGTCAATTTGGTCGAACA
CATGTGTCGTAATGTTTAAGTTCAGTTTTAAATGTAATGTTTTTAATTTATTAATACAATT
TTTTAGTTTCGCTTGTTTA
CTTACGTTTTTAATTTATGAATAAAAAGTTTAATTTAAAAAAATAGGTTGAAAGGTGAG
TAAATATAACCATTAGAATTG
AAAGGTGAAAAAGCGTGACAAAAAATTAAAAGAAAAGAGAAACTGAAGTAGCGTTG
ATGTAAAAGTTAGGGTCCGTTTGG
TACCATGGGTTAGGCAATTTGGATTTGACTTAAAGTTAAAATTTAATGTTTGGTTGCATA
TTTGTACTATTCAGTTTAGA
CTCAAAGCCAAATAATTGGTCATTTAGAAAAGTAGTATGCACGAGCGGGTTTACACGA
ATTTCTTGGTTTTGTATTTTCA
AGTGCGCCCGTATATTACATGTACCACTAATTTACTTTTTTATTATTGTTTTAGAAATTATT
TTTAATTTTTAATTATAT
ATATTTATTTTAATAATTTATTTATTTATATTATATTTTCTCTTTCTTACATCAAATTCAAAC
TCAAATCCTAATCTAA
ATCCAAATCCTAATCCTAATCTAAACCCATCTACAAACCAAACGGGCCCTCAATGTTTT
GAAACCCGGGTCGACCCGGTC
GGGTTAACCGGTTGGACCGGTTCCGGGCAAAAAAACGGGTTAAACTAACCCGGGTTT
CTAAAACCGGATTAACCGGTTAG
ACCCGCCGGGTTTTGAGTGACCCGGGTCAACCAGGTTCTCGAAACCCGGCCGGGTTT
GGTTTTTTTCAATTTTTTTTATA
AAAACCGGGTTGACCCGGCGGTTGAATCGGGTTAACCCGGCAGTCGAACCGGTTATTT
TTTTTTAATATTTTTTAATGAA
AAACCGGGTTGACTCGGTGACTCGACCGCAATTATAAACCCTTTTTTCAAAACATTTGT
AAAAGTACTTAGGGTCCGCTT
GGTTCAAAGTGGCGAAAGGAAAAGGGAAAGGGAAAGGGAAAGGAATGCAAAGGAAT
GAGTGAGGTGGGTAATCAATACGT
TACCCCCCAATTTACTCTATAATGGTTCATTACCCACTACGGGGTAATCAAAACCTTACC
CCTCCCCAACCAAGCATCAA
TTTATTTGTGATGTGGGCCCTTTTGAATACTTACCCATTCCCCACCCCCCCTTAACCCCT
AACCAAGCACGCCCTTAGTA
ATTATTTGTAAAGGACGTGAAACATTATTTCATGTCGACGCTTTTCCCCCTCCAATAAGA
CAATAAATTATTTATGAAAT
TAATTGATCTTTACGTCTTAAATATCATATCCATAATAATAATTAAAAATGATTATAATTTG
CGGTTGAAAGTCACCGAA
AAGGGGCTTCATTTGACTTACCATCACCTCTAACATCCTTTTCAATTCCAACAATCTCCA
CGACCAAATTCAACCTCCTC
GTCAATTTAATTTAATGTAAGTTTTCATTTCATAATCTCCATTAAAATTCATTAATCCCATG
ATTAATTAGTCCTAATTA
ACATACCCCATTTGATTATTTGATTAATATTATGTTCATATTATGGAAACATGATTATTATT
CCATGTAATAAAGTTGGC
TTTAAAGTTAGGTTAGCATATCCACTGAATCAGTATGTATGGATTTTATTTACATTAATAA
AAAACATGTTTCTTTATTG
CAATTGCTTTTTGAATATTATTATTTAATGTCAATGAATTGGTTGGGTAGCATCTCCACTG

AATCATTATTAATTAATGT
TGGGTTGTTTCTAGATTGTTTATTACAGGGATATAACTCAGTGGATTGGTTTTTTTAAGA
TTGTGATTAAGTGCTGTTGG
TTTGGAGGGTTGTTCGAGCATATGCGACGAGTATACGTAAATGTGATAGTGCAGAAGTG
CGTATTGTTGTTGGTGTAATC
>CsCML50
AAGAAAGTTCATGCTTTTCGTTATTACGAGTTATTATGTTTAAAGAGTTGGATAGCATAG
GTCTGTAGATGTATTTATTA
AATGTAAACGTTAGTTGCATGTAAATGCTAGTAGAATTAGCATTTTTTAACTTGAAGTTT
TAAAAAAAAGCAAATGTTAG
TTGGTTGTCACGCAATCCTTGTATACAAAACGCTACCCTAGACGCGAGTTGCACTAAAA
ACACTCACAAAAGCTCGCTGT
CAAACATCACATAGCAATAGATTTGGGGAAGCATATAAGGTAAACAAGTTTTGTATGAA
AAAATATTGAAGCTTATCGAG
AACTTGTCTTAATATCCAAGTCGACAGAAAGAACTGTCGTACTAGCTAATACCCACATG
CAACTATGAAATATTACTTGG
TCGACAAGTTTGTAAGTTCGATGAGCACACGAATACACGGTTTGGACCCACTTGTTAC
TCCATATTCTAATCAGTGTTGT
TATAAGTATTTTGCGGCTTTTGTTCATCTATTCACCAAAGTCAAATTTTGCCTCTTAAAAT
TACAAATATATAGTCAACT
CGGCTGTAATGCAAATATAGCCCTCTTCATATACATGTTCACACGCTATAATTATTCCCTC
CGTCTCATAATTTAACTCC
GTTTTAACTTTTTGGGGCATTTACATATATATCCTTGCAAAATTCAAAAATTACATGTATA
TCCTTTTGATGCATTAAAA
GCATTGTACTTTATGCATCAAAAACTGGTAAAAGGGATATATATGTAATTTTTGAAATTT
ATAAGGATATACATGAAATT
ATGCCTAACTTTTTAAATCAATTTTTTTTTTAAATATTTTACGAAAATTATATCAATGCA
AAATACATTTAAAACACAA
TTCATTCATATATTTTGCATCAAGTTTTATATAATACAAATAAAAACATTTAAGGTTAAAG
TTGAATTCAAAAAGTATAA
ATGAACTTAAATTTTGGGACGAAGATAATATATAATTAGACGTTTTAAAAAGTTTAGTAT
ATGAATACCTTTCATATTAT
GAATTTAATTTGTTAGAAAAAAAGAAAGAATATATAACATTAGCTTGAGTATGTGTTTTC
TTTATATTTTGTTTGTTAAT
TTCTTGAATAAAAAGTTGAGCCACCCTAACTTGTAAAGGAGTAAAAATTATGTAATGAA
GGGACATATTATAATTTTGTT
TTTTAGCTAAAATATATATCTGAAAATATCTCAAAATACAAAGGGGTGGCATTAATTAAT
CGAATGTGATATATAGGGAA
TAACGAGTGTTGGAAGACCCACGCGATGCAGCGGGATTGCTTAAAGGACGTGCTTTGC
AACGACACTTAAATCTCAGGGG
CTGTTTTTGCCAAGTTGCGAAATATTATTAATGCTATAGGACTTACTTTGCAACGACACT
TGAACCTCAGGGGCTGTTTT
TGCCAATTTGACAAAGTTTGCATTTAATGCACTTGGTTGTGCTACAGTACTAATGTTTTG
CTTGTTGTGCAAAACTTATG

TACGCTTTTTAGTATTATATACTAATAAATTTTTATATTTTTTTAACCGAGACTGTTAGACA
ATTAGATTATTGGCTCAA
ATTTGATCGTGGGTTTGAGGTTTATTAGATTTAAGGTTTCTGTTAGCTTATTGTCAAAGC
TATAGTTTATTATAAATAAA
CATGCTGCTCTGCTATGTAATTGACAAGTGATTCCATCAATACTAATAATGTTTACAGAG
ACATATGCAAATAAAAAGTC
AAGATATAATACCAAGTTAAGGAAGAGCCCGATATTATCCAAACTTTAAAATTAGAATAT
AAAGTCAAACAAAATGATAA
GGACAAAATGTACGAACAATACAAAAATAAAGTCAAAAGAAGAATGAATACCAAGTC
AAATGGCACTTTCGTAAATATCT
TAGCTTTCCATATTTATATTCACACACCAAAACCTAACTTTCTCACTCACAATTCGTATCT
CATTATTTCTCCAAAAACC
>CsCML51
TTTACTATTCGATTCATGTATTTCGTACCTTTGAATATTGAATTGATTTGTATGGTTAATAT
GAGTGGCTAAGCACCTCT
TGGTGCTGGCCTAGTTATGATTCCTTAATTGTTTTGTTTGCGTTTATATTACTTCCATATAA
TTATTTCTAAGTGTATGC
ATGCATAGTTTATTGGTAGCCACCCTTGGTTCGGTAGTTTTGAATGGCGGTAGAGGCCA
ATTGCCTAGGAGATATGACCA
ACTGACCCTAGGTGAGCGGACTGAGACGGGAAGGGTAAGATATGACCAACTGACGAC
CCTTTCTACTTGTGCGATTTGAC
CAACTAATCACCTACCGTTTGGATGACATGTCAAATCTGTTGATAGCTGTAATGATTTGC
TCAAAATTATAGGGTCTGCA
ACCACCCATGCTTAATTGCTGTGTGTTGTATGGTCATCAATTCACCATGGTCTATTCCTT
GTTGGAATCCAAGAATTGGC
TAGCCCACAAGTATATGGCTTGTGGTAAAGGCAACTGCATCCATATTTGGTTATCAATTC
ACCAATTCTAAGAATTGACT
AGTCCTGAGCAAACGGCTACAGGTAAAGACAAGTCAAATCCGTTTATATAGTTGCTGAT
TTGCTCATAAATCAGATGGTT
ATCAATGTACCATAGTTTACGAACTTAAACATCTGGCTACTCCAGTAGTAATGGCTATTG
GTAAAGAGACATCAAATCCA
TTATTTTCAATGATTCACCTCTCACAGGAGTTCACACACCTGGAAGTGATGATAATAGT
ATGCAACTAATAAGTTTTGAT
GTACATTATGTCATTTGTTGCTGCAAAGACCTGGAGCTTCAAAGTTTGATCATCATAGA
GATGAGATTATTATTGGTTCT
TTGCTACTGATGTTTGAGAACAATCCTCAAGTACTGAAGTCTCAAAAGATCTTTCAAGG
TCAGGAAACTGGAACTTAACC
AAGTCAAATTCCAGTTTAGGGGAGGATTGTTGGGAATTATGTGAAGTAGGAATTCTGCT
GTCATTACTGCCGAAGGTTTC
TGCTGAAGTAGAAGCAGAATCTGCTGAAGAAGTGATTCTACTGAAGAGTAAAAGCGTT
GCCGTGTTACTGCTGAAGGGTG
AAAGCTGACTAGGATCAGCATGCGCTTTATGAATTAAATGCTAACTCATTATTATCTTTG
AACGGCCCGAGGTTAGCATG
GGTTTTAATTGTACTGCTCAATATAGTTTAGTGTATTAGTCATTAAATGCATGCTTGCATG

CTTAGTCGATTGGGTAGTA
ATAGATTAGGGTTCCTTGTTTATAAAAGGAACCTCATAGTATGTAATATTGATCAAATCT
GAAACATAGAGTTTCATTTA
ATAAAAAGATCCATAGAGGATCTAAGTGTTCTTAGTTATAACATCTTGTATTTACTTTCC
GCTATATAATACGAGTGTTA
GAGACTGTCTATTAGTTTGTTTATGATTACATAGACAACACTAACGATCCACGAGTGCTT
AAGATCTGTCAAGGTCTGAG
AGTTTACAACAGCTTTACCGAATACTACACTACACACGACCGGGTACACTGCCCGTTAG
TGCACTAGGGTAGACCATAGG
TATAACGAGACTTTATAAATATTAAAACCTGGTCAGTAAAACTTGCATCAGTATCTTTCT
TAGCCAATCCAAGTATGCAC
GTGATAACTTGAGTCAAGTTGGGGCAATATGAGCCATTGGATTTGATCCAAAATGACTC
AAATCAAAAGAACTTCTTTTG
ATTTGAGCCATTGGATCAAATCCAATGGCTCATATTACCCCAACTTGACTCAAGTAACTT
GAGAGGATCTCATCCCCTAA
AAAGTTTAAAAGCAATTGTTGAAATAGTCAACATTTACGAGCCCACTTTTTGTAAATTA
TTTCCCAATCTTTTAAAGCAA
ACTTTCTTCATAGTAGTTGTTGCACTGGTCACCAAATTCACCCCAAATCACACAAATCC
AATTCAGGGGTTTTTCACAAC
>CsCML52
CTCATTTTAGTTCTATGATGTATGTAGGTTGTTTTGCTTAAATAAATTCCAAGTATTGTTG
TTATAAGAATTTATGGGCA
TGTCCATAATCCTTGGTTATTTTGGATGTAAAAGGTTGGTTTTTCATATATATTTAGCATT
TCACCCAAAAAAAAATAATA
ATAAATAAAAATCATGAGACATGCACATTTATTTTATTAGCAATTATACACGCACAAAGA
TGTTTACAGTTTCATTGCCA
AATTTACTTCTTTTTTGTTTAAAAACATAATATTACCTATTATTAGTTATGAATATTCTCTT
TTAAGATATACTCATGAT
TTCAGGAGTTTTTAAACTAGTGTAAAAGTAACTTTGAGATAGAGCTATGTGAGATAAAA
CTATGAGATAACATTAATTTA
TAGATAACAACTTATGCATTCTATAGAGTTATCAGTTTTGTGATAACTTAGTTTATTTGGT
TAAACATCTATCAAAAGGA
TTTGTTATTCATACGCTTCTCATTTGTTTAAACAAAGATAAGCATACAAATAAAGGATGA
CGTACACTAAACGTACATAT
CACTTTATTCTCGTTCAATTATTTGTTGTTGTGTATTTGACACCACATAAAAAACCTTGT
TTATTCTAATTTACAAAGAG
AAAGTAATAATAATAGTAATAATAAAACTATTTATTTATGCGAATAAAACTACCATAAGGT
AAATGATGATGATAAAAAG
TAGGTAGCAACCATCCGGTAGTTCGTAGTTGGGCACGTGATCCCTCACTCATTGGGACT
TCTTCTATTTTATGCACCAAA
TAATTAGATTAGATTCCTTAAACTTTTAGTATAAAAGGTTTCCTCAAAAAAAACAAATTG
TGTTGTTTGTTTTCATTCGG
TATATACTAAAAAAGGTACGATTTCATCACCATCGTTATTATTGTAGCTCACTCCAACAA
TCTTTTTAAGGTTAGATTTA

TGTCATTTATATGTTTTTTTTTTTTTTTTTTTTTTCCATAATTTAATATCGAAAATCTTTTT
ATTGCTCTTGCTGGATA
ATTTCGCTAAGCTTATATGAATTTATATGTTGAATAAGGATGAACTTGTATATGCTGATTT
AAGTTATTCTTTTATGGTT
GAATAATGATCATGTATTCATTATTGCAAGTAAATAAAAGTTCAAACCAATGAAGAACC
TTAATTTGTTTTTTTGCATGG
GAGCATATTTATTCCAGTTATTGTAGTCAATCAAAATTTCTAATCTTTTAGTGTAAAATCT
TGAATTAAATAATTTTCAC
CACAAAGTATATATGTAACACTAACTGTGCTGAACTGCTGATCTATATGTACCGGTTGTC
ATATAATCAGTTGTTTGATA
CTTAAAAGGTTCTTGGAAGTATGTGATTGTAATGATCAGTTTAATGAATCTTTGGCATTG
CTAGTTTAAGTGCTTATTTG
CCATTTTAAAGTAAATAAGCATTGAAAACTAAAGAGGTTCACTACTGCTTATGGCTTTT
TTAAAAACAAATTTTGAATAA
GCAGTCCACTGAACACCTCAAAACAACTTTTACTACTTAGTTTTCGAATTAAAAGCAA
CAAACAACTCTTATATGTGTTT
ACAGATAGCTGATATAGGAGATAACAATAACATATAATCACATATAAGAAACTAAATACT
ACGTACTAAGTATTGATCAT
ATTGTTCGATGGAAAACTGATTTCCATGTACTTGTGATATCGTTATACTTGGAATTTTTTA
TCTTCATGTAATCTTTTTA
TAGCAATGTTTACTAATGAACCTTTTCCTACTTCAAACGGAGATAGACTGATTGATCCG
CATCCAATTCGCTAAAATTTA
TATCTCATCGGCTACCAGAAATGTTTGAAAGGTGACTTTCTATCAGCTGTTTAAGTTTCA
TTACTTTGCTTATGTGTGAG
TCTCGATTGTCTAAGCCTTAGACTTTTCCTTTCGTCATACTTTCAGTAACGATAATGGAG
TATTTTGATATACAAGTGTC
>CsCML53
TCATCTATGATAGATTCATTTCTCTTTTTCTTGGACTCCTATGGAGTATGGACAATATCCA
TATTTCATTTCCATATAAT
ATGAACCCCTCATTTTAACCAAACACTTAGAATCATTACCACTATCATTTCCTCATTCGG
GATACCAAACGCACCCTTAT
TTGTATTGAATCCCTAGTTAGTAATGACTAAGCGAAAAAAATGAGTGAGCCTTGAAATT
TAGAGATTTATTTTGATTTTG
ACTTTGACTTTGCAACAGAAAAGAAAAGGATATCAAATTACTAAAAATGCAATAAAAC
AATAGTGAAAGGGCAGAAAACA
GTATATATCTTCTGATTCCACAAAAATTCAACACACGATTTATCTCTTTAATTCTTTATGT
GTTCACAAATATCAACAAG
ACACAAAGGTTACACAAACCCCACCACCACGCACACTTGCAATACAATACTACAATAG
TATTACTTAAATCCCAACTGCT
GAGATCGACCTTTGTTGGTGGGTCTTGTTTATTTCTTTATTAATATAAATTCTTGTTTTAA
TCAAACAACAAAAAAACCCT
GATTGATTTTGTTGTTTTCATTTGTTCATACTTTTGGTGAGAACTGCTTACTTTTGCTGA
GCTATGTTTATTAGTCTATT
GATTATCTGAATAGGTTTTTGGTTTTGTTTTTATGGCAGAAATGATGTTAGAGGGTGTTT

TGGATTGCTTATTTGCTTCA
AGATATAAGCTCTTTATAAGGTGATAAGCACTAAAATAAAGCAATCTTAAACACCCTTTT
AGCAGCTAAGTACCAAAATA
AGCAATCTTAACACCCTTTTAGCATATAAGCCCTAAAATAAGCAATCTTAAACATGACC
AACATTTTGAAAAGGACGGAA
GACATCCAGATACCTTATTTGTTAATTTCATAATATATTAATGCCCATTTGTTGTTTGGTTA
ACTAAAATGTTGTTGTTT
TTGTCATTATTGCATAAAAATCTATGCTTTGTTGTTTGAAGATAAAATCTGCAGTAGATG
CTGCTGTTCTTTAAGATGTT
ATTAAAATGATAGTAATTTATGTCAACTAGGATATTTGTTTAGATTTTAGTAAACGTACTG
GTCCTACTAATCAAATGTT
TGCAAATTGCAACTTCAGTTTATATGTGTATTTGCATTTGAACCAATTATGGTGGTTTGA
AGCGACTATAATTAGTTTTA
AGTGATTGTGTAAATTGATTATGATTTTCTGATTTTTATCTTTGAATATGGTAAAAAGTGA
ATTTATGATCCAAGATAGA
CTATATTGTAGTTTCATTTTTGAGGATCTGTGACTAATTGATTAGGTTCTTGCACTTCATA
AACTTTAAGATCCTATCAA
GATTCGGTCTTTTCTAGTGCGGTTTGCATTTAGGAGCATTGGTTAAGAAGGGTGGTATG
TAATTTTGGGTGCGCGAAAAC
CTGACCGATTTAAATAAAAATTGGAGCATGCGGGCTGGTTTGAATGGTTCTGGTTTTGG
ATTGAGTGGACCAAAATCCGT
ATATTTGGTTTGTTTCATCTCCAAAAGTAGAAAATGATCATAATGTTGTTATTTTTTAAGT
TGATTACAACCACTTGAAG
AATCAAAACAAGATAAATTATTCGGGGTCTTAAAGTTAGGGATAATTTGGTGATTTTAC
ATCTGTCCGACTAATCAAGTT
TCGTGTGGCAATGGATTAGCTAGATAGATGTAAAAGTGCCTAACTGTCCCTAAATTTTA
GAACCTTATAAATTGTCTTAT
ATTGGTTCTTCAAGCTTATCCCAGTCTGACCAATCTGATAATTTAAGGGTTGAATGATCA
GAATTGTTAACAAGTTATTA
CCTATACATATCCGTGGATATTCATTCCGCGGAAATGACTCCTTTGACAAAAGTCAAATT
AGGATGATTTGTTGCAGATA
GTTAGCATTTTTGCTGTTATTTCTTGTACTATTATCGGTGCTTCTTTGATGTTTAGCGAAC
TTTGTATTACTAGTTGACA
>CsCML54
CAATGTCCTGGGTCAAGGAGAATCGTCGAATACGCTTAAGTAAGGTGACTGCATCTTTC
TCAAGTTCTCCAAAAGAAGAA
AAGGAGAATGGAAGAAACCCATATCCAATATCCGCACATTTAGCATCGTATTTGACCCG
TTTACGCTGCGCAGCATCACT
GATCGCTCGCCCTGGTATAAAATCAATCAAACCGGATTGCGTCAAAGGCGACGAACCC
GTAAGATCAACACATACGTCGC
GTCCACTATCCCAAGAGTAGAGTAATATGTCATCCGGACGCAAGGATCTGTCCCCATCT
CCAAAAAGCCCAATATCCACC
TCCTTACGAGCAGAAATCCCTGACCGAGAGCATATATCAACAAGAACATCGCGTACAA
GGTTGTGACGATGTTTAATGCC

AACAATACCCGCACATGAAACAGCGTGGTCCCCGTAAATATCTCCAGGAAAAACCTTA
GAGTAGGCAGAACACGGTACCT
GAACAGTGAAAAGAGGAACGCCCAAACGATAACACAATACACATCGATAAGCCCTAG
CGTTCATAGTTTGCCCCAATCCA
GATATCGGAGCCACCCTAAGCCAATCCGAGGTGTGATCCTCCTTTAAAGAGCTCCATAA
CGCCATCTGTCGGGAGGAAAG
ATGGAAAGTAGACTCAACAGATTGGGTAACCCGATCGAAATATATGTCTGCCAATTTCT
TCATAAGTTTGGGGGCAGCGA
CCTCGCTAGGGTTACTCAAAAGGTCAATATCCATATTCACATTAAAAGCACGTAGGGCA
TCATCAAAGGAGGGACCAGCA
GTTACAATACCCGAACTAAGGAGAAGCTTAGACTGTAAACTAGCAGATTGATTATTATT
ATTATTATTATTATTATTATT
TAATTATTTGAAATACAAAAATTGATAGGTTGATATCAAATCTTCATATCAACGTTTGTTT
TTTAACCTTTTCTTATAAA
GCCAGTAACTCAAATGCTAATATCAATTTCCTTTTATTTATTTTTTTATTATTATTATTATTT
TTTTTATTGTTTGGGGT
TCTTTAAAATTCTCCACAACTATTATATATAAATAATATTAGAATTAATTTTCAATTGTGCA
CATTATTACGAATTGCAC
CCAACAAAAGATCTTCCAATATTGCTTTTGCGATAGAAGGTTAGCATATACTCAGTGTAT
TTAAGGTAGCAATTTAAATA
ATCATATTATTTTAATGATGATTTAGGTAATCAAATGTTTAAAACGAAAATTTAAATGATA
AAATTCATTTTTATTCATG
TATTATATACAATAACCAAACAAGAGAATGCTCTATATCACACGCATTATTTATTTGTACC
AAACAGTAGAATCGTTATT
CTTTCTCAAACATGATTCACTTTTTCTGCAATTCCATTCTCTATTACGATTTCATTCTATG
GCATTTTCTCGTACCAAAC
GTGCCCTTAGTAGACTCAACCATTAAACTAATTGACAAAATTTGACCGGACACGTAAG
AAAACAAAAGAAATAACAAATT
TAAAAATTAAGTAGAAAGTATTTTTACCGTTAAAATAAAGTTTAGCAGGTGAAGAAAA
AATAACAAAATTTCGAATACCG
AATTTCTAATATCGAAACGAATTCAAATGTTTTGCTAGCATTAATCTTATCTTTTTTTCCC
TGTTATGGAACAATCTAGA
AATTAATTGTATATTCAATACGCGGGGGACCAACATATCAAGTTGGCAACACCTCTTAA
CTTTTTCTTGCCAAACGTCAC
GTTTTCCTTCCTCCACATTATTACTATTAAAAATATCAAAGCTTCCTAGAAACCACAATA
CGCGAATTATCTGCTGTACC
CATCCTTCTGTCTCCCCGATGGCTTTTACTTCATCTTCCAGGAAAATACCCTTTCTCACC
CACCTTCCTATATTTATATA
CTTTGATCACTTCATTTCCAGTATCATACAAACTCAAATACCCTACAAACGCAAATTATA
ATACTAGCTAGCTAACATCA
>CsCML55
CATTTATATTTAAAAAATTAAATATATTATATCAATAAGTTTTTCTTTAAAAACAGTTGTA
ATGTTCTCTCATTGAGAGA
AAATATATGCCCGTTTGTTCCCACTTAACTTTTAAATGGGCAATATATGCACATTTCAAA

AAAAAATGAAAGGGCTAAAT
GTTACCAAATCTTTTATAAATTTGCCCAAATTAGCCTTTTTTTTTATTTTGAGCACAAATT
GCCACCTCCAATATTTACG
CACACAAGATGTTTGATGAAATTGTAAAACTATATTTTAATGCACCCACAATGCATATTT
AGCTATTTTTATTCAAGTAC
GATTTAAACTATTGATCTTTTGTGTTTGTTAATTTTGTAAAAAAAGTTGCACATTCTGGT
TTCCTTAGGTTTTTAACCAT
AGTTTTGACACATAAAAAATCTGAGTTTGAAAATTTGTTAAATATGAAAGTTATAGTTTT
TTGTCCGTTCTTTACCTAGG
TTTTTTAGTCGTTAAAAAAATGCTTTGTATGACTGAGATATATCAACTTTAATATAACTAT
ATTTAACTATGATGCACCA
ATTTTCTTTGTGCATCACATTTAAGAACTTAGTGCATCATAGTTAAGAACTCATTGCATC
ACAATAAATAATTTAGTGCA
TAATGAAAGATATCCTTAAGTTGCATCCAAACCTCATGAGCGGTTGTCCTATCGTCAATC
ATCATATCTACTAGTTTAGG
AGAGATGGTGGAATACATCGACATAACCACAAGTGAGTCATTAGTGACATGGTGAGTAT
CAATAGTAGAGGTGTTTGTTG
ATGTTGATGTTGATGTTGATGTCGGTGGGGAGTCTAGATGATGATGCACATCACAAGTC
TTGCAAAAACGTTTAAATAAC
GCGCTACACGAATTGCAGTTCAACTTATCTTGATCAAGAGGTATGGGGATACACACCTT
AATACTAGCAACGGAATATGT
TTTATCAATCGGTTGTTGAGGGTTTGATGTAATATCGTCAGTCATGATGCAATAACCGAA
TAGACAATAAAGAGCAAGCA
ACAAAAAGCAAAAAATTGATGTGAAATAAATAAAGAACCGAGAGTTGGAGGGTGCAA
CGTTCGGTTAGGATTTAGGGATA
TGTAACGAGAGAATCGTAAGACTCTGATAGAATATTAGAATTAACTAATCCCCATAAAA
AAACATATTATGCGTCATTGG
AATGCATATATATACGGAATAAGGTCCTCTTAAAGTTCAATTTGAGCTTGATAGATCATG
TTGAATTTTTCGTGAAGAGT
AGGGGCGGAGCCAAAAAATTTTGGTCAGGAGGGCACACTTTGATAAAATTTTGAGACT
ACACTATAAATTAGTACTTGGG
GAGGGGGGGGGGGGGATTACGAAAAAAACCAAATATTTTTGAAAATTTACACTATAA
TTTTTTCGCTAGAGGGGCACCT
GCCCCTTGTGACCCTTTTAATATGGGACATTTATTTTACTTTAATTCAAAAAATTTCTCAC
GAAAACTTCAACATCATCA
TCAAATTCAAAATGAATTTAAGAGGATTTTGATATTATAGACACAAGCACTCAGGTATC
CGAATAAGGCTAGGAAACACA
TGGGCGAAGGCCCGAAAACATACTAACCGACTAACGCTAATAAATACAAATAACAATT
TTGTTTGGGAGAAATTGCATAT
CCGTGATTTGGTACTCGGAACGTACTCAAAACCGTCCAAAAAATGGATTTGTCCCATGC
ACGAAGCATCCTATAACCTTC
CAAAACGAAACCAACAACTTCACACATACACCAACTATGCAACATCCAATCCCCACAC
ACCTTCGACGTACCCGTTAGGC
AATTGCTATCAAATAAACCCGTCATATGCATGCCTACTTTAGCTGTACCCAGTTGTCACT

TAACGACTACCTCTATATAT
AAACCATCCAAACACATTATATGTATCAAACATCAAAACCCCATCTTCAATCTTCAACTT
TTTTATTTATAAAAACATCA
>CsCML56
CTGAAAATGCCTTTTCTAGTTCATCCTTATAAGTCATAGTTGCCATCAACAATGTAGGTT
CGTTGTCTTCTTCTACAATG
TTTGTCTCTTCTTCTTTATTCTTCTCTGGACAATTTCTAGCAATATGTCCAAGGAAGAAT
AAAGAAGAAGAGACAAACAT
TGTAGAGGCTGTTGGTTTTAATGTTTTAAAGACTTCTGTAATTTGCTTCGTAAACATTAT
TATGTACTGTAATGCATAGG
ATAAAAGAACCGTATATTAGTTTGTTAGTATTTGGCAAAAGCCTGGAATTTTAATTGGGC
TTGGGCCAAATGGGTCAAAA
TGGTTTCTCTGTAAACCAAAGGAACACGACAAGTATTTGGATGATTACTACGCGTCAAT
TGATCCAAAGGAAACTTGCAT
GAACATTAAAGGCTTGGAAGATCTCGAATGGAAGGATGGAATGATCCATGATTATTTGG
ATGACGAATTCTTAAGCATGA
ATGGAACTCTTTACATCATGAAGGTTGATTCATTTAAGAAATTTATTTCATTTTTAATCAT
GATAGAAGATGACAATCTA
GTATACAAAAACTGGAATGTTTTAAGCAAACACTTTGAAGAGATGATAAAATGGTTTTA
CCAAGTTTATCTTGGTCGAAT
CATGGTGGATCCCTTGCCCCCCGTCATCAACGGTATAAAGATCGATTTACTTGGTCTTTA
TAAGATAATAGGTAGTCTAG
GAGGCTATCTTGGGGTTACCTTTGGTAACCAATGGGGAGATGTTGCACTTATACACGGA
TTGGAACGAGAACATGATGAG
GAACTAAAACAATGTTACAAAAGAAACATTGACTTAGTACGGTGTTATTTCGAGACTAC
ATTACGAGCCCGAGGGAAGAT
AGCCAAGAAAGAAGGTTATGGGAAGGCCCCAAAGAGTGAAGAAGGGTCGAATGGGC
AGTGGTCACAAGATGGAAATGCAC
AAGTTCTCAACACGTATAGCAACAAGGATACATGGATGGACAAGAAGGATTCACAAGA
AAAGATGGGCGTTTGAAGTCTT
TACACATTAAGGGGGACAATGTTGGTTTTAATGTTTTAAAGACTTCTGTAATTTGCTTTG
TAAACATTATTATGTACTGT
AATGCATAGGATAAAAGAACCGTATATTAGTTTGTTAGTATTTGGCAAAAGCCTGGAAT
TTTAATTGGGCTTGGGCCAAA
TGGGTCAAAATGGTTTCTCTGTAAACCATTGTGTCGGTTAGTTTATATTACGCCTCTTCT
AGGTTTTGTGAAACGTTATA
TCACTTATTTGGTACCGTTTCACTTCATATAAAAGGGAGGCAGTATAGTCGTTGTAATCA
TCAATTCATACTTCAATAAT
ATTACTTCGTTTATACAAAGTTTCTGTGTTATTTACTTTTCTGCAAACTGCATACAAACC
AAACATCCTTTACTGCTTTT
TCTGTATCAACAAGAAACGACAACTATGTCAAGTTTCAAACACAACTCAGTGAGTACT
TGGCCAAGAGGAACACGTTTAC
ATCAATAAATAAGTGAGCATGAACAAACACAAACGAAACCGAATTTTGTGTATATCTTG
TGCTTGTTTGTTTGTTCGTTT

AAAGTTTTAATGAAAACATAAACGAACACGAGCAAACTTATTTCTTAATAAACAAAC
ATGAATACAAATATCCATTTGG
TTAAGCATTTATATTACGACGTTTGTTCGATAGAACTTAAACAATCGAACAAACACCTAT
CATGCTTCGATTTTTAGCGC
GCTACTTCTTGATAACCGGAGCATGGAAGTTTGGTCTTCTCTGTCTCATATTTTATTTTTC
GATCTTAATTGACTCTCAA
TTTCTTAAAGCTTTTTGGTTTCGTGGCCTTCTATTAATCCAATCATTTGAAAGACTAAAA
TTACAGACAAAAAATAAGTA
ACCGAAAAGGCCCCAGTTAAAGCTTCGCTTTGGGCCTCCAAACGGCTTGAGCCGCTC
ATGTACGCAGCTAAAAAGAAATA
>CsCML57
GACTTAATTAATTATATAATGAAAGTTGGTTGGTTAAATCTAGCATAAAGATGCTAAGGG
AAGAAACCATTGCTCGAAAA
AAGCTTATTTAGTAGTTTTACTTTACCAATATTAGCTCATTCAGTTACGATCTTCAAATAC
TACTTATTAATTGTTGGAC
TTGTAGCATCTTGCATTGCTGAGAGCCAGGACACGGGTTCATCCACAACATCATCAAC
AAGAATGATGCCACAAGATTCA
AACGAGTATGTTGTTCTCTTTGAAGTAATTTACAATTAACATATAACATATGGGATCGTAT
TATATCTTGTGCTAACCCT
GTTAATTGCTTCTTCCAACAGTGGCTTCCAGGTTACAGAAGCTTTGAGAGTGCAGATG
GAAGTCCAACGAAGATTGCATG
AGCAGCTAGAGGTTCGTTCATTGACATTGCATGTTTCTTGCAGCTTATTATTATTTTTTCA
TAGTTTTTAATAAGGTGGT
GACTTTGACGTATTCACCTATGAATGGGTATAATTGGGCTGAACGGGTCAAGGCAGGTT
GAAAGTGGCCGAAATTCTATA
TTTAAATTCAGTTAACTACTAAATCGCTTTATTAAAAAAATATAGATTCTTGGTGTTGAAT
ATAATTTTTGTAATAATAT
TCAACGCACAAATTGCTTATATAAATTGATAAGAGAATGTTTCGGGTCAACCCGATCAG
TACAAATTTATCTGTTTTGAC
TCCTATACCTTCTTTTTTCTGATTAACATTTTTGTAGTTTATTGACTGGTATGCTAAATGA
CACAATTACCCATTCACAG
GAGGGGTATATTTGTAAATATGATAGCTCCCTTTTTTCACTCTACATCTTAAGTCACACG
AACAATGAATAAAGCACAGC
ACTTTCTTATTATTCCTTTTTTCAATCTTGAAAACAAGATAATCATTACATCCGTTGAAA
AGAATACCATTTTCTTTTTT
GTGCTGTAAAGTTTCACATGTTTGATCAAAATTCTGTCTTTAAACCTACCAACTATTTTC
AGGTTCAACGTCGGCTCCAG
CTTCGAATAGAAGCACAAGGAAAGTACCTTCAATCAATCTTGGAAAAAGCATGCAAAG
CTCTAAACGATCAAGCAGTTGC
AACCGCTGGTCTAGATGCAGCTAGAGAGGAGCTTTCGGAACTCGCAATCAAGGTAGCA
AATGACTGTCCTCCATCTGTTA
TCCCAATCCCGTCACTTCCTGATGTCGCAGCTTATCTTGAAAACGGAATCGCACCCAGC
ATTGACAGCTGCTTGACATCA
AACAGCAACCCTGTTTCACCCATGGGCATGAAGAAAAGACACCGGGCTATGTTCAGCA

ACGGTGGAGACTCATTGCCACA
AGCAGAATGGATGATGTCCGGATAGTTTGATGCATCTTTCTATCTCAGAAATGTATTCTT
CTTTGAATTCGTTTTGTGGG
TCTGGTTTTGTTCTATTGAATCGATAGTTCTTAACCATCTGTAACCTTGCAAATACTATCT
TTAATCAATTCAAGATGTT
TTACTTTTCATAGTCTATTTCATTTGCTCTTACAGGTGTAATAAATGAAATGGGTACATAC
CATTTTGCTTTTTAATTAT
TTTCTTTGGTTAAATGATGGAAATCCGGAGGTCTTCTAGGAAGCAGTCTCCTAACCCTC
GGGTTTACCTAAGGGTAAGGG
TATGATCTGTCTCCATTATACCTCACCTATACCCTACTCATGCGGGATTGGATATCGTTGT
TGTGGTTAAATGATGGAAT
GTTATTGGTGGGGGCCATTGTTACATCTGCCCATCATTGACCCATCCACCTTCAAGATTA
TGAGCATAATAAGTGGAACA
ATTTTTAAATTCTAAAGTCACAAGAACTCAAGTACTACCATTGTCATTTCACTTTTTGAA
AACCAAATTCCATAAAACTT
TTCTATTTAAGTTTGCAATTTTTTACCATAATACTAAAATTTATCAATTCTTATCTCATCTT
GAAATCATAAAAGCTTCA
>CsCML58
GCCGACATATGTTGGCCTAGCCCTTCAATGGGGATAACCGTCAAGAAATCTTGAGCATG
AGGAGCTCGGAGACATTCAAA
CACCGCTTTAAAGTTTAAACACAAACATACATCATAATACACACACAAAAGTAAAATAA
GAAAAAAAGTCAGTCATTTAG
ATTACCGACTTATTTTTTTTTTTTTTTTTTTTGAAAGGCAAAAGCAAATATATTAAAACT
CAAACCACAGATACAAATA
CATAGAAAATGCAAACCCCTTTCTATAAGATATAGATACCAGGGCGTGAGACAAACCAC
CAAAATCAATGAGCAGTCTCA
CGAGCATGCAAACACACCCCAAAGAGAATCACGAGGGCTAACTAAATTATGACCGAA
CCCAAGAGAGAAACAACAAACAA
AAATACATAAACATAACAGTTCCAGCAGCCAATTAATCACGATCGGTTTAGATTACCGA
CTTATGCTCAAAGCTGGTCCA
AGACACGTGGGTGAGCGAGTATTTTGTTTAGCTATAATCCACTTCCTTGTACTACTACCA
TAAAAATATGTTACTATATA
TAAAACAACTCTAAAAATATATAGTTAGCTTATGTTTGATAGAACTAGCAGTAGTTCTAT
CAAGAAGAAGAAGGAAATCA
CAATAGCTGTTTATTTTATAAAATTATTTATTAGCGTAGATATGTCAGTGATTATAAAATAT
AAAATAACCTAACATTGT
GTTTTAGAGTTCAGTTTCGGAAAGAAAAAAAATGAAAAAAGAAACTAAATAAATTTGT
GTTCTCGAGTTAATTTTTATGA
AAAGAAAAGAAATGGAAGAAAAATTTGGATTAAAACTTTCATTTAAAAAAATGTGAAA
AAATGAGAGAAATTAAAGTTAT
ACAGTTAAGAAAACAAATTAGTTATACTTTTTGAATTTTAACTCTTTTAAATATGGGTAT
AATTGTAAATGGATCAATTT
TTTTTTATTTTTTTTTATTATTATTATTTTTTTAACTGAAAAACACAAAAACTTAGGGTG
CGTTTGGTTGCCCACTATG

AGAATGAGAGTATCACTATGAAAGTGATTAAAGGTGTTTGTTTAAGATGAGAGGGTCC
ACCACTATGATAATGAATGATG
AATATATTCCATACTCAATATACATCAACGAAGATAGGTATGGATATTTCATAGATGAGGG
ACCCACCACTACTATCAAA
CTCATACTCATTGTCATTCATGACAGTATACCAAACGCACCCTTATTATTATTATTATTTC
ATTTAAGAATGCAAAACTT
TAATTAATTTTTGATTTTTTTTCTCTCTTTAAAACTCAAAGCACGATAAAAGTTAAACA
TTAAACATTAAAAAAAAATGA
TGCATATATTTACAATGTAGACAACTACCTACATTTCTTTTAAAAAAAACAAAAAATGC
ATGAAAGATTTACTGCAATAA
CTAAACTGTAATTTAAAATAAATAATTAAAAAAAAAAAACAAAAAGAAAAAAAAAATTG
CCTATCATTTTCTATCATTTTT
CCTAAAATTTAATGGTAACCACCATAGTTGTTAAGATCCCGATCCTGATTTTAGGATTTT
ACGATTTTACGATTCTACAA
TGCGAAAACGATCCGGATCCTATGTAGGATCTTAACATGCTTAAGATCTTATAGGATCCC
GATCTTGCCCATGAGATCTT
AAAATCTAATACGATCCGATCTTACTTTTTCTTAAATCGCAAATTAAAAAACTACAAAGT
TATGCATTATCCTTTAATAT
AATTTGAAAAATAACACGTGATTCTTTTTAATCATTTCATATTCATAATTTGAGTTTGGTT
GGATAAAGTTTCTGAAACA
CCCCCCTTCATTAAATACATCATTACCATAAACAGAAACTAGAAGCAGAAGAAGCTATC
CTCCCAGAACCATCCAATTCC
AAAACCGAGTTTCATCACGCGTAAATTCATCCCTATAAAATCAATCACCCCTCAATCAC
AATCTCAATTATCACAAAATC
>CsCML59
ATTTTATCTGCCTAGCCTCAATTGGCCCATATAATAACTAAATGGGTCGAAATTGTCATCT
CTTGTTCTTGTGTTTTGAC
TTTGTTTGTGTTGCTGATTTGCAGGATGGTCGTATAGATTACAACGAGTTTGTAGCAATG
ATGCATAATGGAAATACTAC
TTTGACAAAGAAAGAAGTGAAGGATAATTTTAGCGTAACCTTGACAGAAGCAGTGCC
AGTTTTTTAATATAACTTTTTTG
ATTGTTTTTTGCATCTAAATGTCAAAAAGATTCATCTAAAACTCTAAACTAACAGATGTT
AATACTCAAATAGTTCATAA
GGTGTTGCATTTTGACTTGTAGATTGTACCTTATCTTTATACACACACATGTGAAATGAA
AAGATAAAAGAGTGTATTTT
GTACATGGACAAAGAGAACAATTTGAATTTGTGATTGTCGGTCCTAATATACCGTTAGG
TCTGGTTCTTGGCATGTATGT
ATAATTGCATAGAGTACAGCTCGTGTGGCGGGTCTATATGGCTGTTTGATGATTTTGGTC
AACGTCTTGCACTCGAGATA
AAATTATCTATGCGCAAAGAACGACGTTCAATGGGACAAAGTTAAAGGTTAGGATCTG
TGGCTTATACTGCCATTGCGAG
TCTGGTGAAATATATACTGCCATTGCGAGTCTGGTGAAATAGCGAACCTGATGTAATATG
TGCCGGACTTGGAACTTCTA
TTTATGCATGGAGTAAACCTATTTGCTTAGACTCGTAGCCCATTTTCATTTGTCCAATTA

ACAAAACTTAATAATGACTT
ACGGTATATCTATGCTATGCTAAGAAAAAGTTGGTATAACTTCTTTTAGCAAAAATATTT
TAGAGCAACAAATAGGTTCG
AACCTAGGACCTTTAGTTCAAGGATTCAAGGCTATAACGAACCCATCTATGGTCTAAAA
TATACTAACCTTTTACAAAAT
ATATTACTTATCTGCTTTTAAATAGAGTTGACCATGTTATTGTCTCTTTGTCTACTTATTTG
AGACGAGAGCAATGAATT
TTATTTAACACAAAAACGTCACTTATGGTGTTGAAGTATGTTTTCGTGAGGTAGTGAGA
TTTCTCCAATATCTTTTGGTC
TTGTTAAGTTGAGCCTTTTGGATATTTGCGTGGATGTAACAAACGCGTTAGCCTCACCT
TGGTTGTGACCCCAGGACCAA
GCTTCGGGGTGAGAGTAAGTAAAGGTTTCACCGAGTGGTGTGTGTAATAAGTGTGTGT
GAGTATATTGAAATTGCTACCT
TTTACAAGTTATTGTATGTGTATTTATAGTGTAAGCTAATCATACCTAGTCGCTGTCTTGA
TCGCTATATCTGACACTGT
CGCTACCGTTGCTTCTGACACTGTCCCTGACTAGCGTCTGACACTGCTTCTTACACCCA
GGAGATAATAAGACGAACTAT
TTCCAGGCTAAATGCCAAATGTGGTGTCAGGTCAACGATCCTTTCGGTTCTTGGACCTT
TCGGTTCTTTAGCCCCTTCGG
TCCTTGGGTTTTCCGGTTATTGGATCGTTCGACCATATACGTCGTCATGTCCCCACAGTA
TCATATTATTTGTCTAAGAC
TAATGATGTGATACTTATTTAGTCGTTACTTTTTTCCTGTGTCTTTCGAGTGCTCGTTGTT
TCGTTCGTCTTATTGGGCC
ACGCGATGGTTAATAGGCCCAAGCCCATTGGGTTAAATAGGTTAAAACCTAAATTACCG
AGGTTCGTGGGATCGTGCCAC
GTGGCGTCACGTGCTCTTCACAACGGTTCAGATAGGGCGTGTGGATTCTGACACGATG
CGACGTTTGAGTACCTCCCAAT
TGCCTATATAAGTCGTTTTTATTTCCTTTTTACTATTTTTACTTTTTACTCCCATATTCCTTG
TCTGTCGCTACCAGGTC
TTCCGGCCTGCTACTCAGGTGCGTTTATCTTTCTCCTGTTTGTTCGTCATTCGTGTTACC
TTCCCCTCAGTTTCCATTTG
>CsCML60
CAATTTCATAATATATGTCTACATTCTAACCATTGTTTTGTAGATTTTCAAAATTCACTTT
GAGTACTTGAAGCTCTCAA
TAAGGGGGAGCTTTCGTCAAAGCAAAAGCATTGCAAAACTTTCTTCTTGGCCTTCTCA
AAATACTTGACGCCCTCACCAA
GGGGGAGCAAAATATGCACTACTTTACCCTTCTTATTTCTTTCTCATCTCTTTATAATCTA
TTTCTAATCTCTTTCTTCT
CTTTCTTATTTGAGTTAGTTTCTTTGCGACGCTCTTTTTGTTTCGACAAAAAGGGGGAG
AAAAGAGTAAAAAAAAAACTT
AAATTCAAAATTTATGAATTCTAATTTTTACTTAAAATAATGTTATGTTTTGTCATCAACA
AAAAGGGGGAGATTGTTAA
TACAAATAAACCGTAAAAATGTTTATTTATATTTTACGATCTTCTTATGTTTCGTCGATAA
CAACAATAAAATTATTTTA

ACGAGATAAATAAAATACGTAAGTTTTTGAGCCAATAGTTTACTAACTCATTTCACTAAA
CAAATAAAATCATGTTTAGT
TAATAGTTTATATAAAACCAAAACCATTTTTATGAAACACATATAAGTAATATTTCTTACT
TACATTTTCATAAAATACA
TACTTTAGAACTAACTAGTTAAATGTAATATGTTAGTAAATAGAGTTGGACTATCCATAA
CCCAAAACTCATAACCCATA
ACCCATGACCCATGCTCATTACATCATGCATGACATCACAATGGGTAAAAACCATGACC
CGTGATTCAAATTCCATGACC
CAAAGTCCATAACCCATGATCCAAGATTTCCATTGCATCATGAATAACATCACATGACCC
ATAACCCAAAACCCGAAATT
GTAACCCAAAATCCGGACTGGACTGACCCAGGTGTCGCATCCCTTGGATTGCACTGGC
CGGATCCCTCTGGGAATGGATC
TGGCGAATTCGGTTGATCAGACAATTATTTCGTTATAAACAGTGAGTCATTTCGTGAATT
ATTCTTACCCTTTTTAGATA
TATATTCTTACCTCACTAACATAAGATCTTTGGATCATCCATGTAGCTTTTCTCTTAGTGA
TTCTCTAGGTTTCTCTTAA
AGGCGAGATCACAACGATTAGCATAGAAGACTTGCAAGCTCGAAAATATGGGTTACTA
GACTTCACCATCATAAGATCAC
ATAAGTGTCTCTTGTCGTTGATTCCTCTCAAGTATGTCATAGGGGTGTTATGTTAACTAG
TGGTGCTTGGATTAGTTCCT
CCATTGTAAAGGAACTCGGTATCTAGCCTTGTACCAGATAAAGTGTAGGTGACGTAGGT
TTGGTTATACCTTGTGAAAGA
ACCCGATATCTAGCCTTGTACCTGATAAAGTGTAGGCGGCGTAGGTTTGGTTATACCTTG
TGGAAGAACCCCGTATTTA
CCGGATAAATTGTAAGGGACGTTATGAAGGCTTCTCCGCCAAGATAAGGAGCTTGTGG
TGTAGTGTTTACGAAATCTCGA
GTTGGACACTCAGGGAGTGGAGTAGGGCAAGGAAGGTGACCTAGTAACCGAATCACT
ATAAATACTTGTGTCTCACGGTT
TTATCTTTCTATACCTTACTTCATACTCAATATTCTCATATATTTAACTATCAATATCCATAC
TTCAAAATGAATTGACA
TAAATAAATCTAAATGTGTTAAAACGGACTTTTAATCGTTAAAACTATTCAACCCTTCCT
CCCTTTAGTTTTCTACACCC
TGGTCAGAAACTATTCAAACGGCACCTCATGTGTGCATTTATAAATGGGTCTTTTATGTT
GGGATGAAAGAAGTAAAATT
TAAACAGGATATATATGAAATGTGTATGTATTTAAAGGGATGTATATGCAAATATTGGACC
TAAATTCCTCAAAGATAAA
AGCAGATGATTCCTTTCTTCCATTCTGACAACGCGCTTTGTTAACCCAAAAAGAAAAG
AAAAACCCTAATCAAAAAAAGA
>CsCaM3
GTGTCTGGAACATGGATACAGGTGCGAGCTCACATCTGAATGCATCTGTTAATAGTCTT
AGTAACATTATTAATACTTGC
ATGTATCCATCTGTCTCGGTTGGTGACGGGCATAACATTCCGGTCACCAATACGGGTCG
TAGCATTTTGCCTACTCCTTC
TCGACCCCTTTACTTAAATAACGTTCTTATTACCCCTCATATTGTCAAAAATCTCATATAT

GTCCGTCAATTTGTCTGTG
ACAATAATTGCACTGTTGAATTTGATCCTTTTGGTTTTTCCGTCAAGGATTTCATGACAC
GTCGGGTGCTCCTCCGATGT
GACAGCACGGGCGACCTTTACCCTGTCACAGCCCCATGTCCTATTCCCCAAGCTCTTCT
TGTAAGTCAACATACGTGGCA
TCAGCGCCTTGGACATCCAGGGAGCAAAGTGCTACGTCATCTCGTCTCAAATAATTTTA
TATCTTGTAATACAGAGAAGC
CGCCTGTGCTTTGTCATGCCTGCCAGCTTGGCAAACACGTTCGACTTCCATTTGATAGT
TCTGATACTATTGTTAATGCA
TGTTTTGATGTTATTCATTCTGATGTTTGGACTTCACCAATTTCGAGCCTCTCGGGTTTT
AAATATTATGTTTTGTTTTT
GGATCACTATTCTCATTTTGTTTGGGTCTACCCGTTGATGAACAAATATGATGTATTATCA
AAATTTGTTCTATTTCGCA
AATACGTTCACACTCAATTTAAGAGTGAAATCAAATCGTTTCAGTGTGACCATGGCGGT
GAATTTGATAACCGCACCCTA
CATAAACTTTTTGCCGACAATGGCATTCAGTTTCGTTTCTCATGCCCCAAGACATCTCA
ACAAAATGGGAAGTCAGAACG
GATGATTCGCACAATCAACAACCTCATTCGAACCCTACTTTTCCAGGCCAACCTTCCAC
CAACCTTTTGGGTTGAAGCTT
TAAACATGGCTGTCCATTTAATCAACTTCTTACCTTCTACAGCTATTGCCAACGAAATCC
CATATACCCGACTGTTTGGC
CAAAACCCGGATTATAGCCTTATACGCACATTTGGTTGCTTATGTTACCCCCACTTATATC
CTAATCACAAACTTGAACC
ACGCGCCCAACAGTCCAAAGATCTCTGAGTAAAGATGAAGTTCAATATTGCTAATCCA
ACCACTGGGTGCCAAAAGAAGC
TTGAGATTGACGATGATCAGAAACTTCGGGCTTTCTATGACAAGAGGATCTCGCAGGA
AGTCAGTAGAGATGCCTTGGGA
GAGGAATTCGAAGATTATGTCAAATATGATTGCTTGAGAGATACAAATTACAATGATAC
AGATATATAGATGTGCATAGA
ATCATCTAGAAAACGGGCTAGAGCCCATTACAACCTGCTTACTTATTTGAATATTCTGAC
CCGGGTACTGAACCGAATAA
CATGTTGACCCCCCTGGCTGAACCGGGTAGTCATGCTGACCCGTTAAGTTGCACTGTTG
AACCATAGGACCAAGTGACCC
CCCTTGCTGAGTCGAGTAGCCAATTGGACCTGTCAAACCTCCTTGTTGAACATGGTAA
GAATGCGGCAGTCCAAAAAGAC
CAGGTGAACCCCCTGGAGATACAAACATACTGTGTGCCTAATATTAATTGATTGAATCC
CAAAATCACAATTATTGATTC
AATTATTCATTCCTATTCCTTCATCTTCATTCTTTCTTAACACAATCCACAAAAACTCAA
ATCACACTCTTCACATAACT
TCACATTCAACCCTTCACAAAACTTATAAAATTTTTAATTCTTCACAGAATAACTTTATA
AAATTTAATTTTATATAAAA
CCAAAATGAATTTCTTCAAATCAACCTTATTCGACGAAGACCCTCCTCAACCAGAAAC
CACAAACGCACCTTCCTCCCCT
CCACGCGCCGGCGCGTACACCGATGATCAGATCTCTGAATTCAAAGAAGCTTTTAGCC

TATTAAGGAACTCGGAACAGTT
>CsCML61
AAGCTCAAAACCTGTTTCCAACCACAACTGGATATGATATCACATTAGCATCCCAAACA
CTGTTACCTTTTATAACAAAA
ACTTAAAAATTTCTGTGATCATTGCTAAACATATAATAGGTTGGTTAGCTTATGATAGTG
AAGTACTCCTATTGCATATT
GTAGTGCGTCATGACCCTATCAGAAGTTGTTATAAGAAAACAGCATGGTCATTGAAATA
TGGTTATTATTAGCATTGTCT
TTGTTCTAGATTTATCCCACATCGGTACATGTTTATAGTAAAACTAACTTTATAACTGAA
ATACGATTTTAACTAGAAAA
TGAGTATCGATGGATGTGTTTATAATAAACTCGTCTATAAATGCTTTGTTTTTTTAACTTG
CTAATTTCGAGTTTATAAT
AAACTCATGATGCATCACTATTTTAATTTGATGATTTTTTTGTTTTGTTACAGGTACTTCG
ACCACCATCGTGTTTGGCT
AATAATTGATCGAAAAATCAATTTTATACCTTTTTTACTTTGATACTTTTAGTACTCAGCA
AGTATTTCGAAACGTTGTG
TTCAATTAACTTTGGATGAAAAATTAGTTCGGGCATAAAACTCTTAACTATCAAAAAGG
TATATCTTAAGGCATCGACTA
TAATAATTTACGAAAAGATTTTGAAAAAGATACGTAAAGGTATAAAAAAATTTAAACTA
TAATGAGCATTTATTTATTGC
TTGTATATATTCACTTCACGCTTGTGTGACATTGAATAGATCTAGTATATTAAATCTCTTA
ATACTTATTGCATTTGAAT
TATGGGTGATGTTTTTTTTTTTTTTTTTTTTTTTTTTTATAAAAGGGTTAATTCACCCCGG
TAAAATAAATTATGGGTGA
TGTTTATAAAGCTTTAAAAGGTTTATACATAATTAAGATAGATTCATAGAACATGAAGTT
TATAAATTGATAATATGATA
TAACTATAAATCTTTAAAAAGGTTTACCAATCACCAGGGCCGTCTTTGATGGCGTGCAA
GCTGAGCGACCGCTCAGGGCC
CAGAATTTTTTGGGCCCTAAAGTTTGTCCCATTAATAATTATATGCATGTGTAGACATATT
TTTCAAGAAGAAAAAAAAA
TATTTAGACGGGAAAATATCCTATATGAGTAATATGATATAACGTTTAGATAACCGATTGC
ATTATATCTTATAAACGGT
GATACTTTACATATAATATTGTTTAACGAAAGGCCTCTTTATATAATTTCGTACAGGGTCC
TCAAAAATTTCGCCGATTC
ATGAGATGGCCCTGCCAATTACCACACTGGATAACCTTAGAGGCGGAAAGCTTAAGGG
AATTGATACTTACACCACATCT
TTTGATGCATGCACCACAATTTGTGCATTACTGTACCGTACAATACTGTAATGCCCAAAT
TATGGTGCATGCATCAAAAG
ATGTTGTGTGAGTAACCTCCAAAGCTTAACTCACGACACACCGATAAAGAGTCCACTA
AGACTCCACGTCCATTACCTTG
TATCTGTAGTATTGCATACCTTGTATTTTATCTACTTATTTTTATCATAAGTAGATTAATTTA
TCTTCTAAACTAAAACA
TGAATATAATCTTCTAAGTTCTAACACATTCTTTTAGTCATAAGACACGCTTTTAGAAAT
ACAGGTAGCAAAAGAAAGGG

AAAAAAAAAAAACATGAATAAACGAGAGTTGTAACTGTCACAATCAAAACTTACGTGA
CCAATTTATTATATGTAACGAAC
TTTCCTAGCAGTTTCATTATTCGCTATCAATTCCATGAAGAAACTGCTTGTAAGCTTCAT
AATCAGTTCCACCACGTTAC
ATTTCAAAACCAAACCAAATAAATTCAGTATTAGTGCCAAACCACGCGTAAAATCCATA
CCTATATAAGTACTTAAACAT
CAATCCATAATGTTATCATACATATAGTTCATAACTCAGCTCATAATATAACCATCAAGCA
CTCAAGAAATTTTAAAAGC
>CsCML62
TCATTTTTATTGAAAAATCAATTGGGCAAATAGGAGAACTCAATTTCGCCACGTAAGCA
AAATTGTGTGATGTGATTGGT
TAATAAAAGTCAAAATTAAATTGATTTTGGTCAAACTTTAATAAATTTGGTGGAGAAAT
TTGGTTGATAAAAAAAAACACA
TAAAAATATAGGTGTCAAATTTGTGAAAATAAAAACTTAAAAAAAGAGAAGGTAATTA
ATAAAACTTTAGTGGTTGAATT
TCTAAAAATTATATAAAGTTAAGTTTAATATTATAAGCCTCCATATTTTTTAATAATTTTGG
ATTATAAACAAATACCCC
CTTATACTTTATACCCTTTATACTTTATATCTTAAAAAATACAATGTGTATGTTGTTTTAAA
ATAACAACATATAAATTT
GCATAAAATTTATAAACAAATAACTTTGGATTATAAAAAAATAACAACATATAAATTTGC
ATAAAATTATAAGTATCAAT
TTTAAACTATATACGTTCAATAAGGACTTATTTATTTTATTTTTAAAAGTTTAATCTATGTG
GACATGAGTTTACATTTT
AAATATGACTAAATTACTGAAAAAGGGAATATCATTATCAAATTGTAAGCACTGGTTACT
TTAATATAAAAATAAATATG
CGCAATTGTACTTATAACAATAATTCATGTAATTCTTATTTATTTATATAAGGATATATAGA
AAAACATTTTAAAAAAAC
TTTAAGGAGTATACTTTAACTTTATCAACGCTCATTATGTGATATTAAACGTGCATCATGT
GTACAACATAATATCAGTA
GTGAAATTTAAATAACAACATCAACATCTTTGTCAGCTAAATAAAATTAAATCTTGAGTT
ACCTAACATAAATTTTTACA
AATATTTTTTTCAAATTCTAATGGATATTAATGCATAATGAAAAACTCGTGCATAAGTATT
AGTTGTACTTATATCATAA
TTTTCATGGGCAAATTCTTATATGTGTATATACTGAACTAAAATGTTAAAATAAATTTGAT
TTATATGTAAACTCTGTGA
GTGCACGAAACTTATAATTATATAATTATATGCATTACCTTTAAATTCACAATATTCTGTAT
TAATACAGAATAATTTTT
TTTGTCAAAATCCCGTGCGTGCACGGATATTATAACTAGTTTATGAAAATGGTTATTGGT
ACATCCTTCTAAAATGATTG
TAATACTTCCCTTCCCTTTAACTTTTTTTTTTTTTTTTGCTTTTATTATAAAACTACGAAA
CGGAAATAACGAAACAGAA
AATATAAAGTTTGACAAGTCAAAAGTAGTAAAATACAAAACATACGGAGCAGAAAATC
ATAAAAAAAAATGTCTTGTCTTG
TTAAAAAGTGGGTAAAAGTAAAAAAAATGTAAAATGTATAAATATAAAGTTTGACAAAT

CAAAAGCAGTAAAAGACAAAA
AACATAAACAAAAAATCATAAAAAAAAAAAAAAAAAAAAAAAAAAGAATAAAATGAGTAA
AGTAATTCACGAGCTAAAGTAAA
AATCATAAAACGGTTATGTTTATAGTGATTTTATTATACTTCGTAAACCAATTCACGAGCT
AAAGTAATGTGTGCATGCT
TTTGTTGTTTAGGTTTCACTCAACTTCATGGAAAATTCAGAGTGTTTATGGTGATGTCGC
CCAAGTCAACCAGTCAACGC
TGTGTTAACTCACCATCCTAATTAAAACAACCTTTTAACAAGTCTTAAGACTACCAATTT
CAAGATTCTAAATGTCGTTG
TCAGCATTTTCCTGCAATCTTTCTAAGAAATGTTCTTGTAAACTGGACAAAATACAATG
CTATAGCACATTCTTAGACAA
TATATAAACCTTACATCCCATGCCATCAAACATCCAACATTTGCACCCCATCCGTGTTTT
GTACAATAAACGTAGGAAAC
TCATTTGATCAAGAATGGTGACACCATTGATAAAATTTTCATCTTCCATCCTTTTACACT
TCGAAATTGCTTTTTGTAAA
>CsCML63
TTGTGACGGGTCAGAACTCAAAACTCGTGTTTTCAAGAGACGGCGTCTAGTTGTGAAA
GAGTAGGCCGTCGCTACTCAGA
CGGCCTAGATGCGATCCAGACGACGTCAGAAGCTGAAGAGGACGGCGTCACGGACAT
AAGGTGACGCCGTCGCAAGGTTA
ACGTGGCCAAGACGAAACCCTAATGGCGGCGACACGACGGCGTCACTAAACATAAGG
TGACGCCGTCGCAATTGTAATCT
TATATATAGAATCCGAATTTTTCAGTCTTTGTGTGGAGAGTCTTTGGAGTAAAAAACCTA
AAACCGAACCTAAGGTGTCT
TGTACAGTATTTTAGGGTTTTTAACCCTATCTTACACCAAGATAAGTCTTTGCTTCATTA
ATTCAATTATGTCTTTGATT
ATTTGTGTGTATGCTTGCATGTTCTCCATGTCCGGCTAATTTATTTGTGTGTTTGCTTAGG
AAGTATGAAACTTAATTAT
TGAATATTGATTGGTTGTTTCTTATCTTAGTTGATTAATGATCTAGTAATGCGATAGGTCA
TTCGTTGTGTACTTAATGC
TTGATTCATCTTTGTGAACCTTAGAGTATGATTACCTGAGTGATTGTAGGCTAAACCATC
CGAAGAGCGCCTTAGAGTAG
GAGTCTATAATCTCTAAGTGGATTTGTAGACAAGACCCTTTGGTAAGTTCAAAGAGTTA
GGGAGAAACGCATACCCTACA
ACTTTAGAAACACAACGCAACTAGGAATAAGACGTGACACTCAACCATCAATAGGAGA
TAATTAAGGAGTAATACAAACT
AAGTAAACACATCTTTAAACTATCATACAAGCACACATTAATTAGACATAGACTCCCAC
AACCTTAGTTTAGATAATTAG
ATAATAGTAGAGTAACCTGTAGAAATACCAACCCCCCAAATCCAATACCCGAGATTCGT
ACCTTTCCATTTTCATAAAAT
ATAAAAAGATAAAAAAATAATCTTCCTCAACCTTAACCTTAGCGTAGTAACTCCCTACT
AGCATTCCTATTCCCCTAGAG
AACGATATCAATACCCGTGGCTTACCCTAATTACTACACTACAACGATTAGGTTCACTGC
CTAGGGTGTTATAAAACTTG

CTCGAAGAAAAGGAAGTTTCCTTGACCCCGAACGACGCGTGTAGCGTTATCAACGTGC
AAAAACGGACAAAAAGGTGCAA
AAATTGACGAAAACGTGCCAAACAGACGAAAACTTGCAAAACAGACGAAAACGTGA
AAAAATAGACGAAATGTGCAAAAA
CGGACGAAAACGTGCAAAAACAAACAAAAACGTGTAAACATGGACGAAAACAGTAA
AACCGGTTTTTATACGTGCAAAAA
CATGTAAAACCGCATGAAAACATGCAAAAAAAGACGAAAACGTGCAAAAAACAGAA
GAAAACGTGCAAAAACAGATGAGA
ATGTGTAAAAACGGATGAAAACGTGCAAAAACGGACTAAAACTTGTGAAAACAGACG
AAAACGTTCAAAAACGTGTAAAA
ACGGTTTTTATTCGTGCAAAAACGGACGAAAACGTGTAAAAACCAACGAAAACATGT
AAAAAGCGACAAAAACGTAAGAA
ACTCAAAATTTAAGTTATTTGCTAACGGCCGTTAGTATAAAGGACTAACAATGCAAGTT
TATTTTAGTCATATGGGTAAA
GTCAGATCAAAATTTGAAAGTACGTTGGTGAAGTAAAGTAATAAAAAAGTAAATATTTC
AACTCGGCAAATTAAAGAAAG
TAGGTTGCTATTTTATGACATTTACCTTAAAGTTTATAGTGATCCCACTTTTATTAAAGTG
TTTGATTTTTATTACCTAA
CAATAATTATTGGGATATATCCTTCATGTATACAAATACAAATTCGGTATCATATCGAACG
TATCAGTATAACCTATATA
ATCAGTTAAAAATTCTCCCATATGTACGGATGTTAAACCACACGCACAATCAATATTCAC
AAACACACGAAACATCGAAC
>CsCML64
AAAGTCAAACTTTCCCTGAATCACTAACTACCAGGCTCAGTGTCATCACTTACTAGAAG
AAAGTAGCCTTGTCACTTACT
CTAAATAGCTAATATAAACAGAGACAAAATACATCAGTATTTTGTCTTCTGTAACCTGTC
AGTTTGTCCTCTAGGATAGA
GTTAAAAAACCATACTTGTATAAATAGCATAAGAGTTGGAGTGGAATATTTTTAAGCTTG
TGCATTAATTGTAAAACACA
CACACAAGCCATCTTTCACTCCTCCAAATACAACTCTTTCCCTGAGATAGCTCAGTGGT
ACCTTTGTAGTTGTGCTCAGA
CTTAGCCTGAGCTTAGTGAGGAGTCAAACCATACTTGTCGTTTCAATCCCAAGTACTAT
TCCGCAAGTACTTGGGTAATG
TATTCTTTCTGATTCATTTCTAATAAAGAATATAAACGTTGAAAATTAATCAGTGTGTTTT
TACTTGCATTATTTCAGTC
ATTATTCCCAATTAGTTATTATAAATCATTTTATATGATTGTGAAACGGGAACAACAGAG
ATAAAGATAGCCACTCTGTC
TCAAACCCCGCCACATTGTCATTCTATTTACAGTCCTAGTGTTTGGTAGTGACCAGTAC
GATTAGCCTCACAATTTTTGT
CCTTTTCTCATAAGTTTGCTGATCTTGTCACTAACAAATTAGTTACAAATGTCTATGAAA
GAACACCCTTTTCATGTTTT
TGGTTCGCCAACCCCAAACTCAGCGCAGCAATTATTTAAGTTCATCATGTACTTTGGAT
GCAAGAGGCCGGGTTATCTGT
TATTTCTGTTTTTGCTTCTCCTTCATCTTGTGCATTAGAATTGGTATGCATGTCACCATCT

TGTAAAGGACAATTTAGAT
AACTTGAATGAAGAACAACTAAACAGAAACAAAATTGATAATCATATTGTTTATTGAAT
ACTAAGGGTGTGTTTGTTTCA
TTAAGACGTTTTGGGTCTGAATCTGACTACCACATCTTACTAGTAAGAGATGGCTAGAA
AGTCTAAATCTGAACGAAATA
ATGTTGTTTGTTTTTGTATAATTGTCCGAATGATTTTAAGTGCTCGTTTTTGCAATTTGTT
CATTAAACCAACGCAATGC
GATAATAAGTAACTTATGTAAATATGAAACAAGACTTTTTATCATAAATATAACATAGTTA
ATAAACAATCAAATTTATT
ATATAAAGTTTACAAAATGAGTAAGAGGTAGTAAGACAAGTCCCCCACCTCTTACTCAG
TAAGATGTTATATGTCTAACT
GGGTCTTACTCAAAAGAAAAAAACAAACAAGCAAAACGTCTGCCCTCCTTGTACTGG
TAAGAGTAAGAGGGAGTAAGAGG
GCCAGTAAAATTTTTTTTTTAAACCAACACACCCTAAGTTAAATTATGACCAAGACTA
AACCAATGATATTTGCATATG
AAAGTTTGTTGGTCCAAGTAATTCTTCAACCACCATAATCATCTATCTTTTGACAACTTA
ACCAAAAAATGAAAGAGACAT
GATAGAAATAACTAAAGCCAAAGTATCATTAAAAACAGATATTTTTAAACACAGGTGTT
ATATTTTAAGTAATTGTCAAT
GTAATACAAAATTACTTTACAAATCACCTATTCCATTACTTCTAGATTACGTTAATATGTG
TAATCGAATGAAGACAAAA
TGTAAATAAACAATATACTAGTGTAGACGTGTAGTATTAAACAAAAGATAAGAAGTGAA
CCACGTTTGCAGAAATAAACG
CGGCAATGGTTTGGGTCGGCACACAGCTTAGGTTCCCCTTCTCTGCGGCTACCTCTTTC
TATATTTATCCAACACATAAT
CATACAGATTACGTACTTGTTTCTATAATAACCCAATATAAATACATCTAACCCCAAAAC
ATTTTATCACAATTATTTCA
AAACACAAGCATTTTCTAAAGCGAGCACTCGAAACTTGAAGTTTTATAATTTAGGTATT
TCATCAAACACTCTGCAAGAT
>CsCML65
ACACCCACTTTCTGAAATTTACAGAAAACATTTTAGACTTGAGATAAAATAAAAAAGTC
GTAACTTATTTTTGGGATGAT
TTGTATAACGCAAATTAGGGTTTATGAATCATTGTAGGAATCAAAGCTATAACCCCAAA
ATTAATTGTCATTTACAACAC
ATAAATCAAAACCCTAAAATTAAAACTAAATGGATACTTACCAAACACTATACAAATCA
CCGGCACAGCGATGTAACAAA
GATTTTTTTTCTTGATGTAACCAATGTTTTTTTATCTGGAACGATGTTGGTGTTTGGTCC
ATGTTTTGGTTATTCTTCA
CCTGCATCAACCATTAACAAATAAAAAAAATATTAATTAATTATCCATTCTCAAATCACA
ACAAACAGGAGAAGTAATAA
AGCAAAACAATTGAAAATTTGAAACTGAAAGAAGGAGCGTACAAATCATTAAATTTAT
ATGAATCTACAATTGATTTTAT
TTGATTTATGGAACGATTGTTTTGATAAGGAGGTACCGATTGTTTTGATATATGGAGCGT
GTAGAGAGGGATCGTATAAA

ATTGGTGCAGCAGAACTTGTACTATGAAGCTAAATAAGGCAGGGCATTAAACGAATTTT
TAATGCAGGGTGCTTAGTAGG
CTTGTACAAAAGCTTATTTATTATATATAAATAGATGTCTAGTTTGAATTTTCTAAGTCT
TTCTTTCTTAACTTTAACA
ATAAATAATTTTGGTTGTGTTATATAGTATTTGATGCAAAGTATATGAATAGATTGAATTT
TAAATGTACTTTCACTACG
TACTATTTTTATCTAATACTAATAACACAAGCAAAAATATTTAAGGTCAAGGTTGTAAAA
GAAAGACTTGAAAAGTCAAA
CCTGGACTAATAATATGGGACGGAGGGAGTATATAATTATTTCATATTTGTGGCGCTTT
ATGTCACGCCTCAAAAACG
CAAAAAATTCGCTTTGATCGTCAAAACATGAGGCTTAGTCATGCCGTCATATCATGCCT
CGCGCTATTTATAACCTTGTT
ATTTACACATCTTCCTATGACGGAATGAGCCAAAAATAAAACATGGGACGAACAAATAT
ATGTTACATTAACATTTTTTT
TTTTTTTGAACTTGTTACATTAACATTTAGAGAAAATACGAGTGTAGAAACCCGCGCGA
TGCAGCGGGATTGGCTTCAAA
GGACTTACTTTGTAACGACACTTAAACCTTAGGGACTATTTTTGCCAAATTGCTATAGAT
AGCAATTAATGCTACAGGGC
TTACTTTGCAACTACACTTAAACCTCAGGGACTGTTTTTGCCAAGTTGTGAAACTTTGC
ATTTAATGTTTGGTTAGGACT
TACCTTGCAACGTCGCTTAAACCTTAGGGACTATTTTTGCCAGATTGCTATAGATTGTAA
TTAATGATCTAAGACTTATT
TTGCAGCGCCATTTGAACCTCAGGGACTATTTTTGCCAAGTTGGAAAAGTTTGCAATTA
ATGCTTGCATCACACAACTTG
GGTCCTGCCTTTTAGTTAATATATATAATAAAATACAAGCTTAGTTATAAATATACGAATT
AGATTAGTAAATAAAGAGT
TTTGTACAATAAACTAGTTAAATCATAGGGTTCTTTGCTTAGCCGAGTAAAACAAATATG
AGGTGATCATTTAATAATAA
TTACACGATGATTATTTATTTCAACCATGTGCAACCGAAAGCGTGTTGTATATTTCCATG
TGGACTCGGTTTCATAAATA
TTTTTGCCAATTTTCTAATGTTATAATGTGACAATAATGAGTTTTGATCAAAATGTACAA
ATCACAAAGCATATCGTACA
ATCTTTTTATACATGCAACGATTGAAAACCAATGTAAAAAGGTATGTATAAACTGCCCCT
TTTCTAATACCCTTAACCTT
AGCCACTTGTCTTCTTCTTTTATTATATAGCTTCAATCTCCATTCTCTAAACACTAACAAG
AAAAGCAAAATTAACAAAC
>CsCML66
AATACTGTCACAAGCTTTTTGAGACATACGTCTTATAGGCAAAGGTGGATAGCTATTTTT
TCATTACTATATATATGCTT
TATAAAAACAATATAAGATATGAAATTTCCCCATGAAGTTGAAAATAATATTAAAAGCAC
GTATTTTTCTTTCACTGTTT
TAGCCGTTGGGCCACTTTTCCGGCCCTAACTTGTATCATCCTAAAACTGCTTCATATAAG
CAGATAAAAGTGCTAAATTT
TAGACAAATTAAGCGGCTTAAAAATACCAAGTATCAACCATGGACCATCTAAATGTGAA

AAGATCAGATGTTAAAAGATC
AGGAGCAGAGTATATAACTAATCAAACGGGTTAAACAAATAAAATTAAGTTAACATGTG
AAAATATCATATGTGTCGCGC
AGTTTGAAAGTCACAAAGGTCTCTTTCTACACAATGGTTACTTTTACAGGATGTAAGAC
ATGGACATGATAATGATAGGG
GTCAACCCAGCCCAATATGCTCATCTTTATCACCCCTAATTGTTCTACTGAAATTGCTTA
TAAAACGTGTAATGCATGAG
AAGGTTTACTTACAGTGGGCGCTTTGGTCTTCTATATACTCTCTTGAGTGTTTCAACATG
TACTTTTTCTGAAGTTGGTG
ACAGTTTATATGAACCTGCATCAGAATCATGTTTTGCATCAGCATAGCCAAAATATAAAA
AGAAATTATACCTGTTACGT
TTCAGCAATCATTAGCCTGCAACACATTAGTATAGACACGGAGCAGAACTAAAACTTAA
GGCAATGGATTTTGAAAGATG
ATACCAACAGGAAAGCGATAACAAATATCATACACATCAGACTTCGTTAACGGATTCCC
TCCTCTTGTCTATGGTCAATG
CAAATACTATCTTCTATCGCAGTGTCTCACAAGCTGAGCAGTTATAAATAGTTCATGTGT
TGGTTATCTACGGTCTACCA
AATCATAATGTAGTTCCAACTCAATTATTCAATAAAAATACAAAATTGACACCCAAATAA
AAATAATGAGATCAATAGAC
CCACAAGCCTTAACAAAAATAGTTGAAATCTCTTTGTACTTATAAAACCTTAAATAAAA
GATTATCAACTCCAACGCTAT
AACTAAAAGGTAATAATGTACTTATAAAAAGAAAATTATGATGTACAAAGGGTTACCAG
TGTCCATGGCTGCTAAAATTT
CATTCATGAGACCAATTATCTTGTCAGTCCGCTGCAGTCCTTCTTTGGACATTTTTTGCA
ACTAAACGCACCTTAAACCT
CTGATTAAACAAATAGCTAATACCACCATCATCTGAGAGGTTCAAGTAGGAAAACTAAG
AAGGTGTATAGTTGAACCATA
ATACCATATACAAATGCGCGACCGATATAAATACATTAGCATCATCAGTACTTGAATTAA
AACAATTCAAGTGCTACATG
TCTTTTAAAACATTAATTTGGCATTCCGAGTAATAACTTACCTGATACATAATCCAGAGC
TAATAAGTCATAATCCGAAA
CCAATCCCACCTGCAGTAAGAGCAAAGAATGAACCTCTGAAGACTTCTTAAGAGAATT
TACAGGTATGGATACGCAATCA
CTCAAATAGACACGGACAATAGCTCCGGAAAAGTAAACAGCAGAAAAATAAATCAAC
AAACCATAGGTCATTAGCAGCAA
ACCATAGACATATATATAAAAGTGTACGAAATAAAAAGTATGGAGATTCATGGTACCCC
GTTTGGTTCAGAATTGCGAGT
GCGGGACGGGATATAAGGGTATCCAACCTGCCATGGCTGCAAACACAACGTCTGCTAC
TCCGTCAAGATACGGGTTGATT
TAACCCATCACGATTCAGCCCCGTAGTCCGAGTACTTGAATTCAATTTTTTCCGAACCT
ATTCATAAAACACCATTTACA
AAACCGACTAATATAATACAGCATATGCAATAAGATTGTACAGCTCTTACCATTGGCCTA
TGTTGTTGCTGATACATACA
>CsCML67

TCTCTAAAACTCATCTTATACGCCTCTTGTGTTTATGATTTAGTGTGTTTGTGAAATTCA
ATATGTTACATTTTGTAAGG
TTGCCCTAAGAGCACATGATAAGAAGGCAAAAAGGACTTAGGGATTTACAAAAAATGA
CTCAATGACTCATAAAAATTTT
ATTTTTTGTGTCTTTTTTTTGTCTTCTTATCTTGTGCCCGCACAAGATATGAAAACCTATT
TTATATATATCACAGGGTT
CTAAAAAGCGCGACATGACGGCGTTAACGCGTAACTAAGGCGCGAGGCGTGGCATGC
AATTTACGAAATAGATCAATACG
TCAGCTCTAGGTGTGACGTGAGATGAGACGTGAAAAAGCACGATAAGACGCGCTAAA
ACGCGAAGCGTGACTCCTTTTTC
TAATTCAACTCCAATAAAAAAGAAATTTTTGAAAAAAATGATAAAGATTACATTTAATAT
TAGTTTTTTGAATTTATTCA
TTATGTATAATATTTTTTATATAAATTCAATATATATAAAATATTTTAAAAATATATAAATATT
TCATATTTTGTGACAC
TTTAAATCATAACTTAAAATACTAAAGCTTTGTCATGGCTCATAAAACGTAAAGCTTAGC
CATGCCAAAACATTACGCCT
CACTACTATTCGAGAACAACCACTGTTTTAAAAGGCTCGTAAAGGCTTCGTAAAGGCG
TGAGGCTTGATCATGCCGTCAC
GCCGCCACACCGCCTTATGACACATGTTTTTGTAAACTCAGATATATATAAATTATGATAT
AAATATATAAATATTTTCA
TACTTTTTGTCGTTTTATGTTACGTCTTATAAACGCAAAAACTTCACCTTAATCACAAAA
ACAAAAACGTAAAACTTAGT
TATGCCGTCGTGGCACATCTCACTCGATTTATAACCTTGAGAACAACACATAATTTGGG
TACAATACTTTAGCATAAAGA
ATCAAATCTTTACGGTATATATGTGGTTACACAATTTGTGAATCACTAGGCTTTATGTCCA
TCAGTTATGTGACACGTGG
AAAGAATGAGGAACACTGGGCGGTGGAGGTTTCTCTATGTACAGTAGAAGAATCCAA
ACCTTTTTTCTTGACCCATCTCC
ACAAAGTAGAGAATCATACAGCTGTTGACCGCCTCTTCAAAATATCCTTTCGTCATCGC
TAACGTATAACCCCCCAATTTT
ATATTATTTTATTTTCTTGCACATGTTATAGTCTTTATGAATGTTTAGCTTGTTATGGAATA
ATGACAAGTGCATTATTA
ACTAGAACAAATCACCTTCTTTATATGATCTTTAATTCAGTGTCAACTATCACGCAGATC
TTTTGGTTCATTTGCCAAAA
ATGACATCACGCCACTAATAAATGAATACTCTAAAACAAAAGTTATGTTTTGTCAAATG
TCGACATAAATCTCATTTGGT
AAGGGTAATTTAATGCATTTTCTTTTTTTGTTATGCATGAAATCTTTTCTTTCTAAAAGTT
CATGGTAAAGATATATTTT
TGGCGAGTGCATTTTAATATTGAGGATAGCAAAAAGAAAAATAAAAGATTGTTTTTTGA
CATTTATATTCTCACATAACT
TTTTATACATTTTTAAATTGTAAAAGTATTAATTATCAACACACTCTAAAACAAAATGTG
CAAATTAATTTGACGAGTGT
ACAAATAACTTCCATTTTTTTCCCCCTTAAAGCATGATATTACGCGTTTTGCTACTTCATA
TTAGCAGGTCGATATTAGT

ATTCCTTTTTTTCCTGGTCAACAAATAAATAATTTAAAAACTAAGAAATGAGCGTGCTC
CGAAAGCCAAACTTCCTTAGC
CATATATTATTCCATTGACCCCATAACCACGCCTATATATATATCTAACGAATCCCCCTTAT
TCCCGGAGGAAGAAAAAG
AAAAAACATTCACACACATTCATTCCAAACCACAAAAACCCTTTCATTAATTCTCACAC
ACACACAATACACCAACAACT
>CsCML68
GAGTGCTATATCAGTTAATTTGGGAAGATCAAAATTGTATTTTCTTCAAGCAAATATAAG
TAAAACCTTAAAGTTTTGGG
AGGGCCATTAAGATCCGTCTATGGTTATACTAATACGAACAGTCATCTAATATGAGTAAT
TGGGTACGTGGTAATTGTTA
GAAGAATTAAAAGTATTAAAAGAAAAAAAAAATACGGAACAAAATAAAAAGGGGGTG
AGGATGGTTAAATGTACCTATTT
AAAATATGAATAAAGATGTAAAAGAGCAAAAGAGAACTTTTCTGATTACACGTCAAAA
ATCATGTGTGTTCATACATTAT
AAAGATTCTTTGGTATCTATATTTTCTAATGTTGTAACATGGAAGGAAGAGGTTTCAATA
ATTACTAAAGCTTTAATGAG
ATAACCCCAAAAAGGTAATGGATTAAAAGATTCCAGGTATCTCTCATATGCCTCAATGC
AAACAAAACCTCAAAACATTC
TCATATTCATCATTAAGATCAAATCATCAGCTTTGAAAAATCAAGTATGCCTTCCAAAGG
AGACGAGGATGATCGCAACA
AAACCTTAGGTAAACCAAGAAAGTTTCTTGGAGTAAGACAAAGGCCATCAGGAAGAT
GGGTATCCGAGATCAAAGACTCA
ACAAGAAACCTAAGGCTATGGCTAGGCACATACGATACTCCCGAAGAAGCAGCCATGG
CCTATGATAGTGTTGCACGCAT
TTTACGCGGGAGGAATGCAAAGACAAATTTCAAGTACGAGTGTCTCATTGCTACTCAT
GAAGAGATCTCCAATTTACTAA
GCAGAAACCCTAAACTCTTTCATTTTCTCAAATATGCCACCATGAAAACCTTTTCATCAT
CCTTAAGAGCAAGTGATATT
GTAAACAATATTACACAAGATGAAACACCCCTTGCTTGTTCTTGGGCTTCATGTGATGA
TGGACTTAGGGTTTCTTTTGA
TTCTCAAGATGACGGAGAGAGAGTTTACCGAGGCTCAGATCATCAACTAGAAATTCTAAG
GTTTATTCTTCTGTTTACGTAG
CTCCCTCTTTCAATGTAACTAAAGAATAGCTTGTTGATGGTGTTGATTTCAACTTTTCTG
AATTTATGTTGCTGATCAAT
TTAGGTTTTGCCAAAAAATATTATCAACCGTTTCCGTCTTGTTAATGTATCAAATTTGTTT
TAAAGACATCATTACTTTG
TTGTATTATGTAAGTGACATTGTCTATCAGTATGTTTTATGCTTTATTGTATGTAGTGTTTT
TTTAACGGCTTTTGTTGT
GTGTAGCGTATCTAACTGTACAATACATATCATATTATGTGCATAGAAGATTTAACACAG
TACAGGTTCTGGTGAGTGAT
AGACTCGTGTATTTCTGCGGGCATCGATTCTACATGTGTTGGGATCAGCATTTCAAGTG
ATTAAGTAAATGTGCTAATCT
GTAACAACCGTCTAACATAAATAGAAGCACAGTAAATCTAGTTAACACAAGAACTCAT

CACACGTGACACGTCTAAAGAA
GAAAAGAAGTTAAGATCTATAAAGGCTTGTATGAGTTACTAATAGTCTTAACCATATGGT
TTGATGTTGATTTTTGATTT
TGATTCATGATATCTCTCGATCGTGTCTATGTCAAAATGGTTATTGTGTTTGATCAAACAT
GATTTTAGGTGCTGTGATG
AATTCAGGAATTCAAAGAAGTGGATGAATGAAATTTGTTTTGTCTTTCTAATTTTGGCT
AAAAAATTAAAACATTTCAAA
ATTGGAATTTTCAATACTATATTTCTATGTTTAGGGACTAGATCTATATAACATTGACTAA
AAGACGACCAAAACCAATA
AAAAAATCTCACTTTTTATGCCATTTTATGAGGGAGGGAAAAACATTCTCCTTGTGGCT
CTCATTCAGCTTCCATAACAT
ATATATGCATGCAAACAAGAGGCCACAATCAATATTTGAAGGCCACAAATCTTCATCAC
CACAACACAACCACCATCACA
>CsCML69
CGATTTTCTCCGCTTGAAGAGAGCTTGTTGACATAGTGCAGTGAACCTCGAGTTGGGT
ACTCGGGGAGCGGAGTAGGGCG
AAGAGTAGCTTCATTACCTAACCAATTTATATCTTAGTGTGTTGGTTTGATCTTTCCATCT
CTTTACTTTGATCTTATAT
TTTATATTTCTTAGTTATCCTTGCTAGTTATTTTTAGTTTAATTATATTAAGTTCATATTATT
GTTGTTTGATCTCGCAT
AAACTCGAATCTAGTTTTTTTGAAAAGAACATATTTTAAGGATAAAAACGGTAAGGATA
TTTAACCTCCCTTTCTAGTTT
TTCACATAATTAAATTAAAGGTAAAATTGCAATTTATAATGAAATATGAAAAACCATCAT
TTTGAAGCTTATAAAAACCT
CTTATTTATTCTTAATGGTAAATTTATTATGAAAGAGCCAAAAAAGGCAACAAGAAACA
ACTACTAAACAAGCCCAAAGT
AAAACAGAGCTTAAAGGATACATCCCTAAAAGCTTACTTTTGCTGAAAATGCACAATG
GCCATGGACATACCCATGCTCA
ATCATAAAGTTTAGTCAAACACTTGTTGAAACTTATCTAACATATATAAGTTAATAAGCT
TATAAGCCAATCATATTAAA
CTTAGACTAACATCCCTGAAAACAAAAAATATTCCAATTAAACTTTTTTACCCATTTTAT
AAAGTTAAGAAAAAAAATTC
AGTCTCACAAAAGATTGATAAAACTTATCCATGGGCTTTTAAATTTATTGAAAAACACC
CCAAGAATGCCAACGTATGTA
CTAAACATAATACAATAATAACTCAAGCTAAACGAAAGTAAATGAAATTATTTTCCTAA
GATGAACATAAACACATTAGT
GACGGAGCCGGTAGGGGGGGCTGGAGGGTGCTCGGCCCCCCCTAACCGGCTCAACTG
CCTTAGATATATATGAGAGAAAC
AACCTTGGATTAGTTTCATTTTGATGAAAAAGCAAAGAGGGAAATATAGTTAAGCTCCG
GCCATAAGATAGACGAAGAAG
ATGAAGGTTTAGGTGAGAGAAAAACAAAAAGTGACAAAATTTTGTATTGATAACTGGA
ATAATATGTAGTGTACAACTGG
AGTATATTATTAAGGTCAAGAGTAAAAATCTTCTAATAATACTTTTTTTTTCACCTACACT
GAAGCATACACTATATTTT

GTTTTGTGGTGTATTATATAAATCAACTTACATTAAAAAAATATAAGTGAACTTTTGTTA
AACTATAAGAAATTTGTTT
CTCGACATAAATTTAAAATCTTCTTAGTTTGTTTAATATGTTTTGTTACAAAAAAGCCGC
TGAGTCAATAACATTTTTAT
TATCTCTATTATAGTTGTAACTATAGCATTTAAATCGATACAATCTTACACCTTTTTTTCTA
ACTATATGCCCAACTGGA
TAAAACTGTTTACCAATTGACTTGTTCACTGGTCGATTTTATCGCATTGGATAAGTTGAA
GAACCTTCGGATAAGTAATT
GTCAGTTACCACGGAAAAAAATATCATTGGACCCGTATATCGAACGATTCAAGCTTCAAT
CTAATTTTTAAAAGAAATAAT
TGTATGTTTTTACAAAGAAAAAAAAAAAGCCCCCCCTTAACAAAAATTCGTGGCTCCGC
ATAAATACCACTTTACCCATGA
TACCTTCTACAAAAGCAGAACACGAGCCTTAGATATGTGTAGATATGACAACCTACATG
TTTAGGTGTATTAAAACTTAT
CCATATAACTTTACTCTCAAGATCGACATGCATGGAAAATGCTACGTAATTGCAATGTGT
AGTGTGTACAATCCATATGC
CATCTTCTATATTCCGGCATCTTGCTTTTTAAATTTCCCCATGCTTTCTCATTTATTGCCCA
CGAACCACTACCTTACAA
TACATCTATAAATATCACAACTAATATCGACCATCCATCACAATCTTCTTGCAAAAAACG
TTGATAACCATTAACCAGCA
>CsCML70
AATGCTTTGTGTGTATCTCCTTCTCCAACCGGTTCTGTTTCGTTTTTCTTTCACACAAAG
TCTCCTCGGTCTTTCTTCCC
CAACACCAGATTCGTTTGGTGTTCCAACGATTTGCAACCCCTACGTAAATTGGATCGTG
CACTATCTTCTCTACGCGTCT
CCTTATTCAACGTATGAAGTTTGTTTCTTTGGAAGAATAAAGAAAAACAAACTGATGTC
GCTCAAAGATGGAATTGAATT
CTATCTTCTCCTGCGTCTTGTTTTCTTGGCGTCTCGATGTGGTTTGTATGTGTCGAATATG
GAATGATTGAAACCCTTGC
TCTTGATGCCAATTGTTGTCCCAAATAACTAGATCGATTGATAATAGGTTGCAAAGTAA
AGAATTGAATAAGTAATGTAA
ATATTGTCTATGTGATTAACCTTTTATTCGAATACATGTATACATATATATAAGGTTATGAA
ACGCTGCATGTGAAGCGT
TGCAACCTTTCACCATGTCCCAACCGAATCTTAACAAACATAACTGAATTTCGGTTGTA
GACTTTCATTTTAACATCTTT
GGTCCTTAAGGTTTTAACTAACATAATAACAAGAAGTAATGTCAAGGTTATCGGGTCAC
ATCCTGGCATTCTTCTGATAA
CAAGAGGCAATGGATTTTGATATTCGGTTGGTCCCCACTGGTCCATACCATCTAGCGGG
TAGATAAAGTGTTGGTAAACA
CATCTCTTTAACTGTTGTACTCCCTTAAACCTTTCACCTAAAACAGGTCTCATGAACTTC
CATTTTGTGTTCGGGTTGTG
AATTGGCCAATGATCTCCTATTTTATCCTGATCTTCAACATTAAACTCATCATCAGGACC
TACCTCCTTTTCAATATATT
TTTCTCTTTCTATTCCGTTGAACAATCTAGCCAAAAAGTTATCATCAAACCTTTTAGTGG

GTTTCTTCTTTGTTTTAGGG
GCTGTTTTTTGTGATCTTACGTCAACTACTTCATCTTCACCATCAGACAAGTGATCTAAT
GATGCAGTGTCATCATTATC
AGAGCTGTAAACAGAATAAACATCATAATCATCCTCCTCGCTTTCACTAACAACACTAT
CTGCTCTAATGTGTTCACTAA
ACTCAGAATGTGCCATGTAAATATCTGTTTCACCATTATTCCTAAGAGCATACTGTCTAC
AATTACGCACGCCATCATCA
TTTTTTAAAGACCTTAAGCCATCTTCTAAATTTGTACCAGACACAAGAAAGTATAAACC
TTTGCAAATAGAGTGACTTTC
AGTTTCTAGATAAGAAACAATCTGAGAGTAATCCATCGAAATTAAGTCTATATTTTCGAG
TTTCTTAAGCATACCATAAT
CATACAAAACAGGGTTTGCTTCAAAGACTCCACCATAATGAAAGTTTATGGTTATTTGA
GTAGAACATGATGCCATCTGA
AACAAAAAATAACAAAGAAACATAAATTGAAGCCCTATTAACAAGTTACATGTGAAAA
TAGATGAATTACCTTATGTATG
AAGATGAACTGAGATCAAATGTGCATGAAGATGAACCGTTTATGAGGTGGTTGTGTATT
TTTTTTATGGTTGAGAGTATA
TGACGACATATGTTAATACTAGGGTTTAATCACCAAAATTATTTAATTAATTAATTTTATT
TCCAAATGTGCCACGTCAT
GCTCTTTTCACCGGCTTAACCTGTAGCATGATGAAAATAAACGGTTTCCTAAATTATAGT
GATGGTTTTAAAAGAAAAAT
TAGTAGAGTGACTCAAATAGATATTATGTGAAAGTAGGGTGTCTTTTCAGTGCTAAAAC
CCATCAATAAGTAAGCATGCC
GAAATTGCCATATTGACTAAATTAATTTTAACCTAACAATGGTCACCAAATTACAGAGA
CGGAGAAAGGAATATTTGATG
AGATATTGAAAGGAGAAATTGATTTTGAAAGTGACCCTTGGCCATCTATCTCAAGTAGT
GCCAAACATCTTGTCCAAAGA
>CsCML71
GAATCTTTTGTTTTTGAACTTGTTTCAGTAACCGAATTGTCACGTTTGCGTTTAAGAAC
CTTAGAAACTTCATTTTTTTT
CCTGTAAATGCATCCTTTACGAACTCATCATCACTTGGTGAATATGCTTTTACAGGAACA
TATTTCTTTGCCTTCTTTGA
AACTGTACATATCAAACAAACACATAATACATTTATCAGTAACTGATGAACCAAATAAA
ACCGTGAACAACTAACTGATG
AACATTACAGTTGATGACTATAATTTAAACAAAAACATGATGAACAAAAGATGTTGGCA
AGTCAATAATCTTCGCAAACT
ATCACTATCAATAACTGATGAACAAAAAAATAACTGATGAACAAGGATATTATCATGTTA
CTGATGAACATAACATATAC
TTTGTAAAACTATTATCATCTAACAAAACCAAACAGCCTAATAATCTTTGCAAACTATCA
CTATCAATAATTGATGAACA
AAAAAAATAACTCATGAACAAGGCTATTATCATCTTACTGATGAACATAACATATACTTT
GTAAAACTATTATCATCTAA
CTAAACCAAATAGCCTAATAATCTTCGCAAACTACCACTATCAATAACTGATGAACAAA
AACAATAACTGATGAACAAGC

CTATTATAATCAAACTGATGAACATAACATAGACTATGTAAGACTATTATCATCTAATAAA
ACCAAACAGCATAATAATC
TTCGCAACTACCACTATCAATAACTGATGAACAAAAAAAATAATTGATGAACAAGCCTA
TTATAATCAAACTGATGAACA
TAACAGACTTTGTAAGACTATTATCATCTAACAAAACCAAACAGCCTAATAATCTTTGC
AAACTATCACTATCAATAACT
GATGAACAAAAAAAATAACTGATGAACAAGCCTATTATAATCAAACTGATGAACATAAC
ATAGACTATGTAAGACTATTA
TCATCTAACAAAACCAAACAACCTAATAATCTTCGCAAACTACCACTATCAATAACTGA
TGAACAAAAAAAATAACTGAT
GAACAAGCCTATTATAATCAAACTGATGAACATAACATAGACTATGTAAGACTATTATCA
TCTAACAAAACCAAACAGCC
TAATAATCTTTGCAAACTACCACTATCAATAACTGATGAACAAAAAAAATAATTGATGA
ACAAGCCTATTATAATCAAAC
TGATGAACATAACAGACTTTGTAAGACTATTGTCATCTAACAAAACCAAACAGCCTAAT
AATCTTTGCAAACTATCACTA
TCAATAACTGATGAACAAAAAAAATAACTGATGAACAAGTAACTGATGAACATAACAT
ATACCTTGTAACAATCTATTGT
CCCACTTTTTAATGAATGCAGTACCTTCATTCCCGGGGAGTTGCAGACGGTCGTGTCTA
TTTTTCGATCTTTTTTCCTTG
AAGAGTTCTTCTATTTTTCTGTCATCTAAAAATTTAAATAAAATAAGATTAAAATAATTCT
AATTCTTCCTGAACAGTCA
GATACTCTATTTCTCCATCATTTTCATTTTTATTTTCTTATTTGCTTAAAAAATTAAATAAA
ATAAGATTAAAATAATTC
TAATTCTTCACATAATTTCTCCATCATTTTTTCATTTGTTTTTCTTATTTGCCTAAAAATTT
AAATAAAATAAAATTAAA
ATCAGATTATTCAAAAATAACTAAAAAACTCATATATTTTTCTTTATTTCTAATAAACTAA
CACATAACTGATGAATGCA
AAAACTAAAATTAAAAAAATTAAAACAAAATTAAACAAAAATTAAAAAATAAATCATC
AACTTACTTTTAGTTTTCATCT
TTCCGAGTGCAAATCGTTCACTTTAATCCGTAAATTTGAGTGAAAATCGTGTTTTTTTTA
TCGGAATAAACCCTAATTAG
ATCCCCAATTATAGAGAGATGATTTTGAAAGTGACCCTTGGCCATCTATCTCAAGTAGT
GCCAAACATCTTCTGCAAAGA
>CsCML72
TTGTATGTTTAAAAGTTGTTCATTTTGTTGAGTTGCTGTGTTGACTTAAGCTGTTATATT
GAAATGTGATTGTCAGTTTT
AGAGGGTGTTTGTTTCGGATTATATAGTTGGCTTATGGCTTATTTGGTTTTGGGCTTATGT
TTTAGAGGGTGTTTGTTTC
GTATCTAGTTATTGTACTTGCCACTGAAAAAAAATCACTCTTATTCGGAAAAAAAAAAA
AAAAAGGTTTTTGGCCCAAAA
CAGCATGACTATGTTTCTACTGAAAGTTAGCGTTTAAAATGATTTTTCTACTTTAGTTGG
TTTAATTCAAGTTTTCTAAG
AGTAAGTTCTAGAAGATATCAGATTCATTCTTCGATGAGTTTTGTGTTTGGTTAAATTGG

TGGCTAGAGTTTTGAGTTAC
TCTAGTTATGGTATGCGCCCGCGGTTTGTGTTGAAAGTGATCGAGTGTCTATCTATGTCT
TAGACAAGAATTTATGTCCT
GAGCCGAAAACAGACCAAAGTTTTCAAATTAATTTAAGTATCGAGATTGCAGAGTAAG
CTCTTGACGATATTGGATTCGT
TCTTTCACGATTTCCATATTTAGTTGCCCTAAATCTTTTAGTTACTCTAGACTCTAGTTAT
AGTATAAGCCTGCTTTGTA
ATGTAAGAAATCGTGTATAAGAATGTCAGGACTTGGGATGAAAGGCAGATCGCATATAA
GATTCGGATTTTAGGAACCAT
TAATTAGGTAATAGGATTACCAAAGGGCTTTGGCTCGGTTGGTACCCACCTCCGAAAAA
CCTATGAGCCCACTCGAGAGA
TTTTGTAGATAAAAGGTCATGGGTTCGAGTCTCGGCTCGTGCTCACTCCAGTTGGGGTG
CTCCTCTAGGCCAGGCCAGGA
GAGTTTCCGGAGCTATTCTTTGTGGGGTGGACAATGGCCGGTGCCAAAAGGCACCCGG
GGTCAGGGTGTTCCCGCCAATC
TACACCTTTTATGGAATTAGGAAATAGGATTTTATGCTGATATATATCTGTCTAATTCATTT
CCAGCAGTATCTATTCTA
TGTTTATCCCTATAATTTAGTATTTGGAACCATTAATTAGGAAACAGTATATATTCTTATGT
GATTTTCATGTTTAGTTG
TTCAAGTCTTCACATGTTTTGTATGTCTACTATCTATATTCAGCTTTTATCATTACTCAATA
CACGTACAACATAATAAT
ATTAGTTTCCTGAAGTTATCACTATGTGCAACTCCCATATGTTCTATATAGGTATTACGTG
TGTGGTTAAAGAAGATTAG
CATGTTTTATATGATAAAACGCGTGACATCTATCTCCAAGTCAGTTTCAGGCAACACAC
ATGTTCTGTGTGCTTAACCAC
TCGTGCATATAAGAAGATAAGTATGTTGTGCATGATTAAAGTGTGACGTTAATCCCCAC
AACAAGTCATGCAAAACCAAT
TTGTAATTCATAGGTCATGTCACATGTGCTTGAAGAAATTTAGCATGTTTTTACATAATTA
CATGATGAAACATGTGATG
TTTATTTCCGACCGATTTTATTCAACATCCATATTTTCCACATAGGTATATTCATGTGATTC
AAGAATATTAGTATGTTT
GATTTTATAAATCCCGTGATATTTGTCTTCAAGCAAATTTTCTCAAGTTCATATTTATGAC
AAGATTTGAACAATACCAA
TATACGTTTGCTGAACAAAATTAGTACTTGGTAGCACAACAGTATTTTCTCTGTTTTTTG
TTTGTAACTATTGTAGCGAG
ATTTTCTGATGAAGTATTGTTTCTTTTTAAACAGATATAGTTTGATTGCTCCTCTCATCAA
GTGATTTGCAAACTTTAAA
TCATAGCAGCAATAGGAAATGGTCAAAAGGTTATTTCTAATCCTTCAAGAAGTCGCTAC
AGTTTTTGCATATATTGCGTT
TAGTTATAAGGCTTTTGACATTTTTTTTTTTTACTTTATTCAGGATCAAGTATGGATTATTT
TAATGTGCAACAAATAAT
>CsCML73
TGACTGCTCCCATTAGCAACTAAACGAGCCTTATAGCGGCTCAATGTCCCGTCAGCATG
AAACTTATGCTTAAATAACCA

CATAGATCGGACCATATTAACACCTGTGGGCTTTGGTACAAAAATCCAAGTAGCATTTT
TAACTAACGTGTTATACTCAT
CATACATAGTATTACGCCATTGAGGATCTTTTAGAGCTAGAAACGGAGATTTGGGAATA
GGAGAAATCGAGGACGTGTGT
AAGGATAAACGATCAATGGGTTTGACAATGCCCGATTGAGACCTAGTGATCATGCGGT
GAACGCGTGAGGGTTCGGTAAT
GGGCTGTTGAGATTGAGCCGAGGGATTGGTTGTTATCGTGGGTTGAGCAGTTGGTGGG
TGTTCATGGGTTTGGTTAGTGT
GTTGTTGGGCCGGAGTGGTTGGGCTAGTGTTTTGATCCGAGAGAATGGGAGGTATAAG
GGGTGACTATTGGGTTGAGGTG
TTGGTTTGGTTGGTGGGTGGAGGAGTAGTTTGGGCCGGGTTAATATGATTAGTGGAATG
GGGTTGATCTGGTGGTACAGG
TATATGTAATAGGTGATCAAATAATGGAGTATGCATATGTTCTAAAAAGGTGTACGAGGG
TGGAGAATCGGGGGTCATTG
AGCGGTACTAAAATTGAGTTTCGTCAAAAGTGGCGTGTCTAGAAATAATGATTTTATTT
GTTTCAAGGTTGAGGCAGCGG
TAACCACGGTGATAGGCGGGAAAGCCCAAGAAGATGCACGGGGTTGCACGAGGCGCA
AGTTTGTGAGGGGAGTGTAGGTG
GGGGTAACAAAGGCACCCAAATATGCGTAATCGGGTATAGTCAGGTTCTTTTTGAAAA
AGGCGAGTGAAAGGGATTTCGT
TGTTGATGGCGGATGAAGGTAGAAGATTAAGGAGATAAGTGGCCATATGATGGGCCTC
GACCCAAAATGAGAGGGGTAGG
TGTGCTTGAAAGAGTAGGGTGCGTATGATATTATTGATGGTTCGGATCATTCGTTCGGCC
TTACCGTTTTGTTGAGAGGT
TTTTGGACAAGAAAATCTAAATTGTATTCCATGTATATTAAAAAGTGTGTGAAGTTGGTT
ATTATCGAACTCACCACCAT
GGTCACATTGGAAACTCTTGATTTCACATTTAAATTGATTTTTAACATAATTACGGAAAT
GTAAAAACTTATCGGAGAAG
TCCACAAGTCGGAATGAATAATATCAAAGCAATTTTCAACAATAGAATATGAACTATGA
AAACGGAGCTTCACATGTTTG
CCAAGTTAACAAGCATTACAAACATGTGTAGTCTTTTATTTATTACACGAAATAAAGTG
GCGAGAAGTAAGAGATCGTAG
CACTTCATCACCGTGATAACTGAGACGTTGATGCCTCGTGGAGGAACTTGTGGAGACC
AAAGCAGTAGGGATAGTTAACG
GCTTGGTAACTGGGTAGAGATCGCCCTAGCTGTCACATCGTAGGAGGATATGGCGAGT
CAAGAAATCCTTTACAGAAAAA
CCAATATGGCGAGTCAAGAAATCCTTTACAGAAAAACCAAAATCGCTAAACATGATAG
GTATATATGATTACAAAAAGGA
GATTCTAGAACTAACACATGGACTGTGACTAATACATACGTAATGGGCCTAAATACTAG
TAAATACTCAATGTTATACAA
TTATCGACTAATAGTTTTTTTTTTTTTTTTTTTTTTTTTTTGGTTATCTACCATTGAAATT
ACCTTAAAAAAAACTCTT
ACATTTTTTGACATATGTAGAAGTAGATCATTTTGTTTTATGAATTTTGGAATATCCCATA
TCTTTATTGAACAAACACA

CGAAAAATACTTAAGATCCAACAATAACGGTATTTACTTTTTCATCCTAAATATCGTAAC
CACCCTATGAATGTGTAAAT
ACATTTATCTCAACCCAACAATCAAAACTAATATTTATGCATGCATGTAATTTTCCCATAA
TAATCATAACAAATCTATC
>CsCML74
AAAGCTAGTTGGAGGAAACTGTGGTGAAGTTGAAAAGCTAGTTGGAGGAAACTGTGG
TGAAGTTGAAAAGCTTGTTTCCG
GCAGCTTTTCTGGACTTGGGAATTGCTGAGAAAAAATCTTTGCGAGACATTTTTGTGTG
TGAGTTGTGTGTGTAACTTGT
GACTGGTAGGGTTGTATATATAGGCTCATATAATCCCGCTCAAAACTATACCGTTTGAAT
TTAAAATTTGAATTTGAATT
TTAAAGTACCCCGCTCCTATTTGAATTTGAATTAGCCCGCTCCTATTTGAATTTGAATTA
CCCCGCTCAAAAGTATACGG
TTTTGAATTTGAATTTGAATTTTAAATTTGAATTTGAAATTTGAATATGATTTTTTGAATT
TGAATTTGAATTTCAAAAC
CACCCCTTTAAATAACAATTTATAACCACCGTTTTTCCACATCTACATTAATTCAAAAAA
AACACAAATGTCTCAAAATC
CGAAGTGGAACAAGTCTGAAGAAATGTTGCTTGCGCAAGTTTGGATAGAAATTTCGCA
AGCTATAGATATGGAAAATAAC
CGTTCCGAAAATATTTTTTGGAACGATGTTATGGAGATGTTCAATGATCAGACTACCGAT
GACCATCGTACAAAAAATTC
AATCACGGGTAAATGGACGAGGATTAACGTCGATTGCAAAAAGTTCAATGCAATTTAC
AAACATTTGCAGCGTCGAAGTG
GTCAAAATGACCGCACTCATCTCGAAAATGCGAAACTATACTTTAAGCAACAATTTGGT
GGAAAGGAGTTCAAGTATGTT
CACGTTTGGGAGATTTTGAAAAATTGCCCGAAGTGGGATGTCGAAGATCCGATCCATT
AGTATGTGTTGTATTTTTTTTT
ACTCGTGGCGTTTTTTTTTTCCCGTATCGTGTTTTTTTATTATTACTGTGTTATGTATTGTA
TTTTTATTTTGAATGAAA
TGAATTTAGTTTATTTGGGAAATTGAATAAAAAAAATAAAATGGTAGTGGTGGACCCCA
AATGAAGATGTTGGGGTTTGT
TATGAGTGGGGGGGGGGGGGGGGGGGGGGTTTGTTGTGGTTAGTTGTGGAGAAGTGAG
ATGCCGATGTGGCGCTGAGGTGG
TACTGATAAAGTGATGATGGTCCATCACGACACATTCCCCTCTAAGAGCATGAGCAATG
CATCAAGTCATAAAAGACTTG
ATGCATTTAATGCAATTAATATTTAAGTGTACTATTCAAATTAAAAAGTCATTCTGAGAAT
TTACTACAATGCAAAATGT
TGTATCAAAAGCTTTTCAAATATTTTATATAATAAATTATGATCAAATCTATGCTTTAGAA
GATGAAAAGCTTTTGCGTC
AACCACTTGAAGTTTATCAAGTCTTTGGGTAAAAAGTCTTTTACCAAGTTATGGATGCA
ATGCAAAAAATGGTGTAAATG
ACTTTTACAAATAGTACCAAGTCATTCTTCAAGATAGCATTGAAGATGCTGTAAGTCTC
ATTTGGCTCAATTTCCAGAAC
GACACATTGTTTTTACTTACTCCGTATAATATTTGTAAGCATTACATTTTTACCCTTATATT

ATATTAAGTAACACTTTA
AATTCATTCAAGATGTTTAATCTATTTCTTGTTTCTTAAATGTCTTTTTGATTTTTTTATTT
ATCTTTATACTCCTAATT
TTCTTCACGTGTATTTTAAAATATATACCACAAAACCTATACACATAAGTTAACAAAACT
ATGTGCTCTAAACAAGTTAT
AGGCCCAAAAGAATTAGACCCATCTTAACTAGACTAAGCCCATAAAAGCACTTAACAG
CACGATTACTTTTTAAGAAATC
CAAAATACTAACTCGATTTATTTCAACCAATTATTTCTTTTGCATCCACGTAATATGCAAA
CATGAATTCCCATGCATTT
CTACCTCAAGTTTCTTCCATATTCATCATAAACATCCCAACAAAACCAAACCAAAAAC
AATATCGTAAAATTTCCCAAA
>CsCML75
TAATGATTATAATTATAGAAAGTGTTGATAAACATATAATATTTTATATTAATTAAATGTTG
ATTATTACAATAAGAAAA
GCTTAAGTACAACCCAAAAGAGTTGTACTTAACAAAACCTATTAAACATCTGTTAAGAT
GTTTGCTATTCACCAAATATT
AAACATAAAACAAAAAGAGGAATGCTAGGGCCACATAAACTTTTCACAAAAAATCTCC
ACTCGCTGACGTGTCAGTGGGT
GATTGGTTTTTTGTGGGATGTGGGTGATTGGTTTTTTGTGGGATGGATGTGGATCCCAC
AAAAAACCAATCACTCGCTGA
CACGTCAGCGAGTAGGGATTTTTTGTGAAAAGTTTATGTGAATGTAGCAAATCCCAAA
CAAAAAAGACAAAAATAGTCCA
TGTGGCTTTTTTTACAATTACAAATGTATTATCTGAAAAGTTGTAAAAAGGTCCCTACGA
TTTACTCTTGACTACATAAA
CAATCCCTGTTTTATATTTAAAGACTTTTTGTGTAACAAAAAGTTACATAAGCATGTCTC
TAGTTACTTGCGATTACATA
AAAAGTTTATTTCTAAGGACTTTATTATACATGATGTTAAAATCGAAAAGGCTATAAGAA
TATGTAGGTTTTGCTTGGCA
CAAAACTAATAATTGGAGCATGCCGGTTATATGATAAAATCAAAAAGAACCCGTTTGAT
TGTCATGGGTTTCTTGTAATC
TTTATTGATAAAAATAACCTTGATTGACGTACGTTAGCACCTATCAAAGTCACCGGTATT
GTGTGACAAAACTCAATGAA
TGTTGGGGTCTGCTTGGTATAGGATTGAAATGGAGCAAGTTAAAACTCAATAACGCCTA
ACATCAAGAAACTCAATAATA
AATAAAATGCTTATAAAAGCTTAAATAAATTTTAAAATTGAAATCTTAAAGGCAAAGCT
CAAACATAAACGGATACATTA
GAACAACGAGTAATTGCTATATGAAACAACTGAAATGTCGAAGCCAAAAATTCAGTAT
CTAATTTTCAATAGAGGTGAAC
TATGTTTTTCCCGGTGGTGAAATGGAAGGGAAGAGGAGAAAGATTAGTACGTTTTTAC
ATAGCAAGACATTTTGAAAATT
CGCTATCTTAATACAAAAGGGTATATTAGACATTTCATAATTTATTTATGTGGTAAGAGCA
AGTGGTGCAGTGTCATATG
GCATTGTTATAGGCAATTTTTCTTTTGTCTTATGGCATAACCAAATTGCACCGGCCCAAA
TGCCAAAGCTATGTTATAGG

GCCGAATACATTTGGGTTAGCGAGAGTTATGAAGCTCTTTTTTTTTGAGTGAGTAAGGA
GGGAGTTATAAGAATTTTAAT
AAAAGTGTGTTTATGTTATAGTGATGTTATATAGGGTATATGCTGAGGTGGCGGGAGGAT
GATATGTTATAGGGTGCAAC
TACACCTACCTCTCTAAGAATCAAATTATTATGTGTACATATCAATCCCCATAATTATTTG
ACATTGTAAATTGCCGTCG
TTATGCATGAATAGTGCCCTACTCTTAAACAAATATATAAACTTGGTCCAACTTCATAAG
TTTTAGTTGACTCACCAAAC
TGCACACGTTACTATTTATATTTAAAACCACTATTTTTAGTTTAATTTATAATGTTTATTGT
TTTAAATATATTTTTTTA
GGTTTTTTTAGAAGCACAAATCTCTCAAAAGTGAAGATCGTCCAAAAAGCATAAAACT
TAAGGCATTGACTCTTGACAAA
AGAAGGTGTAAGTCAACTCTTACAATTTCAAAGAAACGACTTCTTTTACCTCTAATTTA
TTGAACAAAGAATTCTCTTTT
GGACACTTATGAACTCTTACCCATTTTCTACGCAAGTTTCCTGTTATTTATGCTACCATAT
TTTCATAATATCAAACCAA
AAACTTCTTTATATTTCTCTCAATCAAATTCTACATATCATATTTTTACAACAAAGATACA
AGTGCATAAGTTGTTGACG
>CsCML76
CAGACTGTCTGCAATGCTTTCTGAATTCCGAGTAAACATATCCTTAACTGTATTATATTAT
TGTTTGGCTGTCTAGCTAA
CAATTTATATATATCTGCTTATTGTCATACCCATAAAATATTGATGTTATTGGTTTTGTTTT
GCAACAATATAGCCTATT
ATAGCCATAGTATTATAAAGTTCTCTGTTCTGTCAAATGGTACCATCTTTGTGTAAGGGG
ATTTGTAAACCAGTAAGTAT
ATAGGGTGATCTGAGCCTAGTGTAGGGTTTATTCGGGCGGACCGCAGATGTCTTCAGTT
TATAAAGAATGCAAAACACCT
ACATTTTCTCTAATGGTGGACAGACACAATGAAGATTCCAAAATCGTCTTCTGAATCAG
TTCATCTCTTAATTCTTGGAT
CAGGTCGCAATGCACAAACATTGAGCTTGCAATTTATGTATGTTATTTTTAAAACCTTTT
TTGGGAAGAAATATAGCTAT
ATTGATTGTTAAGTTAAGAGATCCAATGAAGTTACATATATAGATAAAACATCATCTCAT
TGAAAAATTAAAGCTATTGA
ACTAACAACTTGAAACTGACTTTTTTTTACCGCCCTTGCACGCTTGAACAAGCTGCCA
GGATGTTTTCATAGCAACGTTT
AGTTATGATTATCTCTTCATCTTTATTTTATAAATCTCGACAGAAATCTATGATACCTCTG
ACCCGTTTTCATTAAACCC
TAAACGGAAAAAAGTACCTACATTAATGAATTCAAATGTTATCACGCTAATGTTGTTAA
AACTGATGAAAGTCAGATGAA
GTAAGATAAAAGTGTGTCGGTATTGATAAACCTTTCAACGTGAATGTCTATTTCATGCG
ACAAATGTGTGTTATTTTTAT
GAACTAGTTGGGTCATGTGCGAACAAAGTTCCTTTTTTTTTCTGTCTCATGTGGCAAAT
GTGTGTTATTTTTGATGACAT
AGTTGGGTTATCAGCCAACAATAGCAATTTATTTTTGTCCAAGTCTATTTTAGCTAAAGT

ACATTTTTATATGGGAGATG
ATTTGTACACTCTCAAAAGTGATTGATACACTCAAAATAATTGTGTTTGGACATACCCTC
ACAGGAATGAAAATATAAGT
GTAACAATGAGGGTATAAATGTCCAAATACAATTAATTTGAGTGTACCAATCACTTTTGA
GAGTGTACAAATCATCTCCC
TTTTTATATATATGAAAACATCTTCTAATTTCTGGTTACCTAACGAGAAACAATCATATTC
ATACATTTGAGATGTATCG
AGTTGAATTAGATGCCTATTTATTTATTTAGAGGCATTGCAAATAGTTTCGAAAGTGATT
TTTAAGTGAAAACGATGCAC
ATTAATACTAGCAGCATATTTGACGCGCACATATGACAAAAGATATATACATCGCCTAAC
TTGATGAAATACATCTAATT
TATGTATGAGCATATAATTTTGAGCTAGAAAATATAAGATGCATCAAAATGATTAGGGAT
GAATTTAATTCATGTTGATA
TATCGCTAATTTATAGTTATAGTTATTTCTCATTATTCTCATTTTAAGATTTGTCCCCGTTT
GAACCGAACATACCTAAT
GTAATGTGGCCCTAATAAAAGTATGTAGATTACCATTTTAATAACTATAATGGCAGATATT
GTTCTTAAGTATATGGAAG
TAATAGTTTAAGCTATTTTATCGTTGAAGGGTTGTCTTTGGTTTGCTTAAATAAACACCA
TCAACTTATACGAAGGAAGT
AATCTACTATTTTCATGGGTGTCCACTAATTTCCTCATACATTCCTCCCACTCCTTATTTC
CCTCCTTACCTCAAATTTT
CTCTATAAACTCTTCTCACCATCTTCCCAAATCAATGTGACTCGAATCTTAACAGACTCA
TTATCGTTTCTTCGTATCCT
CTTCGTTAGCCCCTCTCCTTACCTGCCCGTTTCACTACCCCTCTTCCATGGATCTCTCTG
AGATCCATCACTTTTTTCAA
>CsCML77
TTTCTCAGTTTTTATATTTTGATCGCAGATGTGTCACAAGCATGGTGTTATGCATCGTGA
TCTTAAACCTGAGAACTTCT
TGTTTGCAAACAAGAAAGAAACCGCAGCCTTGAAGGCAATTGATTTTGGGTTATCTGT
TTTCTTCAAACCAGGTGCAAAC
TTTGAACATGACCTATTTATATTTGCTCTATAAAACATTGATATGGCCTTATAACTATATTT
GTTTGCACTAAATGAAAG
TCTTTGTAATCTTCTACAGGTGAAAGATTTAATGAAATTGTAGGGAGTCCATACTACATG
GCTCCTGAAGTTCTGAAACG
GGACTATGGTCCTGAGGTGGATGTATGGAGTGCTGGAGTAATCTTATACATTTTGCTTTG
TGGTGTCCCACCTTTTTGGG
CAGGTCAGAACAACCATAAATCTTTAACTTCTTTTACAAAATGATAAAGTCTATACAATT
TATGTACAAGGGAACAGCTC
TAACTGTTTAAAAGGTCACTCATAGTGCCCCTGAATTGCCTTTTTATAGTTCTTTATCAA
CTTTCTTTTCTTTTCTTTTT
TTTTTCTTTTTTTTTTTATCATATTAAATATTATAGTAGTAATTTTTCGTATGAATCTCAAA
AAAGGGGAAAAATGCATA
CTGAACTGGTCAACCCAACTTGACGGGTTACCTGTACCATCCATTTTGCCTCCTATTATA
AAAGTTTACCACTTAGGACA

TGTTAACTGTTCCAAACATGGGGTGGATGTAATAAGATCAGAGCAACCTTATCACAACA
TGATTTTAACATGCATAGCTA
ATCAATACCCGTTGTCTTATGGTATGGAGTAATATGTAAACATATAACGTATTGCGATCAC
ATGTTTATTGAGGTTTAAG
ATGATGTGCTTCTAAATATTTTGCCATCTTAATAACAGAAACTGAACAAGGTGTTGCAC
AGGCAATCATTCGATCAGTTG
TAGATTTTAAGAGGGATCCTTGGCCCAAGGTATCTGATACTGCAAAGGACCTTGTCAAG
AAGATGCTCAATCCAGACCCG
AAGTTACGCCTAACTGCTCAGGAAGTTCTAGGTGCGATACAGATCATTATTTTTTTCTC
ATCATATTCATCTTTTATGT
TCAATATACTCCATTATAATGGGTTTTGTTCAAAATTTCTTAATCTGTTTGCTTTTCTTAA
ATTCTTTCAGATCATCCAT
GGATACAAAACGCCAAAAAAGCTTCAAATGTTTCGTTAGGTGAAACTGTTAAAGCAAG
ACTCAAGCAATTTTCTGTCATG
AACAAGCTTAAGAAGAGAGCTCTAAGAGTAAGAACATGCATTCGTTTTTGAGACAATG
CATCATTTATTATAATATTGTA
AAGCTTTTTCTGGTATATTTGGTTAATCTAAATTCTCTATATAACAAACTTGCAGGTAATT
GCCGAGCATTTATCTGCAG
AGGAAGTGGCGGGCATAAGGCAAGGTTTTGACTTGATGGACACGAACAAGCGAGGGA
AGATAAATATTGTTGAGTTGAAA
GCCGGGTTGCAGAAACTTGGGCATCAAATTGCTGATGCAGATCTTCAAATACTTATGGA
AGCGGTAAGTTTCTAAAGTTG
ACTTCCATGAATTTCTTGTTTTCGAAATGCATGAGATCAAATATATAATTTGTTCAGATC
ATGGAAGTTAAGCAAATGGC
GTTAATTATCAATAGAGGTGGCAATTTTTACCCATTTACTTATGGATGGGTCGATGCAGT
TCGTTTTTAATCACTAAAGG
GTCAAACATGTCAAATAAATACGTTAGTTAACAAGAAAACGGATCAAAGTGGGTTAAA
GTCACCTAAAGTGTATTTTAGT
TCATTTTACCGTCTAAATCATATAGTAAAAAATTGAGATGATGTGTAACAACTACTGTAT
ATAGATGTAGTAATGAAAGG
TGTTACTACAGGGTGATGTTGACAAAGATGGATTCTTGAATTACGGAGAGTTTGTTGCA
ATATCAGTTCACTTAAGAAAG
>CsCaM4
CCACTGATCGGGCAAGAGAGGTGAAGGAGGATGATTGGGTTTTTGCCTTGTCGCTCAA
GCTGTGATCCAAATATGGGGGT
CGGTTAAGTTTGCTATAAAAGCAAACATACCCCATCCAAACTCACGACTAATATACATC
CTCACCTCATGCATTGTATGA
CTTACTGATTATCCCTAGACTTAAAAAAGTAGTCGATCTACTTGTGAACTCAATCGGATT
CTTTCATATTCGATTAGGGT
TTATATCAAACCCTAAACCTTGCAATCTTTTCCAAGTGGTTTTATTGTTTGGTGTGTTGA
TCTCTTAGCTCTATAGTTCC
CTGTTGAATCGTATTAGCAACAAGTTTTGGGTTTTTCTGTGTTCGCGTTTCTCGGACAA
AGAATGTGGGATATCGGATCT
CTTCATCCGATATCGGGTAGAAGAGAGAAACAACCGTTTGGTTGTTTTCTCTTTCTCGT

ACAGAAGGAGTTTTGAGCAGG
AAGCAACTATTGACTTGGGTGGTTTTCTTGTTTGAATTATTCAAGTGTTGTCAATTGTTG
ATTGGTTAATTACGTTCTCG
ACTACATTTCTTTATCGAACATATATGTTATCGGGTTTGTGTATGATACTCATAGAGATAT
CTAATTTAGATATTAGGGT
TTCGTTTGGTTTTCTTGTTCGTATACTTGAGTTCAATAAGCCACGCAAGGCTAGGTTTCT
ATTTATCCCTTTTATTCATT
AATGTTTATTATTACGTGTTTAACCTAATTGTGATTACTAGATCCATATCCGCTGCATACT
CGTACATGTTAATGGTTAA
CTTAGTACTAAAATCTCAACAATTTCTGCGCACCTCACGTTTCTGCCTTAGACGGGGTT
GACAGTAGGTGCATTCGTTTA
CTTATTCCGCCATATAACTATTTGGCCGAAGTTAAAAGTTTAAAAATCACATTTACCATT
AATTTGTTGAATTATTTTTA
CTATTCACATTCTTCTAAGAGGGCGCGCTGCAGTAAAATATAGTCACGTTATAGGGCCAT
TTTGGTGCGTATCCCGAAGC
CATGATTGCCATGGCTGCGCCGCTTCTTTGTGTTATAGGGCATTCACTATGGTTACAACC
AGGGACGGAGCTTCAACAAA
TCTCTTGGGGGGGCCGAAATCATGTGAAATTAAGCGTATAGTTTTATATATACACGAGA
ACGTACATATTTTACAATTTT
TAGCCAACTCTACTTATGTAATGGGATCAAATCAGTAAGCATTGCCTGTTATTCAATATG
GTAGTTAGATTATAGATAGA
TAATACAAAAAAAAGGCACTTTATCACTATAGATATTAAGAAATTATATCAAAGTAACAA
TCGGATTAGTTAGATAAGAA
TTGAAATATTAAAGTAGATTTTGTTTTAAAAGGAATGCAAACCAATGGCTGAAAAAAG
AACGTGGAGCAAGAAAATAAGA
AGTGATTGTTTCTTTCCTATTTTTGAGGGAGGGCTAGATGTAAATATATTCTTTAAATCCC
ATATATATCGAACAAAACT
ACCATAACTACCCGATTTTTTGGGGGGGCCATGACCCACCCCTGCCCTCATAAAGCTTC
GTCCATGGTTACAACGGGAAT
TATCGAGTTATCTTTTCTTTATTTTTTTTAGTGTAAGATAAATGAATATTGTGTCTCTCGT
GTACTTATATATTAAGGAG
GGGTTTTTTTTCTTTGTGTTACAAGAATGTTATACGCTGAAAGTTGACGAGATAATGGA
CCAAAATAAACGATAAAATAA
AAGCAAAATGAGGGTAAGGGTAGAACGGTGAAATCGGATTGATAAAAACAGATCACA
TCCGTCAGGATTGGACCACCACT
CACTAATATTTAATTGCAATCGCTTGTATTTAACGCGACCCACGATTTTCATTTCCTAAA
ATAATATCCATCCATTATTC
ATTTTCCCAAAATACAACCCTTTTTTTCCTTCCATTTGCTCTCGTAATTTGTTTTTTGAAA
ACCAAAAAAAGAAAAGAAA
>CsCML78
ACATTATCTGACATTAGTCTGGCGTAATACATTTTGTCTCATATTTCATACGCTTTTTTTG
TAAATCTAATTTAGATAAT
ACTTCCTAGGCTATGTTTTAGTCTTAATTTTGGCGTGGCAAAAACCGCGAACCATAAAA
TGCAACTTTGATTTGTACATG

GCTTGGTATATGAGCCGAACCGCAAATATTCTTGGTACAATTCTTAACACACTACTCTCT
TTTGTATTAAGCCCATACAA
AAAATGTCACCTGGCCATAATAATGTCCACGCGAGGGATGTTGAAGAAAGCGATACAA
ATTTTTTGTGTGCGTTTGAATG
AGGGTTTTCGTTATGAGGCCAACAGATTTGTTGAGGTATGTTATTTATTGGTTTTGTCTA
ATTTTAATGCTTCTGCTAAT
AGTTTAAAGCATAACACATTAGTCTAGGGTGTTACAGTTTTCCAAAATTTCATGATCTGT
TTCGTAAATCTACTTTAGAT
AATAGTTGCTCTGCTAGAAGATTTGGGTACCCGGTGTATCTTAATTTTGGCATGACAAA
AAGCACGAAGGCATAAAATGC
AATTATTTTTTTGTAGCCAATACACTTTATGAGCCAAGGCATAAATATTATGTCTCCAATC
ACTAAAGCATTTTTCATTT
TTCTATTTAGATTATCTAGGATCCGTCAAATGGACATATTAATGTTGAAGTAAGGGGAGT
GGAAGAATTCGATATAAGTG
TTCTTTATAATGCTTGCGTGGAGAAATTGGTTCATGAGTAACTCCTTATCCGAGCTTCTT
CATGACAAAACATCTCCTTG
CATGCTTACTAGAAGATTTTTATATCGATTTCTTACATACTCCCTATATCAATGTTAGTATG
CCCCATTTTACATATTGT
GCAAACTCTAAATAGAAGGTAAACAATGTCTTAGGTGACACGTTTGTTGAGTCGAGCA
ACCTTATTACGCCAGGATCACA
TACAAGATCTATTTGACTTTGAGTTGTTGGAAGTTGGAACATCTAGCAGACAATTGTGT
ATTGAGAAATAGATAGCCCTA
AAGATCTTCCTGGTAAACATGGTGTTCTAAATTTATATGTTTTTGCATATTTTTACAGTTT
CATATGTACTAATTTGTAT
ATCGTAGAACCAAAAATATAATGCATTCTATCTATGATTTTGTAGGTGTAGTAATGTTTCA
CACTTTCTGATAAACGGCG
ATTTTCAAGACAATTTATATGAGACACAGAATCAGTGGCAATCGAACTATTCAGGTTAC
TTTCTATGCAGTTATTCCAAA
TAGTACTAAAAATAAATCAGCAGGAGCTGGAGCCATGAAGAAATGAAAACGGTTCTGT
GTGACTTTATATGTATCTTGCA
TACAAAAACTAAAACCAATGAAAATTGAAATCAATTTTGTATAATTTTATGTGTAAATAG
TTGTTTTCTCTTCTGCTTTT
TTATTATCAAAAAGTCTCTTCTTCTATAATAGTATCGTATACAAACACTTCTTTACAACGC
ACGAGTTGTAATCCTTATT
TATAACAATAAGTAAAGTACGATCTCAATTTAGTTTTCATTGTGACACTAATATGACAAT
GATTCTCTATATATAATTCT
CATCACCACATCTAAAAATGCACATCTAATAAACTCCAAGGTCCAAAACTTACTTCAAC
AAATAACCAAAGGACACCACT
TTTAATTTCCTGAAATGGCTAAACAAAAAATCCGTATGAAAGAAAGAAACAAAACAAC
TAGCCGCATGAGTCACATCAAA
ATAATCAAAAGAAAACAAACAAACAACCCAATCAATTAACTTAAACAAAAGTCATTAA
TATTCCATCATAACGAAGCTTC
CACGAAGCTCACTCTCACTCTCCCTTTTATTCACCTCCTCCTTCCTCCCTCCTTCCCCAC
ACCTAAACACACACTACTTT

TCACACACACACATTTCTAAACACGCACTGAACACACGCTTTTTCACACACTAAACAC
ACACTTCTTTCACACACAGATA
>CsCML79
AAAAAAGCATGTGTGATTTACAACTTTTTAAATTAAAATAGAATCACATGTGTTTTAGTT
TACAACAATAAAAGTAAATC
ACACGGATTTACATTTGTTAACTCGTTGCAAACAGTTGTGTATACATTTGTTAACTTAAG
CTAATTATTGTTTTTTCCCA
ACATAAACAGTATTTTTTTTACATCAAATCACATGCGATTTTACCTCAAATCACATGTGA
TTTACCGCAAATCACATGTG
ATTTACCGCAAATCACAGTGATTTTACATTAAATTATTTTTTAAAGTGAATCACATGTGA
TTTACACATTTTTAAATTAA
AAATGAATCACACATAATTTACATTTTTTCATAGCAGAGCAAAAAAATAAATAACAGAA
TCACACGTGATTTCAGGAATT
TGTTTTATAAAATGAAGGAATAAGTTGATTTGAAATTAAAGATCACATGTGATTAATTAC
TTTAAATATCGAATGTGACT
TGTAAAATCCCATGTGATTCTCTGGTGGATCACATGTGATTCTTTTTGGATCACTTGCGA
TTTTAAAAAATAACAAATCAC
ATGTGATTTTTTTTTACGGATCACATGTGATTTGTCTGGACGGAAATTTTGAATAAAAAC
TTCATAGAAAGTATATAAAA
GTGCATATCTATAGAAAGATGATTTTTTTCTCGTCGCAATGGTGTATTTTTTGTAGCTTGA
AGATTTACCGTTTGCAAGA
TATGTTGTTTTTTAAAAAAAGGTGGAAAGTGGGTTTTCATAGAATTACATTTCTACCCTT
CATTTGTTAATTTTTGCTTT
CATCTCATCCATTGGATGAAAAGAAGATGGATGGTTGAGATCTTATGATTTGATCTTTAG
ATTAGATTTGTTTTCTATTG
ATCTCACCTCATATGTAAATAGGATGTATTAGAGAGTCAATTTCTTTTGGCTTTTCTTTGG
GGTTGTATTATAGATTTAT
AGTATAACCATTTGTTTGAAACTTTCAAAAGACATTAAAGTGATTTTTTTGTCAAAATA
GTTGAAAGTCTATGAAATTAG
GTAATACAAAGAGGTGAATAGTATAATTTATTTGCTCCAGTTTTGATGAAATTGGTGTAA
AGCTCATGGCTAATAAAATA
CTTATTCAACCCTAAATAACGAGATAATCAAAATCAAACTTATTCACTCCAACAATAAA
AAACTAGTTATTTCGACTTAA
ATCATAATAATAGGATTTTGATAGTGATTCAAATAATTCCAAATCCAAAAAGCGATGATA
TTAAAACTTGTTAATCAGCT
TGGCCCAAAATCCTTATTATGTATTAATTGAGTGAATCATTAAAACTACACTTTCTTATAT
AAAAGGCTAATTAAGACAG
TCATATGTAACCACTTAATTTAGTAAACACCCCAATGTAATAGGAAGGTCACCCACGCA
TTGTGACTTAAATGCGTCTTC
ACTAAAACTTTGCTACGCACCCACGCGTGGTTTGATCGAAAAATAGGAGAAGTCGGTC
GATAAACCAATCGACTTGTGAA
TGGTGTTTGTGTGTGAATTTAATTTTTATGAGGTATATTTGTGTATAAATGAGATTTGTTT
AGATGGCCATGTTGATGGT
CTTATTACACCTTGCGGTGATGATATTCGTAGTGTAGTGAAAACGTGATGTAACGCTGA

GTTGACATTGATGATGTTGAT
TACTACTAGACCAAGTCTAATCTTAACACCCTGTTTTACGTTATAATTTGCATGGTTTTGT
TTGACTTAATATGAAAACG
AATAGTCCCTCTTCCGGCAGTTTTGGAGACCACGGACTTGGTCAATAAACCAACAAAA
AAATATCAAAATCAAGTGAATG
TGCCGGCAATTTCCTTCCTACAACTTGGCTTACTATTATTGGGAACTGCGATGAGCACC
CATACTTTCATACCATATATA
CTATACCAATCCTATCACTCTATCAACTAGTAACATAGACTCGATACTATAATGTACATGG
TGTACGAATACACTTCTCA
>CsCML80
CACATGTAATTAAACAGTAATTTGAATATCACATGTTAAATTAGTGCTAAATCACATATG
ATGTGTAATATCACATGTGA
TATTAGTGTTAGATCAAATATGATATACATATCATATGTGATTTAACATATCACATATAATAT
GTAATCACATGTGATAT
TAGTGTTAAATCACAAATGATCATATGTGATTTAACACTAATATCATATGTGATATTCAAA
TTAGTGTTTAGTCACATGT
GATTTTTTTCATGTAAATTTCAGTAACGAAAAAAAATGTAGAAGAAATGAAAAACACG
AAAAAAAATTTTGTCGATAGTA
TATCAAAGTGCCTATCTACAGAAAGATAATTAAATGCTTGTCGTAATGGTATATTTTTTTT
GGTTTAACGATTAACGGTT
TGAAAGATATTAATATTTGAAAAAAAAAGGATGAAAGTGGTTTTTTTAAATTGTTTTGA
TTAATCTCATCAATTAAATCA
AATAAAAATGGAGAGAAGATAGAGGTGGAGATTGGTCCCTAAATAAGTAAAAAATGTT
TTGTCTTCAAATGATCTTAAAT
AGCAAATTTATCATGTTCTTTTAGGAAATACAAAACTTTAAAATTTAAAACACTTAATTA
TCATATATTTTTTTAAACAA
CAATTTTTTCACACCCTATATCAAATATCTCAAGTATATCTTACAAATGTTTCTAGTTAAG
CTTAAATTCACAACTTTTA
AATTGATGGACAACTCACATATCACTAAACTAAACGTTAGTATCTCTTAGTCGCGATAG
GTCATTCAGATACCTTGCACA
TAAACGTTTTTAACCACATATCTAAACACTAAACACCTTAATATTAACAGTGTGTTCTAA
AAATTAAAAAAAAAAAAAAGA
AAAAAGAAAAAAAAAAATGGTTGTCTTTCATCCCAAAGTGGGGTGAAAGTTTTGAAAG
CAAAATCTTCTCACTTTCACTTT
TCTTTCTAAAAAAGAGAACACAACTTTTATTACTTTTTGTTCCTTCCCCCCCCCCCCCCC
CCCCCCCCCTTTACTCAACGT
GAGAACACACTGTAAGTGTAATATGTCCAACTGTAACAACGGACTACCACCCCACCAT
CATCGTCGTTACCCCCTCCTTT
TGTGAGAAATCGGTGGTGCAGACCACCACGTCGTAGAAGCACAAAATATTACTTGTGT
GTGGGAGAGAGTATATTGTGAG
GTATATTTGTGTTTAAGTGGATATATTTTGGTGATTAAGCTGGTAGTCTTGTTACACCTGT
TTGGTGTTGAACTCAGTAA
TGAAGAGAAAGAAAAAATGGTTTGGCATTAGGTTGACAATGGTTAATTCGATGTTGAA
TATAACGTTTAACTATTTAAAG

CACCTGCGTAGTGTTTGGTATGTTGTAATGAAAGCCGGAATGAAAAAGATTATTACGAT
GTAATGAAAAAAAATGTTGTT
TGTTTACATTTATAGAATGAAATGATTAATTACAAGGAATGACCCATTACACCAAAACCA
TTTCTTTGAGGTCCCCAACT
TTTTCTATTACATCTTATAATGGAATCAAAATTACATAATTTTTCTATTACATCTTATAATG
GAATCGAAATTACATAAA
ACTCCATCATTAACTAACCACATTAAATAAATTCCCTATCATCAATGGGTATTTTAGTCTT
TTTTATTATCGATTTATTA
CGTCTTATCATACAAACCAAACAAGATTAATAACGACAATAAATCATTACATTACACTAT
TTATTCATTACATCATTTAT
TTTATTCATTTAATATTTTATTCCGTCCTACCAAACGAACCTATGATTTATTCTAGTTCCAT
GCAAATTTCACCATAAGT
TTGGAATAGAAACTTATGTTCAAAAAATTTACCTTCCCTAGCCCCCCAACCCTATTGAC
AAGTTGTATATTGTCATCCAT
ATTTCACATACTTCTCATATTGCTATAAATAAGTGCATTAATTCTTCCAATTCCAACCCAC
CACCAAGAAAAGAAAACAA
>CsCML81
CAATGTCCTGGGTCAAGGAGAATCGTCGAATACGCTTAAGTAAGGTGACTGCATCTTTC
TCAAGTTCTCCAAAAGAAGAA
AAGGAGAATGGAAGAAACCCATATCCAATATCCGCACATTTAGCATCGTATTTGACCCG
TTTACGCTGCGCAGCATCACT
GATCGCTCGCCCTGGTATAAAATCAATCAAACCGGATTGCGTCAAAGGCGACGAACCC
GTAAGATCAACACATACGTCGC
GTCCACTATCCCAAGAGTAGAGTAATATGTCATCCGGACGCAAGGATCTGTCCCCATCT
CCAAAAAGCCCAATATCCACC
TCCTTACGAGCAGAAATCCCTGACCGAGAGCATATATCAACAAGAACATCGCGTACAA
GGTTGTGACGATGTTTAATGCC
AACAATACCCGCACATGAAACAGCGTGGTCCCCGTAAATATCTCCAGGAAAAACCTTA
GAGTAGGCAGAACACGGTACCT
GAACAGTGAAAAGAGGAACGCCCAAACGATAACACAATACACATCGATAAGCCCTAG
CGTTCATAGTTTGCCCCAATCCA
GATATCGGAGCCACCCTAAGCCAATCCGAGGTGTGATCCTCCTTTAAAGAGCTCCATAA
CGCCATCTGTCGGGAGGAAAG
ATGGAAAGTAGACTCAACAGATTGGGTAACCCGATCGAAATATATGTCTGCCAATTTCT
TCATAAGTTTGGGGGCAGCGA
CCTCGCTAGGGTTACTCAAAAGGTCAATATCCATATTCACATTAAAAGCACGTAGGGCA
TCATCAAAGGAGGGACCAGCA
GTTACAATACCCGAACTAAGGAGAAGCTTAGACTGTAAACTAGCAGATTGATTATTATT
ATTATTATTATTATTATTATT
TAATTATTTGAAATACAAAAATTGATAGGTTGATATCAAATCTTCATATCAACGTTTGTTT
TTTAACCTTTTCTTATAAA
GCCAGTAACTCAAATGCTAATATCAATTTCCTTTTATTTATTTTTTTATTATTATTATTATTT
TTTTTATTGTTTGGGGT
TCTTTAAAATTCTCCACAACTATTATATATAAATAATATTAGAATTAATTTTCAATTGTGCA

CATTATTACGAATTGCAC
CCAACAAAAGATCTTCCAATATTGCTTTTGCGATAGAAGGTTAGCATATACTCAGTGTAT
TTAAGGTAGCAATTTAAATA
ATCATATTATTTTAATGATGATTTAGGTAATCAAATGTTTAAAACGAAAATTTAAATGATA
AAATTCATTTTTATTCATG
TATTATATACAATAACCAAACAAGAGAATGCTCTATATCACACGCATTATTTATTTGTACC
AAACAGTAGAATCGTTATT
CTTTCTCAAACATGATTCACTTTTTCTGCAATTCCATTCTCTATTACGATTTCATTCTATG
GCATTTTCTCGTACCAAAC
GTGCCCTTAGTAGACTCAACCATTAAACTAATTGACAAAATTTGACCGGACACGTAAG
AAAACAAAAGAAATAACAAATT
TAAAAATTAAGTAGAAAGTATTTTTACCGTTAAAATAAAGTTTAGCAGGTGAAGAAAA
AATAACAAAATTTCGAATACCG
AATTTCTAATATCGAAACGAATTCAAATGTTTTGCTAGCATTAATCTTATCTTTTTTTCCC
TGTTATGGAACAATCTAGA
AATTAATTGTATATTCAATACGCGGGGGACCAACATATCAAGTTGGCAACACCTCTTAA
CTTTTTCTTGCCAAACGTCAC
GTTTTCCTTCCTCCACATTATTACTATTAAAAATATCAAAGCTTCCTAGAAACCACAATA
CGCGAATTATCTGCTGTACC
CATCCTTCTGTCTCCCCGATGGCTTTTACTTCATCTTCCAGGAAAATACCCTTTCTCACC
CACCTTCCTATATTTATATA
CTTTGATCACTTCATTTCCAGTATCATACAAACTCAAATACCCTACAAACGCAAATTATA
ATACTAGCTAGCTAACATCA
>CsCML82
AAAAGGAAATTAATTATTTAGAACTTAATTTAAAACATTGACATTAAAACGACATATATG
GAAATTAAAACTAAAGACTA
CTCTTCTCGCTTGCTTCTCTTCTCTTATTTATACATCAAAAGGCATCCCACTTGATTTCTG
AGCCAATCCCACTTGTTAT
CACAGCCATTGGATCAGTTGGGTCATTTTTGGCCATTGATGATATTTGGCGGGAAAATG
GGATATCAACAGGTGGAAGGA
TTGAGTGCAAGGGGGGGAGCATTTAATGTTTCTTGGCTTGGGGTGTTTGGAGGGTAGG
AATAAGGTGGGTGTGATGATGA
TGGAGTAGGGGGAAGAGTTGCAGGTGTTGCAGGCAAGTGGTGAGGGAGAAGATAAGT
TGGAGGGAAAAGTGGGCGGTGGG
GTTTGACTTATTTGACTTGGGGGGGGGGGGGGGGGGTTTAATATTAAGGCAGGGGTC
AGAGTGTATGGCAGGGGTCAAA
GGGATATTGGCAGGGGGTTCAAAAAGGTCCAAAGGGTCATGGCTTGTATTAAATAACA
AGGACCAATATGCAATTTCTGA
AATTTGGAGGACCAAAGCTGCCAATTTCTCAAAGTTGTTTCAAGGGTTACTTTTGCCA
AGTTTCAATAGTATAAGGACCA
TAAAAGATATTTTCAAACTTCCAAGGACTATTTTTGCCAATTTTGAAACTTGAAGGACC
AAACTTGTAAATTCTCAAAAC
TTTTGATATTTTTCACCAAAGGACTAATTATGACAACTTATGATTTAAAAAGGACCTTTG
GTGATTTTTCTTTTAAATTA

AGTGCTCAGGGACTATTTCTGACAACTTTTGAAACTTGGGAGACTGAAACTGCCAATT
ATGCAAACTTAGCTCATTTTTC
ATCTGATTTTAAACATTTTGCATCTGAAACATATAAAATGCAATTTTGAGATATTTTCAGT
ATTTTGATGCATTTCTGAG
CATTTTTAAATGTTTTGCAGTTCAGATTTAACAAAAAATGATGAGTATTTACACACTCAT
CAAATACTCTCATCCTTTTT
TTAAAAAAAATTAATATATCACAAACCGTTAATCGTTATGCAAATGAACTATACCACCGC
GACGGGAATTAAATCATCTT
TCTATGGACGGACACTTCGATATATTTTCGACAATTTTTATTTTTTATTTTTTTCAGTTAC
GTCTGGAAAAATCACATCT
GATTTGTATCACAAATCAAATGTGGTTTATGAAATCACGTGTGATTTGTGATACAAATCA
TATGTGTTTTTTCCAGACAT
ATCTGAAAAAAAAAAAAAAAAAAAAAAAAAAAAAAAAAAAGTTCGTCGAAAATTTAT
CGAAGTCTTCGTTTATAGAAAGAT
GATTTAATTTCCGTCGCGATAGCGTATTTTTTGTCATTTGACGATTTACCTTTTGAAAGAT
ATTTTATTTTTGAAAAAGA
AGTGGAAAGTGGGTGGTGTTTTTTTTTTTTTTTTTTTTTTTTTTTAATGAAAATATGAGT
GACTAAAATTAGTCTATTGA
ATCTTTAGATAATTTTAATTTTACATTGATGTCAACTCTATCATTAGATATTTAGATGGTAC
TATGAGAAAATAAACTCA
AACTATGGCTATCACCATGCATAAAAACACAACAAAGTTGATAACATGTTCCATGAAAT
ATTGCGGTATATTTTATGTAA
AACCATATCTAGGAGTTACATGTAAATGTTACATGTCATTTGTAATGACAGTTACCGTAA
TAAATTCATATCCATTGGAT
CCAGATTCTGCCAGCAATAAATTCAATTATTGTCTCATAATTTTCAAGATCTCGATCTCTA
TTCACCATTAATTCAACCT
TCCTAAACTTAGTATAAACCTACATAATAAATCTAAATTCATCTCTTATAAACAACATTTC
CCTCAATTCAAATTCAATC
TTTTCAAATACAGCAACATTTCGAAAAGATTGAATTTAGTTTCGAGGGAAATATATATGA
TGGAAACCAATGTTAATTCA
